# Tunable Dynamic Excimer Formation in Bisphenalenyl Derivatives through Molecular Packing

**DOI:** 10.1021/acs.jpca.5c07790

**Published:** 2026-03-10

**Authors:** Gisselle Y. Rojas, Domenica R. Fertal, Isabelle A. Herlinger, Mark S. Chen, Lisa A. Fredin, Elizabeth R. Young

**Affiliations:** Department of Chemistry, 1687 Lehigh University, Bethlehem, Pennsylvania 18015, United States

## Abstract

Dynamic excimer formation in solution-phase π-conjugated systems presents a promising route toward tunable photophysical properties, yet precise control over these transient species remains limited. Herein, a series of bisphenalenyl derivatives is shown to exhibit excimer emission that is modulated through strategic tailoring of side chains (ethylphenyl, *n*-butylphenyl, and *n*-hexyl). Two phenyl-substituted derivatives exhibit reversible, concentration-dependent excimer emission consistent with excited-state dimerization. In contrast, an aliphatically substituted bisphenalenyl moiety displays exclusively monomeric emission. Steady-state and time-resolved spectroscopy, time-dependent density functional theory, and diffusion-ordered NMR spectroscopy are employed to confirm that excimer formation arises due to excited-state encounters, with no evidence of ground-state aggregation in acetonitrile. However, diffusion-ordered NMR spectroscopy data reveal dimer formation in tetrachloroethane. Notably, the introduction of substoichiometric molar ratios of HBF_4_ induces excimer emission at even lower concentrations of the bisphenalenyl moiety, demonstrating a route to stimulus-responsive control. These results provide a structure–environment framework for modulating dynamic excimer formation in charged π-systems and inform the rational design of responsive fluorescent materials.

## Graphical abstract

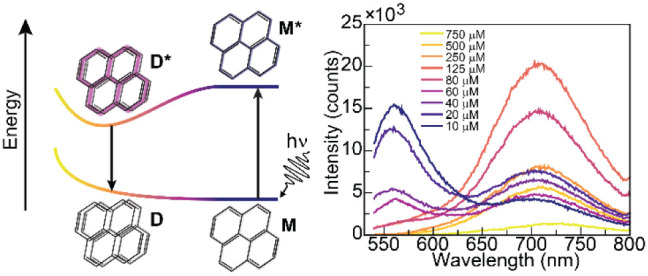

## Introduction

Conjugated organics are critical for cheaper and more flexible optoelectronics,^1,2^ biosensors,^3^ and fluorescent-based switches.^4^ Many of these applications use the inherent intermolecular forces of the π-systems to drive supramolecular or solid-state structure. A fundamental challenge in designing π-conjugated systems is that small changes in their structure or environment can significantly impact their supramolecular properties.^5–7^ In solution, such changes to molecular structure can lead to the formation of static or dynamic dimers or higher-order aggregates arising from their propensity to electronically couple to one another.^8,9^ Aggregation typically results in spectral shifts in the absorption or emission spectrum, altered fluorescence intensity or spectral features, or quenching of excited-state lifetimes.^10^ These properties make it possible to imagine harnessing the ability to use the same molecular building blocks for a range of functions.

Ground-state π-aggregates are formed as a result of ground-state intermolecular forces and typically lead to concentration-dependent absorption and emission, for which the spectral shift is influenced by the π-stacking orientation. For example, J- and H-aggregates lead to a red or blue shift, respectively. When preassociated dimers (D) that exist in the ground state (Figure 1, right) are excited, they form static excimers.^11^ These static excimers are excited-state dimers that exhibit electron densities delocalized across both molecules, impacting emissive behavior.^12^ An excited-state dimer (D*) can then emit as it relaxes back to the ground state of the dimer. The formation of static excimers is concentration-dependent, allowing the absorption and emission to be controlled by concentration. Static excimer behavior is commonly observed in systems such as benzene and pyrene.^13,14^ The static excimer dimer geometry is driven by ground-state π-stacking. When the ground-state dimer is excited to form the static excimer, the delocalized π* orbital often has a node between the two monomers. Therefore, static excimers often exhibit weaker monomer orbital overlap in the excited state, leading to relatively short-lived excited states and moderate fluorescence efficiencies.^13,14^ In contrast, dynamic excimers are formed between a molecule already in the excited state and a monomer, allowing each to reorient upon collision, leading to better monomer orbital overlap in the excited state.

**Figure 1:**
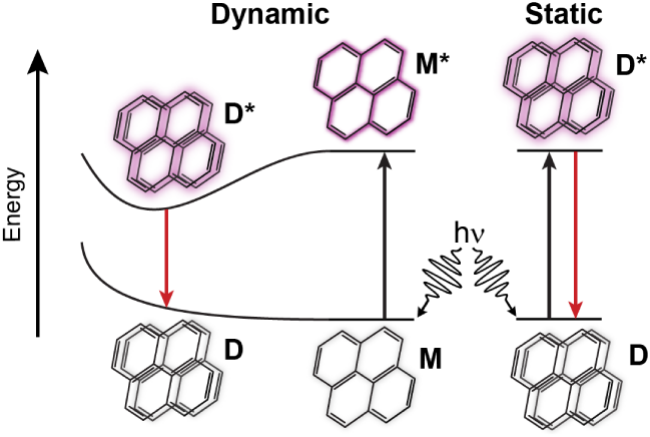
Cartoon mechanism clarifying the difference between dynamic and static excimers. The monomer is represented as M in the GS, and the excited monomer is M*, followed by a local minimum that is driven by π-stacking of M and M* due to strong intermolecular interactions to form an excited dimer, D*. Relaxation of this excited D* forms an unstable GS dimer, D, through a dissociation step and then forms M in the GS. The static excimer starts with a dimer, D, in the GS, and through photoexcitation, an excited dimer, D*, forms.

While most organic moieties aggregate in the ground state, a distinct class of excited-state associations has attracted growing attention due to their pronounced influence on photophysical properties.^15^ These dynamic excimers (Figure 1, left) form dimers only after photoexcitation.^16^

Excitation of the ground state (GS) monomer (M → M*) followed by a collisional encounter between M and M* results in an excimer. This dynamic excimer (M–M*) is stabilized by π–π* orbital overlap. After radiative decay, the unstable ground state dimer (M–M) readily relaxes to regenerate GS monomers (2 M). Unlike their static counterparts, dynamic excimers are not preassociated in the GS, making their formation highly dependent on concentration, solvent polarity, and molecular diffusion in the excited state.

While dynamic excimers are less common, they present a compelling target for rational design.^17–20^ Dynamic excimers are known for their large Stokes shifts (>100 nm) that mitigate self-absorption and enable more efficient detection of emission signals.^21^ Excimer emission is typically red-shifted, meaning that it can be beneficial for red and near-infrared (NIR) applications in biological imaging, where deeper tissue penetration and minimal background interference are critical.^22,23^ The ability to harness tunable and reversible excimer formation that is driven by the solvation environment would provide a spectrally specific emissive sensor^24^ of complex environments. In addition to monomer concentration, dynamic excimers are responsive to external stimuli, including temperature, pH, polarity, and viscosity. The strong intermolecular interactions driving dynamic excimer formation should be tunable such that these interactions can be strengthened in order to induce dynamic excimer formation at low concentrations, affording a powerful platform for designing high-sensitivity environment-dependent emissive probes.

Structural modifications of bisphenalenyl derivatives (Scheme 1) are synthesized that enable reversible, concentration-dependent dynamic excimer emission in solution. The molecular design builds upon prior work that showed strong intermolecular forces caused GS aggregation of 1­[Ph], effectively quenching photoluminescence (PL) in solution.^25^ To achieve dynamic excimer behavior, solubilizing ethyl and butyl side-chains are added to the phenyl groups and directly to the dicationic bisphenalenyl core, disrupting strong ground-state π-stacking. Through steady-state absorption and emission spectroscopy, time-resolved photoluminescence (trPL), transient absorption spectroscopy (TAS), density functional theory (DFT) and time-dependent DFT (TDDFT), and diffusion-ordered NMR spectroscopy (DOSY), excimer emission is shown to originate from dynamic, photoinduced dimerization rather than from preaggregated ground-state species. These findings establish a structure–function framework for modulating excited-state interactions via steric control, offering a tunable platform for designing emissive materials with environment-responsive behavior.

**Scheme 1:**
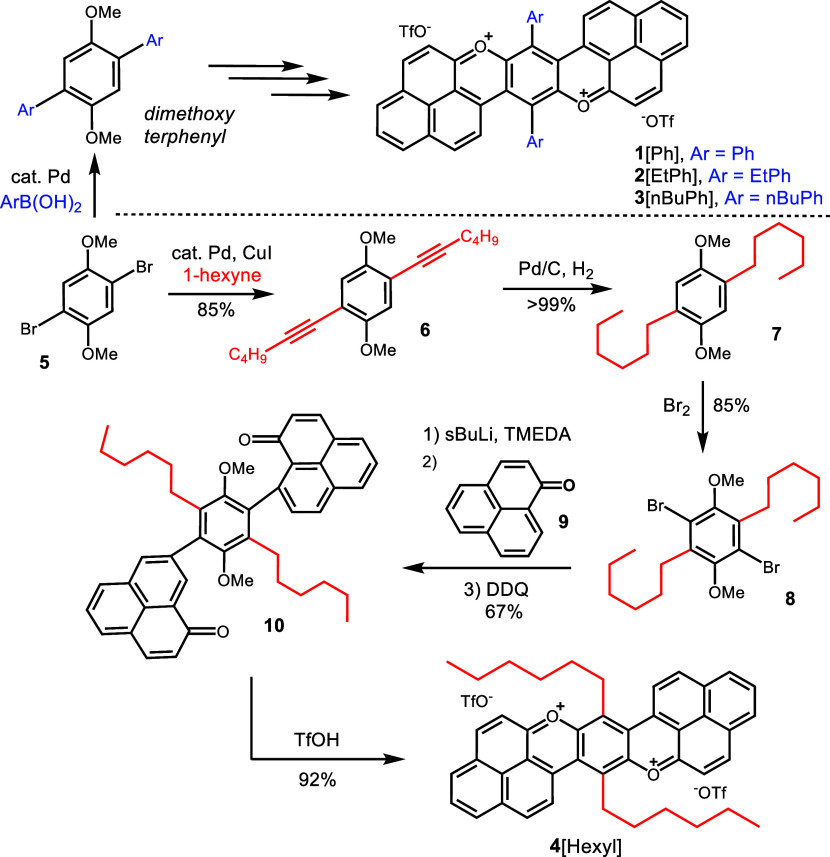
Synthesis Route of **1**[Ph], **2**[EtPh], **3**[nBuPh], and **4**[Hexyl]

## Methods

### General Synthetic Methods

All commercially available reagents obtained from the suppliers were used without further purification. Unless otherwise noted, all reactions were carried out under nitrogen with the standard Schlenk technique, and all glassware used in dry reactions was flame-dried under high vacuum prior to use. Tetrahydrofuran (THF), dimethylformamide (DMF), dichloromethane (DCM), and toluene were purified and dried by being passed through two columns of neutral alumina under nitrogen prior to use. Flash chromatography was performed using VWR High Purity 60Å (particle size 40–60 μm) silica gel. Data from high-resolution mass spectrometry (HRMS) using electrospray ionization (ESI) were obtained at the Notre Dame mass spectrometry facility. All synthetic procedures and characterization are in the Supporting Information.

### ^1^H, ^13^C, and DOSY NMR

All ^1^H and ^13^C NMR spectra were obtained with a Bruker AV-400 or a Bruker DRX-500 NEO with a CryoPlatform cryoprobe. Carbon spectra were measured with a proton-decoupling pulse program.

DOSY NMR experiments were carried out with a Bruker AV-400 with a CryoPlatform cryoprobe. The sample solutions were prepared at a concentration of ∼500 μM and transferred to an NMR tube. All DOSY experiments were performed by using a bipolar gradient pulse paired simulated echo and ledbpgp2s1d pulse sequence at a temperature of 298 K. The longitudinal eddy current delay and the gradient recovery delay were kept at a fixed value of 5 ms. The strength of the pulsed-field gradients was incremented from 2% to 98%, while the diffusion-sensitive period (50 ms) and the gradient duration (0.75 ms) were optimized to obtain a signal-to-noise ratio of >5%. An average of 16 scans was obtained and processed by using Dynamics Center software. The diffusion coefficients were obtained by integrating ^1^H NMR peaks between 0 and 10 ppm for each 2D DOSY NMR spectrum. The average diffusion coefficients were then calculated using the SEGWE software.^26^

### Absorption Spectroscopy

UV–vis–NIR spectral data were measured at room temperature (298 K) with a Varian Cary 5000 spectrophotometer. UV–vis spectral data were measured at room temperature (298 K) with an Ocean Insight diode array spectrometer. All samples were collected in a 2 × 2 mm cuvette. All solvents used for the solution samples were dried and degassed prior to use. Wavelengths are shown in nanometers (nm) and absorption in arbitrary units (a.u.).

### Steady-State and Time-Resolved Emission Spectroscopy

Fluorescence data were measured at room temperature (298 K) with an ISS Chronos BH spectrofluorometer. Steady-state spectra were collected with an excitation slit width of 1 mm and an emission slit width of 0.5 mm. Wavelengths are shown in nanometers (nm), and fluorescence is reported in arbitrary units (arb. units).

Lifetime measurements were determined using the time-correlated single photon counting (TCSPC) technique, in which a nanoLED laser (470 nm) was used as an excitation source. For **2**[EtPh], a 495–600 nm bandpass filter was used to collect the monomer emission lifetime, and a 700 nm long-pass filter was used to collect the excimer emission lifetime. For **3**[nBuPh], a 510 nm long-pass filter and a 550 nm bandpass filter (fwhm = 40 nm) were used to collect the monomer emission lifetime, and a 670 nm bandpass filter (fwhm = 10 nm) was used to collect the excimer emission lifetime. For **4**[Hexyl], a 510 nm long-pass filter was used to collect the emission lifetime. An IRF was collected in the absence of filters in order to deconvolute the emission lifetime data during fitting.

### Transient Absorption Spectroscopy

Samples of **2**[EtPh], **3**[nBuPh], and **4**[Hexyl] were prepared to an 80 μM concentration in acetonitrile and placed in a 2 mm quartz cuvette fitted with a stir bar. An Ultrafast Helios spectrometer was used to collect transient absorption spectroscopy (TAS) data on **2**[EtPh], **3**[nBuPh], and **4**[Hexyl]. A Coherent Libra amplified Ti:sapphire system at 1.1 W and 1 kHz repetition rate was used to generate 100 fs pulses of 800 nm laser light. 80% of the 800 nm pulse was split using a beam splitter and sent to a Topas-C optical parametric amplifier to generate a pump pulse of 590 nm. The pump pulse was further attenuated to ∼1.0 mW. The remaining 20% of the 800 nm pulse was sent through a CaF_2_ crystal to generate a white light continuum to be used as the probe pulse. The data were collected over the available 5.1 ns time window. Each data set comprises three scans that were averaged together with 250 points in each scan. The samples were stirred throughout the course of the TAS experiment, and the absorption spectra were collected before and after each experiment to confirm no degradation occurred during the TAS experiment.

The data were prepared in Surface Xplorer using a previously published method.^27^ A chirp correction was determined from a blank sample and applied to each data set, as shown in Figure S44, Figure S46, and Figure S48. A Python-based fitting program produced by the Young lab, which employs a parallel model of exponential decays, was used to perform global lifetime analysis (GLA) to determine the decay-associated difference spectra (DADS) and their associated lifetimes. Single-wavelength fitting of the bleach feature was also performed, and similar numbers and values of lifetimes were obtained (Figure S50–S52). Multiple fits were performed and compared with residual surfaces to determine the best fit for each data set.

### Time-Dependent Density Functional Theory

All calculations for single molecules and dimers were performed using the Gaussian09 ^28^ and Gaussian16 ^29^ programs, respectively. The relaxed ground state geometries of **1**, **2**, and **3** were optimized using the B3LYP^30–33^ hybrid functional with a double-ζ basis set, including polarization and dispersion, 6-31+G­(d,p),^34–38^ and a complete polarizable continuum model (PCM)^39^ solvent description of acetonitrile. In addition, dispersion was accounted for using the GD3 correction.^40^ Each ground state was run with the known charge and multiplicity of +2 singlet. Each singlet was checked for open-shell character through a stability check, and each geometry was confirmed as a true minimum with no imaginary frequencies.

## Results & Discussion

### Synthesis of Bisphenalenyl Moieties

Synthesis of the aryl-substituted bisphenalenyls (**2**[EtPh], **3**[nBuPh]) followed the reaction sequence of **1**[Ph], described in previous reports.^25^ Here, different *para*-substituents were obtained by using the corresponding commercially available aryl boronic acid (Scheme 1). To obtain the alkyl-substituted analogue (**4**[Hexyl]), Sonogashira cross-coupling^41,42^ of **5** was performed with 1-hexyne to furnish diyne **6**. Hydrogenation with Pd/C (**7**) followed by nuclear dibromination (**8**) then enabled the use of *s*BuLi-promoted addition of two equivalents of phenalenone (**9**) to provide bisphenalenone (**10**). Finally, cyclization with TfOH was performed to generate dipyrylium **4**[Hexyl]. As designed, each of the substituted analogues is noticeably more soluble than **1**[Ph], which is only partially soluble in 1,1,2,2-tetrachloroethane.

### Extremely Solubilizing Side Chains

A structurally modified bisphenalenyl derivative designed to reduce the potential for π–π stacking was synthesized. *n*-Hexyl chains were introduced directly onto the bisphenalenyl backbone, **4**[Hexyl], to mimic other solubilizing alkyl groups commonly employed in organic molecules to disrupt aggregation and enhance solubility in organic solvents.^43^ Alkyl chains display more conformational freedom, which prevents π–π stacking by keeping the π-backbones separated.^44^ The highest occupied molecular orbital (HOMO) and lowest unoccupied molecular orbital (LUMO) of **4**[Hexyl] are delocalized uniformly across the π-conjugated backbone (Figure 2), and the first vertical excitation at 562 nm matches the shoulder seen in the absorption spectra (Figure 3, left). UV–visible spectroscopy in acetonitrile (ACN) confirms that **4**[Hexyl] retains key absorption features characteristic of monomeric bisphenalenyl species (Figure S24) and remains unchanged at 10 μM, 40 μM, and 80 μM. The TDDFT-predicted transitions for **4**[Hexyl] align well with the experimental UV–vis spectrum (Figure 3, left).

**Figure 2:**
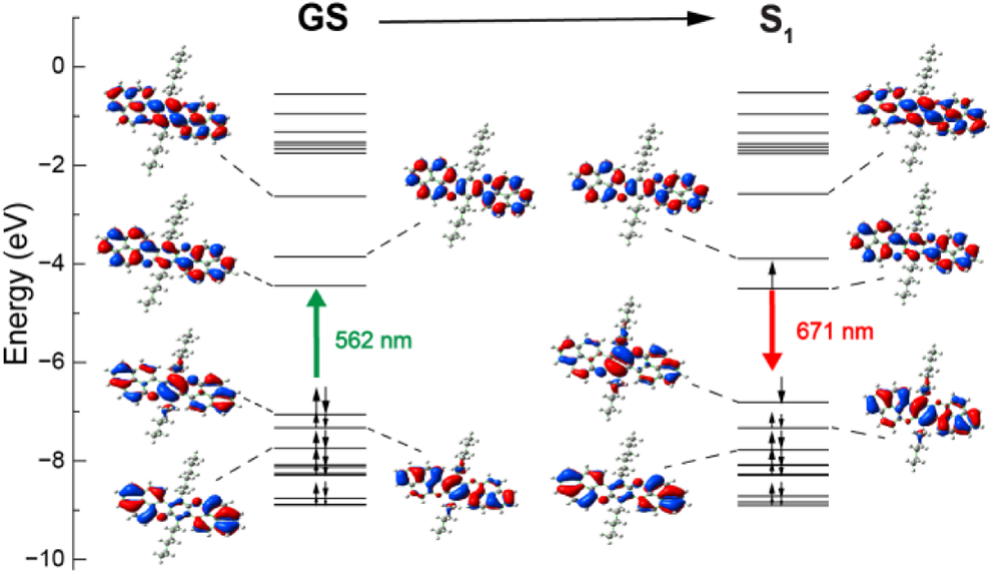
Molecular orbital diagram of the optimized GS and the first excited state singlet of **4**[Hexyl]. B3LYP-D3/6-31+G­(d,p)/PCM­(ACN).

**Figure 3:**
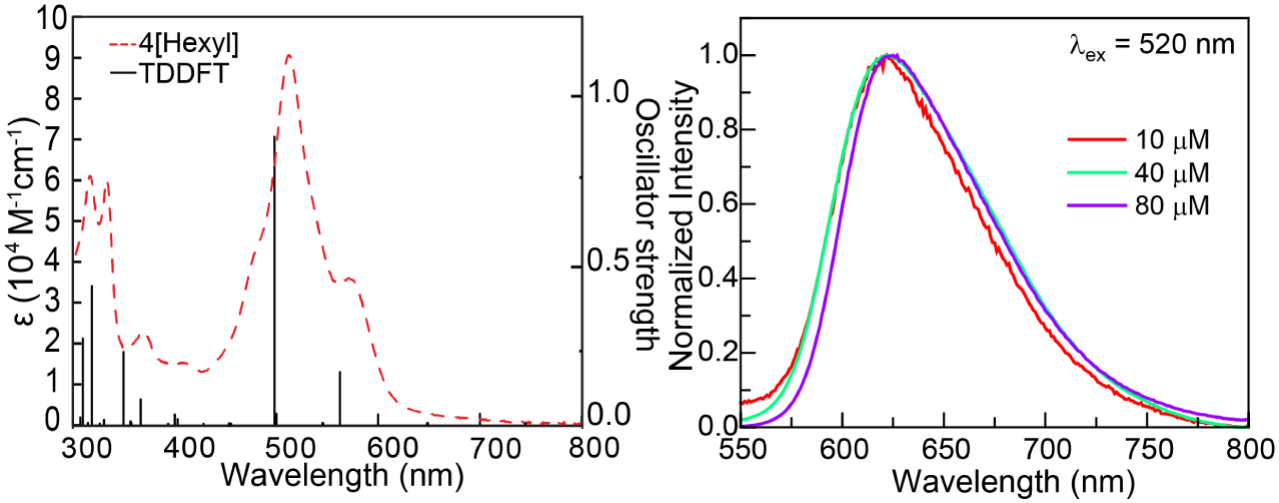
(left) TDDFT calculations of **4**[Hexyl] overlapped with the experimental results (B3LYP-D3/6-31+G­(d,p)/PCM­(ACN)). (right) Emission spectra at 10 μM, 40 μM, and 80 μM (λ_ex_ = 520 nm).

Emission spectra collected at 10 μM, 40 μM, and 80 μM showed a single emission band at λ_em_ = 620 nm, consistent with the parent monomer species (Figure 3, right), that aligns well with the predicted first excited state (S_1_) emission energy of 671 nm. Across all concentrations, **4**[Hexyl] shows an ∼3.4 ns monomer emission lifetime (Figure S32 and Table 1). This matches the longest TAS lifetime of ∼4 ns (Figure S45 and Table 1). TAS also reveals an initial decay of <1 ps followed by a 69.3 ps component (Figure S45 and Table 1). The alkyl side chains prevent close molecular packing and π-stacking that result in **4**[Hexyl] remaining as an isolated, nonaggregated moiety in both the ground- and excited-state configurations. Therefore, for the rest of the article, we focus on **2**[EtPh] and **3**[nBuPh], which show interesting excited-state emission behavior (*vide infra).*

**Table 1:** Summary of Photophysical Properties of **2**[EtPh], **3**[nBuPh], and **4**[Hexyl] in Acetonitrile

	Conc. (μM)	Abs λ_max_ (nm)	PL λ_mono_ (nm)	PL λ_excm_ (nm)	PL τ_mono_ (ns)	PL τ_excm_ (ns)	τ_1_ (ps)^c^	τ_2_ (ps)^c^	τ_3_ (ns)^c^	PL QY_mono_ (%)	PL QY_excm_ (%)
**2**[EtPh]	10	523	560	709	8.04 ± 0.24	1.11 ± 0.01					
	40	523	560	709	7.03 ± 0.96	1.10 ± 0.01				0.64 ± 0.36	0.05 ± 2.11
	80	523	560	709	6.73 ± 0.39	1.09 ± 0.01	<1	97.5 ± 20.9	1.19 ± 0.02		
**3**[nBuPh]	10	523	560	709	2.73 ± 0.04	1.32 ± 0.03					
	40	523	560	709	2.25 ± 0.01^a^	1.36 ± 0.01^b^				0.92 ± 0.06	0.81 ± 0.51
	80	523	560	709	2.81 ± 0.02	1.30 ± 0.01	<1	45.1 ± 26.2	1.15 ± 0.01		
**4**[Hexyl]	10	515	622		3.48 ± 0.06						
	40	515	621		3.48 ± 0.01					13.91 ± 0.00	
	80	515	627		3.41 ± 0.04		<1	69.3 ± 4.99	4.09 ± 0.10		

a 4% signal is 4.86 ns.

b 2% signal is 5.45 ns.

c TAS lifetime obtained from 590 nm excitation.

### Ground-State Solution Properties

To investigate the nature of electronic transitions and assess their aggregation behavior, ground-state absorption spectra were experimentally collected and predicted with TDDFT for **2**[EtPh] and **3**[nBuPh] (Figures S54, S55 and 4). Both **2**[EtPh] and **3**[nBuPh] have a slight twist across the central ring, as seen previously in **1**[Ph],^45^ leading to HOMOs (Figures S54, S55 and 4) that are centered on the central ring and the side phenyl rings. The LUMOs are delocalized across the whole bisphenalenyl backbone. The electron density localized on the phenyl groups in the ground state promotes a planar geometry.

**Figure 4:**
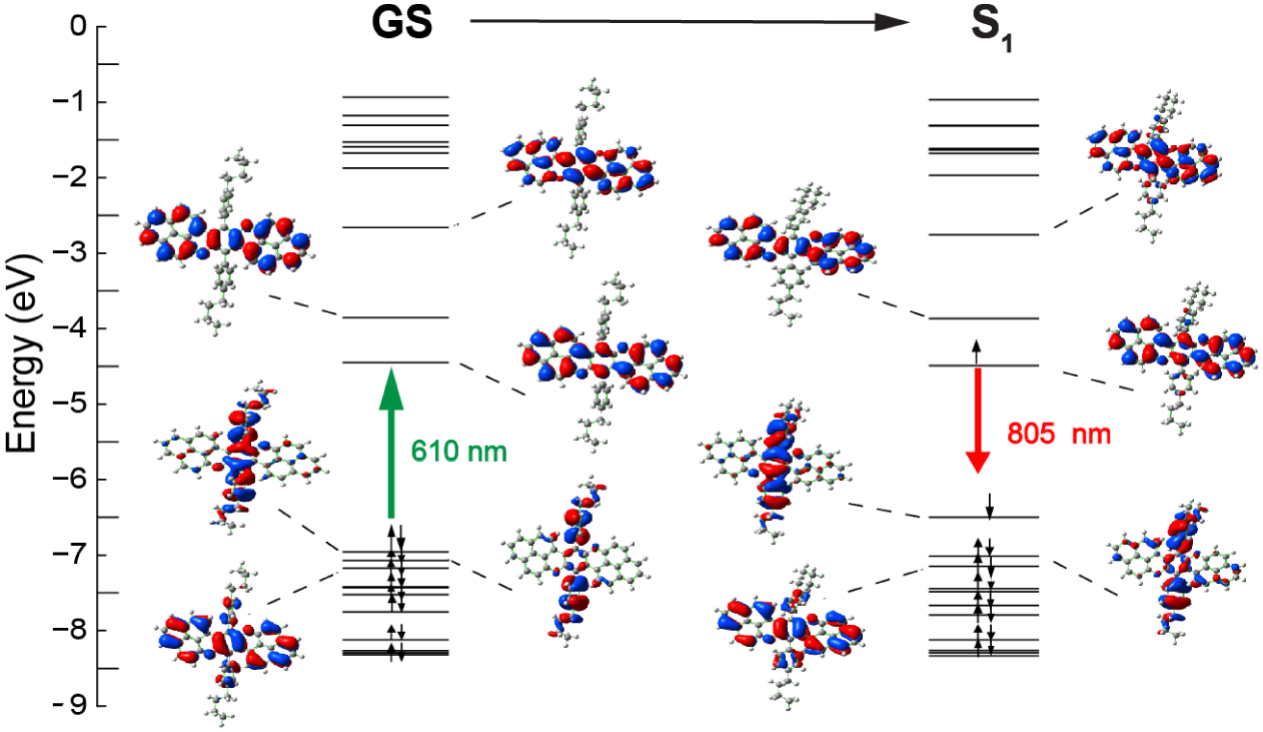
Molecular orbital diagram of the optimized GS and the first excited state singlet of **3**[nBuPh]. B3LYP/6-31+G­(d,p)/PCM­(ACN).

The visible region of the UV–vis absorption spectra is well represented by the predicted transitions of the singlet ground-state molecules (Figure 5, left), indicating no ground-state aggregation. The first excitation energies of the monomeric **2**[EtPh] (601 nm) and **3**[nBuPh] (610 nm) are both HOMO → LUMO transitions (Figures S53 and 5). Importantly, the absorption spectrum of both **2**[EtPh] and **3**[nBuPh] remained unchanged (Figures S2 and S5) when the solution concentration was varied between 10–120 μM for **2**[EtPh] and 10–80 μM for **3**[nBuPh].

**Figure 5:**
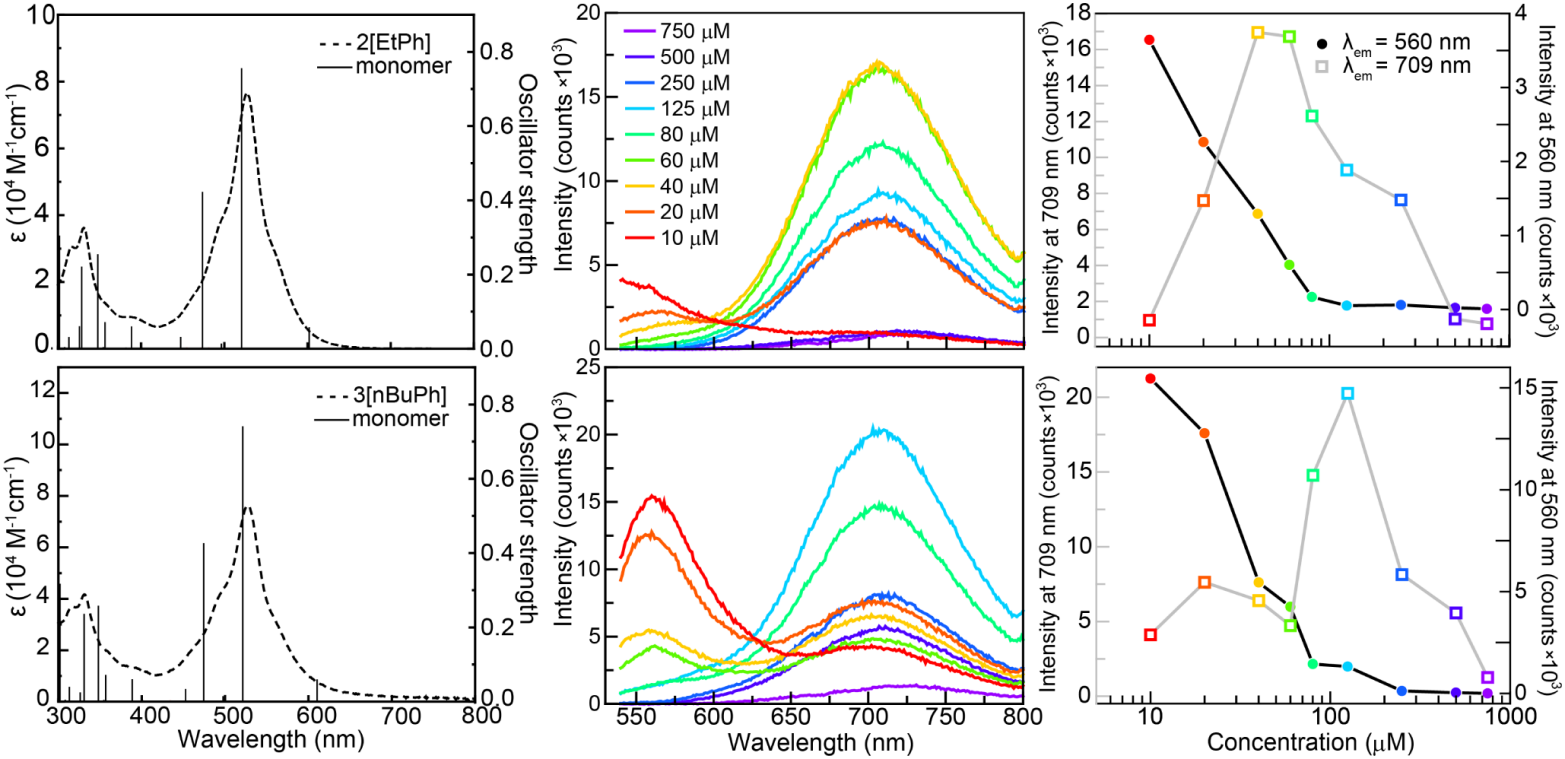
(left) UV–vis absorption spectra (dashed line) of **2**[EtPh] (top) and **3**[nBuPh] (bottom) in acetonitrile and transitions (solid line) for bisphenalenyl moiety monomers at the B3LYP-D3/6-31+G­(d,p)/PCM­(ACN) level of theory. (middle) Concentration-dependent emission spectra (λ_ex_ = 520 nm) of **2**[EtPh] (top) and **3**[nBuPh] (bottom) in acetonitrile. (right) Intensity at the observed emission wavelength for the monomer (λ_mono_ = 560 nm) and excimer (λ_excm_ = 709 nm) at various concentrations for **2**[EtPh] (top) and **3**[nBuPh] (bottom).

DOSY NMR was employed to confirm that **2**[EtPh] and **3**[nBuPh] exist as monomers in the GS. DOSY NMR provides experimentally determined diffusion coefficients for species in solution that can be directly correlated to their hydrodynamic size and therefore can be used in these studies to distinguish between monomers and aggregates of the bisphenalenyl moieties. DOSY NMR spectra (Figure S37, S38) were obtained at significantly higher concentrations (∼520 μM) than those used in the UV–vis experiments (typically ∼10–500 μM), ensuring that if any aggregation were to occur under spectroscopic conditions, it would be even more pronounced under the NMR conditions. Analysis of the DOSY spectra yielded diffusion coefficients of *D*_**2**[EtPh]_ = 9.07 × 10^–10^ m^2^s^–1^ and *D*_**3**[nBuPh]_ = 9.11 × 10^–10^ m^2^s^–1^. The molecular weights calculated from these diffusion coefficients were found to be MW_**2**[EtPh]_ = 938 g mol^–1^ and MW_**3**[nBuPh]_ = 977.82 g mol^–1^.^26,46^ The predicted molecular weight falls within ∼5% of the actual monomeric molecular weights, strongly suggesting that only monomer species are present in the ground state, even at the high concentrations required for NMR analysis. The calculated hydrodynamic radii of *r*_**2**[EtPh]_ = 8.39 Å and *r*_**3**[nBuPh]_ = 8.36 Å are consistent with their expected monomeric dimensions. For reference, the end-to-end length of a single bisphenalenyl unit is 7.53 Å (Figure S57). Taken together, the experimental and computational evidence suggests that **2**[EtPh] and **3**[nBuPh] exist in a monomeric ground state in solution, indicating that the bulky side groups effectively break up the aggregation observed in **1**[Ph].

### Photoluminescence

Both the absorption and emission spectra were collected at a wide range of concentrations. While the absorption remained unchanged (*vide supra),* the PL spectrum changed significantly as a function of concentration (Figure 5, middle column). Emission spectra of **2**[EtPh] and **3**[nBuPh] reveal two peaks. The PL intensities of the orange (λ_mono_ = 560 nm) and red (λ_excm_ = 709 nm) peaks change relative to each other as a function of concentration (Figure 5, middle column). At low concentrations (<20 μM), the orange emission (λ_mono_ = 560 nm) dominates, with minimal red PL (λ_excm_ = 709 nm). As the concentration increases, the red emission (λ_excm_ = 709 nm) becomes increasingly prominent relative to the orange emission peak. The dominance of the red PL at high concentrations is indicative of an excimer, allowing the orange PL to be assigned to the monomer (Figure 5).

This excimer must be dynamically formed as the ground state is purely monomeric (*vide supra*). Therefore, the increasingly prominent red PL indicates the formation of M*–M complexes through dynamic excited-state interactions. The increase in excimer intensity (λ_excm_ = 709 nm) up to ∼60 μM indicates that the population shifts from isolated monomers emitting (lower concentration) to excimer-forming dimers as molecular encounters (M + M*) become more frequent at higher concentrations. Interestingly, at concentrations above ∼60 μM, the excimer emission intensity of **2**[EtPh] begins to decrease (Figure 5, right column). It seems that at high concentration, additional aggregation pathways may become competitive for **2**[EtPh], possibly leading to static aggregates or nonemissive species that quench excimer fluorescence. Such behavior is also observed at higher concentrations above ∼125 μM in **3**[nBuPh]. The different high-concentration onset (>60 μM for **2**[EtPh] and >125 μM for **3**[nBuPh]) is likely due to the increased solubility and steric protection provided by the bulkier butylphenyl side chains.

### Excited-State Dynamics

The photophysical dynamics and comparison between monomeric and excimeric species across varying concentrations and solvent environments provide a full picture of the deactivation pathways in **2**[EtPh] and **3**[nBuPh]. The excited monomer has a lifetime of ∼7 and 2.5 ns for **2**[EtPh] and **3**[nBuPh], respectively. Both **2**[EtPh] and **3**[nBuPh] show some excimer formation even at 10 μM, with excimer lifetimes of about 1 ns for both.

TAS measurements were collected for **2**[EtPh] and **3**[nBuPh] in ACN at 80 μM, providing the dynamics of the excimer in each. The TAS excited-state lifetime of the monomeric species is not obtained because of the low signal in TAS measurements at concentrations low enough to observe monomer-only dynamics. Key excited-state lifetimes are shown in Table 1, while full TAS spectra, GLA fitting results, and detailed discussion are available in the Supporting Information (Figures S43–S52). Three components were identified in the kinetic fitting: an early subpicosecond decay (<1 ps), a second component with a lifetime of ∼97.5 ps for **2**[EtPh] and 45.1 ps for **3**[nBuPh], and a final component with lifetimes of ∼1.1 ns for **2**[EtPh] and for **3**[nBuPh]. Single-wavelength fitting of the bleach component produced a similar fitting model in terms of both the number of lifetimes and their values.

Complementary trPL measurements were conducted (Figure S29–S35 and Table 1). To identify the excited-state lifetimes of monomeric and excimer species, trPL measurements were performed at three concentrations (10 μM, 40 μM, 80 μM) for **2**[EtPh] and **3**[nBuPh] in ACN. Emission decay profiles were selectively monitored using a 495–600 nm bandpass filter and a 700 nm long-pass filter to isolate monomeric and excimer emission, respectively. For **2**[EtPh], the monomeric emission lifetime (τ_mono_) slightly increases with a decrease in concentration from ∼7 ns at both 80 and 40 μM to ∼8 ns at 10 μM. The excimer emission lifetime for **2**[EtPh] is ∼1.1 ns. For **3**[nBuPh], the monomer lifetime is ∼2.5 ns, and the excimer lifetime is ∼1.3 ns for each concentration.

In the case of **2**[EtPh] and **3**[nBuPh], TAS and trPL support the presence of a monomeric excited state (M*) that, under appropriate conditions, evolves into a dynamic excimer (M–M*, Figure 6). Upon excitation in the singlet manifold, each molecule undergoes rapid internal conversions and vibrational cooling (<1 ps), resulting in a thermally relaxed singlet excited state (M* or S_1_).

**Figure 6:**
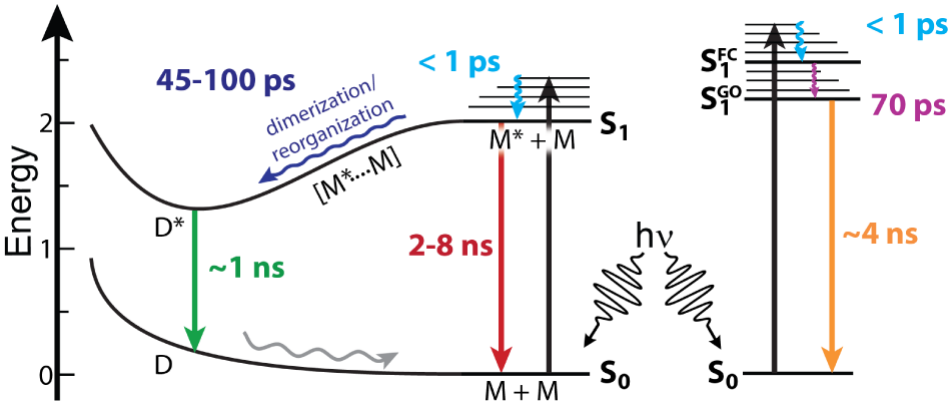
(left) Proposed photophysical mechanism of (left) **2**[EtPh] and **3**[nBuPh] in ACN and (right) **4**[Hexyl].

In **2**[EtPh], the excited-state molecule can relax via two routes. In the absence of significant intermolecular interaction, such as at low concentrations, radiative decay proceeds from the monomeric excited state with an observed emission lifetime of ∼7 ns. Molecular encounters that result in an M–M* excimer show a lifetime in TAS measurements on the time scale of ∼98 ps. The absorption of this dimer species is computed to be indistinguishable (Figure S62–S63) from the monomer; thus, formation of the excimer does not result in a typical spectral shift associated with J- or H-type aggregation. This lifetime reflects the dimerization process as well as the structural relaxation from the S_1_ Franck–Condon geometry. This dynamic excimer emits at λ_excm_ = 709 nm and decays with a characteristic ∼1.1 ns lifetime, as observed with both TAS and trPL. The reversible, concentration-dependent behavior observed for **2**[EtPh] suggests that moderate steric bulk allows sufficient conformational flexibility to enable productive M–M* encounters while still limiting excessive aggregation.

In **3**[nBuPh], the molecule can also undergo one of the two competing pathways. In the absence of significant intermolecular interaction, the excited molecule returns to the ground state via monomeric emission with a lifetime of ∼2 ns. While substitution of ethylphenyl for butylphenyl introduces slightly greater solubility, it also enables a larger twist across the center ring, stabilizing the singlet excited state in **3**[nBuPh]. This enhanced solvation and favorable excited-state π–π* orbital overlap seem to speed up the M–M* formation and structural relaxation from the S_1_ Franck–Condon geometry, within a ∼35 ps time scale. The dynamic excimer in **3**[nBuPh] decays with a similar lifetime of ∼1.1 ns as observed in **2**[EtPh]. The data suggest that solubility and side-chain extension work in tandem to fine-tune the excited-state organization, allowing excimer formation to occur more efficiently without triggering ground-state aggregation.

In contrast, **4**[Hexyl], which incorporates long, flexible alkyl chains, follows a distinct monomeric pathway. No excimer formation is observed experimentally, as outlined above, meaning that the formation of an excimer is not involved in the photophysical mechanism described below. The photodriven dynamics begin with excitation, followed by rapid internal conversions to a vibrationally hot S_1_ (M*) state (<1 ps). TAS reveals an ∼69.3 ps component attributed to solvent reorganization around the excited-state dipole, followed by relaxed monomer emission with a lifetime of ∼4 ns. It is notable that the fitted DADS for the ∼10s ps lifetime (τ_2_) for **2**[EtPh] and **3**[nBuPh] (Figure S43) are similar to each other but are different from **4**[Hexyl], which indicates that τ_2_ represents a somewhat different process in the **4**[Hexyl] mechanism. The longer monomer emission lifetime, compared to those of **2**[EtPh] and **3**[nBuPh], is supported by the lack of significant geometric change between the ground and excited states (Figure S60) in **4**[Hexyl]. The optimized **4**[Hexyl] excited state is still relatively high in energy due to the small geometry change from the initial Franck–Condon state. In contrast, the redistribution of electron density onto the π-backbone induces torsional twisting of both **2**[EtPh] and **3**[nBuPh] upon excitation. The calculated dihedral angles between the phenyl ring and the π-core increase upon excitation, with θ_**2**[EtPh]_ = 8° and θ_**3**_[nBuPh] = 12° (Figure S58, S59). This twisting distorts the π-conjugated backbone and destabilizes the ground state by ΔGS_**2**[EtPh]_ = 0.97 eV and ΔGS_**3**[nBuPh]_ = 0.28 eV, resulting in shorter monomer lifetimes, in accordance with the energy gap law. These observations highlight how side-chain substitution tunes the balance between monomeric and excimeric behavior.

### Excited-State Mechanism Summary

The photophysical mechanisms assigned to **2**[EtPh], **3**[nBuPh], and **4**[Hexyl] were developed using the steady-state and time-resolved data (*vide supra*) and are shown in Figure 6. Experimental data including emission (*vide supra*) and NMR data (*vide supra*) indicate that dynamic excimers form for **2**[EtPh] and **3**[nBuPh], but not for **4**[Hexyl], which is reflected in the proposed excited-state mechanism. The TAS data revealed three lifetimes extracted from GLA for each moiety. The emission lifetime data collected for both the monomers (**4**[Hexyl]) and excimers (**2**[EtPh], **3**[nBuPh]) align well with the nanosecond (τ_3_) lifetime from TAS data, enabling that TAS lifetime to be assigned as the final deactivation to the ground state. The excimer should therefore be formed prior to that time scale. The τ_1_ < 1 ps lifetime is too short for excimer formation or geometry reorganization processes and is more typical of vibrational cooling or IC to the S_1_ state and was therefore assigned as such. Because τ_1_ and τ_3_ can be assigned to thermal relaxation and emission, respectively, τ_2_ must represent excimer formation and geometry reorganization. Because of the evidence of excimers and the similar τ_2_ DADS values for **2**[EtPh] and **3**[nBuPh] (Figure S43), τ_2_ is assigned to geometry relaxation and excimer formation for **2**[EtPh] and **3**[nBuPh]. The lack of excimer emission and different DADS indicate that τ_2_ represents a different process in **4**[Hexyl] than the other moieties. Therefore, the similar lifetime (τ_2_) is assigned to only geometry relaxation for **4**[Hexyl], as captured in the mechanism described in Figure 6.

### Control of Dynamic Excimer Formation

It is clear that dynamic excimer formation depends on nuanced intermolecular interactions between GS and excited-GS monomers, as well as the monomer–solvent intermolecular interactions. Thus, excimer formation was explored in viscous solvents and acidic environments.

### Solvation

Tetrachloroethane (TCE) was used to probe the effect of solvent viscosity on dynamic excimer formation. TCE is approximately 4× more viscous than ACN, and the increased viscosity is expected to reduce the diffusion rate of molecules, thereby decreasing the frequency of encounters between M and M*.^47^ Interestingly, **2**[EtPh] showed more excimer emission at low concentrations compared to ACN, and evidence of a red-shifted ground-state aggregate in solution emerged for **3**[nBuPh] in TCE.

Concentration-dependent UV–vis and PL studies of **2**[EtPh] in TCE revealed behavior consistent with excimer formation (Figure 7) at concentrations even lower than what was observed in ACN. At low concentrations (10 μM), the excimer emission peak at λ_excm_ = 709 nm dominated the emission spectrum, with only a minor shoulder peak from the monomer at λ_mono_ = 560 nm, suggesting excimer formation occurs more readily in TCE than in ACN. UV–vis spectra across the 10–200 μM range mirrored those in ACN, showing no substantial shift in absorption features, although solubility challenges limited measurements above 200 μM. Notably, emissive excimers appear more favorable in TCE than in ACN, as evidenced by longer emission lifetimes. For **2**[EtPh] and **3**[nBuPh], the excimer emission lifetimes increase from 1.1 to 1.3 ns in ACN to 3.5 ns in TCE (Figures S33-35, and Table S2). This is likely due to the increased viscosity of TCE subduing nonradiative processes, allowing for longer-lived M–M* dimers that result in more emission, although not necessarily more M–M* pairs.

**Figure 7:**
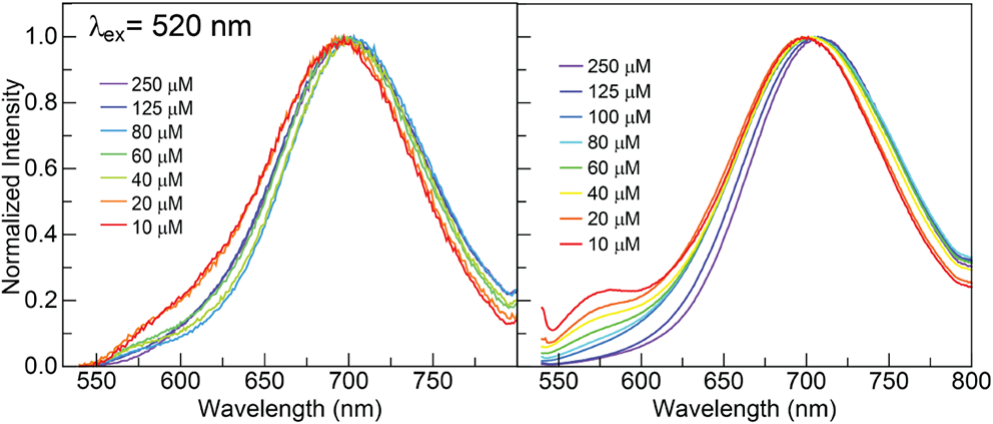
Concentration-dependent emission spectra (λ_ex_ = 520 nm) of (left) **2**[EtPh] and (right) **3**[nBuPh] in TCE. Normalization of the PL spectra of **2**[EtPh] and **3**[nBuPh] performed at the emission maximum.

Similarly to **2**[EtPh], **3**[nBuPh] showed excimer formation (Figure 7) at concentrations even lower than what was observed in ACN. The absorption spectra of **3**[nBuPh] are consistent over concentrations ranging from 10 to 125 μM (Figure S16). Emission indicative of the excimer (λ_excm_ = 709) is more prominent at lower concentrations in TCE (10–20 μM) compared to ACN.

To identify if ground-state aggregates form in TCE, DOSY NMR was employed. The diffusion coefficient of **3**[nBuPh] was *D* = 1.64 × 10^–1 0^ m^2^ s^–1^, corresponding to a calculated molecular weight of 1906.45 g mol^–1^, which is approximately twice the monomeric value and within ∼6% error of the expected dimer. The hydrodynamic radius *r* = 10.64 Å is significantly larger than that observed in ACN. Similarly, at ∼500 μM, **2**[EtPh] in TCE showed *D* = 1.58 × 10^–1 0^ m^2^ s^–1^ with a molecular weight of 2073.70 g mol^–1^, further supporting the formation of dimeric or aggregated species at the higher concentrations used for NMR. The corresponding hydrodynamic radius of *r* = 10.94 Å indicates an increase in effective molecular size and supports a more spherical geometry, consistent with dimer formation. These results indicate that both **2**[EtPh] and **3**[nBuPh] form ground-state dimers *in TCE at extremely high concentrations*, in contrast to ACN in which it does not form such ground-state dimers even at NMR concentrations. This behavior is likely facilitated by the lower polarity or higher viscosity of TCE, which reduces solvation of the charged bisphenalenyl cores and enables closer intermolecular association. Additionally, the formation of a shared solvation shell around a dimer may be energetically favored in this solvent environment, stabilizing the aggregated state relative to the fully solvated monomer.

The results underscore the pronounced influence of the solvent environment, particularly viscosity and solvation dynamics, on the aggregation and excimer-forming behavior of bisphenalenyl dication species. The increased molecular dimensions and lengthened emission lifetimes of excimer species in TCE suggest that solvent tuning can be an effective strategy for modulating excited-state interactions.

### Addition of Acid

Previous reports suggested that increasing the polarity of the chemical environment can enhance fluorescence through the formation of aggregates in solution, including via the formation of excimers.^10^ Acid additives are known to influence supramolecular interactions and can modulate aggregation or excimer formation through changes in polarity, hydrogen bonding, or ion pairing. To investigate the effect of the chemical environment on excimer behavior in **3**[nBuPh], acetic acid and trifluoroacetic acid (TFA) were initially selected.

Addition of acetic acid (p*K*_a_ (ACN) = 23.5)^48^ at a 1:0.001 molar ratio to **3**[nBuPh] in solution induced a distinct blue shift in the absorption maximum (λ_max_), consistent with ring-opening of the phenalenone core and indicative of compound degradation. In contrast, titration with TFA (p*K*_a_ (ACN) = 12.65)^49,50^ at up to a 1:1 molar ratio caused no observable changes in the absorption spectrum (Figure S20), suggesting structural integrity of the chromophore was retained.

Emission measurements in the presence of TFA revealed a pronounced enhancement of excimer emission (Figure S23) up to a 1:1 molar ratio of **3**[nBuPh]:TFA. Remarkably, when a stronger acid, tetrafluoroboric acid (HBF_4_, p*K*_a_ (ACN) = 1.80), was added to **3**[nBuPh] in ACN, complete red-shifting of the emission to λ_excm_ = 709 nm was observed at an extremely low molar ratio of 1:0.001 **3**[nBuPh]:HBF_4_ (Figure 8). This pronounced shift suggests that the higher acidity of HBF_4_, along with its smaller BF_4_^–^ counterion, more effectively facilitates ion pairing or coordination with the dicationic core, thereby promoting excimer formation even more efficiently than the bulkier TFA anion. The observed differences in excimer enhancement among the acids likely reflect their relative acid strengths and dissociation behaviors: stronger acids with lower p*K*_a_ values generate a higher concentration of available counterions capable of stabilizing the excimer-forming species.

**Figure 8:**
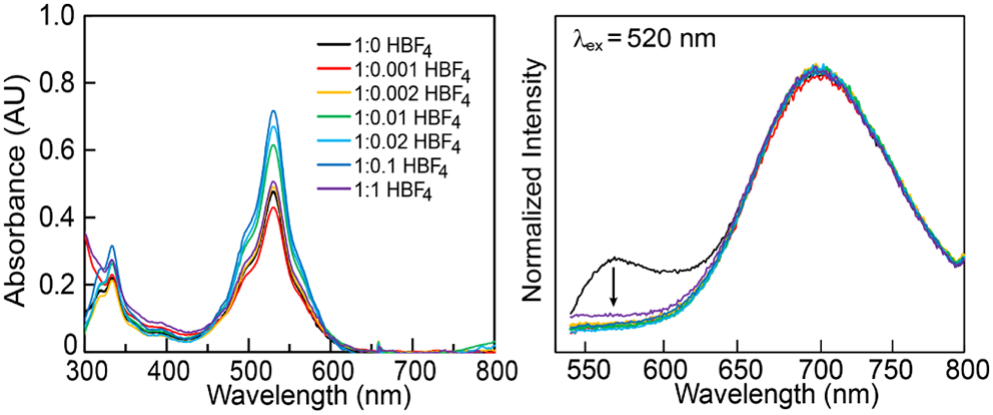
(left) UV–vis absorption spectra and (right) PL spectra of **3**[nBuPh] upon increasing the concentration of HBF_4_ (λ_ex_ = 520 nm, 0.1–100 mol %, c_**3**[nBuPh]_ = 40 μM).

DOSY NMR was used to provide insight into ion pairing and solvent–solute interactions between **3**[nBuPh] and the trifluoroacetate or BF_4_^–^ anions (Figure S41, S42). The diffusion coefficient of **3**[nBuPh] in the presence of TFA was measured to be *D*_**3**[nBuPh]_ = 10.50 × 10^–10^ m^2^ s^–1^ with a MW_**3**[nBuPh]_ = 701.24 g mol^–1^. The molecular weight is much lower than that of the monomer species (1011.5 g mol^–1^), indicating that the TFA anions are not interacting with the cationic core. The diffusion coefficient of **3**[nBuPh] in the presence of HBF_4_ was measured to be *D*_**3**[nBuPh]_ = 8.14 × 10^–10^ m^2^ s^–1^, corresponding to a calculated MW_**3**[nBuPh]_ = 1278.96 g mol^–1^. The molecular weight is higher than that of the monomer alone (1011.5 g mol^–1^), but lower than that of the dimer (2023 g mol^–1^). The additional weight corresponds to approximately 2 BF_4_^–^ anions in solution, suggesting that the ions are associated with cationic **3**[nBuPh]. The corresponding hydrodynamic radius of *r*_**3**[nBuPh]_ = 9.31 Å also reflects a slight increase in molecular size and spherical character, consistent with enhanced ion pairing or solvation effects involving BF_4_^–^ and the dicationic core of **3**[nBuPh].

The introduction of HBF_4_ proved to be a highly effective strategy for promoting excimer formation in **3**[nBuPh], even at extremely low molar ratios. The strong acid induced a complete red-shift in emission at λ_excm_ = 709 nm and enabled stronger ionic interactions between the dication and BF_4_^–^ anions, as supported by DOSY NMR analysis. The observed increase in molecular weight and hydrodynamic radius suggests the formation of stable ion pairs or solvent-shielded aggregates in solution. Compared to bulkier acids like TFA, the smaller BF_4_^–^ anion of HBF_4_ facilitates closer interaction with the charged core, offering a powerful tool for controlling the photophysical behavior through acid-anion coordination. These results highlight the sensitivity of excimer emission to subtle changes in the ionic environment and provide a tunable approach for modulating excited-state properties.

## Conclusion

Through targeted molecular design and detailed photophysical analysis, a tunable platform for controlling dynamic excimer formation in solution-phase bisphenalenyl systems is established. Modifying the dicationic core and introducing bulky or solubilizing side chains allow for solution aggregation in the ground or excited state depending on the solvent environment. Structure–property relationships between viscosity and ionic interactions enable control over the monomeric and excimeric photoluminescence pathways.

Among the derivatives studied, **4**[Hexyl] showed no evidence of excimer formation, supporting the role of side-chain solubilization and geometry retention in maintaining monomeric excited states. In contrast, **2**[EtPh] and **3**[nBuPh] displayed concentration-dependent emission consistent with dynamic excimer formation in ACN. Steady-state spectroscopy, TDDFT calculations, and DOSY NMR confirmed that these species remain monomeric in the ground state in ACN and interact only upon photoexcitation. Excimer emission was observed over a broad concentration range, extending to ∼125 μM, offering a wide experimental window for studying dynamic excited-state interactions. TAS and trPL clarified the kinetics of excited-state evolution, identifying distinct lifetimes for monomer and excimer decay processes.

Solvent and chemical environments were also found to influence excimer behavior. TCE increased aggregation tendencies, while the addition of HBF_4_ promoted excimer emission in **3**[nBuPh] even at substoichiometric levels, highlighting the tunability of excimer pathways through acid–anion interactions.

Together, these findings offer a framework for designing solution-phase molecular systems with controllable excimer behavior. This work provides foundational insight into structure–function relationships in π-conjugated materials and may inform future development of responsive emitters for use in optoelectronic, sensing, or dynamic fluorescence applications.

## Supplementary Material



## Notes

Synthetic procedures; ^1^H, ^13^C, and DOSY NMR; additional steady-state spectroscopy data; concentration-dependent spectroscopy data in TCE; spectroscopy data with the addition of acid; fit analysis of time-resolved photoluminescence and transient absorption spectroscopy data; TDDFT molecular orbital diagrams; TDDFT ground-state and excited-state geometry optimizations; and a TDDFT table of singlet transitions (PDF)

## Abbreviations

GS: ground state
S_0_: singlet ground state
S_1_: first singlet state
PL: photoluminescence
DOSY NMR: diffusion order spectroscopy, nuclear magnetic resonance
M: monomer
M*: excited monomer
D: dimer
D*: excited dimer
