# Supplementary material for: Tunable Dynamic Excimer Formation in Bisphenalenyl Derivatives through Molecular Packing
Source: J Phys Chem A. 2026 Mar 10;130(12):2488–98. doi: 10.1021/acs.jpca.5c07790 (PMC13034411; doi:10.1021/acs.jpca.5c07790)
Supplement: Supplementary file 1 [file jp5c07790_si_001.pdf]

Supporting Information for

Tunable Dynamic Excimer Formation in Bisphenalenyl Derivatives through Molecular Packing

Gisselle Y. Rojas, Domenica R. Fertal, Isabelle A. Herlinger, Mark S. Chen, Lisa A. Fredin*, Elizabeth R. Young*

Department of Chemistry, Lehigh University, Bethlehem, Pennsylvania 18015, USA.

Contents

General synthetic methods 7

Synthetic procedures and characterization 7

Scheme S1: Synthetic sequence to obtain ethyl-substituted bisphenalenyl 2[EtPh]. 7

Scheme S2: Synthetic sequence to obtain nbutyl-substituted bisphenalenyl 3[nBuPh]. 8

Scheme S3: Synthetic sequence to obtain nhexyl-substituted bisphenalenyl 4[Hexyl]. 10

^{1}H-NMR of 4 13

^{13}C-NMR of 4 14

^{1}H-NMR of 2[EtPh] 15

^{13}C NMR of 2[EtPh] 16

^{1}H-NMR of 5 17

^{13}C NMR of 5 18

^{1}H-NMR of 6 19

^{13}C-NMR of 6 20

^{1}H-NMR of 7 21

^{13}C-NMR of 7 22

1H-NMR of 3[nBuPh] 23

^{13}C-NMR of 3[nBuPh] 24

^{1}H-NMR of 9 25

^{13}C-NMR of 9 26

^{1}H-NMR of 10 27

^{13}C-NMR of 10 28

^{1}H-NMR of 11 29

^{13}C-NMR of 11 30

^{1}H-NMR of 12 31

^{13}C-NMR of 12 32
^{1}H-NMR of 4[Hexyl] 33
^{13}C-NMR of 4[Hexyl] 34
Steady State Spectroscopy 35
Figure S1: Full UV-vis Near-IR spectra of 2[EtPh] (solid green line), 3[nBuPh] (solid blue line) from 300nm-2000nm range. 35
Figure S2: (left) Concentration dependent absorption spectra of 2[EtPh] and (right) normalized absorbance at λ_{max} = 520 nm (c = 10-120 μM). 35
Figure S3: (left) Concentration dependent PL spectra of 2[EtPh] and (right) normalized PL spectra in acetonitrile. 36
Figure S4: Intensity at observed emission wavelength for monomer (λ_{em} = 560 nm) and excimer (λ_{em} = 709 nm) at various concentrations (10-750 μM) for 2[EtPh]. 36
Figure S5: (left) Concentration dependent absorption spectra of 3[nBuPh] and (right) normalized absorbance at λ_{max} = 520 nm (c = 10-80 μM). 37
Figure S6: (left) Concentration dependent PL spectra of 3[nBuPh] and (right) normalized PL spectra in acetonitrile. 37
Figure S7: Intensity at observed emission wavelength for monomer (λ_{em} = 560 nm) and excimer (λ_{em} = 709 nm) at various concentrations (10-750 μM) for 3[nBuPh]. 38
Figure S8: PL spectra of 3[nBuPh] at various λ_{ex} (420 nm – 600 nm, increments of 20 nm, c = 80 μM) and absorption spectra (black dash line) in acetonitrile. 38
Figure S9: Emission excitation spectra of 3[nBuPh] at different λ_{em} (620 nm – 700 nm, increments of 20 nm, c = 80 μM) and absorption spectra (black dash line) in acetonitrile. 39
Figure S10: PL spectra of 3[nBuPh] at various λ_{ex} (420 nm – 600 nm, increments of 20 nm, c = 20 μM) and absorption spectra (black dash line) in acetonitrile. 39
Figure S11: Emission excitation spectra of 3[nBuPh] at different λ_{em} (620 nm – 700 nm, increments of 20 nm, c = 20 μM) and absorption spectra (black dash line) in acetonitrile. 40
Table S1: Summary of dielectric constants and viscosity values for solvents 40
Figure S12: Solvent effect of 3[nBuPh]: absorption spectra of 3[nBuPh] in TCE (blue line, λ_{max} = 541 nm), DCM (purple line, λ_{max} = ~ 535 nm), ACN (black dashed line, λ_{max} = 523 nm), and DMSO (red line, λ_{max} = ~500 nm) 41
Figure S13: ^{1}H NMR of 3[nBuPh] in DMSO: ring opening of the π-backbone occurs in the presence of DMSO. 41
Environmental changes using viscous solvent and acid additives 42
Figure S14: Concentration dependent absorption spectra of 2[EtPh] in TCE (c = 20-125 μM). 42
Figure S15: Concentration dependent PL spectra of 2[EtPh] (λ_{ex} = 520 nm, c = 10-200 μM) in TCE solvent. 42
Figure S16: Concentration dependent absorption spectra of 3[nBuPh] in TCE (c = 10-125 μM). 43
Figure S17: Concentration dependent PL spectra of 3[nBuPh] (λ_{ex} = 520 nm, c = 10-250 μM) in TCE solvent. 43
Acid conditions 44

Figure S18: UV-vis absorption spectra of 3[nBuPh] in acetonitrile and upon addition of TFA at various molar ratio (3[nBuPh]:Acid, c = 40 μM)........ 44

Figure S19: UV-vis absorption spectra of 3[nBuPh] in acetonitrile upon addition of HBF_4 at various molar ratio (3[nBuPh]:Acid, c = 40 μM). 44

Figure S20: Excitation scan of 3[nBuPh] in acetonitrile and substoichiometric molar ratio of TFA overlapped with respective UV-vis spectra upon addition of TFA (λ_{obs} = 640nm, 0.1-100 mol%, c = 40 μM)........ 45

Figure S21: Excitation scan of 3[nBuPh] in acetonitrile and substoichiometric molar ratio of TFA overlapped with respective UV-vis spectra upon addition of HBF_4 (λ_{obs} = 700 nm, 0.1-100 mol%, c = 40 μM)........ 45

Figure S22: Emission spectra of 3[nBuPh] in acetonitrile (λ_{ex} = 520nm) at increasing concentration upon addition of TFA (0.1-100 mol%, c = 40 μM)........ 46

Figure S23: Emission spectra of 3[nBuPh] in acetonitrile (λ_{ex} = 520nm) upon addition of HBF_4 (0.1-100 mol%, c = 40 μM). 46

Photophysical properties of 4[Hexyl]: 47

Figure S24: Normalized UV-vis absorption spectra of 4[Hexyl] in acetonitrile (red line = 10 μM, green = 40 μM, purple = 80 μM)........ 47

Figure S25: Absorption spectra (red dash line, c = 10 μM) and excitation spectra of 4[Hexyl] in acetonitrile at different λ_{em} (570 nm – 620 nm, increments of 10 nm). 47

Figure S26: PL spectrum of 4[Hexyl] in acetonitrile: Monitoring emission behavior at various λ_{ex} wavelengths (500 nm – 600 nm, increments of 20 nm, c = 10 μM)........ 48

Figure S27: Absorption spectra (red dash line, c= 80 μM) and excitation spectra of 4[Hexyl] in acetonitrile at different λ_{em} (570 nm – 620 nm, increments of 10 nm). 48

Figure S28: (Left) PL spectra of 4[Hexyl] in acetonitrile: Monitoring emission behavior at various λ_{ex} wavelengths (500 nm – 600 nm, increments of 20 nm, c = 80 μM). (Right) PL spectrum of 80 μM 4[Hexyl] in TCE at λ_{ex} = 520 nm........ 49

Time-resolved Photoluminescence (trPL) 50

Figure S29: trPL of 2[EtPh] in acetonitrile collected at various concentrations (top to bottom: 10 μM, 40 μM, 80 μM) using a 495 – 600 nm bandpass filter to selectively collect monomer emission, shown with a fit to a monoexponential function. One trial shown to represent the three trials collected. 51

Figure S30: trPL of 2[EtPh] in acetonitrile collected at various concentrations (top to bottom: 10 μM, 40 μM, 80 μM) using a longpass filter of 700 nm to selectively collect excimer emission, shown with a fit to a monoexponential function. One trial shown to represent the three trials collected. 52

Figure S31: trPL of 3[nBuPh] in acetonitrile collected at various concentrations (10 μM, 40 μM, 80 μM) using a (top row) bandpass filter of 550 nm to selectively collect monomer emission and (bottom row) a bandpass filter of 670 nm to selectively collect excimer emission. 53

Figure S32: trPL of 4[Hexyl] in acetonitrile collected at various concentrations (10 μM, 40 μM, 80 μM)........ 53

Figure S33: trPL of 80 μM 2[EtPh] in TCE using a (top row) bandpass filter of 550 nm to selectively collect monomer emission and (bottom row) a bandpass filter of 670 nm to selectively collect excimer emission. 54

Figure S34: trPL of 80 μM 3[nBuPh] in TCE using a (top row) bandpass filter of 550 nm to selectively collect monomer emission and (bottom row) a bandpass filter of 670 nm to selectively collect excimer emission. 55

Figure S35: trPL of 80 μM 4[Hexyl] in TCE using a 510 nm bandpass filter. 56

Table S2: Comparison of photophysical properties of 80 μM 2[EtPh], 3[nBuPh], and 4[Hexyl] in ACN vs. TCE 56

Figure S36: Proposed mechanism (left) of the monomeric species of 2[EtPh] and 3[nBuPh] and (right) 4[Hexyl]. 57

DOSY NMR 58

Figure S37: DOSY NMR of 2[EtPh] in acetonitrile only with TMS as an internal standard. 58

Figure S38: DOSY NMR of 3[nBuPh] in acetonitrile only with TMS as an internal standard. 59

Figure S39: DOSY NMR of 2[EtPh] in TCE only with TMS as an internal standard. 60

Figure S40: DOSY NMR of 3[nBuPh] in TCE only with TMS as an internal standard. 61

Figure S41: DOSY NMR of 3[nBuPh] in ACN and TFA (1:0.1 molar ratio) with TMS as an internal standard. 62

Figure S42: DOSY NMR of 3[nBuPh] in ACN and HBF_4 (1:0.1 molar ratio) with TMS as an internal standard. 63

Transient Absorption Spectroscopy 64

Transient Absorption Spectroscopy Experimental Methods 64

Overview of Transient Absorption Data 64

Figure S43. (Top) Representative spectra of each molecule in acetonitrile at 590 nm ex. (Bottom) DADS determined from GLA fitting with lifetimes. 66

Figure S44. TAS heat map of 80 μM solution of 4[Hexyl] in acetonitrile before (left) and after (right) chirp correction. 67

Figure S45. TAS data and fits of 4[Hexyl] in acetonitrile. 68

Figure S46. TAS heat map of 80 μM solution of 3[nBuPh] in acetonitrile before (left) and after (right) chirp correction. 69

Figure S47. TAS data and fits of 3[nBuPh] in acetonitrile. 70

Figure S48. TAS heat map of 80 μM solution of 2[EtPh] in acetonitrile before (left) and after (right) chirp correction. 71

Figure S49. TAS data and fits of 2[EtPh] in acetonitrile. 72

Figure S50: Single wavelength kinetic traces and fits of 2[EtPh] at 500 nm (top), 525 nm (middle), and bottom (540 nm) across the ground state bleach feature. Lifetimes resulting from the best fit are shown at the top. 73

Figure S51: Single wavelength kinetic traces and fits of n[nBuPh] at 495 nm (top), 529 nm (middle), and bottom (565 nm) across the ground state bleach feature. Lifetimes resulting from the best fit are shown at the top. 74

Figure S52: Single wavelength kinetic traces of 4[Hexyl] at 500 nm (top), 510 nm (middle), and bottom (525 nm) across the ground state bleach feature. Lifetimes resulting from the best fit are shown at the top. 75

Computational Calculations 76

Figure S53: Electronic transition states of 2[EtPh], 3[nBuPh], 4[Hexyl]. 77
Figure S54: Molecular orbital diagram of 2[EtPh] and predicted PL mechanism. B3LYP-D3/6-31+G(d,p)/PCM(ACN). 78
Figure S55: Molecular orbital diagram of 3[nBuPh] and predicted PL mechanism. B3LYP-D3/6-31+G(d,p)/PCM(ACN). 78
Figure S56: Molecular orbital diagram of 4[Hexyl] and predicted PL mechanism. B3LYP-D3/6-31+G(d,p)/PCM(ACN). 79
Figure S57: Diameter of simulated geometry structure of 3[nBuPh] to calculate bare radius of molecule (7.53 Å) using PyMol software. B3LYP-D3/6-31+G(d,p)/PCM(ACN). 79
Figure S58: Geometry structure of 2[EtPh] GS (green) and opt S1 (cyan). B3LYP-D3/6-31+G(d,p)/PCM(ACN). 80
Figure S59: Geometry structure of 3[nBuPh] GS (blue) and opt S1 (purple). B3LYP-D3/6-31+G(d,p)/PCM(ACN). 80
Figure S60: Geometry structure of 4[Hexyl] GS (blue) and opt S1 (red). B3LYP-D3/6-31+G(d,p)/PCM(ACN). 81
Figure S61: Potential energy curve (PEC) of 3[nBuPh]. 81
Figure S62: Three dimers cut from the 1[Ph] crystal structure and the optimized 2[EtPh] and 3[nBuPh] dimers. The long 2[EtPh] and 3[nBuPh] dimers had have the C-C intermolecular bond and the intermolecular dihedral constrained to not optimize to an eclipsed structure. B3LYP-D3/6-31+G(d,p)/PCM(ACN). 82
Figure S63: TDDFT of the 2[EtPh] and 3[nBuPh] monomers and dimers. 83
Figure S64: TDDFT of the crystal structure and 2[EtPh], and optimized structure of 3[nBuPh] (left to right) of the long dimer. 83
Table S3: Basis set superposition error (BSSE) and corrected interaction energies of 2[EtPh] and 3[nBuPh] ring overlap and eclipsed dimers. B3LYP-D3/6-31+G(d,p)/PCM(ACN). 84
Table S4: First 30 TDDFT singlet transitions of 2[EtPh] monomer at the ground state singlet geometry. B3LYP-D3/6-31+G/PCM(ACN). 84
Table S5: First 30 TDDFT singlet transitions of 3[nBuPh] monomer at the ground state singlet geometry. B3LYP-D3/6-31+G/PCM(ACN). 86
Table S6: First 30 TDDFT singlet transitions of 4[Hexyl] monomer at the ground state singlet geometry. B3LYP-D3/6-31+G/PCM(ACN). 89
Table S7: First 20 TDDFT singlet transitions of singlet 2[EtPh] overlapping ring dimer. B3LYP-D3/6-31+G/PCM(ACN). 91
Table S8: First 20 TDDFT singlet transitions of singlet 3[nBuPh] overlapping ring dimer. B3LYP-D3/6-31+G/PCM(ACN). 104
Table S9: First 30 TDDFT singlet transitions of singlet 2[EtPh] eclipsed dimer. B3LYP-D3/6-31+G/PCM(ACN). 116
Table S10: First 20 TDDFT singlet transitions of singlet 3[nBuPh] eclipsed dimer. B3LYP-D3/6-31+G/PCM(ACN). 138
Table S11: First 30 TDDFT singlet transitions of singlet 1[Ph] crystal long dimer. B3LYP-D3/6-31+G/PCM(ACN). 147

Table S12: First 20 TDDFT singlet transitions of singlet 2[EtPh] constrained long dimer. B3LYP-D3/6-31+G/PCM(ACN). 173

References 183

General synthetic methods

All commercially available reagents obtained from suppliers were used without further purification. Unless otherwise noted, all reactions were carried out under nitrogen with standard Schlenk technique, and all glassware used in dry reactions were flame dried under high-vacuum prior to use. Tetrahydrofuran (THF), dimethylformamide (DMF), dichloromethane (DCM), and toluene were purified and dried by passing through two columns of neutral alumina, under nitrogen, prior to use. Flash chromatography was performed using VWR® High Purity 60Å (particle size 40-60 µm) silica gel. All 1 H and 13C NMR spectra were obtained with a Bruker AV-400 or a Bruker DRX-500 NEO with a CryoPlatform cryoprobe. Carbon spectra were measured with a proton-decoupling pulse program. Data from high-resolution mass spectrometry (HRMS) using electrospray ionization (ESI) were obtained by the Notre Dame mass spectrometry facility.

Synthetic procedures and characterization

1,4-dibromo-2,5-dimethoxybenzene (CAS #2674-34-2) was purchased from Sigma-Aldrich or synthesized according to the literature.[1] Phenalenone (CAS #548-39-0) was purchased from Sigma-Aldrich.

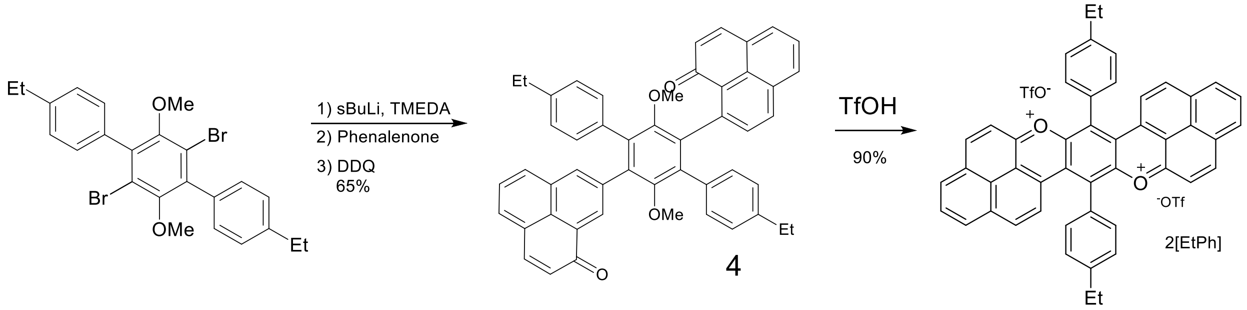

Scheme S1: Synthetic sequence to obtain ethyl-substituted bisphenalenyl 2[EtPh].

9,9'-(4,4''-diethyl-3',6'-dimethoxy-[1,1':4',1''-terphenyl]-2',5'-diyl) bis(1H-phenalen-1-one) (4) A flame dried RBF was charged with **6** (4.48 g, 8.89 mmol, 1.0 equiv.). The flask was backfilled with nitrogen and TMEDA (5.3 mL, 35.6 mmol, 4.0 equiv.) was added along with dry THF (120 mL). The solution was cooled to -78ºC for 20 min before 1.3 M sBuLi in hexanes (27.4 mL, 35.6 mmol, 4.0 equiv.) was added dropwise. The reaction was left to stir at -78ºC for 45 min, then phenalenone (3.20 g, 17.8 mmol, 2.0 equiv.) dissolved in dry THF (15 mL) was added slowly to the reaction mixture. The reaction was kept at -78ºC for 20 min before warming to room temperature and allowed to stir overnight. The reaction was quenched with saturated NH_4Cl and extracted with DCM to yield a suspension. The reaction was then concentrated to a slurry via rotary evaporation and filtered through a 0.22 µm nylon filter. The resulting orange solid was transferred

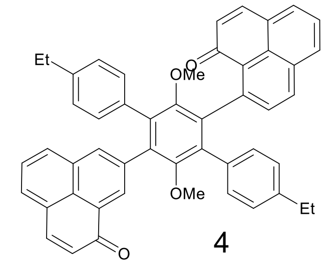

to a 100 mL RBF, which was then charged with DCM (50 mL) and DDQ (4.04 g, 17.8 mmol, 2.0 equiv.). The flask was heated to 50ºC and allowed to stir for 1 hour. The reaction was poured into a separatory funnel, followed by 4 M NaOH (20 mL). The separatory funnel was shaken vigorously until the organic layer turned orange, which was then extracted 3 times with DCM, dried under $MgSO_4$, and concentrated under rotary evaporation to furnish 4.06 g (5.78 mmol, 65% yield) as an orange solid. ^{1}H NMR (500 MHz, $CDCl_3$): δ 8.01 (d, J = 8.0 Hz, 2H), 7.94 (d, J = 8.0, 2H), 7.68 (d, J = 7.0 Hz, 2H), 7.67 (d, J = 8.5 Hz, 2H), 7.62 (d, J = 10.0 Hz, 2H), 7.54 (dd, J = 8.0, 7.0 Hz, 2H), 7.10 (d, J = 7.5 Hz, 4H), 6.79 (m, 8.0 Hz, 4H), 6.59 (d, J = 9.5 Hz, 2H), 2.88 (s, 6H), 2.39 (q, J = 7.5 Hz, 4H), 1.01 (t, J = 7.5 Hz, 6H). ^{13}C NMR (125 MHz, $CDCl_3$): δ 185.9, 150.5, 143.5, 142.0, 140.2, 136.6, 134.2, 133.3, 133.0, 132.9, 131.6,

131.3, 130.7, 130.4, 130.1, 129.4, 128.3, 128.0, 127.8, 127.7, 126.6, 126.0, 60.3, 28.4, 15.2. HRMS (ESI, m/z) calcd. for $C_{50}H_{39}O_4$ $[M+H]^+$: 703.2843; found: 703.2842.

[di(4-ethylphenyl)-pyrano[2,3-g] chromene]$^{2+}$ bis(trifluoromethanesulfonate) [2[EtPh]**]** A screw top vial was charged with 1,1,2,2-tetrachloroethane (TCE, 1 mL) and trifluoromethanesulfonic acid (62.9 µL, 0.711 mmol, 5 equiv.). A solution of **7** (100 mg, 0.142 mmol, 1 equiv.) in TCE (5 mL) was added to the vial dropwise, and then the reaction mixture was allowed to stir at 120°C for 12 hours, during which the reaction turned from dark red to magenta in color. Afterwards, the mixture was added to diethyl ether to precipitate a metallic brown solid (90.0 mg, 0.1391 mmol, 90% yield) that was collected by filtration. (120 mg, 0.128 mmol, 90% yield). ^{1}H NMR (500 MHz, 5:1 CD3CN:CF_3COOD): δ 9.44 (d, J = 9.0 Hz, 2H), 9.29 (d, J = 7.5 Hz, 2H), 9.19 (d, J = 8.0 Hz, 2H), 9.13 (d, J = 9.0 Hz, 2H), 8.62 (t, J = 8.0 Hz, 2H), 8.21 (d, J = 8.5 Hz, 2H), 8.20 (d, J = 8.5 Hz, 2H), 7.79-7.72 (m, 8H), 3.03 (q, J = 7.5 Hz, 4H), 1.52 (t, J = 7.5 Hz, 6H). ^{13}C NMR (125 MHz, 1:1 CD3CN:CF_3COOD): δ 165.8, 151.6, 149.0, 148.8, 147.3, 144.71, 142.6, 133.8, 132.6, 132.2, 131.6, 131.4, 131.3, 131.1, 130.9, 129.6, 129.2, 128.1, 123.8, 122.1, 120.8, 29.8, 16.2. ^{19}F NMR (376 MHz, CF_3COOH): δ -79.28. HRMS (ESI, m/z) calcd. for $C_{48}H_{32}O_2$ $[M]^{2+}$: 640.2391; found: 640.2379.

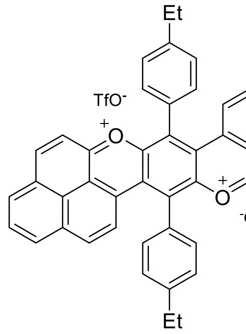

2[EtPh]

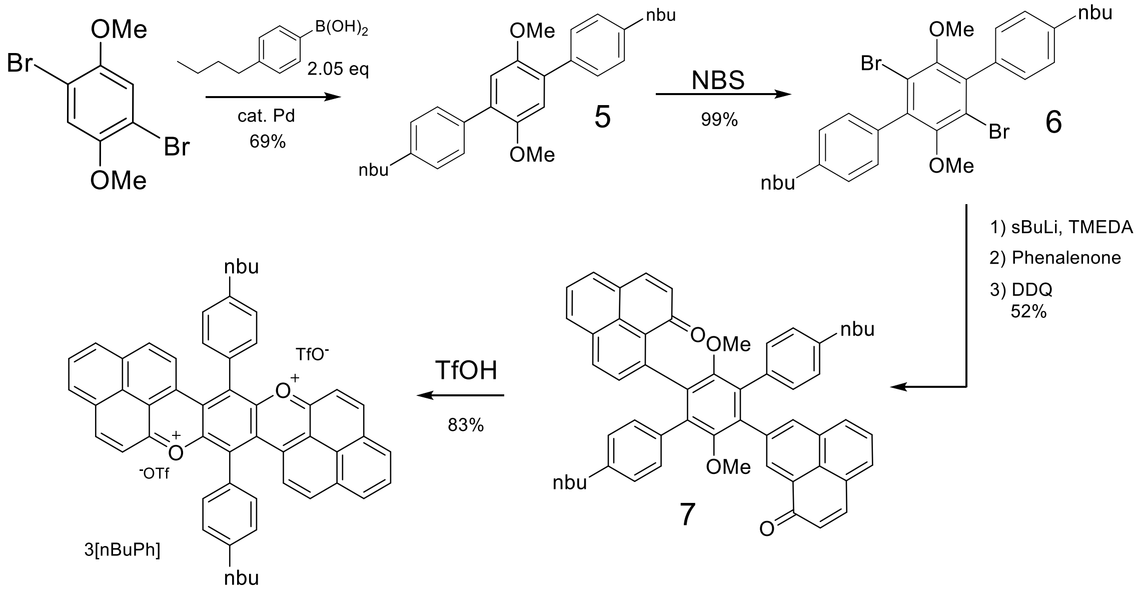

Scheme S2: Synthetic sequence to obtain nbutyl-substituted bisphenalenyl 3[nBuPh].

4,4"-dibutyl-2'5'-dimethoxy-1,1':4',1"-terphenyl (5**)** A flame dried RBF was charged with 1, 4-dibromo2,5-dimethoxybenzene (10.3 g, 39.6 mmol, 1.0 equiv.), 1-butylphenylboronic acid (9.92 g, 81.4 mmol, 2.05 equiv.), potassium carbonate (19.2 g, 139 mmol, 3.5 equiv.), toluene (80 mL), deionized water (40 mL), and a stir bar. $Pd_2(dba)_3$ (457 mg, 0.395 mmol, 1 mol%) and PPh_3 (457 mg, 0.395 mmol, 1 mol%) was added last to the flask before the reaction mixture was lowered into a bath that was preheated to 90 °C and stirred for 12h with a condenser. Upon completion, the reaction was diluted with water, extracted 3 times with DCM, dried on MgSO4, and concentrated via rotary evaporation. The solid was then recrystallized in EtOH to provide

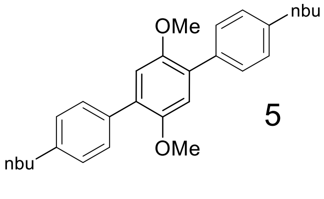

5

9.01 g (31.1 mmol, 78% yield) of white needles. ^{1}H NMR (400 MHz, $CDCl_3$): δ 7.51 (d, J=8.0 Hz, 2H), 6.98 (s, 1H), 3.80 (s, 3H), 2.68 (t, J=7.7, 7.9 Hz, 2H), 1.71-1.63 (m, J=7.2, 7.9, 7.6, 8.2 Hz, 2H), 1.47-1.38 (m, J=7.2, 7.5, 7.5, 7.4, 7.3 Hz, 2H), 0.97 (t, J= 7.3, 7.4 Hz, 3H). ^{13}C NMR (100 MHz, $CDCl_3$): δ 150.7, 141.9, 135.7, 130.2, 129.4, 128.3, 114.8, 56.5, 35.6, 33.8, 22.6, 14.15. HRMS (ESI, *m/z*) calcd. for $C_{28}H_{34}O_2$: 402.2555; found: 402.2559.

2',5'-dibromo-4,4''-dibutyl-3',6'-dimethoxy-1,1':4',1''-terphenyl (6)
A flame dried RBF was charged with 5 (4.0 g, 13.8 mmol, 1.0 equiv.), ammonium nitrate (221 mg, 2.76 mmol, 0.2 equiv.), 1:1 DCM/acetonitrile (250 mL) and a stir bar. N-Bromosuccinimide (6.14 g, 34.5 mmol, 2.5 equiv.) was added portionwise to the reaction flask and the reaction was allowed to stir for 24h at room temperature. Upon completion, the reaction was washed with H2O, extracted 3 times with DCM, dried on MgSO4, and concentrated via rotary evaporation. The solid was then recrystallized in Acetone to provide 5.26 g (11.8 mmol, 86% yield) of white crystals. ^{1}H NMR (400 MHz, $CDCl_3$): δ 7.28 (t, J=2.3, 2.8 Hz, 4H), 3.42 (s, 3H), 2.71 (t, J=7.8, 7.8 Hz, 2H), 1.73-1.65 (m, J=7.5, 7.8, 7.5, 7.8 Hz, 2H), 1.47-1.38 (m, J=7.5, 7.4, 7.5, 7.4, 7.3 Hz, 3H), 0.98 (t, J=7.4, 7.3 Hz, 3H). ^{13}C NMR (100 MHz, $CDCl_3$): δ152.1, 142.8, 137.9, 134.1, 129.9, 128.2, 119.6, 60.7, 35.6, 35.5, 22.6, 14.2. HRMS (ESI, *m/z*) calcd. for $C_{28}H_{32}O_2Br_2$: 558.0829; found: 558.0769.

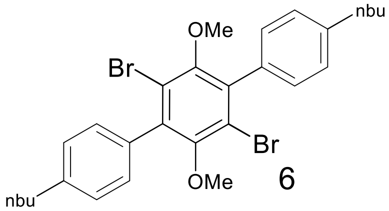

6

9,9'-(4,4''-dibutyl-3',6'-dimethoxy-[1,1':4',1''-terphenyl]-2',5'-diyl)bis(1H-phenalen-1-one) (7) A flame dried RBF was charged with 6 (4.0 g, 8.89 mmol, 1.0 equiv.). The flask was backfilled with nitrogen and TMEDA (5.4 mL, 35.6 mmol, 4.0 equiv.) was added along with 120 mL dry THF. The solution was cooled to -78ºC for 20 min before 1.3 M sBuLi in hexanes (27.4 mL, 35.6 mmol, 4.0 equiv.) was added dropwise. The reaction was left to stir at - 78ºC for 45 min, then phenalenone (3.20 g, 17.8 mmol, 2.0 equiv.) dissolved in dry THF (15 mL) was added slowly to the reaction mixture. The reaction was kept at -78ºC for 20 min before warming to room temperature and allowed to stir overnight. The reaction was quenched with saturated NH4Cl and extracted with DCM to yield a suspension. The reaction was then concentrated to a slurry via rotary evaporation and filtered through a 0.22 µm nylon filter. The resulting orange solid was then transferred into a 100 mL round bottom flask, which was then charged with DCM (50 mL) and DDQ (4.03 g, 17.8 mmol, 2.0 equiv.). The flask was heated to 50ºC and allowed to stir for 1 hour. The reaction was poured into a separation funnel followed by 20 mL of a 4M NaOH solution. The separation funnel was shaken vigorously until the organic layer turned orange, which was then extracted 3 times with DCM, dried under MgSO4, and concentrated under rotary evaporation to furnish 3.74 g (5.78 mmol, 65% yield) as an orange solid. ^{1}H NMR (400 MHz, $CDCl_3$): δ 8.03 (d, *J*=8.5 Hz, 2H), 7.95 (d, *J*= 7.9 Hz, 2H), 7.71 (d, *J*= 8.40 Hz, 2H), 7.68 (d, *J*=7.03 Hz, 2H), 7.61 (d, J=10.0 Hz, 2H), 7.56 (t, J=8.1, 7.1 Hz, 2H), 7.07 (d, J=8.12 Hz, 1H), 6.76 (d, J=8.1 Hz, 1H), 6.58 (d, J=9.6 Hz, 1H), 2.90 (s, 1H), 2.35 (t, J=8.0, 7.3 Hz, 1H), 1.33 (m, J=7.4, 7.5, 7.6, 7.6 Hz, 2H), 1.08-0.99 (m, J=7.5, 7.5, 7.5, 7.3, 7.3 Hz, 2H), 0.74 (t, J=7.3, 7.4 Hz, 2H). ^{13}C NMR (100 MHz, $CDCl_3$): δ 185.8, 150.5, 143.5, 140.6, 140.1, 136.7, 134.2, 133.4, 133.0, 132.9, 131.6, 131.3, 130.7, 130.3, 130.0, 128.2, 127.9, 127.8, 127.2, 125.9, 60.3, 35.1, 33.2, 21.9, 13.9. HRMS (ESI, *m/z*) calcd. for $C_{54}H_{46}NaO_4$: 781.3257; found: 781.3288.

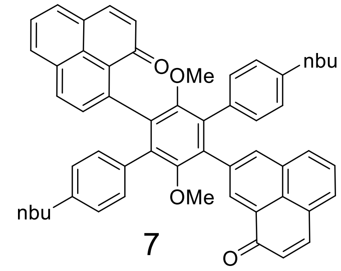

7

[di(4-butylphenyl)-pyrano[2,3-g]chromene]$^{2+}$ bis(trifluoromethanesulfonate) [3[nBuPh]] A screw top vial was charged with 1,1,2,2- tetrachloroethane (TCE, 1 mL) and trifluoromethanesulfonic acid (68.3 µL, 0.773 mmol, 5 equiv.). A solution of 7 (100 mg, 0.155 mmol, 1 equiv.) in TCE (5 mL) was added to the vial dropwise, and then the entire reaction mixture was allowed to stir at 120°C for 12h, during which time the reaction changed from a dark red to magenta color. Afterwards, the mixture added to diethyl ether to precipitate a metallic brown solid, which was collected by filtration. (90.0 mg, 0.1391 mmol, 90% yield). ^{1}H NMR (400 MHz, CD3CN): δ 9.47 (d, J=9.4 Hz, 2H), 9.31 (d, 7.7 Hz, 2H), 9.22 (d, 7.7 Hz, 2H), 9.12 (d, J=9.2 Hz, 2H), 8.63 (t, J=7.7, 7.9 Hz, 2H), 8.22 (d, J=9.0 Hz, 2H), 8.17 (d, 9.0 Hz, 2H), 7.78-7.71 (q, J=8.2, 8.8, 8.3 Hz, 6H), 6.31 (s, 1H), 3.00 (t, J=7.6, 7.8 Hz, 4H), 1.78-1.76 (m, J=2.5, 2.5, 2.5, 2.5 Hz, 2H), 1.65-1.56 (m, J=7.3, 7.6, 7.6, 7.3, 7.2 Hz, 6H), 1.3 (t, J=7.4, 7.4 Hz, 6H). ^{13}C NMR (100 MHz, CD3CN): δ 165.6, 151.5, 148.8, 147.0, 144.51, 142.3, 133.5, 132.5, 132.0, 131.5, 131.4, 131.2, 128.9, 127.9, 123.7, 121.9, 120.6, 75.4, 36.2, 34.5, 23.1, 14.3.HRMS (ESI, *m/z*) calcd. for $C_{52}H_{41}O_3[M]^{2+}$: 713.3037; found: 713.050.

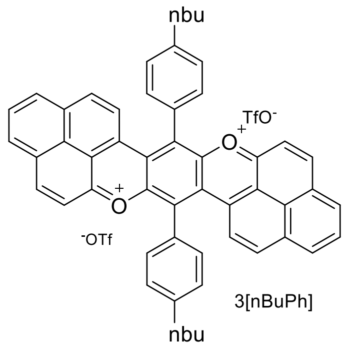

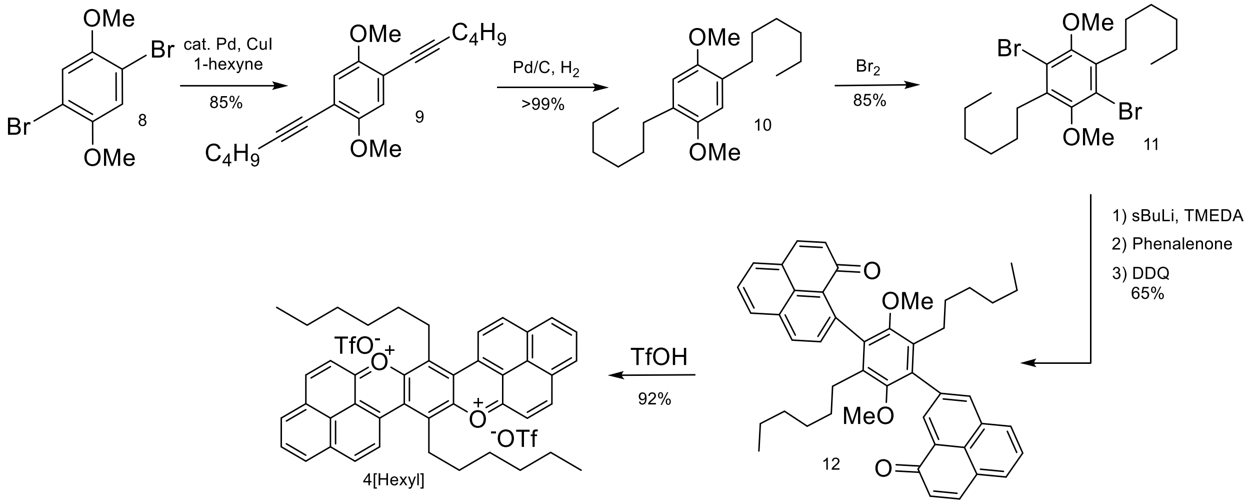

Scheme S3: Synthetic sequence to obtain nhexyl-substituted bisphenalenyl 4[Hexyl].

1,4-di(hex-1-yn-1-yl)-2,5-dimethoxybenzene A solution of triethylamine (27.97 mL) and piperidine (83.9 mL) was added to a flame dried RBF and degassed for 30 minutes. 1,4-dibromo2,5-dimethoxybenzene (14) (5.00 g, 16.78 mmol, 1.0 equiv.), 1-hexyne (6.75 mL, 58.73 mmol, 3.5 equiv.), copper(I) iodide (639 mg, 3.36 mmol, 20 mol%), and a stir bar were added. The flask was backfilled with nitrogen and was heated to 80□C for 15 minutes. Catalytic $Pd(PPh_3)_4$ (1.94 g, 1.68 mmol, 10 mol%) was added to the flask last and was left stirring for 12 hours. The reaction was quenched with 1 M HCl and extracted 3 times with DCM, dried under $MgSO_4$, and concentrated under rotary evaporation and then purified by column chromatography (40% DCM/Hexanes) to finish 3.82 g (11.00 mmol, 65% yield) as a light yellow solid. ^{1}H NMR (500 MHz, $CDCl_3$): δ 6.83 (s, 2H), 3.79 (s, 6H), 2.46 (t, J=7.00, 7.50 Hz), 1.61-1.55 (q, J=6.8, 7.10, 7.95, 7.50 Hz, 4H), 1.49-1.42 (m, J=7.15, 7.50, 7.70, 7.40, 7.20 Hz, 4H), 0.9344 (t, J=7.15, 7.80 Hz, 6H). ^{13}C NMR (125 MHz, $CDCl_3$): δ 153.7, 115.7, 113.2, 96.0, 76.6, 56.4, 30.9, 22.1, 19.6, 13.7). HRMS (ESI, m/z) calcd. for $C_{20}H_{27}O_2$ $[M]^{+}$: 299.2006; found: 299.2006.

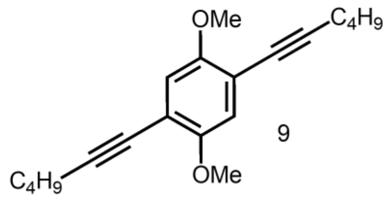

1,4-dihexyl-2,5-dimethoxybenzene A flame dried RBF was charged with **9** (and a stir bar then backfilled with H_2 gas. A solution of DCM (28 mL) and ethanol (28 mL) was added and degassed for 20 minutes. Palladium on carbon (10wt%) and left stirring for 10 hours at room temperature. The reaction was filtered through celite and concentrated under rotary evaporation to finish 3.61 g (11.78 mmol, 93% yield) as a light yellow solid. ^{1}H NMR (500 MHz, $CDCl_3$): δ 6.65 (s, 2H), 3.78 (s, 6H), 2.57 (t, J=7.7, 7.9 Hz, 4H), 1.56 (q, J=7.55, 6.85, 7.60, 7.75, 6H), 1.36-1.30 (m, 12H), 0.90 (t, J=6.45, 6.7 Hz, 6H). ^{13}C NMR (125 MHz, $CDCl_3$): δ 151.3, 129.4, 113.2, 56.3, 53.5, 31.9, 53.5, 31.9, 30.6, 30.3, 29.5, 22.8, 14.3. HRMS (ESI, m/z) calcd. for $C_{20}H_{35}O_2$ $[M]^+$: 307.2632; found: 307.2631.

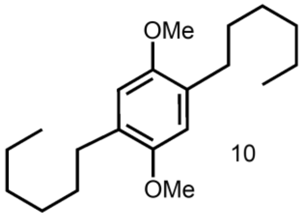

10

1,4-dibromo-2,5-dihexyl-3,6-dimethoxybenzene A flame dried RBF was charged with **10** (3.61 g, 11.78 mmol, 1.0 equiv.), acetic acid (118 mL), and was stirred until solution was clear. Bromine (3.02 mL, 58.9, 5.0 equiv.) was added and was left stirring for 12 hours. The reaction was quenched with saturated sodium bicarbonate (15 mL) and extracted 3 times with DCM, dried under $MgSO_4$, and concentrated under rotary evaporation to finish 5.52 g (##) as a light yellow solid. (^{1}H NMR (500 MHz, $CDCl_3$): δ 3.80 (s, 6H), 2.79 (t, J=8.05, 8.15 Hz, 4H), 1.58-1.51 (m, 5H), 1.45-1.39 (q, J=6.95, 7.55, 7.15, 7.20 Hz, 4H), 1.34-1.31 (m, 9H), 0.91-0.88 (t, J=6.8, 7.6 Hz, 6H). ^{13}C NMR (125 MHz, $CDCl_3$): δ 152.3, 136.1, 119.7, 61.3, 31.6, 31.5, 29.6, 29.6, 22.7, 14.2. HRMS (ESI, m/z) calcd. for $C_{20}H_{33}Br_2O_2$ $[M]^+$: 463.0853; found: 463.0842.

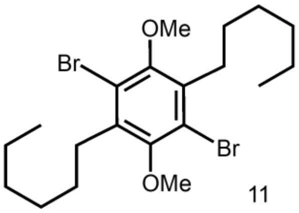

11

9,9'-(2,5-dihexyl-3,6-dimethoxy-1,4-phenylene)bis(1*H*-phenalen-1-one) A flame dried RBF was charged with 11 (4.0 g, 8.89 mmol, 1.0 equiv.). The flask was backfilled with nitrogen and TMEDA (5.4 mL, 35.6 mmol, 4.0 equiv.) was added along with 120 mL dry THF. The solution was cooled to -78ºC for 20 min before 1.3 M sBuLi in hexanes (27.4 mL, 35.6 mmol, 4.0 equiv.) was added dropwise. The reaction was left to stir at - 78ºC for 45 min, then phenalenone (3.20 g, 17.8 mmol, 2.0 equiv.) dissolved in dry THF (15 mL) was added slowly to the reaction mixture. The reaction was kept at -78ºC for 20 min before warming to room temperature and stirred for 12 hours. The reaction was quenched with saturated NH4Cl and extracted with DCM to yield a suspension. The reaction was then concentrated to a slurry via rotary evaporation and filtered through a 0.22 μm nylon filter. The resulting orange solid was then transferred into a 100 mL round bottom flask, which was then charged with DCM (50 mL) and DDQ (4.03 g, 17.8 mmol, 2.0 equiv.). The flask was heated to 50ºC and allowed to stir for 1 hour. The reaction was poured into a separation funnel followed by 20 mL of a 4M NaOH solution. The separation funnel was shaken vigorously until the organic layer turned orange, which was then extracted 3 times with DCM, dried under MgSO4, and concentrated under rotary evaporation to furnish 3.74 g (5.78 mmol, 65% yield) as an orange solid. ^{1}H NMR (500 MHz, $CDCl_3$): δ 8.26 (d, 2H), 8.11 (d, 2H), 7.83 (d, 2H), 7.81 (d, 2H), 7.75 (d, 2H), 7.67-7.64 (m, 2H), 6.64 (d, 2H), 3.28 (s, 3H), 1.29-1.22 (m, 7H), 0.97-0.83 (m, 10H), 0.57-0.54 (t, 3H). ^{13}C NMR (125 MHz, $CDCl_3$): 185.6, 151.2, 140.4, 136.5, 133.5, 132.9, 131.7, 130.9, 130.1, 126.2, 60.7, 31.0, 29.7, 29.4, 27.9, 22.2, 13.8. HRMS (ESI, m/z) calcd. for $C_{46}H_{47}O_4$ $[M]^+$: 663.3469; found: 663.3452.

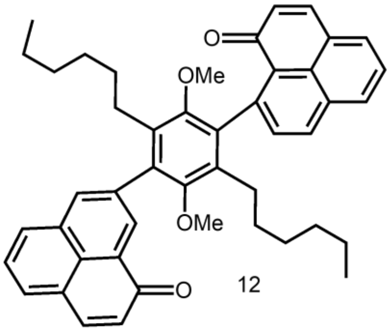

12

[di(4-hexyl)-pyrano[2,3-g] chromene]$^{2+}$ bis(trifluoromethanesulfonate) [4[Hexyl]**]** A screw top vial was charged with 1,1,2,2- tetrachloroethane (TCE, 1 mL) and trifluoromethanesulfonic acid (68.3 µL, 0.773 mmol, 5 equiv.). A solution of 12 (100 mg, 0.155 mmol, 1 equiv.) in TCE (5 mL) was added to the vial dropwise, and then the entire reaction mixture was allowed to stir at 120°C for 12h, during which time the reaction changed from a dark red to magenta color. Afterwards, the mixture added to diethyl ether to precipitate a metallic green solid, which was collected by filtration (680 mg, 0.756 mmol, 84%). ^{1}H NMR (500 MHz, CD3CN): δ 9.75 (d, 1H), 9.59 (t, J= Hz, 2H), 9.49 (d, 2H), 9.40 (d, 3H), 8.75-8.70 (m, J= Hz, 4H), 2.38-2.35 (t, J= Hz, 4H) ^{13}C NMR (125 MHz, CD3CN): δ 152.3, 136.1, 119.7, 61.3, 31.6, 31.5, 29.6, 29.6, 22.7, 14.2. HRMS (ESI, m/z) calcd. for $C_{44}H_{46}O_2$ $[M]^{2+}$: 606.3492; found: 606.3492

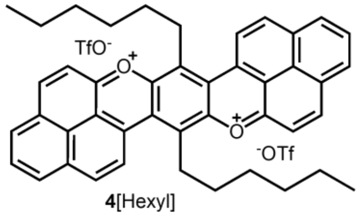

4[Hexyl]

^{1}H-NMR of 4

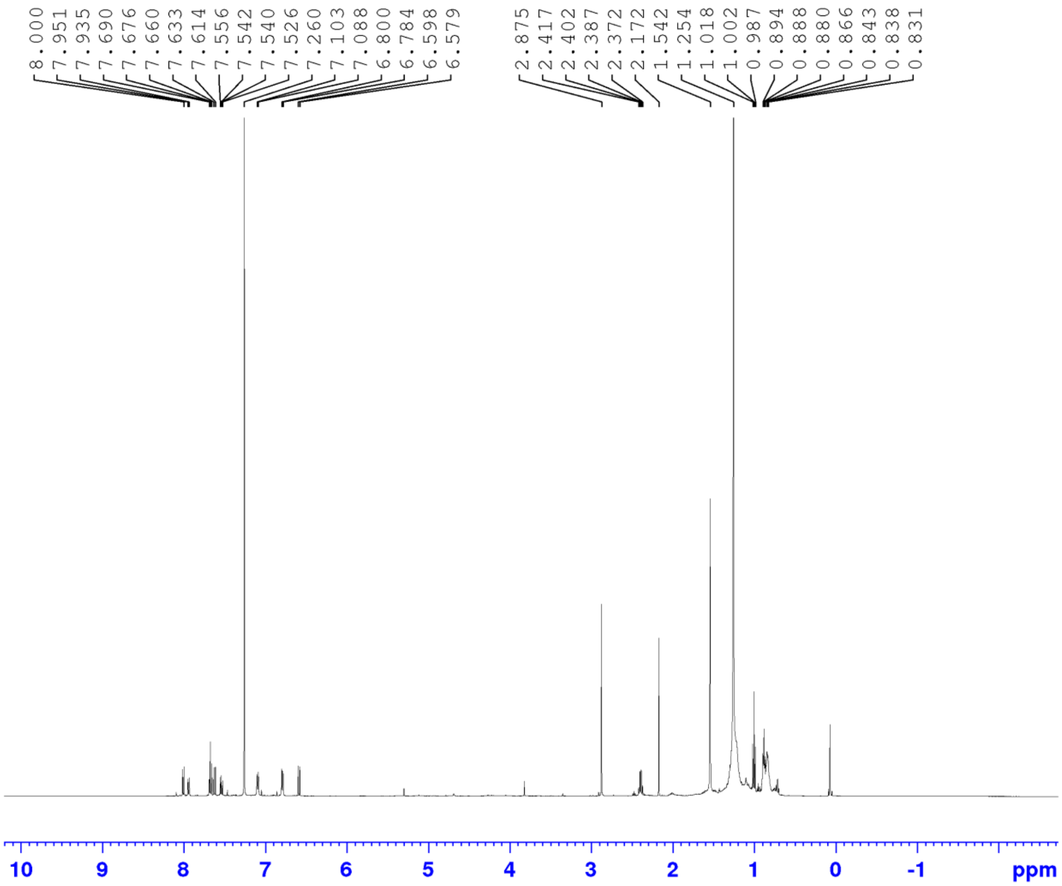
8.000
7.951
7.935
7.690
7.676
7.660
7.633
7.614
7.556
7.542
7.540
7.526
7.260
7.103
7.088
6.800
6.784
6.598
6.579
2.875
2.417
2.402
2.387
2.372
2.172
1.542
1.254
1.018
1.002
0.987
0.894
0.888
0.880
0.866
0.843
0.838
0.831
10
9
8
7
6
5
4
3
2
1
0
-1
ppm

^{13}C-NMR of 4

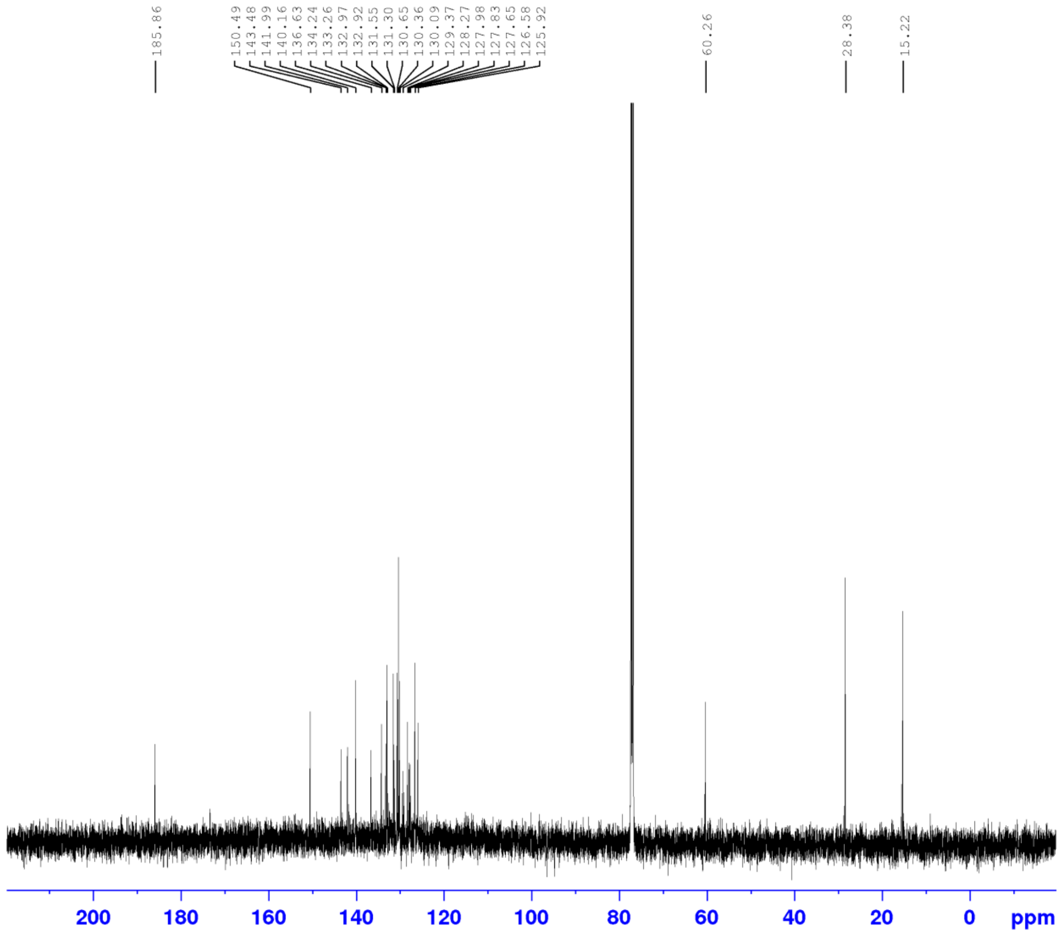

185.86
150.49
143.48
141.99
140.16
136.63
134.24
133.26
132.97
132.92
131.55
131.30
130.65
130.36
130.09
129.37
128.27
127.98
127.83
127.65
126.58
125.92
60.26
28.38
15.22
200
180
160
140
120
100
80
60
40
20
0
ppm

^{1}H-NMR of 2[EtPh]

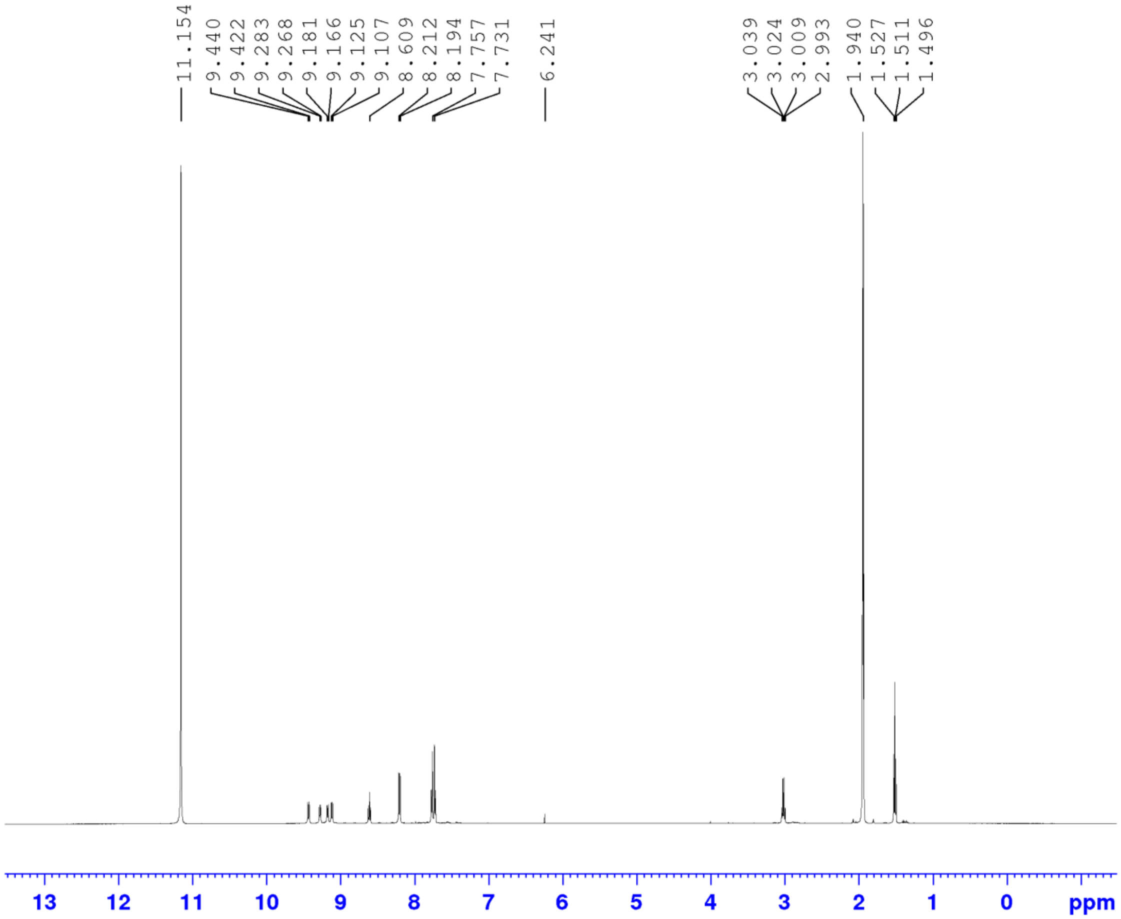

^{13}C NMR of 2[EtPh]

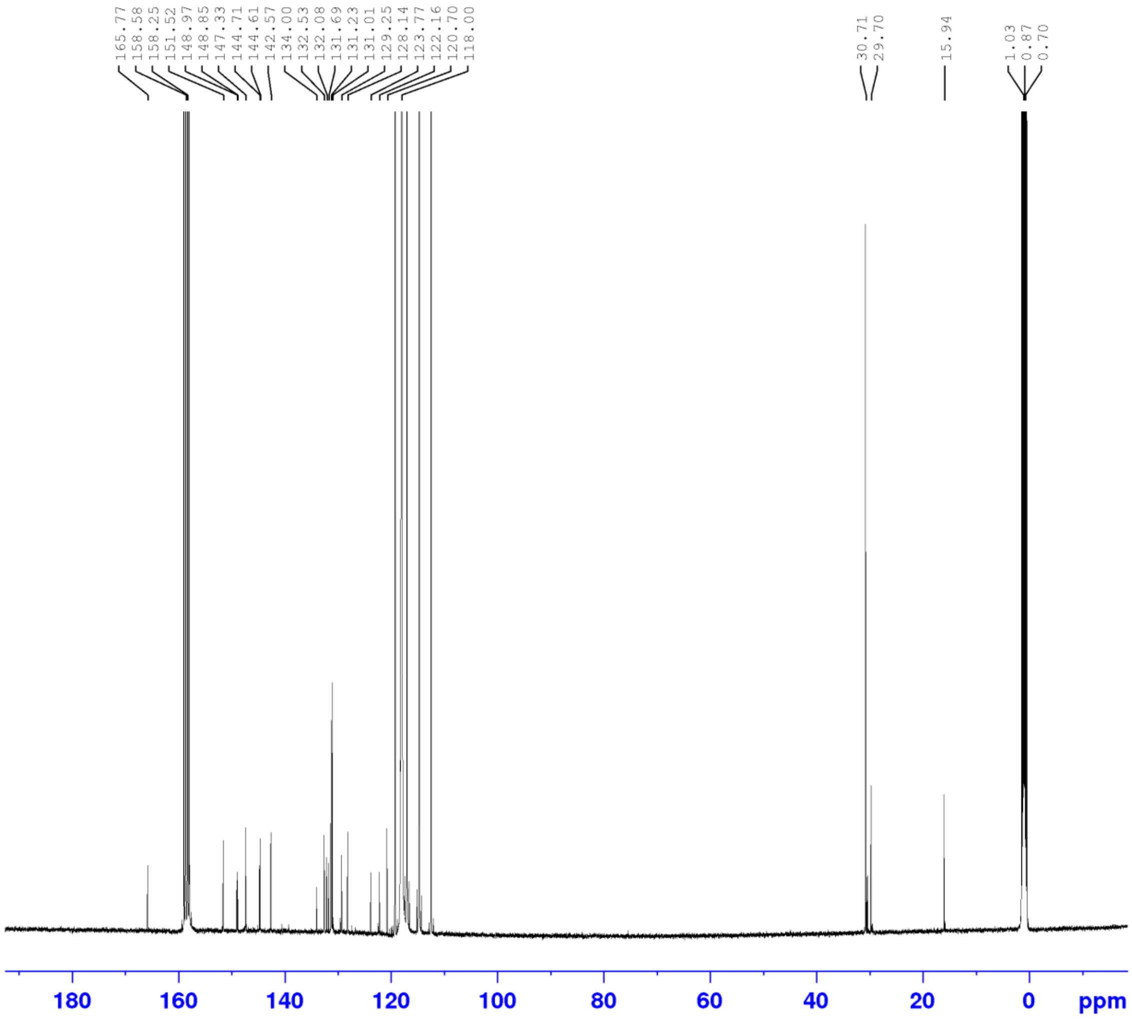

^{1}H-NMR of 5

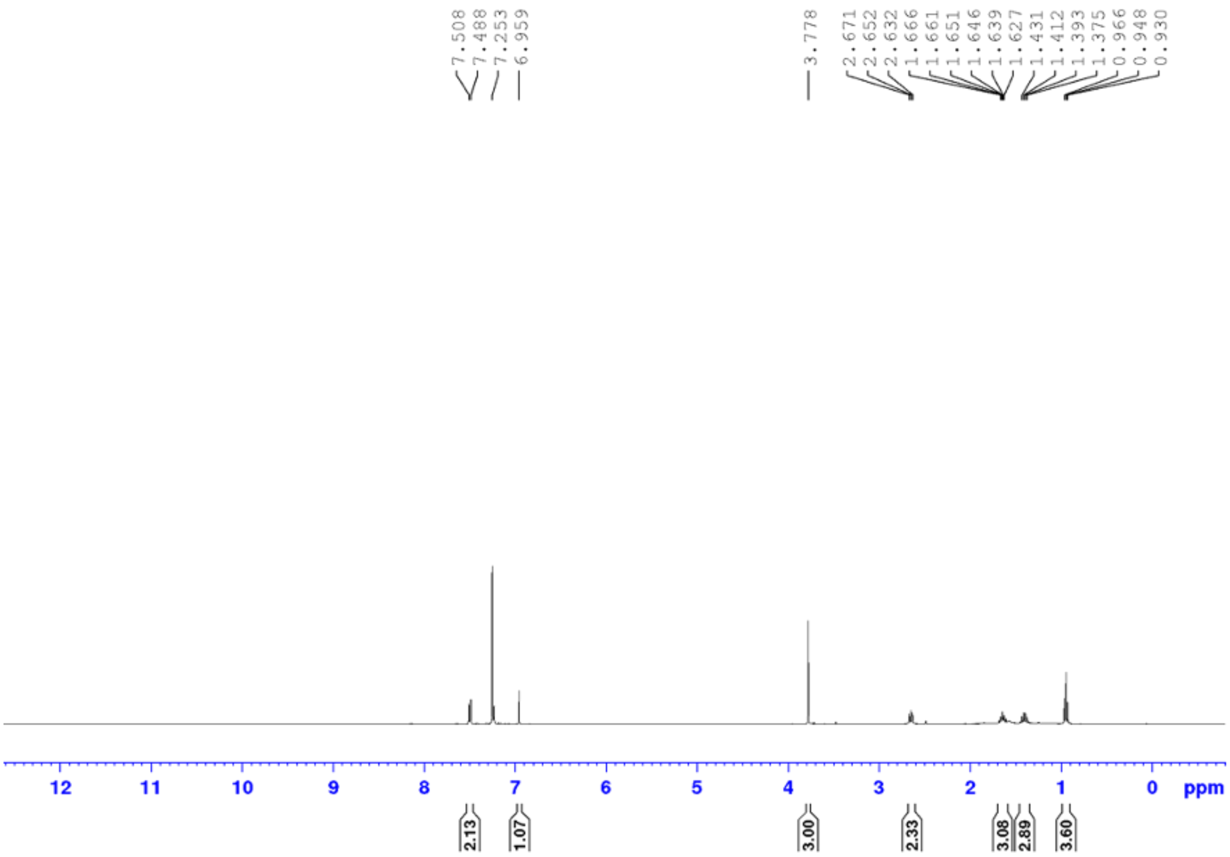
7.508
7.488
7.253
6.959
3.778
2.671
2.652
2.632
1.666
1.661
1.651
1.646
1.639
1.627
1.431
1.412
1.393
1.375
0.966
0.948
0.930
12 11 10 9 8 7 6 5 4 3 2 1 0 ppm
2.13
1.07
3.00
2.33
3.08
2.89
3.60

^{13}C NMR of 5

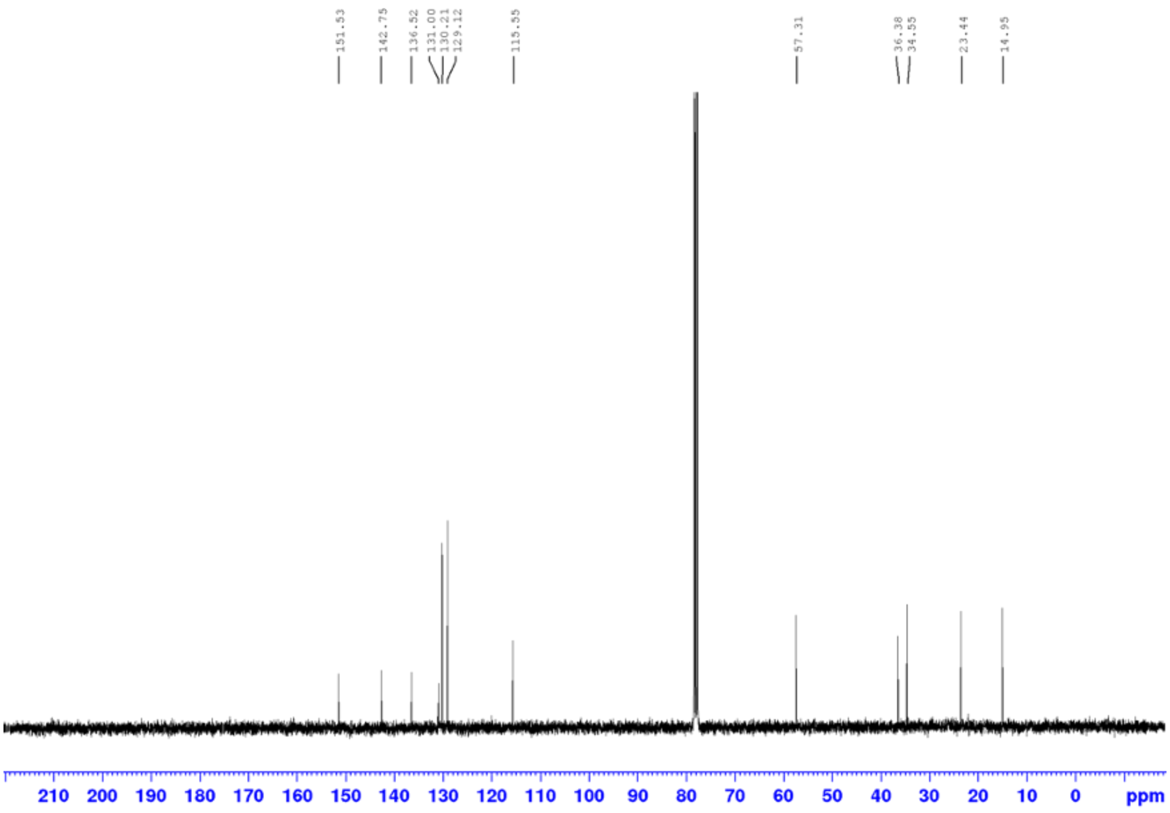
151.53
142.75
136.52
131.00
130.21
129.12
115.55
57.31
36.38
34.55
23.44
14.95
210 200 190 180 170 160 150 140 130 120 110 100 90 80 70 60 50 40 30 20 10 0 ppm

^{1}H-NMR of 6

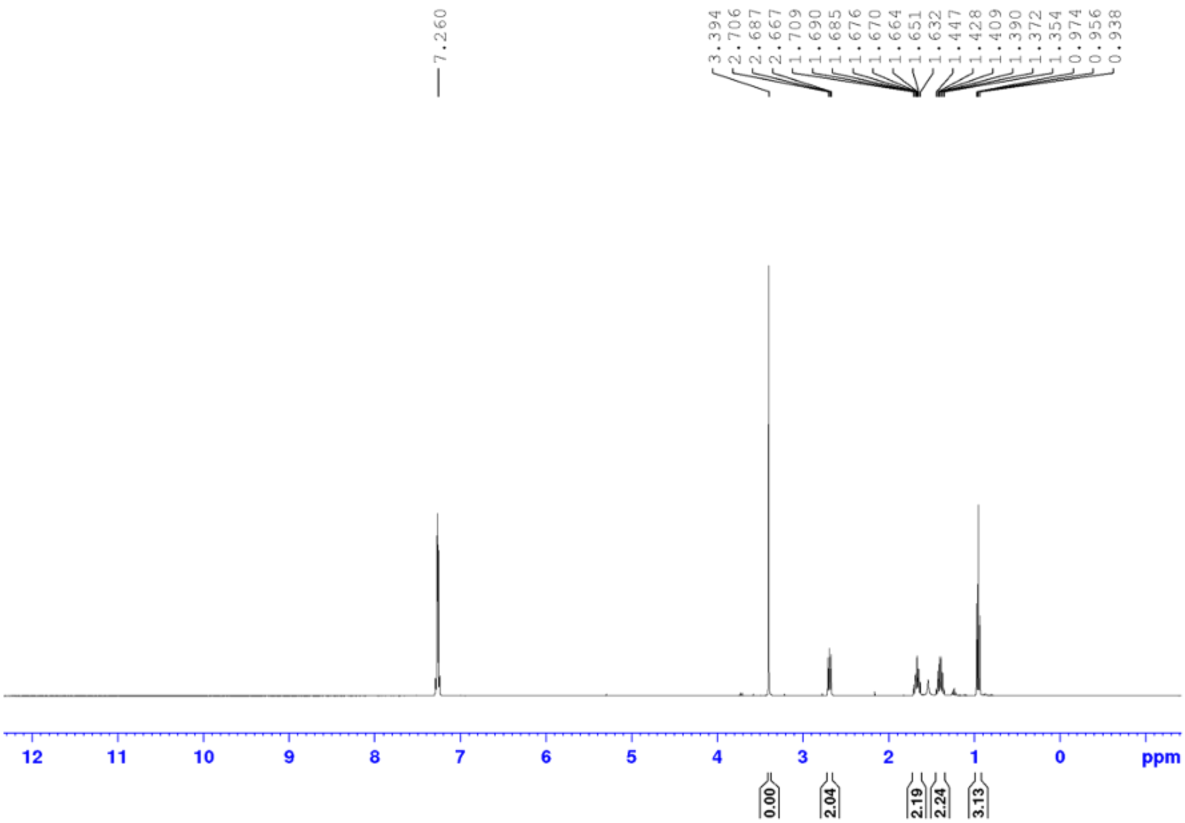
7.260
3.394
2.706
2.687
2.667
1.709
1.690
1.685
1.676
1.670
1.664
1.651
1.632
1.447
1.428
1.409
1.390
1.372
1.354
0.974
0.956
0.938
12
11
10
9
8
7
6
5
4
3
2
1
0
ppm
0.00
2.04
2.19
2.24
3.13

^{13}C-NMR of 6

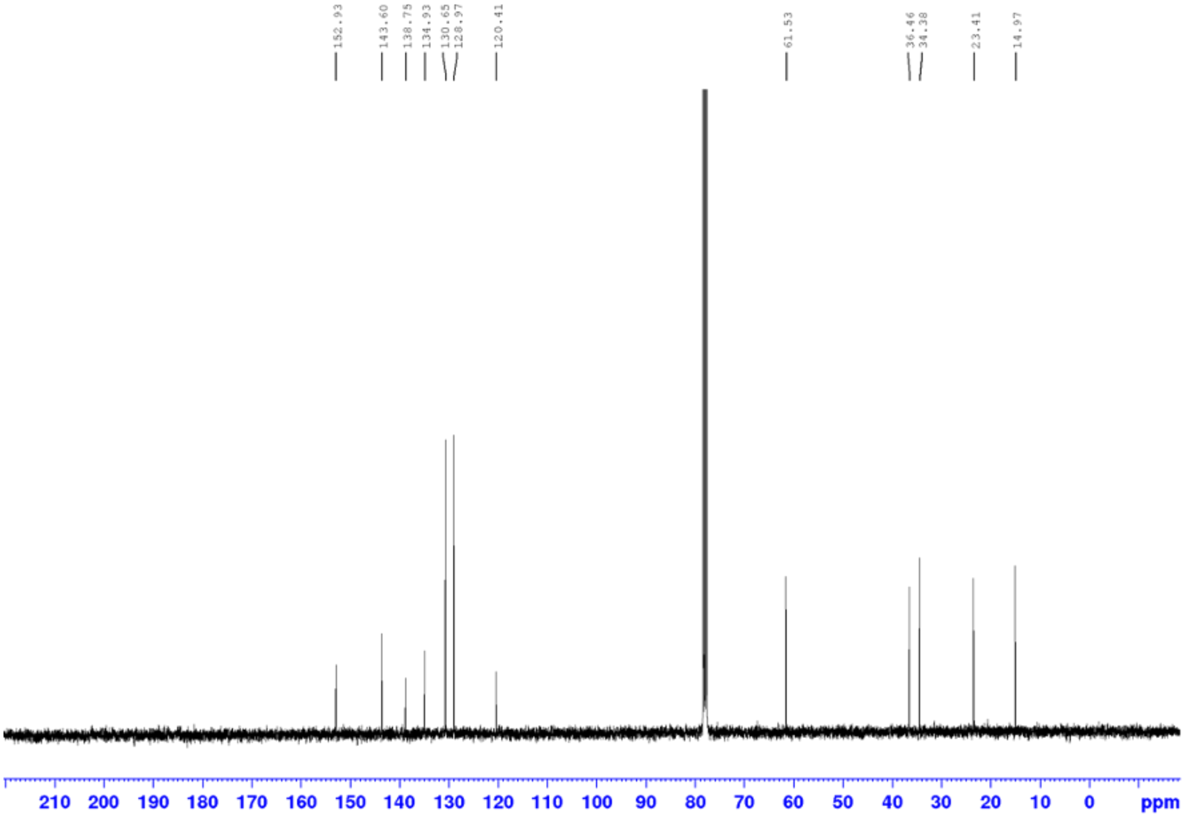

152.93
143.60
138.75
134.93
130.65
128.97
120.41
61.53
36.46
34.38
23.41
14.97
210 200 190 180 170 160 150 140 130 120 110 100 90 80 70 60 50 40 30 20 10 0 ppm

^{1}H-NMR of 7

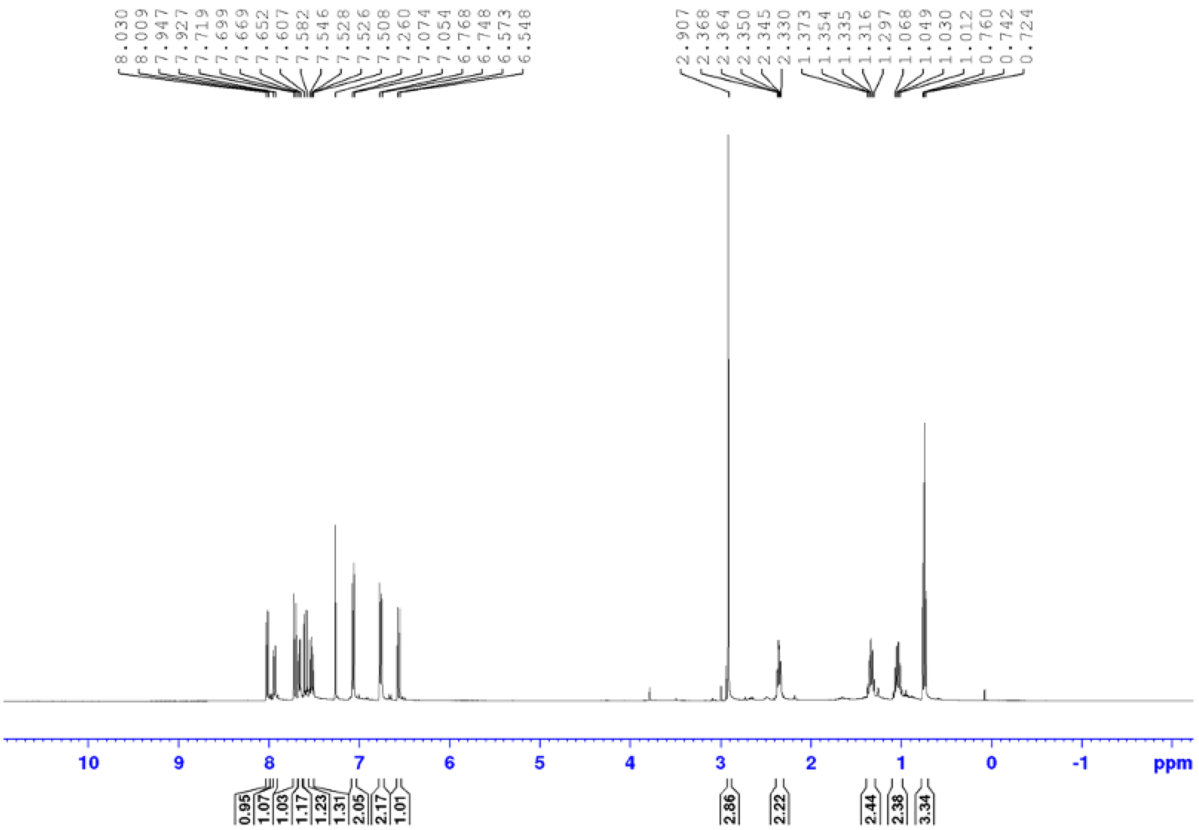

^{13}C-NMR of 7

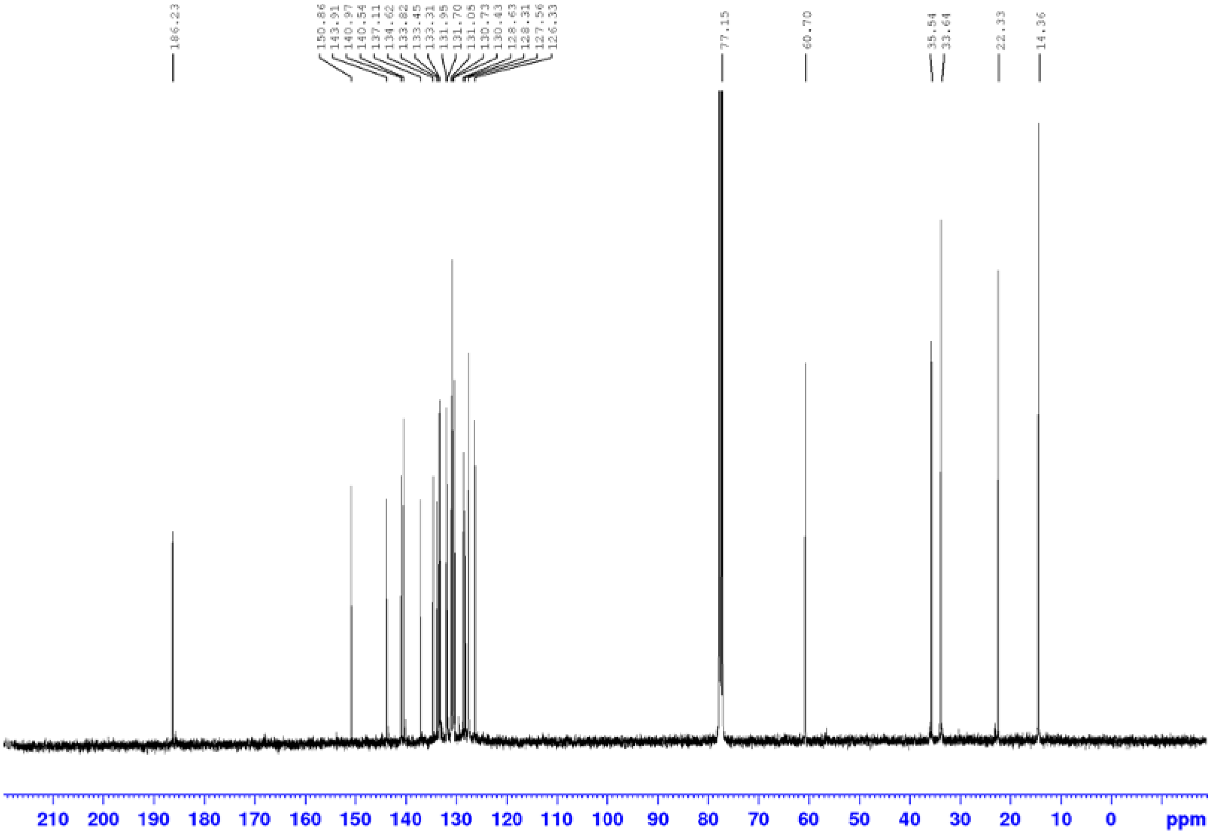

186.23
150.86
143.91
140.97
140.54
137.11
134.62
133.82
133.45
133.31
131.95
131.70
131.05
130.73
130.43
128.63
128.31
127.56
126.33
77.15
60.70
35.54
33.64
22.33
14.36
210 200 190 180 170 160 150 140 130 120 110 100 90 80 70 60 50 40 30 20 10 0 ppm

1H-NMR of 3[nBuPh]

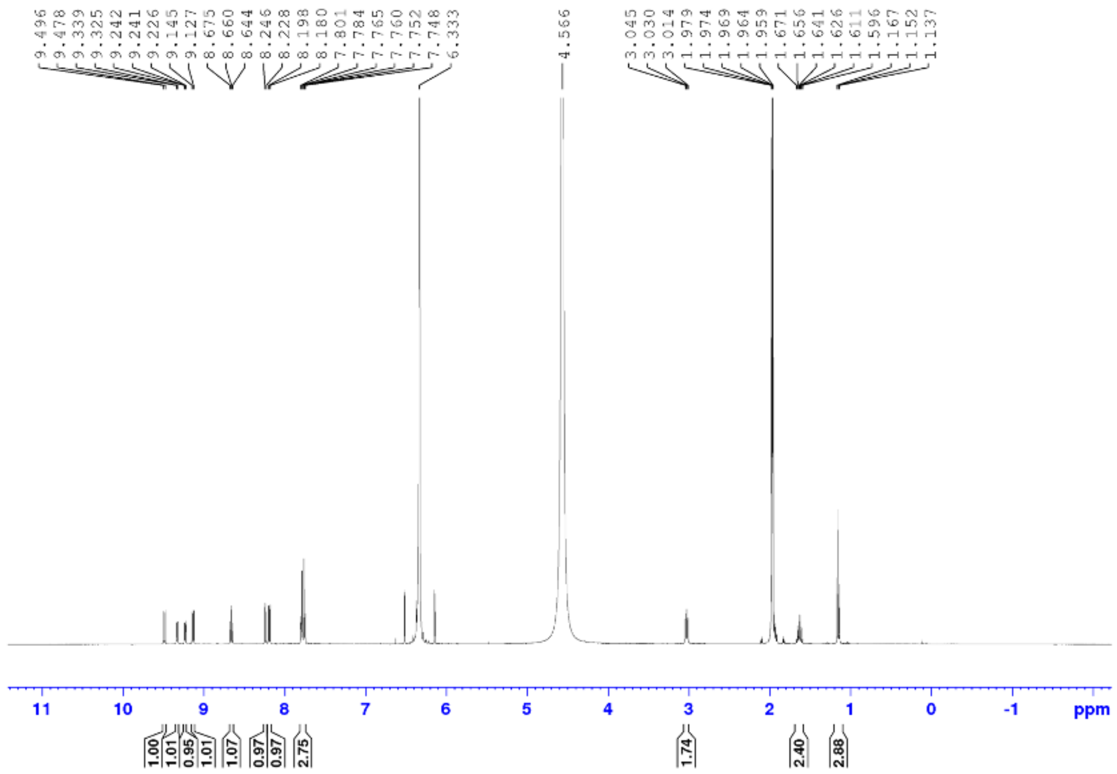

^{13}C-NMR of 3[nBuPh]

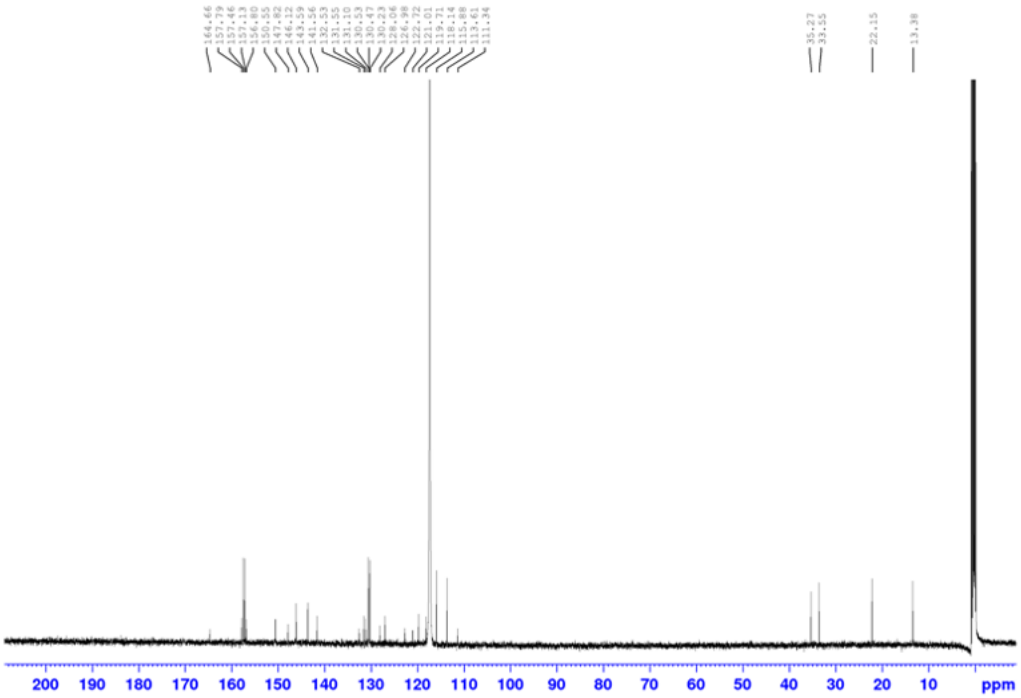

^{1}H-NMR of 9

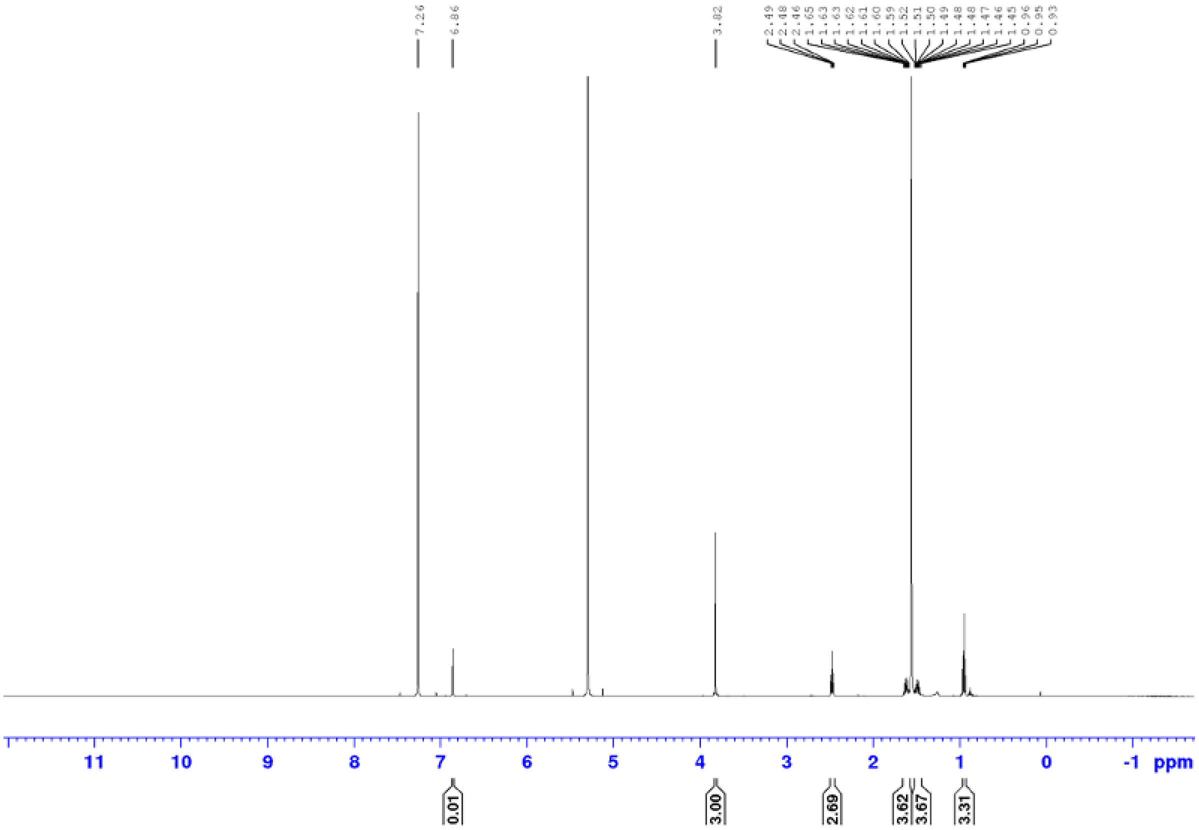
7.26
6.86
3.82
2.49
2.48
2.46
1.65
1.63
1.63
1.62
1.61
1.60
1.59
1.52
1.51
1.50
1.49
1.48
1.48
1.47
1.46
1.45
0.96
0.95
0.93
11
10
9
8
7
6
5
4
3
2
1
0
-1
ppm
0.01
3.00
2.69
3.62
3.67
3.31

^{13}C-NMR of 9

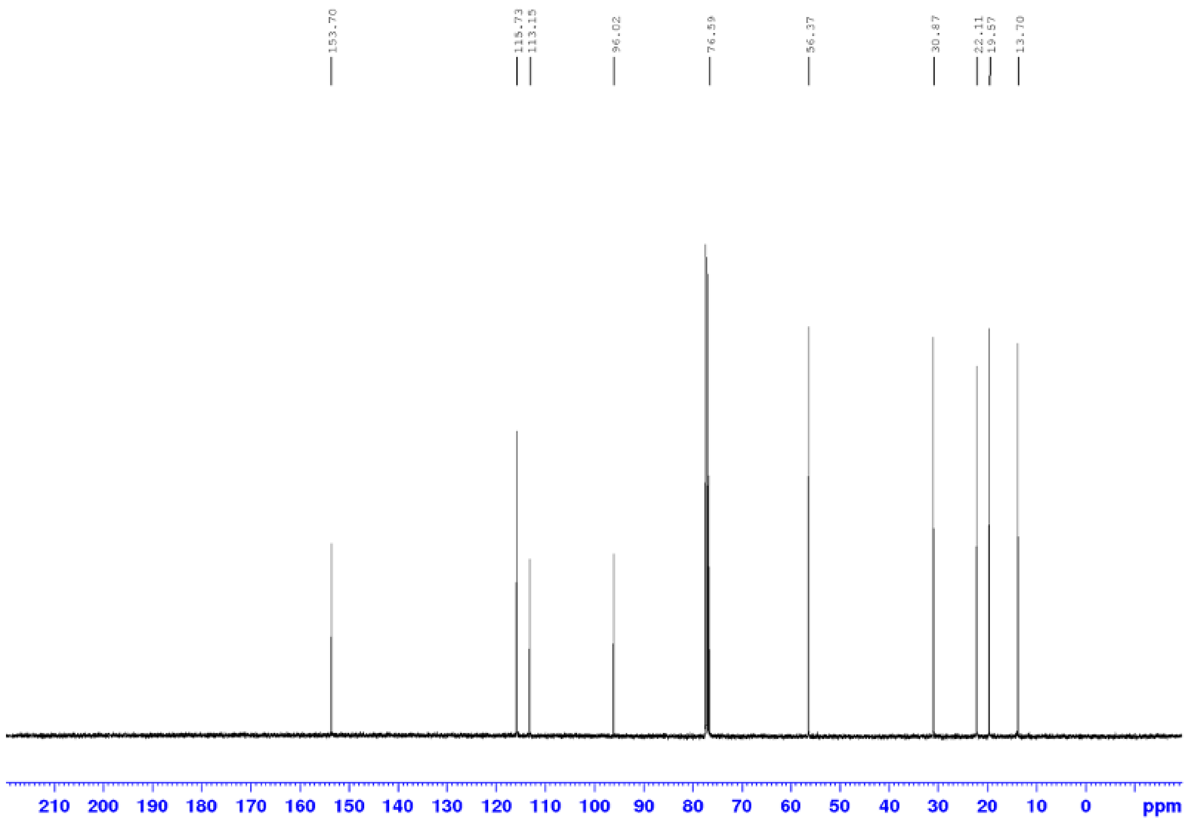

153.70
115.73
113.15
96.02
76.59
56.37
30.87
22.11
19.57
13.70
210 200 190 180 170 160 150 140 130 120 110 100 90 80 70 60 50 40 30 20 10 0 ppm

^{1}H-NMR of 10

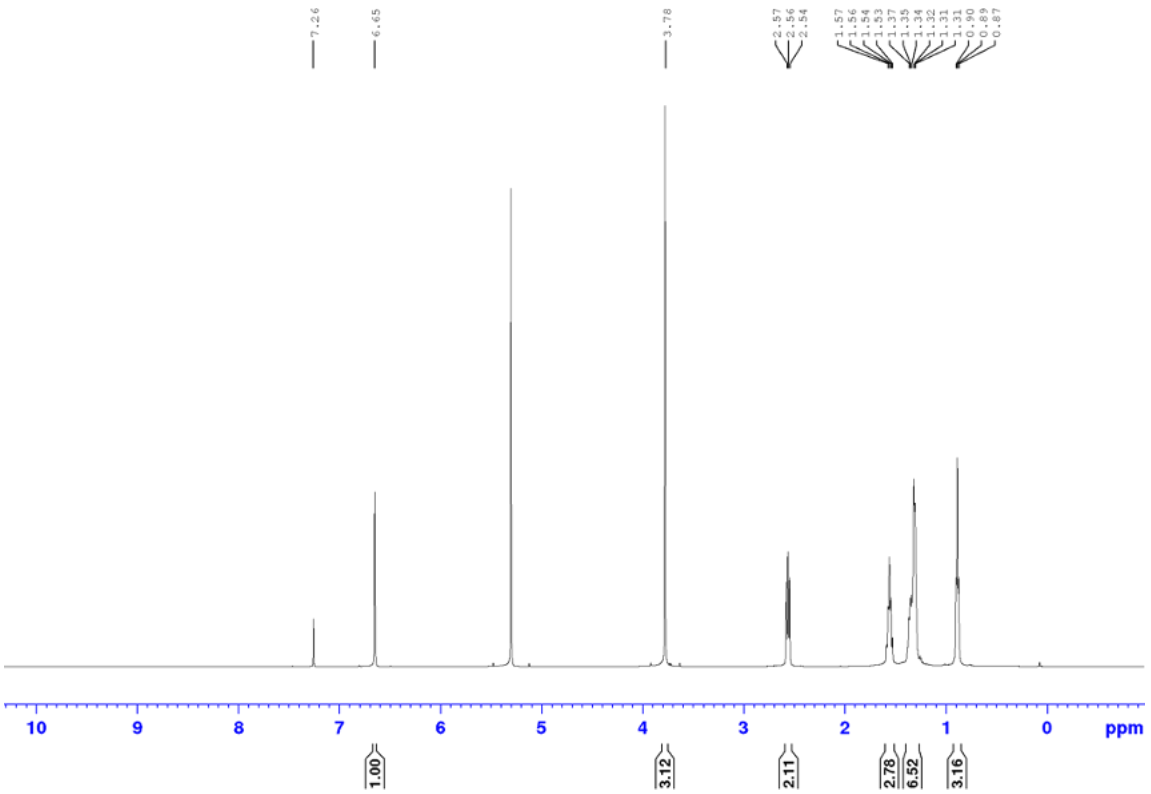
7.26
6.65
3.78
2.57
2.56
2.54
1.57
1.56
1.54
1.53
1.37
1.35
1.34
1.32
1.31
1.31
0.90
0.89
0.87
10
9
8
7
6
5
4
3
2
1
0
ppm
1.00
3.12
2.11
2.78
6.52
3.16

^{13}C-NMR of 10

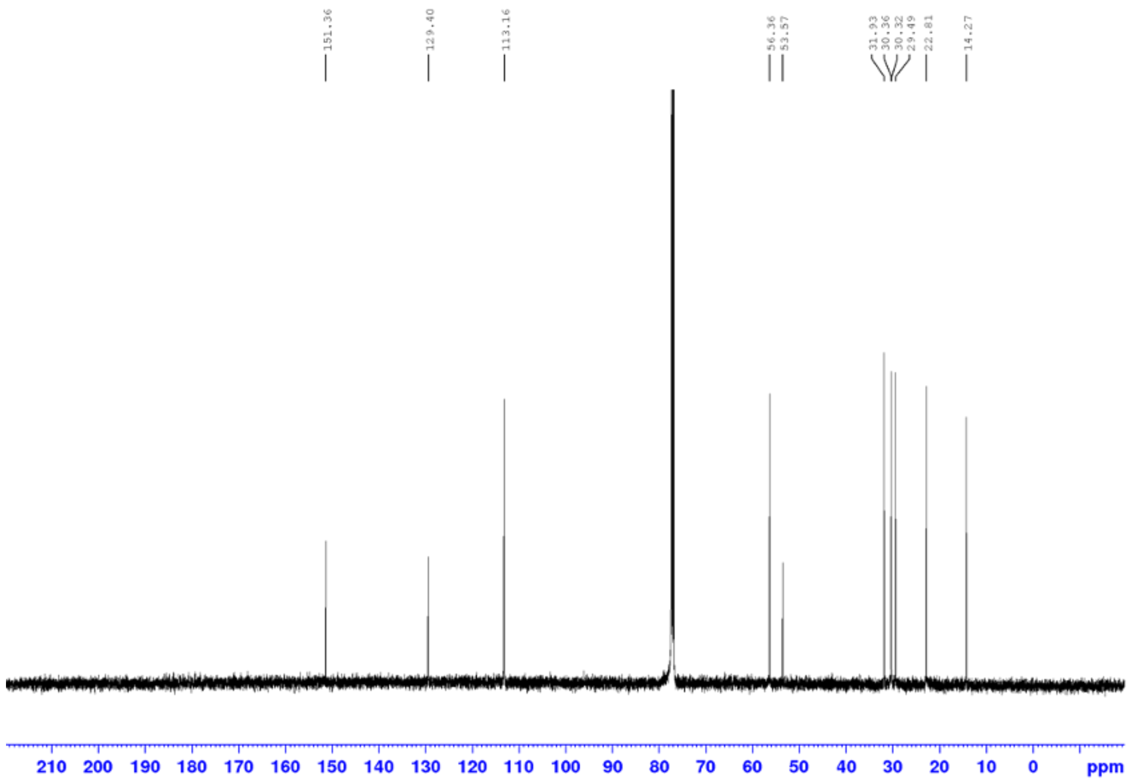
151.36
129.40
113.16
56.36
53.57
31.93
30.36
30.32
29.49
22.81
14.27
210 200 190 180 170 160 150 140 130 120 110 100 90 80 70 60 50 40 30 20 10 0 ppm

^{1}H-NMR of 11

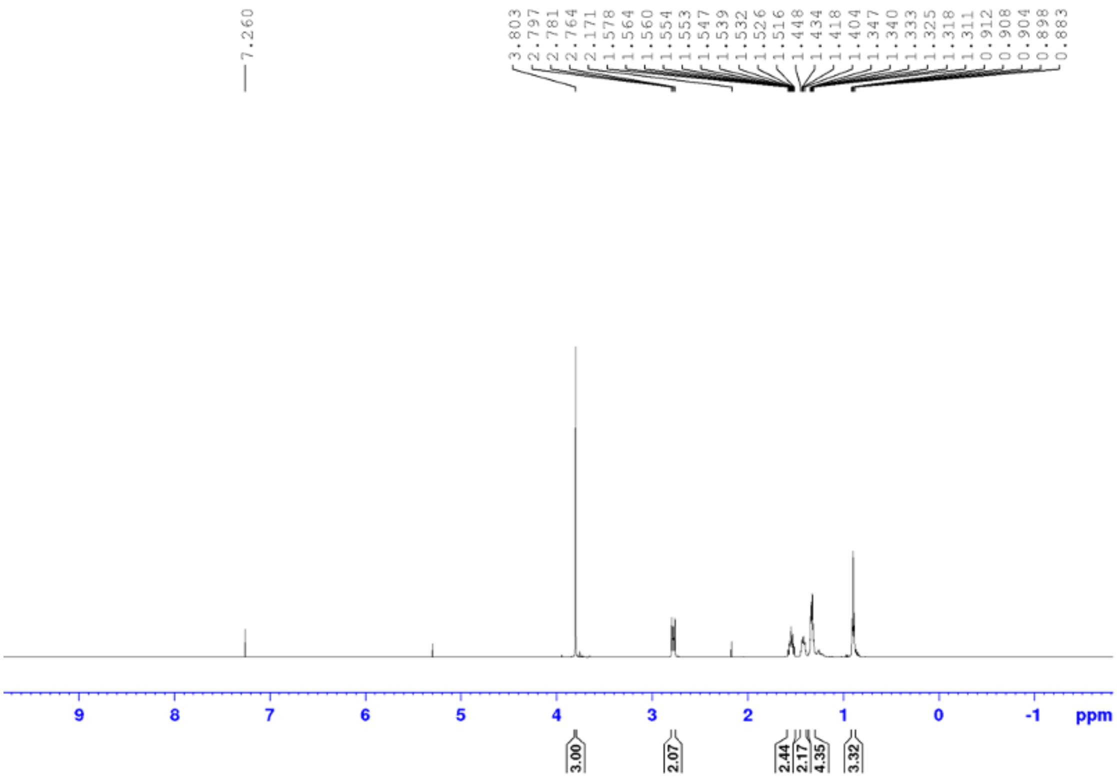
7.260
3.803
2.797
2.781
2.764
2.171
1.578
1.564
1.560
1.554
1.553
1.547
1.539
1.532
1.526
1.516
1.448
1.434
1.418
1.404
1.347
1.340
1.333
1.325
1.318
1.311
0.912
0.908
0.904
0.898
0.883
9
8
7
6
5
4
3
2
1
0
-1
ppm
3.00
2.07
2.44
2.17
4.35
3.32

^{13}C-NMR of 11

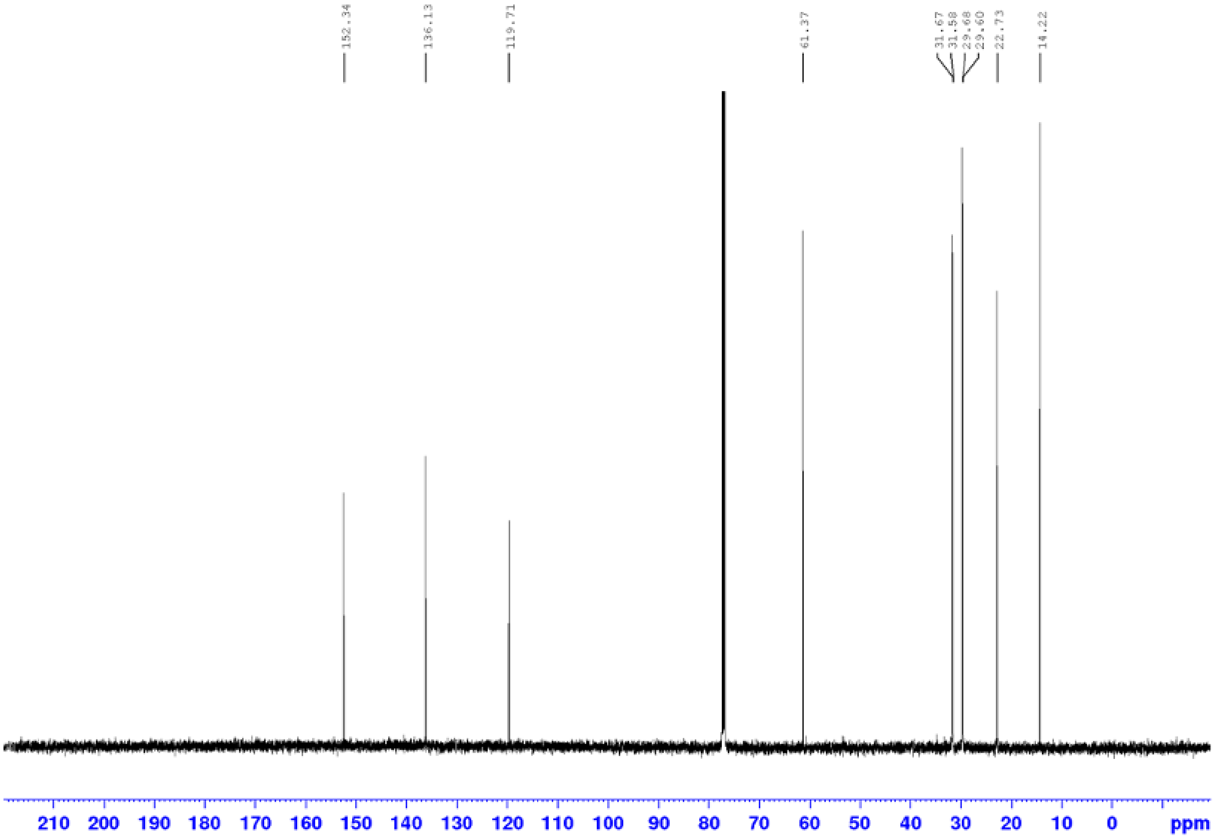
152.34
136.13
119.71
61.37
31.67
31.58
29.68
29.60
22.73
14.22
210 200 190 180 170 160 150 140 130 120 110 100 90 80 70 60 50 40 30 20 10 0 ppm

^{1}H-NMR of 12

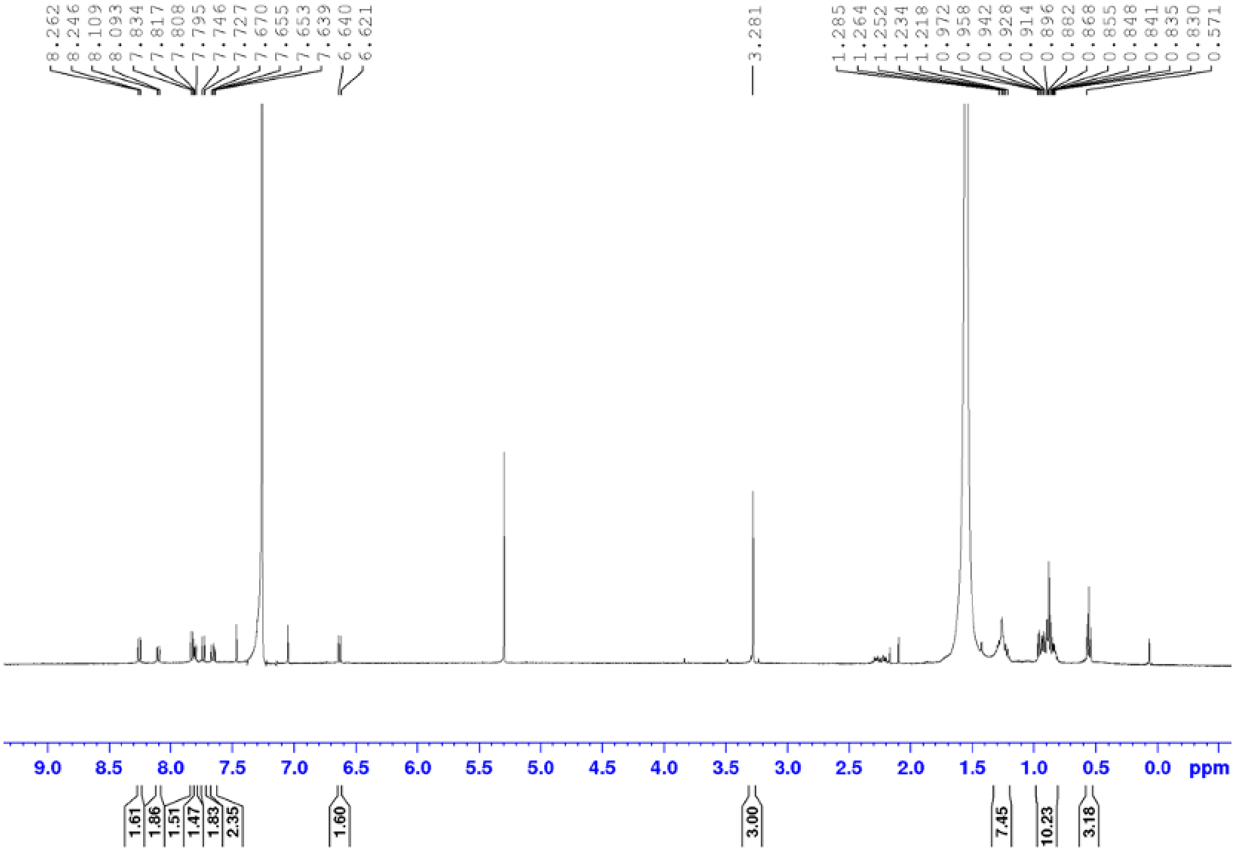
8.262
8.246
8.109
8.093
7.834
7.817
7.808
7.795
7.746
7.727
7.670
7.655
7.653
7.639
6.640
6.621
3.281
1.285
1.264
1.252
1.234
1.218
0.972
0.958
0.942
0.928
0.914
0.896
0.882
0.868
0.855
0.848
0.841
0.835
0.830
0.571
9.0 8.5 8.0 7.5 7.0 6.5 6.0 5.5 5.0 4.5 4.0 3.5 3.0 2.5 2.0 1.5 1.0 0.5 0.0 ppm
1.61
1.86
1.51
1.47
1.83
2.35
1.60
3.00
7.45
10.23
3.18

^{13}C-NMR of 12

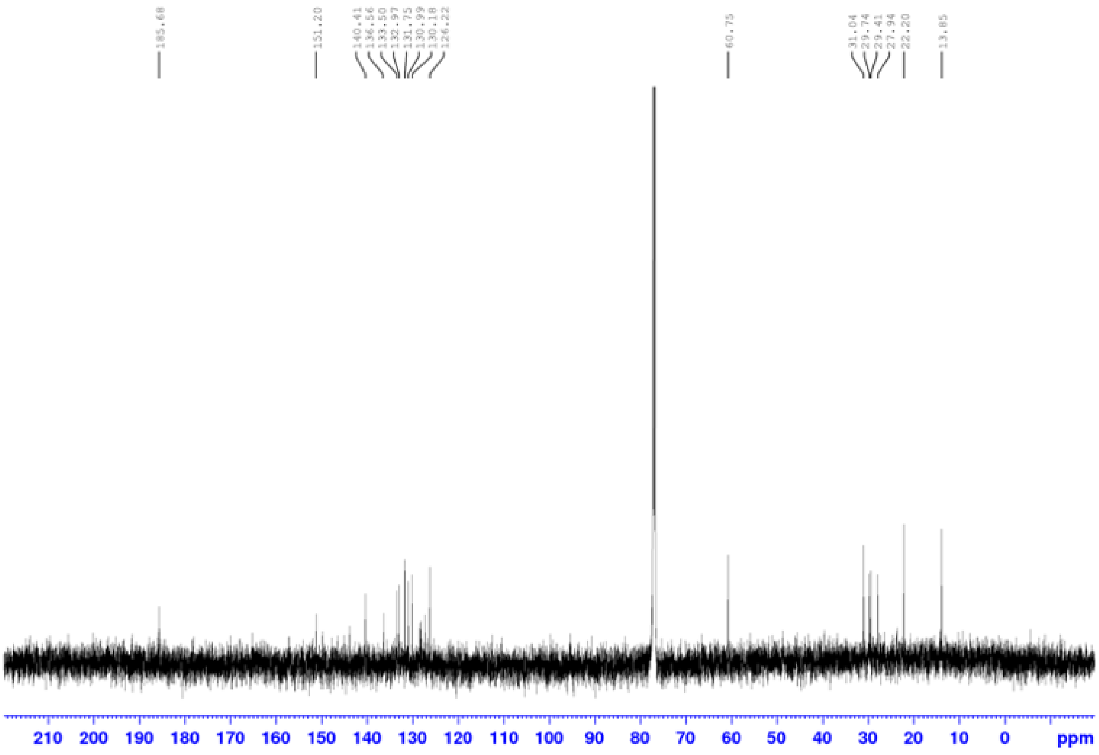
185.68
151.20
140.41
136.56
133.50
132.97
131.75
130.99
130.18
126.22
60.75
31.04
29.74
29.41
27.94
22.20
13.85
210 200 190 180 170 160 150 140 130 120 110 100 90 80 70 60 50 40 30 20 10 0 ppm

^{1}H-NMR of 4[Hexyl]

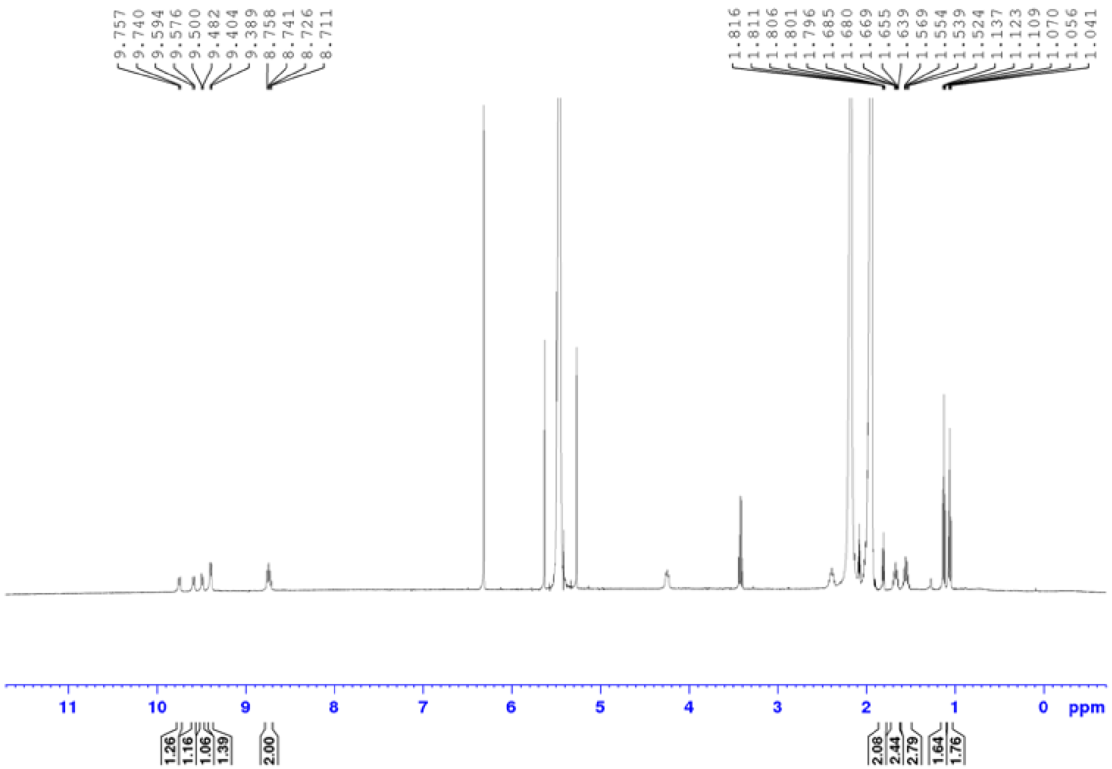

^{13}C-NMR of 4[Hexyl]

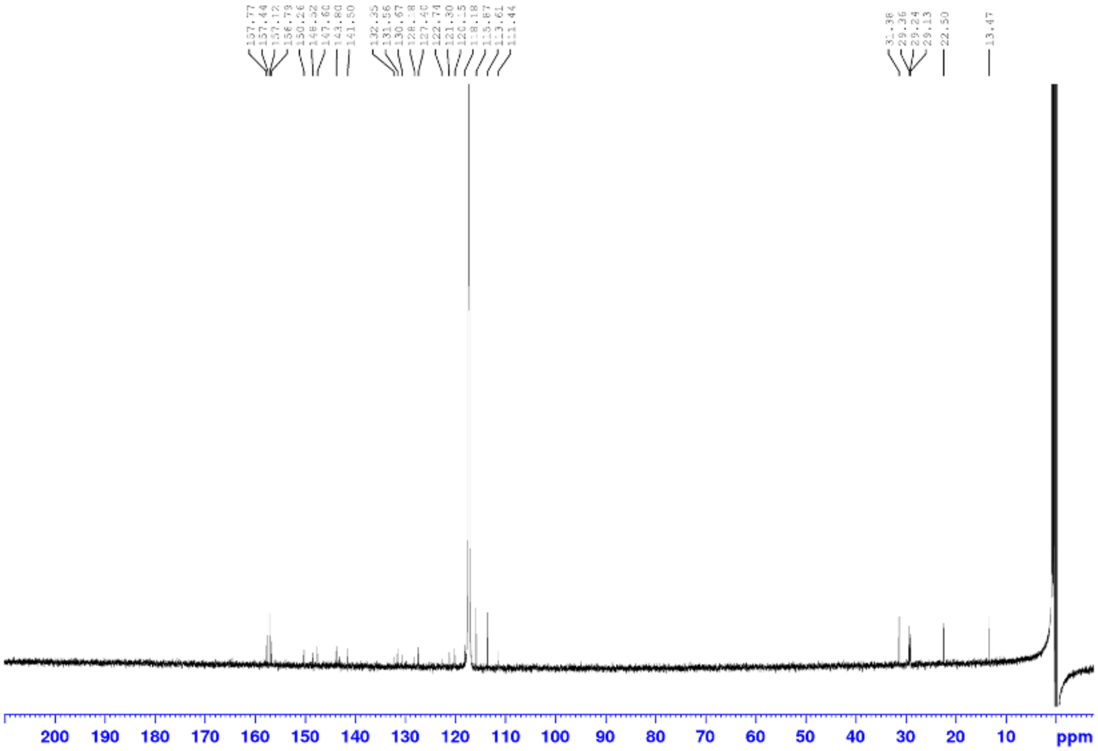

Steady State Spectroscopy

UV-Vis-NIR spectral data were measured at room temperature (298 K) with a Varian Cary 5000 spectrophotometer. All samples were collected in a 2 mm × 2 mm cuvette. All solvents used for solution samples were dried and degassed prior to use. Wavelengths are shown in nanometers (nm), and absorption in arbitrary units (a. u.). Fluorescence data were measured at room temperature (298 K) with an ISS Chronos BH spectrofluorometer. Steady-state spectra were collected with an excitation slit width of 1 mm and an emission slit width of 0.5 mm. Wavelengths are shown in nanometers (nm), and fluorescence is reported in arbitrary units (arb. units). Lifetime measurements were determined using the time-correlated single photon counting (TCSPC) technique, where a nanoLED laser (470 nm) was used as an excitation source.

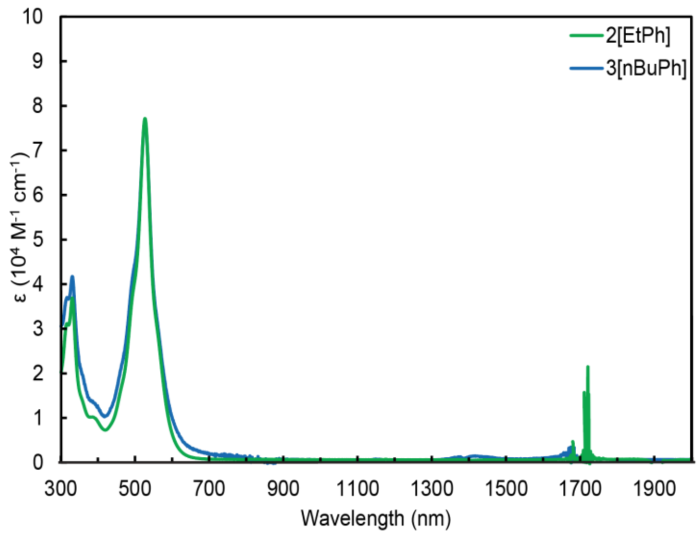

Figure S1: Full UV-vis Near-IR spectra of 2[EtPh] (solid green line), 3[nBuPh] (solid blue line) from 300nm-2000nm range.

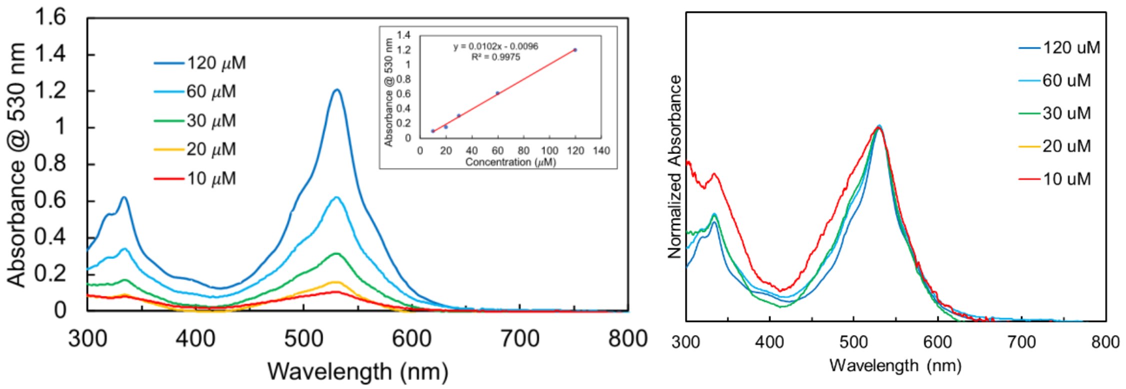

Figure S2: (left) Concentration dependent absorption spectra of 2[EtPh] and (right) normalized absorbance at λ_{max} = 520 nm (c = 10-120 μM).

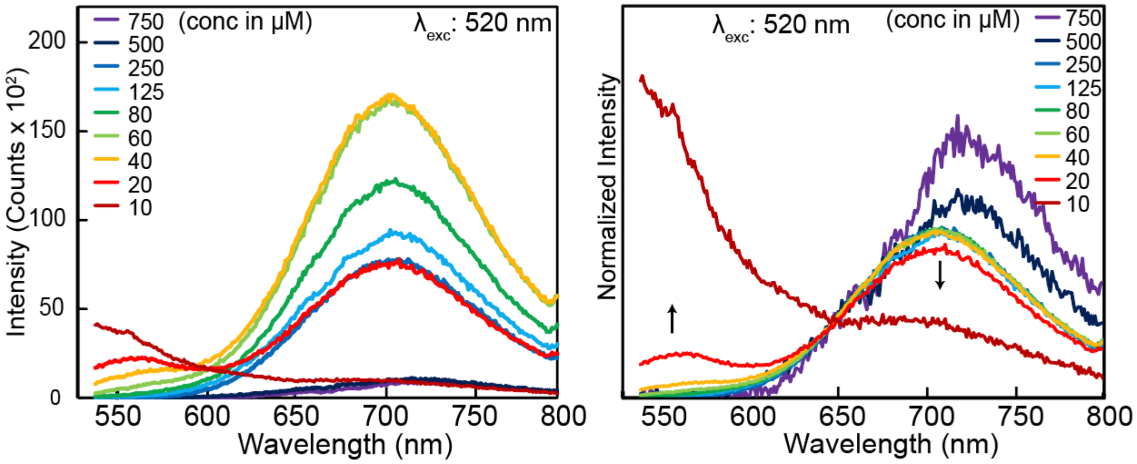

Figure S3: (left) Concentration dependent PL spectra of 2[EtPh] and (right) normalized PL spectra in acetonitrile.

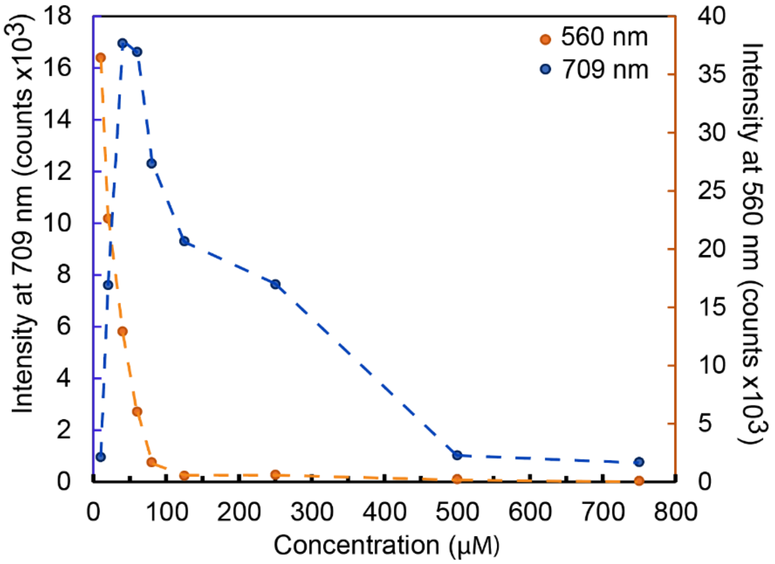

Figure S4: Intensity at observed emission wavelength for monomer (λ_{em} = 560 nm) and excimer (λ_{em} = 709 nm) at various concentrations (10-750 μM) for 2[EtPh].

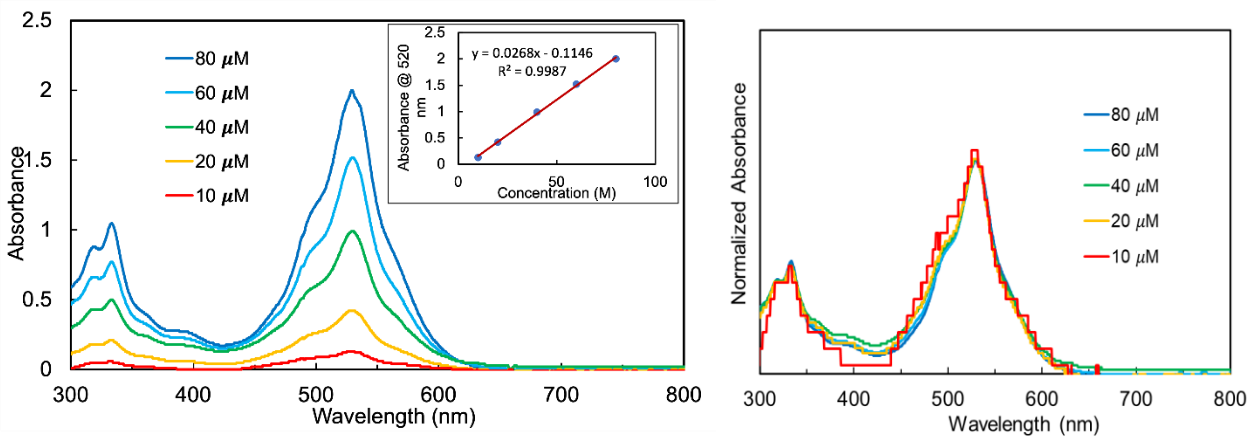

Figure S5: (left) Concentration dependent absorption spectra of 3[nBuPh] and (right) normalized absorbance at λ_{max} = 520 nm (c = 10-80 μM).

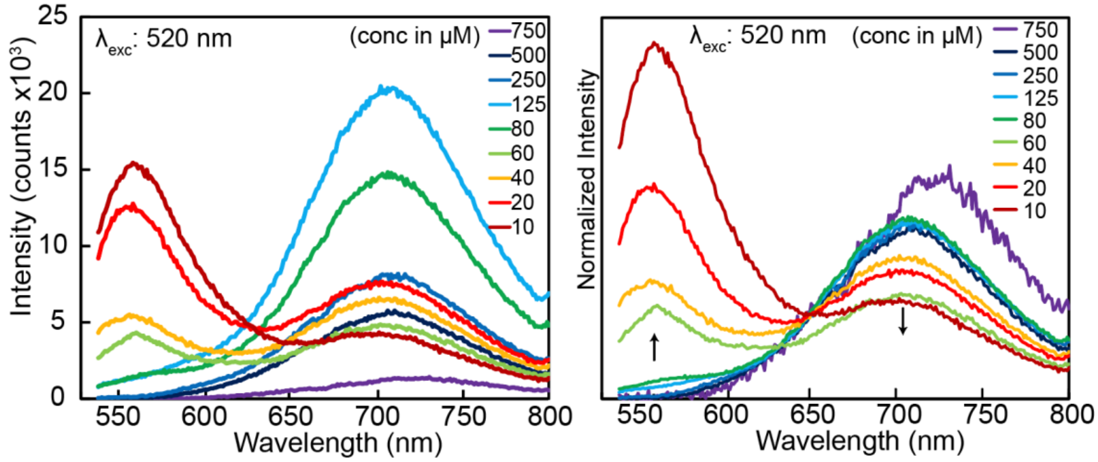

Figure S6: (left) Concentration dependent PL spectra of 3[nBuPh] and (right) normalized PL spectra in acetonitrile.

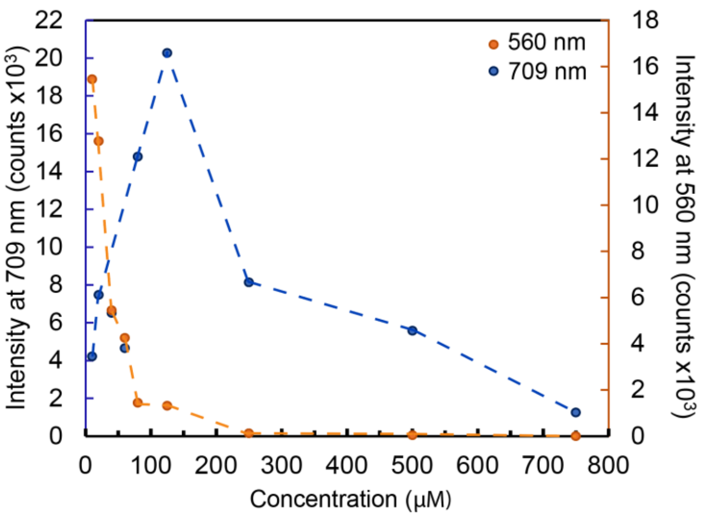

Figure S7: Intensity at observed emission wavelength for monomer (λ_{em} = 560 nm) and excimer (λ_{em} = 709 nm) at various concentrations (10-750 µM) for 3[nBuPh].

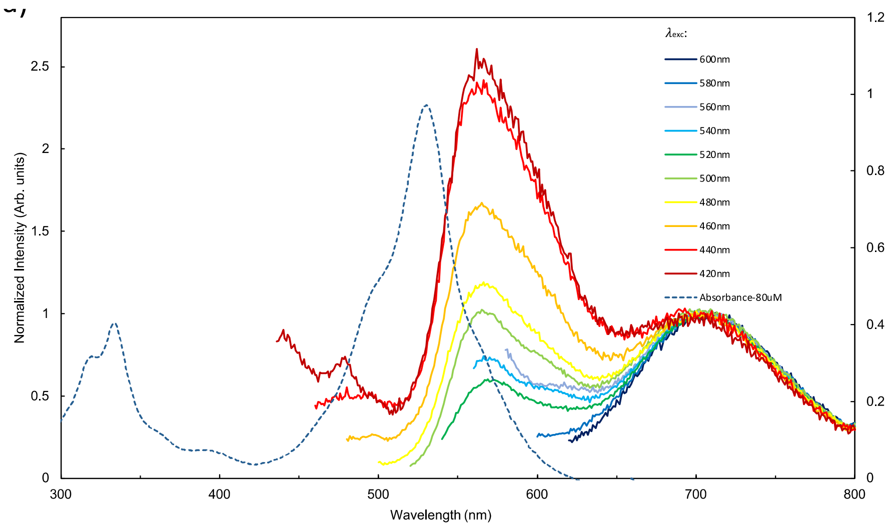

Figure S8: PL spectra of 3[nBuPh] at various λ_{ex} (420 nm – 600 nm, increments of 20 nm, c = 80 µM) and absorption spectra (black dash line) in acetonitrile.

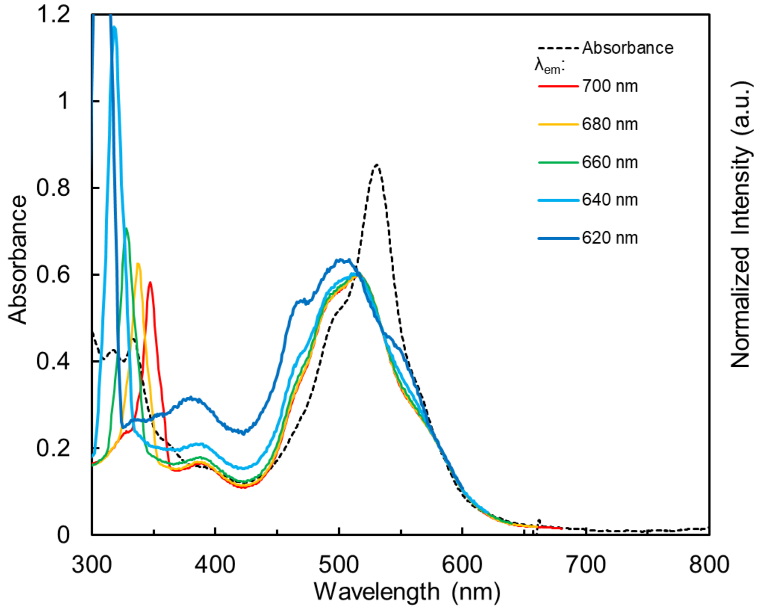

Figure S9: Emission excitation spectra of 3[nBuPh] at different λ_{em} (620 nm – 700 nm, increments of 20 nm, c = 80 µM) and absorption spectra (black dash line) in acetonitrile.

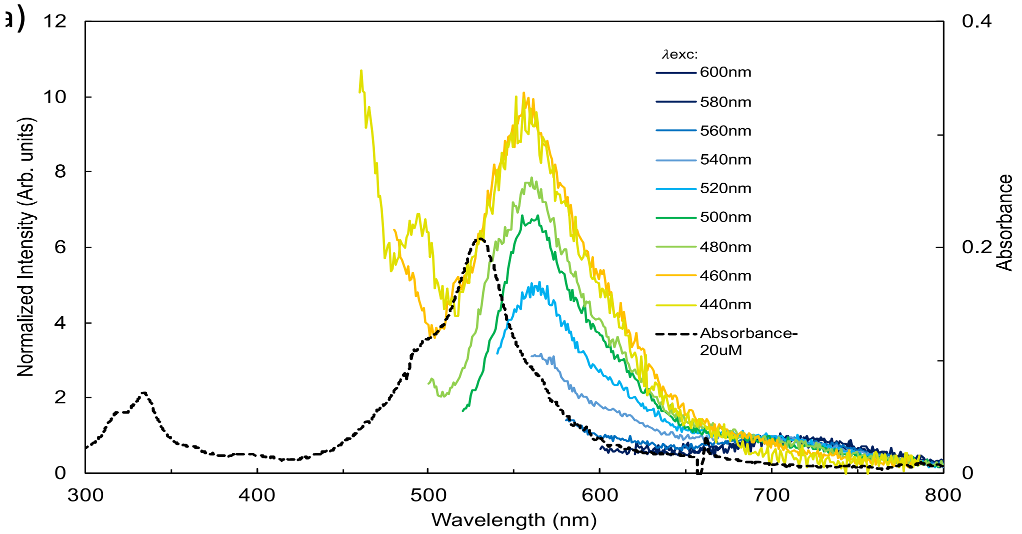

Figure S10: PL spectra of 3[nBuPh] at various λ_{ex} (420 nm – 600 nm, increments of 20 nm, c = 20 µM) and absorption spectra (black dash line) in acetonitrile.

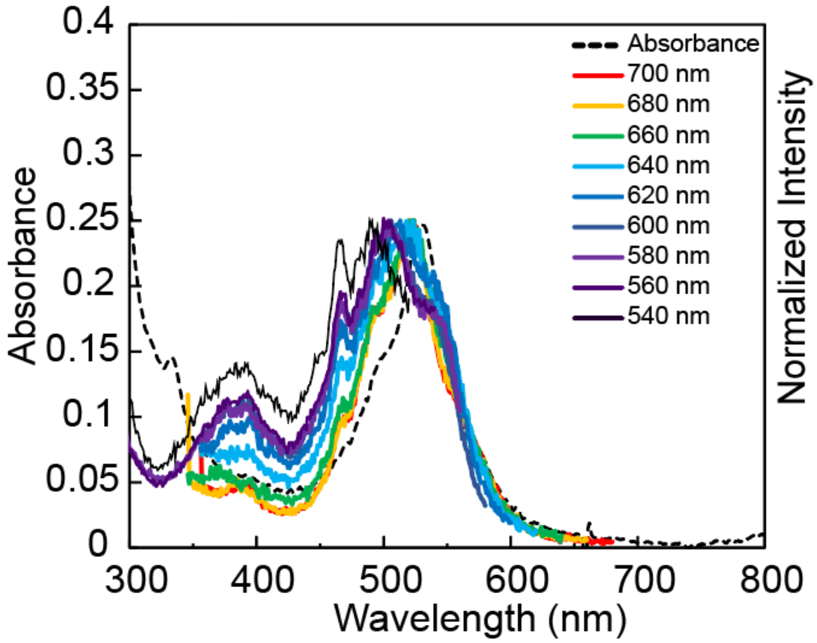

Figure S11: Emission excitation spectra of 3[nBuPh] at different λ_{em} (620 nm – 700 nm, increments of 20 nm, c = 20 µM) and absorption spectra (black dash line) in acetonitrile.

Table S1: Summary of dielectric constants and viscosity values for solvents

Solvent	Dielectric constant (ε)	Viscosity (cP)
1,1,2,2 tetrachloroethane	8.42	1.2
DCM	9.08	0.413
ACN	36.6	0.35
Dimethyl sulfoxide	46.6	1.99

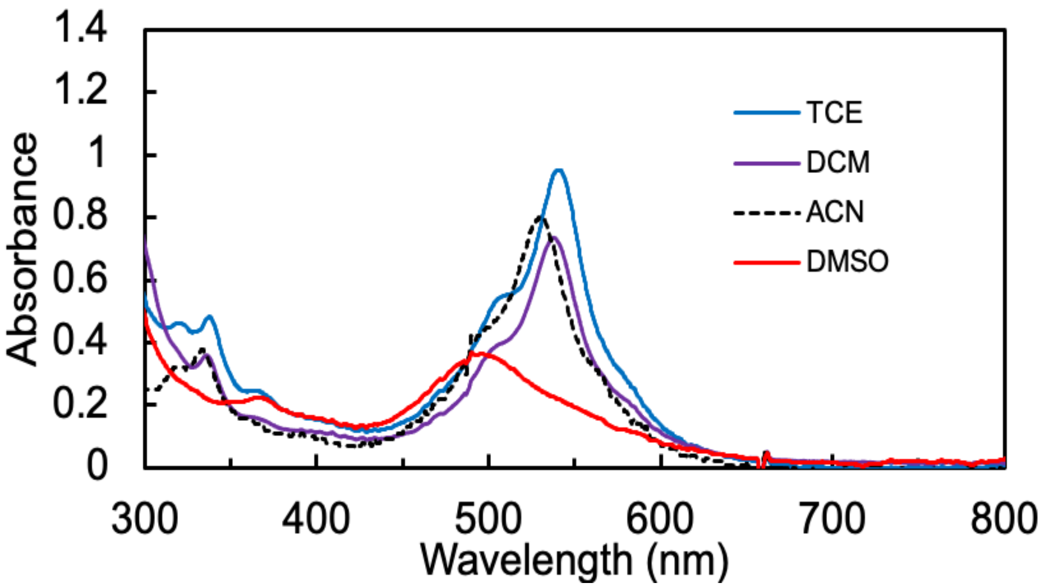

Figure S12: Solvent effect of 3[nBuPh]: absorption spectra of 3[nBuPh] in TCE (blue line, λ_{max} = 541 nm), DCM (purple line, λ_{max} = ~ 535 nm), ACN (black dashed line, λ_{max} = 523 nm), and DMSO (red line, λ_{max} = ~500 nm)

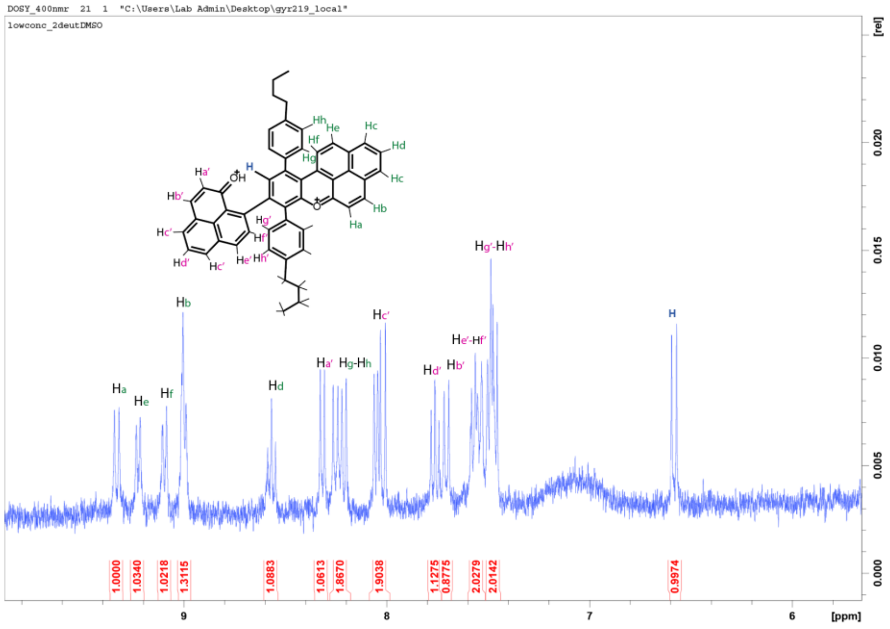

Figure S13: ^{1}H NMR of 3[nBuPh] in DMSO: ring opening of the π-backbone occurs in the presence of DMSO.

Environmental changes using viscous solvent and acid additives

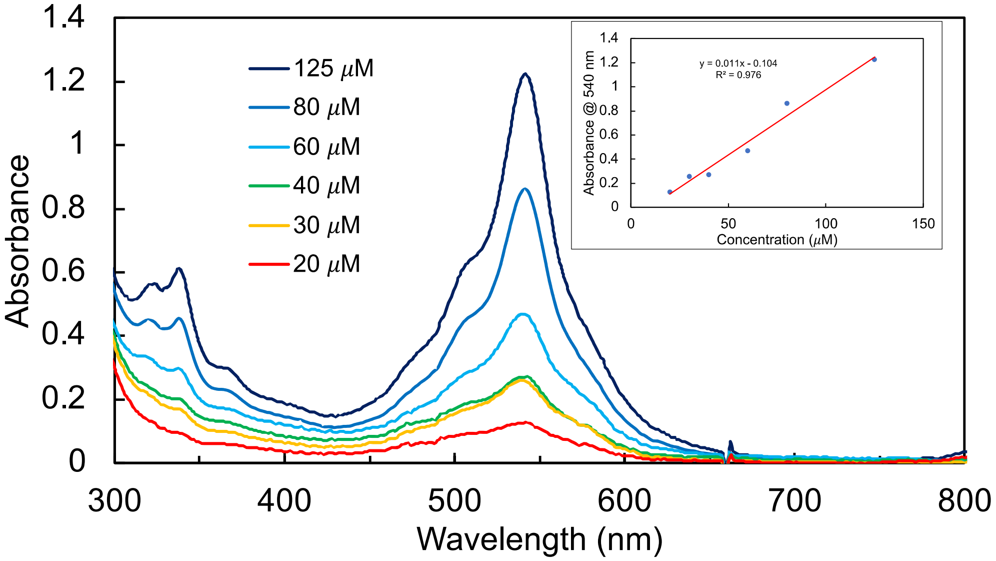

Figure S14: Concentration dependent absorption spectra of 2[EtPh] in TCE (c = 20-125 μM).

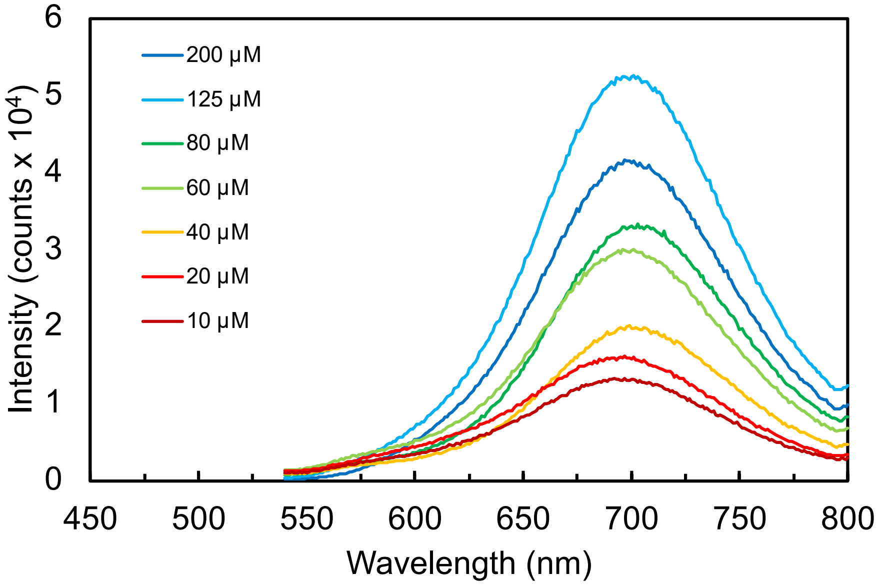

Figure S15: Concentration dependent PL spectra of 2[EtPh] (λ_{ex} = 520 nm, c = 10-200 μM) in TCE solvent.

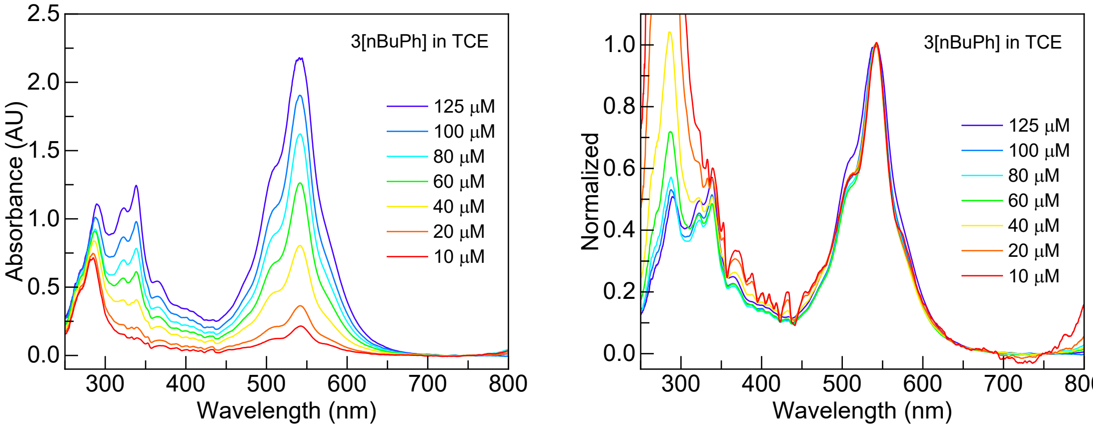

Figure S16: Concentration dependent absorption spectra of 3[nBuPh] in TCE (c = 10-125 μM).

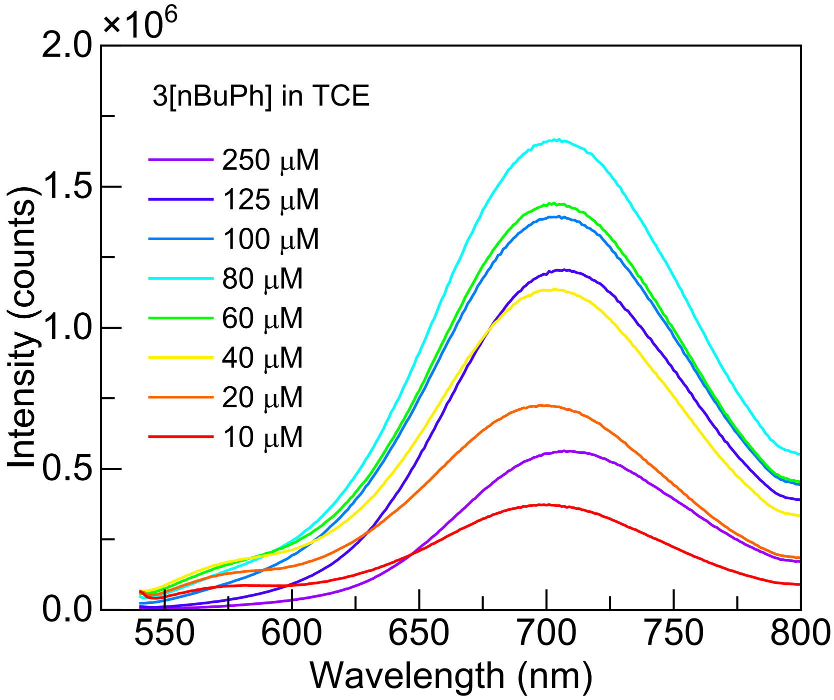

Figure S17: Concentration dependent PL spectra of 3[nBuPh] (λ_{ex} = 520 nm, c = 10-250 μM) in TCE solvent.

Acid conditions

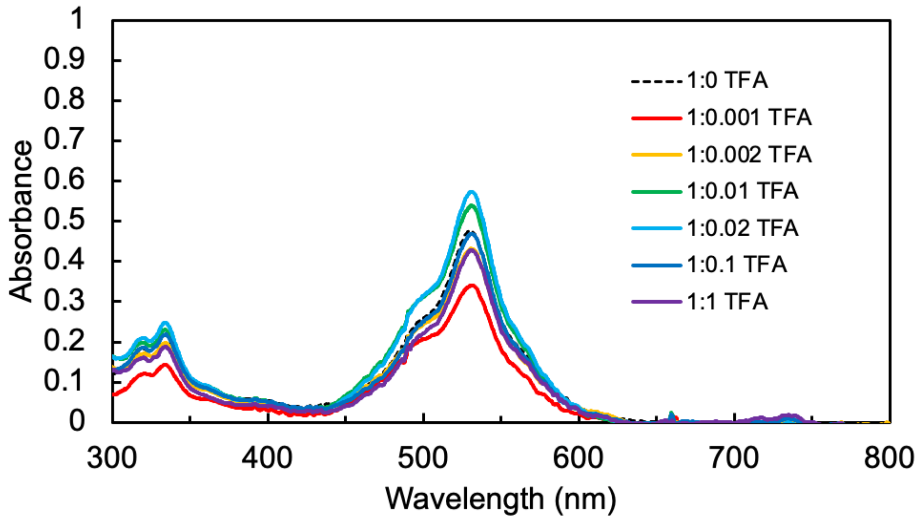

Figure S18: UV-vis absorption spectra of 3[nBuPh] in acetonitrile and upon addition of TFA at various molar ratio (3[nBuPh]:Acid, c = 40 μM).

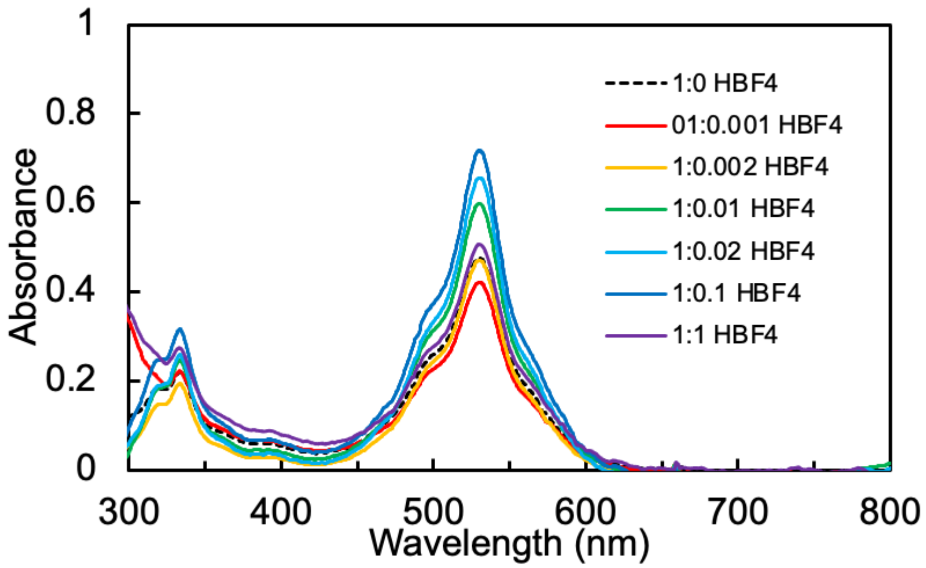

Figure S19: UV-vis absorption spectra of 3[nBuPh] in acetonitrile upon addition of HBF_4 at various molar ratio (3[nBuPh]:Acid, c = 40 μM).

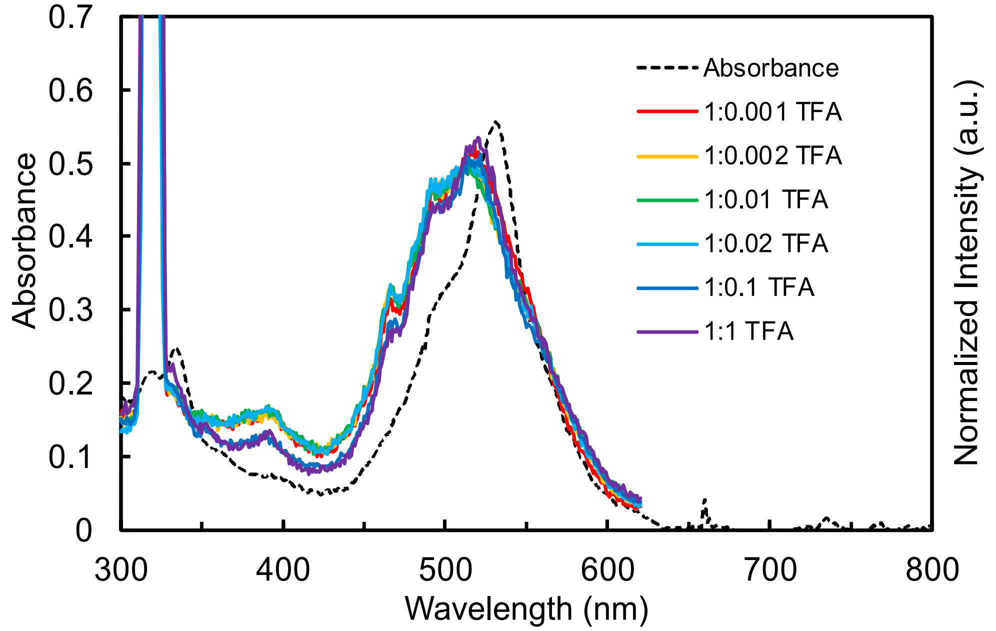

Figure S20: Excitation scan of 3[nBuPh] in acetonitrile and substoichiometric molar ratio of TFA overlapped with respective UV-vis spectra upon addition of TFA (λ_{obs} = 640nm, 0.1-100 mol%, c = 40 μM).

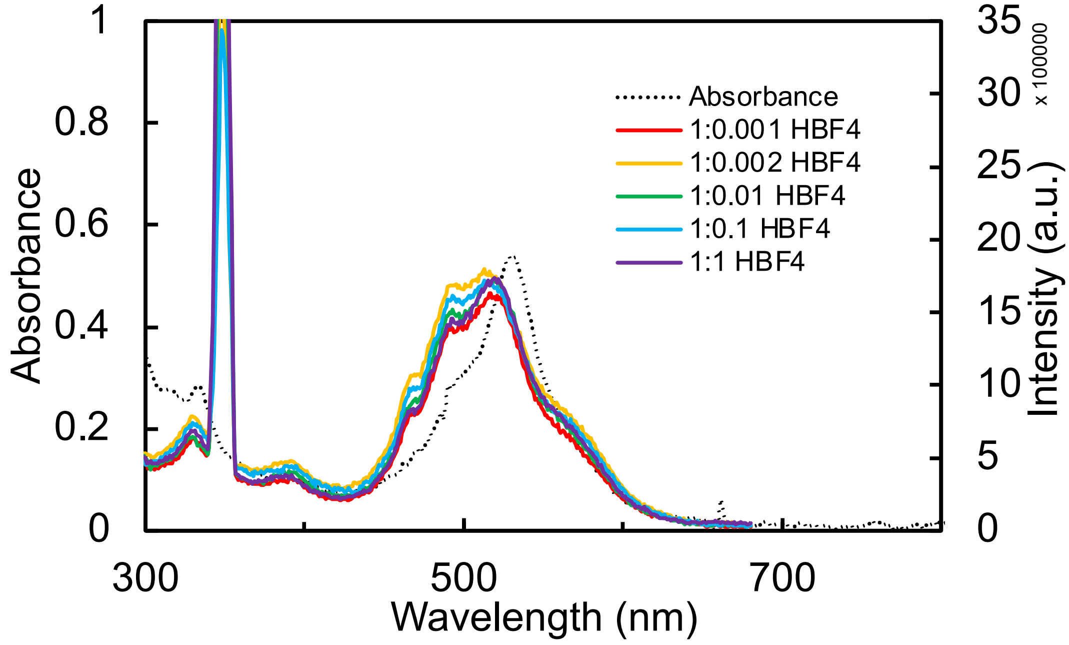

Figure S21: Excitation scan of 3[nBuPh] in acetonitrile and substoichiometric molar ratio of TFA overlapped with respective UV-vis spectra upon addition of HBF_4 (λ_{obs} = 700 nm, 0.1-100 mol%, c = 40 μM).

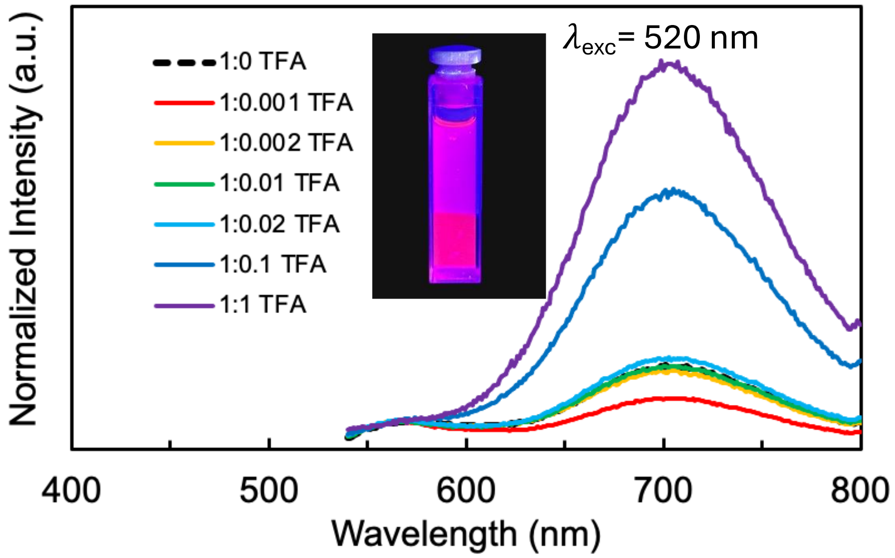

Figure S22: Emission spectra of 3[nBuPh] in acetonitrile (λ_{ex} = 520nm) at increasing concentration upon addition of TFA (0.1-100 mol%, c = 40 μM).

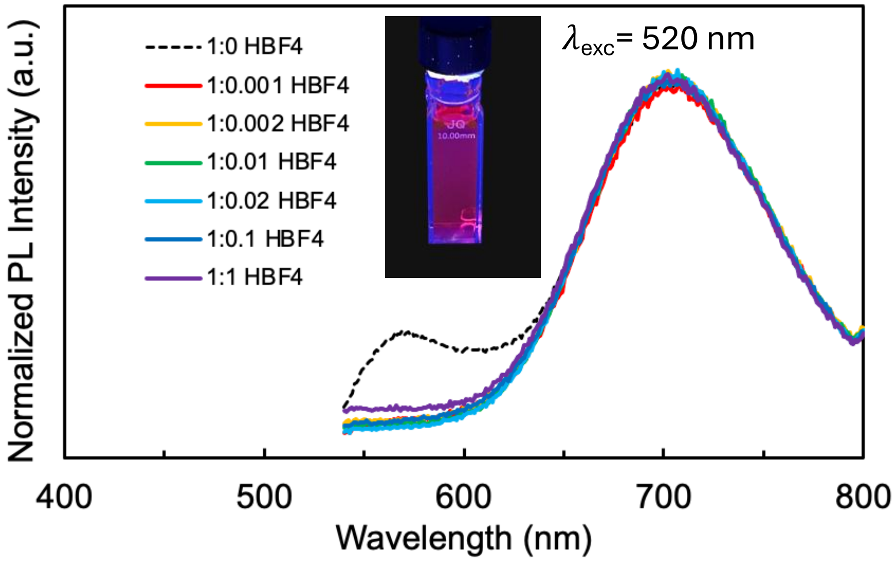

Figure S23: Emission spectra of 3[nBuPh] in acetonitrile (λ_{ex} = 520nm) upon addition of HBF_4 (0.1-100 mol%, c = 40 μM).

Photophysical properties of 4[Hexyl]:

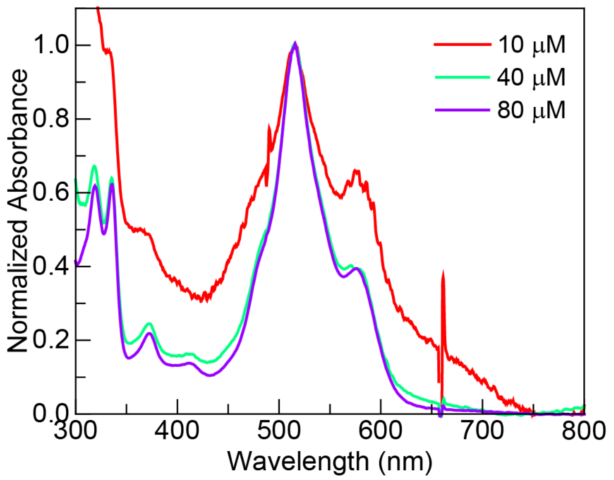

Figure S24: Normalized UV-vis absorption spectra of 4[Hexyl] in acetonitrile (red line = 10 μM, green = 40 μM, purple = 80 μM)

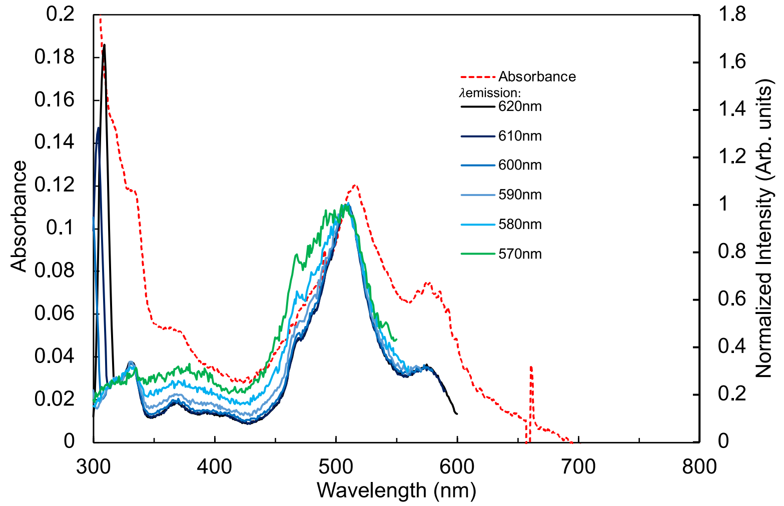

Figure S25: Absorption spectra (red dash line, c = 10 μM) and excitation spectra of 4[Hexyl] in acetonitrile at different λ_{em} (570 nm – 620 nm, increments of 10 nm).

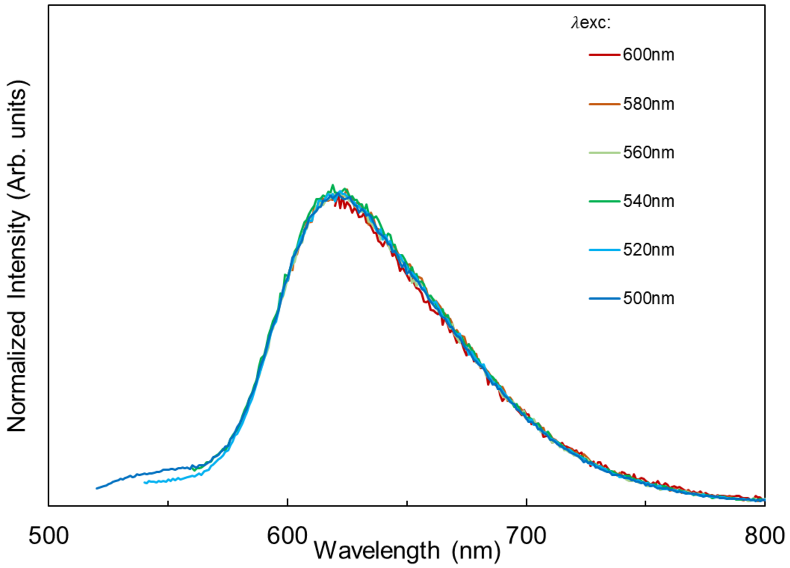

Figure S26: PL spectrum of 4[Hexyl] in acetonitrile: Monitoring emission behavior at various λ_{ex} wavelengths (500 nm – 600 nm, increments of 20 nm, c = 10 µM).

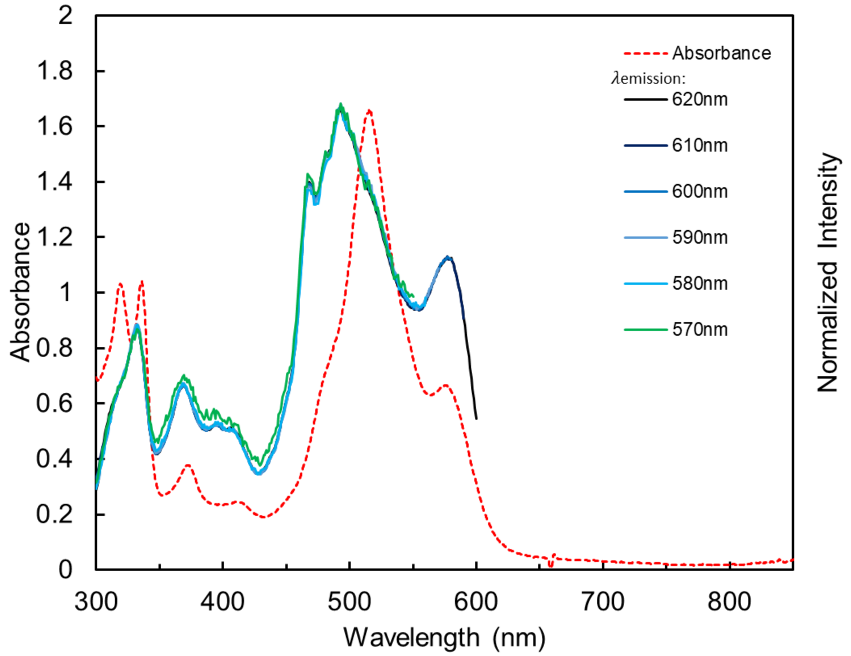

Figure S27: Absorption spectra (red dash line, c= 80 µM) and excitation spectra of 4[Hexyl] in acetonitrile at different λ_{em} (570 nm – 620 nm, increments of 10 nm).

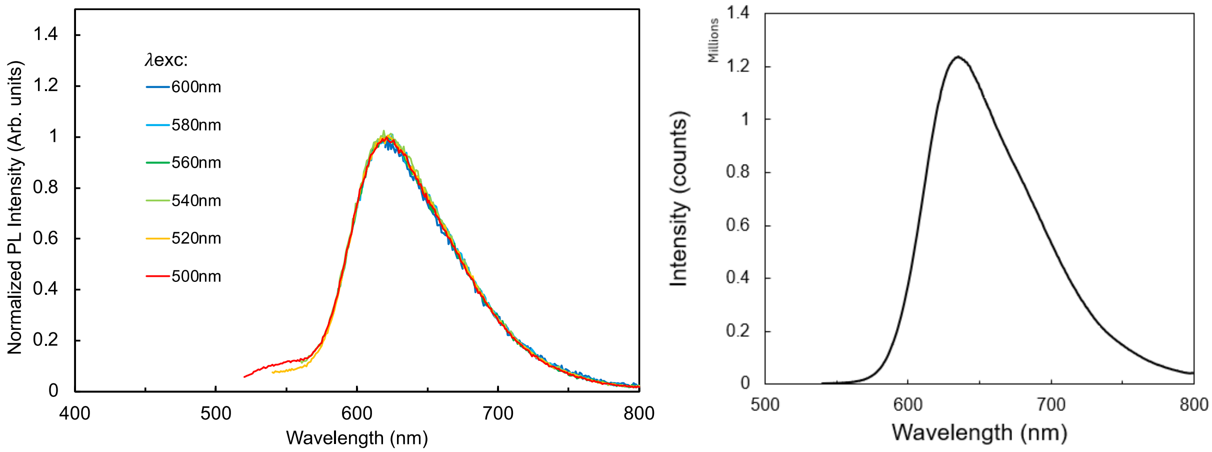

Figure S28: (Left) PL spectra of 4[Hexyl] in acetonitrile: Monitoring emission behavior at various λ_{ex} wavelengths (500 nm – 600 nm, increments of 20 nm, c = 80 µM). (Right) PL spectrum of 80 µM 4[Hexyl] in TCE at λ_{ex} = 520 nm.

Time-resolved Photoluminescence (trPL)

Lifetime measurements were determined using the time-correlated single photon counting (TCSPC) technique, where a nanoLED laser (470 nm) was used as an excitation source. For 2[Etph], a 495-600 nm bandpass filter was used to collect the monomer emission lifetime and a 700 nm longpass filter was used to collect the excimer emission lifetime. For 3[nBuPh], a 510 nm longpass and a 550 nm bandpass filter (FWHM = 40 nm) was used to collect the monomer emission lifetime and a 670 nm bandpass filter (FWHM = 10 nm) was used to collect the excimer emission lifetime. For 4[Hexyl], a 510 nm longpass filter was used to collect the emission lifetime. An IRF was collected in the absence of filters in order to deconvolute the emission lifetime data during fitting.

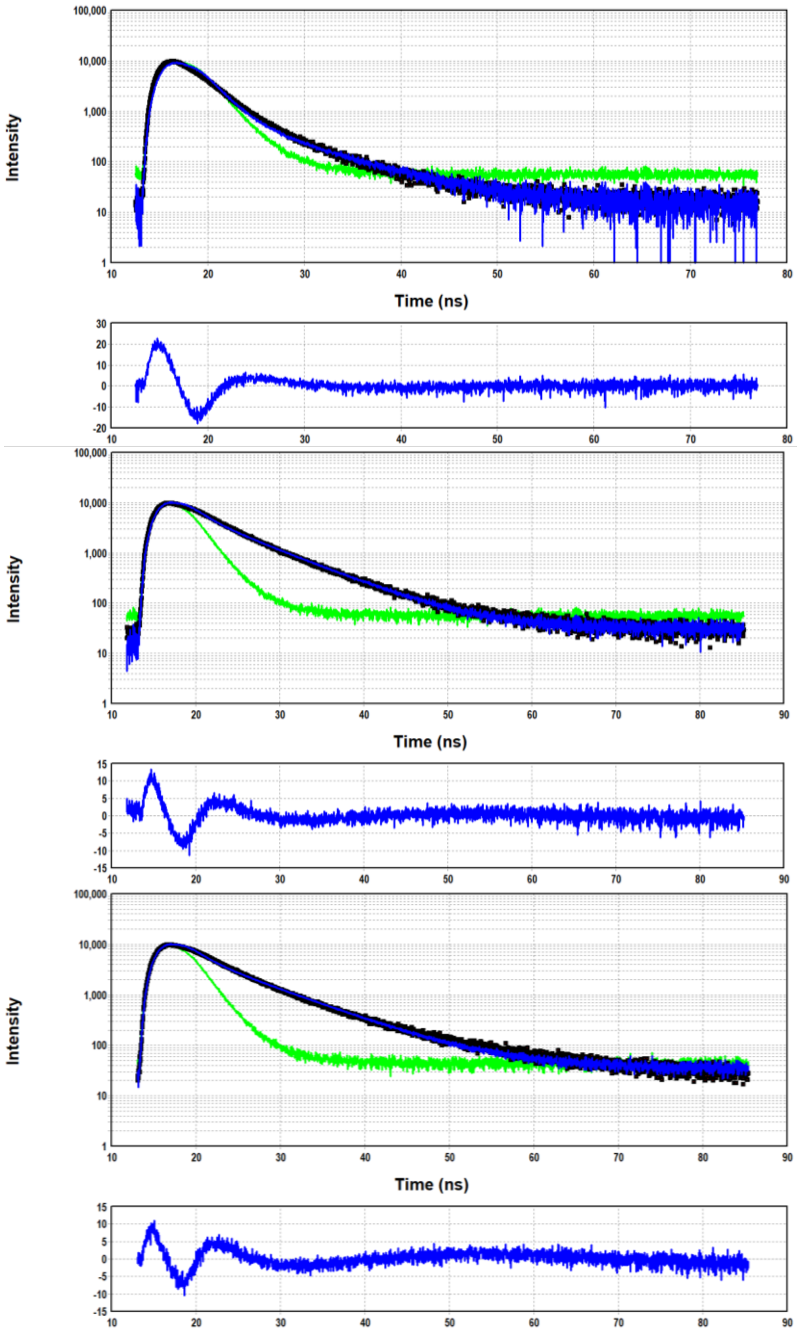

Figure S29: trPL of 2[EtPh] in acetonitrile collected at various concentrations (top to bottom: 10 µM, 40 µM, 80 µM) using a 495 – 600 nm bandpass filter to selectively collect monomer emission, shown with a fit to a monoexponential function. One trial shown to represent the three trials collected.

The fitting of 2[EtPh] emission data was performed using additional lifetimes. The use of additional lifetimes did not significantly improve the shape of the residuals from that displayed in Figure S31 and therefore a monoexponential fit was retained. The residual represents less ~0.3% of the overall signal.

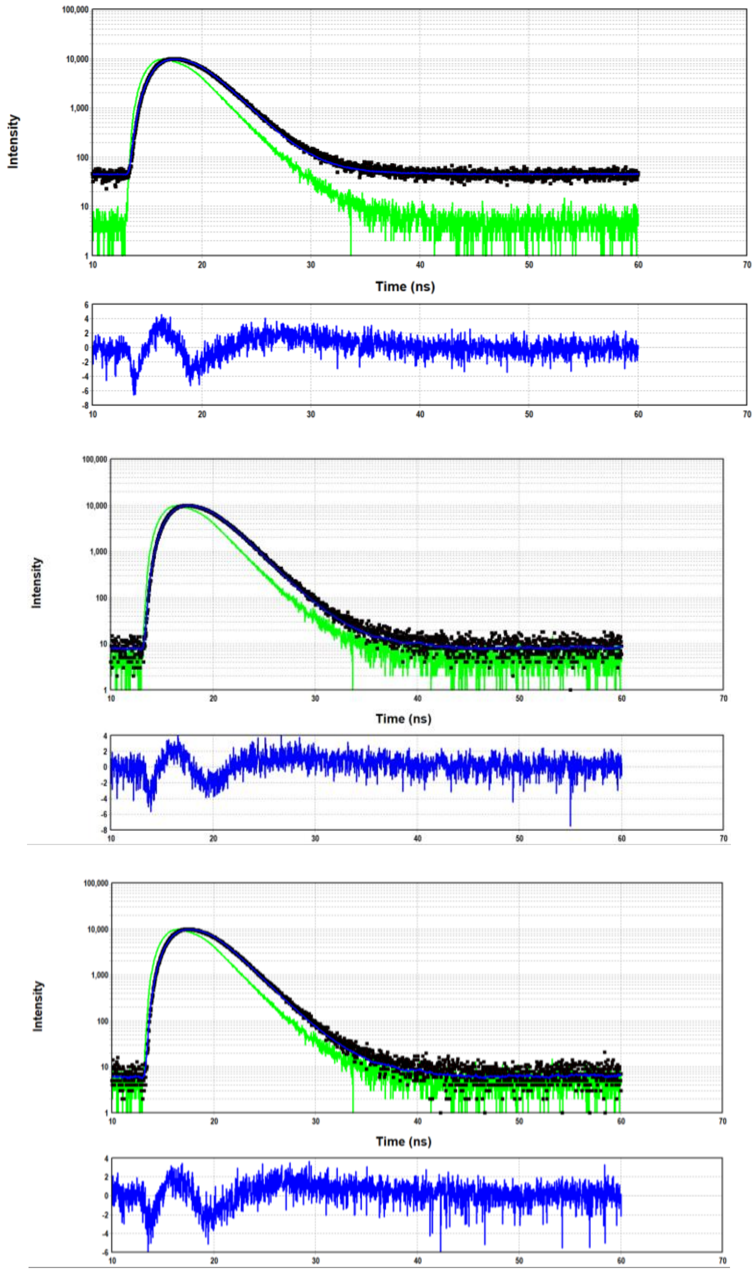

Figure S30: trPL of 2[EtPh] in acetonitrile collected at various concentrations (top to bottom: 10 μM, 40 μM, 80 μM) using a longpass filter of 700 nm to selectively collect excimer emission, shown with a fit to a monoexponential function. One trial shown to represent the three trials collected.

The fitting of 2[EtPh] emission data was performed using additional lifetimes. The use of additional lifetimes did not significantly improve the shape of the residuals from that displayed in Figure S32 and therefore a monoexponential fit was retained. The residual represents less ~0.3% of the overall signal.

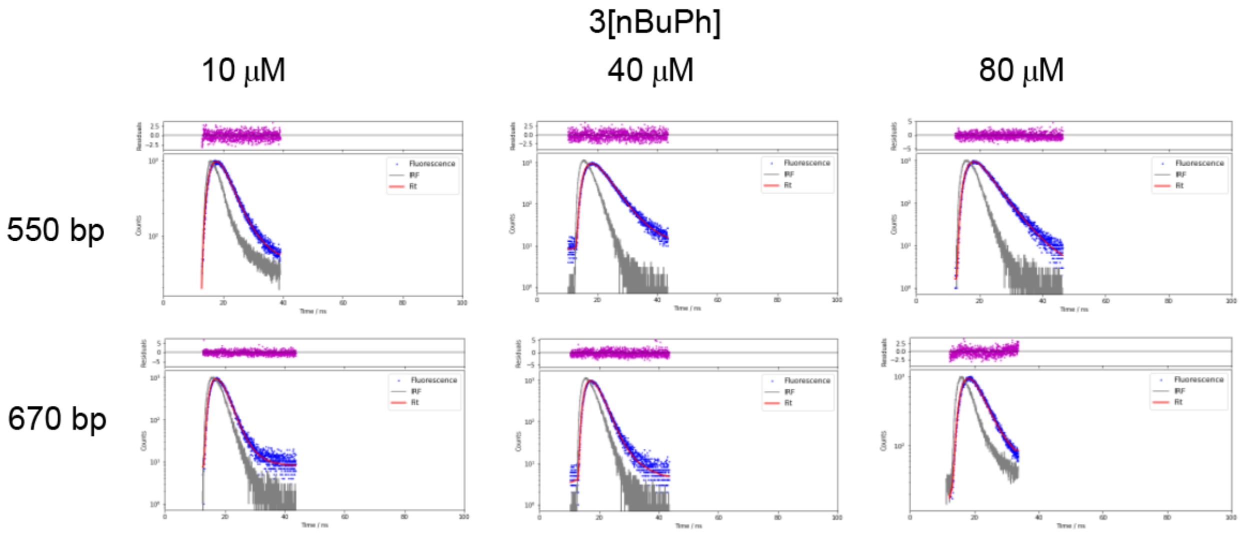

Figure S31: trPL of 3[nBuPh] in acetonitrile collected at various concentrations (10 µM, 40 µM, 80 µM) using a (top row) bandpass filter of 550 nm to selectively collect monomer emission and (bottom row) a bandpass filter of 670 nm to selectively collect excimer emission.

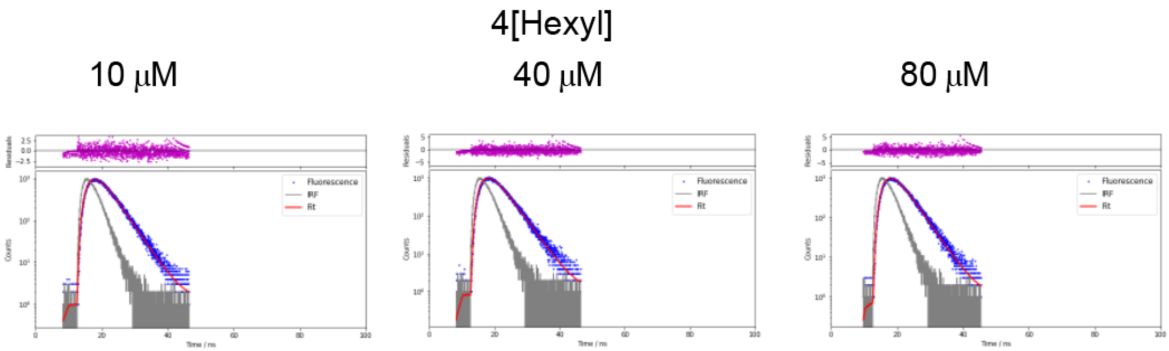

Figure S32: trPL of 4[Hexyl] in acetonitrile collected at various concentrations (10 µM, 40 µM, 80 µM).

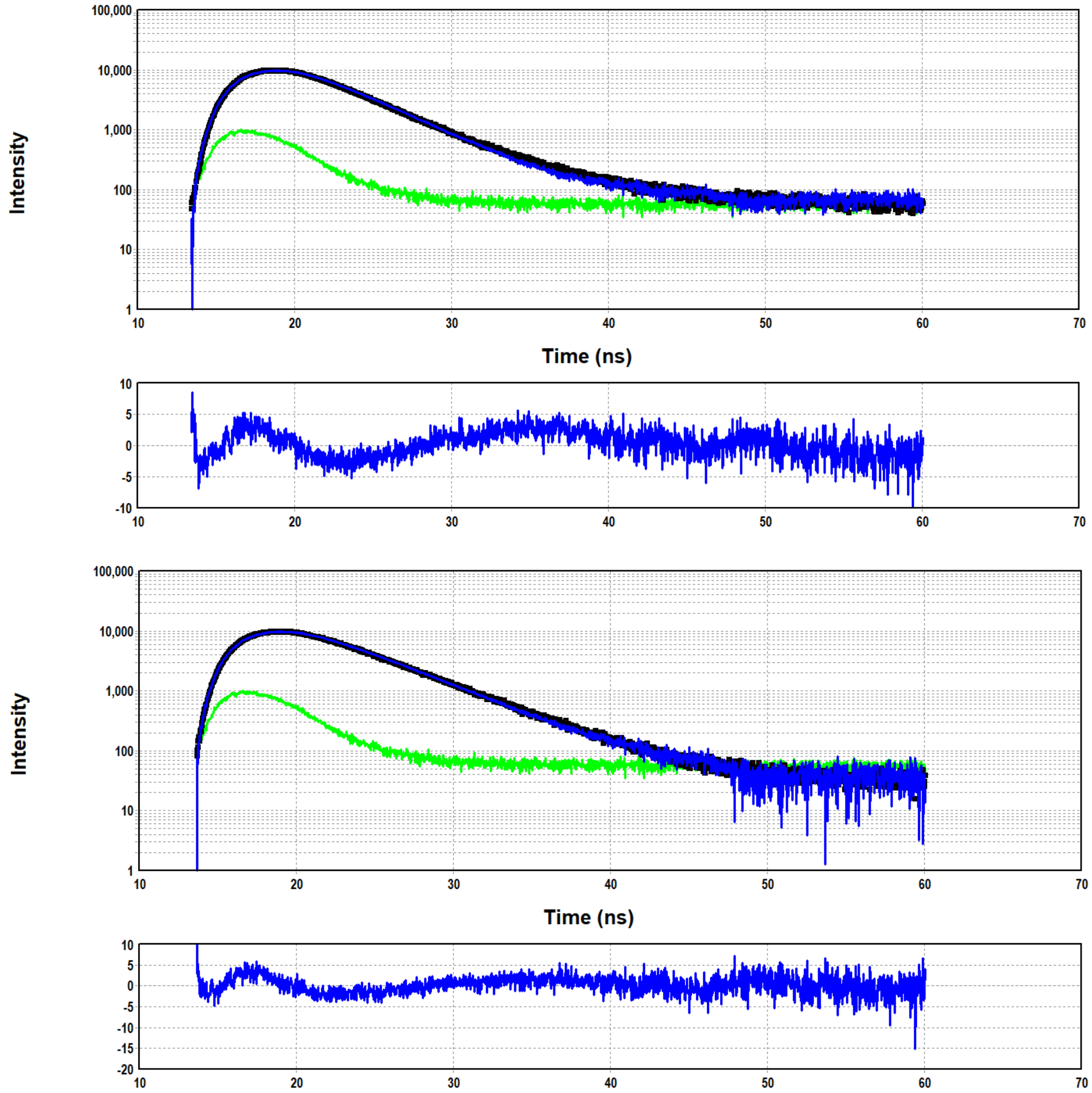

Figure S33: trPL of 80 µM 2[EtPh] in TCE using a (top row) bandpass filter of 550 nm to selectively collect monomer emission and (bottom row) a bandpass filter of 670 nm to selectively collect excimer emission.

The fitting of 2[EtPh]emission data was performed using additional lifetimes. The use of additional lifetimes did not significantly improve the shape of the residuals from that displayed in Figure S33 and therefore a monoexponential fit was retained. The residual represents less ~0.3% of the overall signal.

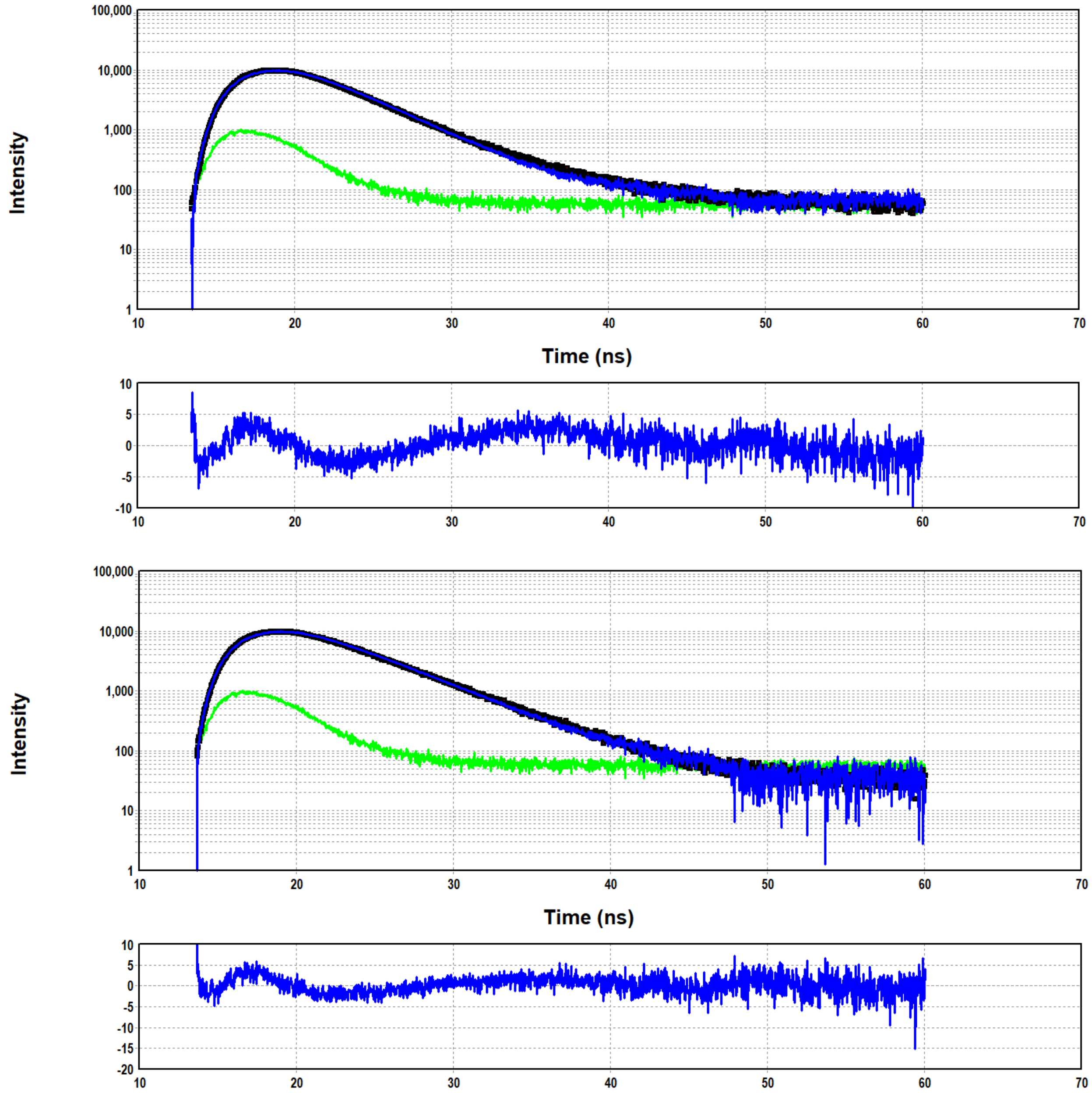

Figure S34: trPL of 80 µM 3[nBuPh] in TCE using a (top row) bandpass filter of 550 nm to selectively collect monomer emission and (bottom row) a bandpass filter of 670 nm to selectively collect excimer emission.

The fitting of 3[nBuPh] emission data was performed using additional lifetimes. The use of additional lifetimes did not significantly improve the shape of the residuals from that displayed in Figure S34 and therefore a monoexponential fit was retained. The residual represents less ~0.3% of the overall signal.

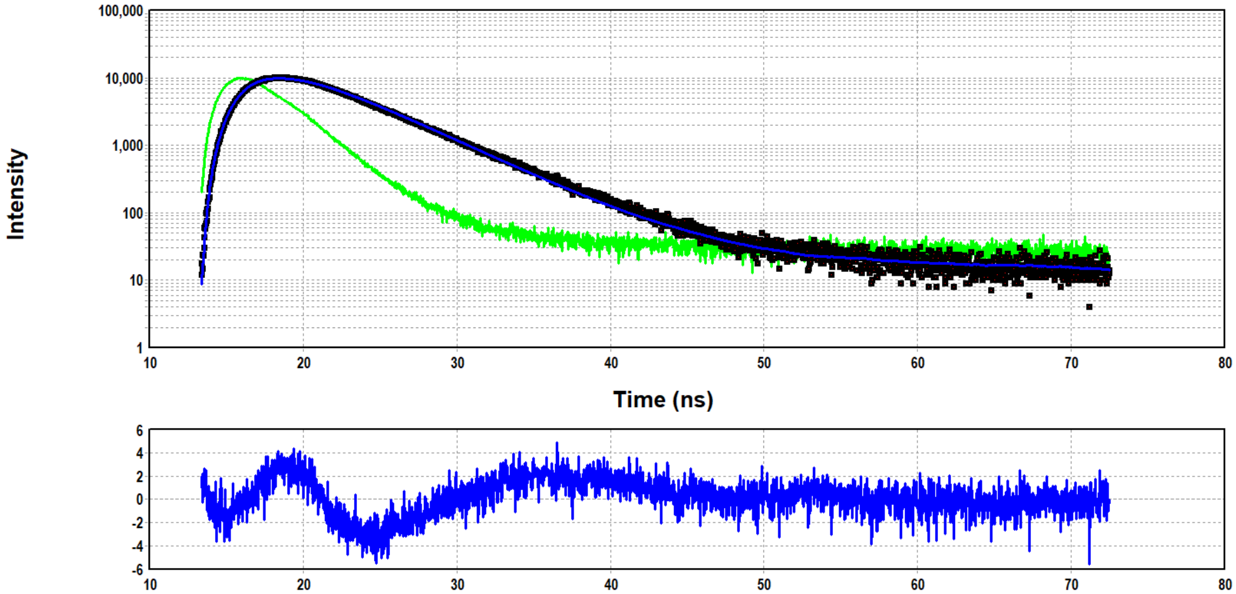

Figure S35: trPL of 80 µM 4[Hexyl] in TCE using a 510 nm bandpass filter.

Table S2: Comparison of photophysical properties of 80 µM 2[EtPh], 3[nBuPh], and 4[Hexyl] in ACN vs. TCE

	Solvent	Abs λ_{max} (nm)	PL λ_{mono} (nm)	PL λ_{excm} (nm)	PL τ_{mono} (ns)	PL τ_{excm} (ns)
**2**[EtPh]	ACN	523	560	709	6.73 ± 0.39	1.09 ± 0.01
	TCE	541		703	4.18 ± 0.24	3.52 ± 0.07
**3**[nBuPh]	ACN	523	560	709	2.81 ± 0.02	1.30 ± 0.01
	TCE	541		705	2.82 ± 0.02	3.53 ± 0.02
**4**[Hexyl]	ACN	515	627		3.41 ± 0.04	
	TCE	528	635		3.73 ± 0.05	

Summary of monomeric photophysical properties in acetonitrile

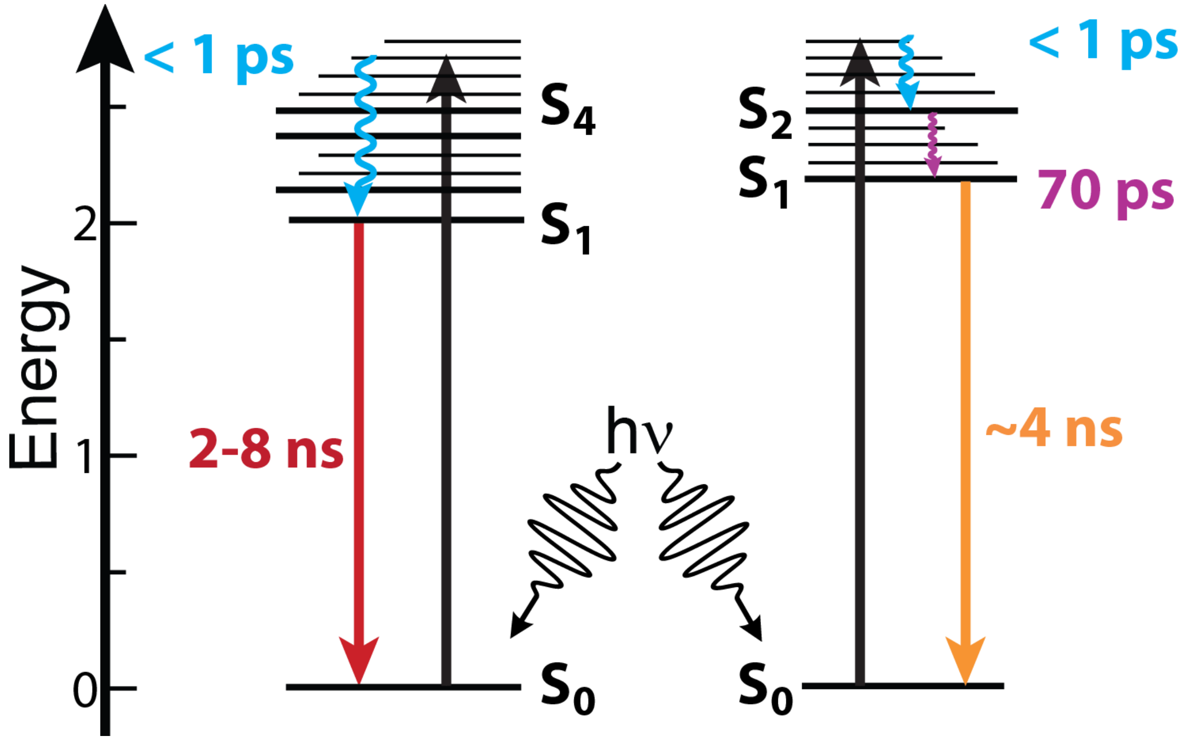

Figure S36: Proposed mechanism (left) of the monomeric species of 2[EtPh] and 3[nBuPh] and (right) 4[Hexyl].

DOSY NMR

The DOSY NMR experiments were carried out with a Bruker AV-400 with a CryoPlatform cryoprobe. The sample solutions were prepared at a concentration of ~500 µM and transferred to an NMR tube. All DOSY experiments were performed by using a bipolar gradient pulse paired simulated echo and ledbpgp2s1d pulse sequence at a temperature of 298 K. The longitudinal eddy current delay and the gradient recovery delay were kept at a fixed value of 5 ms. The strength of the pulsed-field gradients was incremented from 2% to 98%, while the diffusion-sensitive period (50 ms) and the gradient duration (0.75 ms) were optimized to obtain a signal-to-noise ratio of >5%. An average of 16 scans were obtained and processed by using Dynamics Center software. The diffusion coefficients were obtained by integrating ^{1}H NMR peaks between 0 and 10 ppm for each 2D DOSY NMR spectrum. The average diffusion coefficients were then calculated using the SEGWE software.[2]

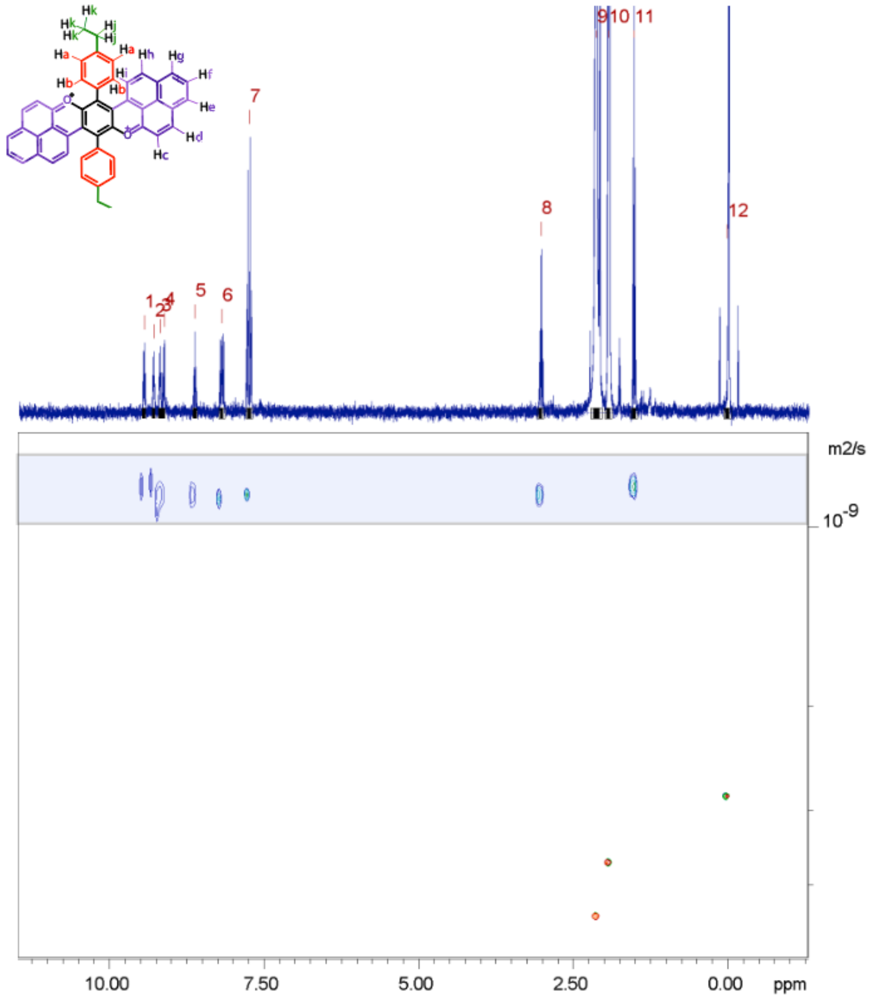

Figure S37: DOSY NMR of 2[EtPh] in acetonitrile only with TMS as an internal standard.

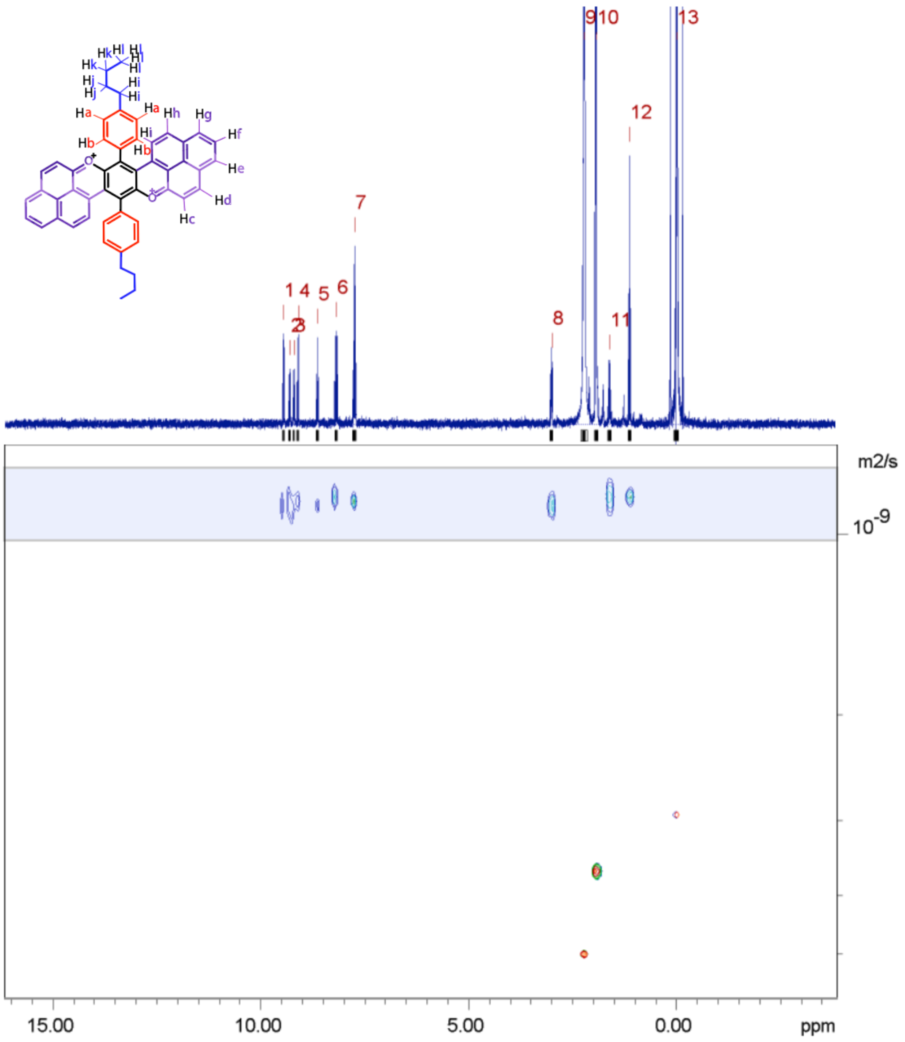

Figure S38: DOSY NMR of 3[nBuPh] in acetonitrile only with TMS as an internal standard.

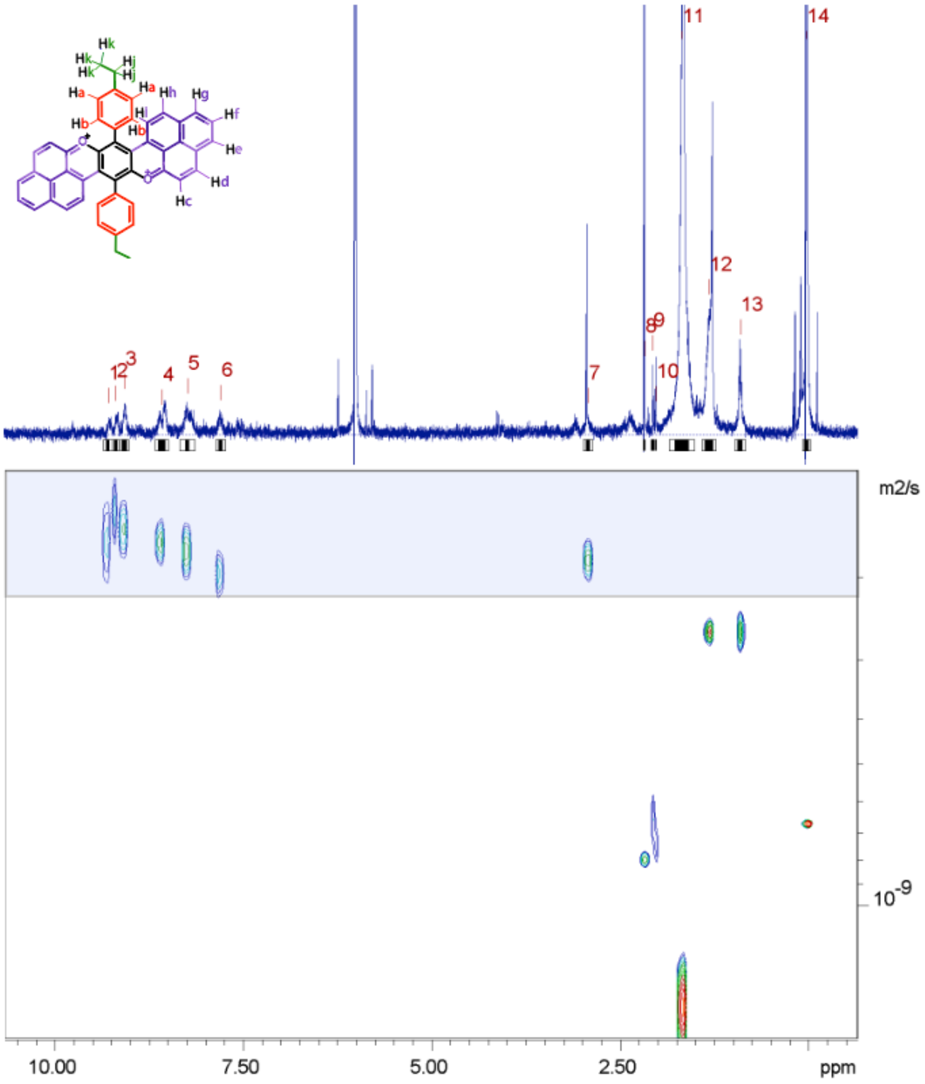

Figure S39: DOSY NMR of 2[EtPh] in TCE only with TMS as an internal standard.

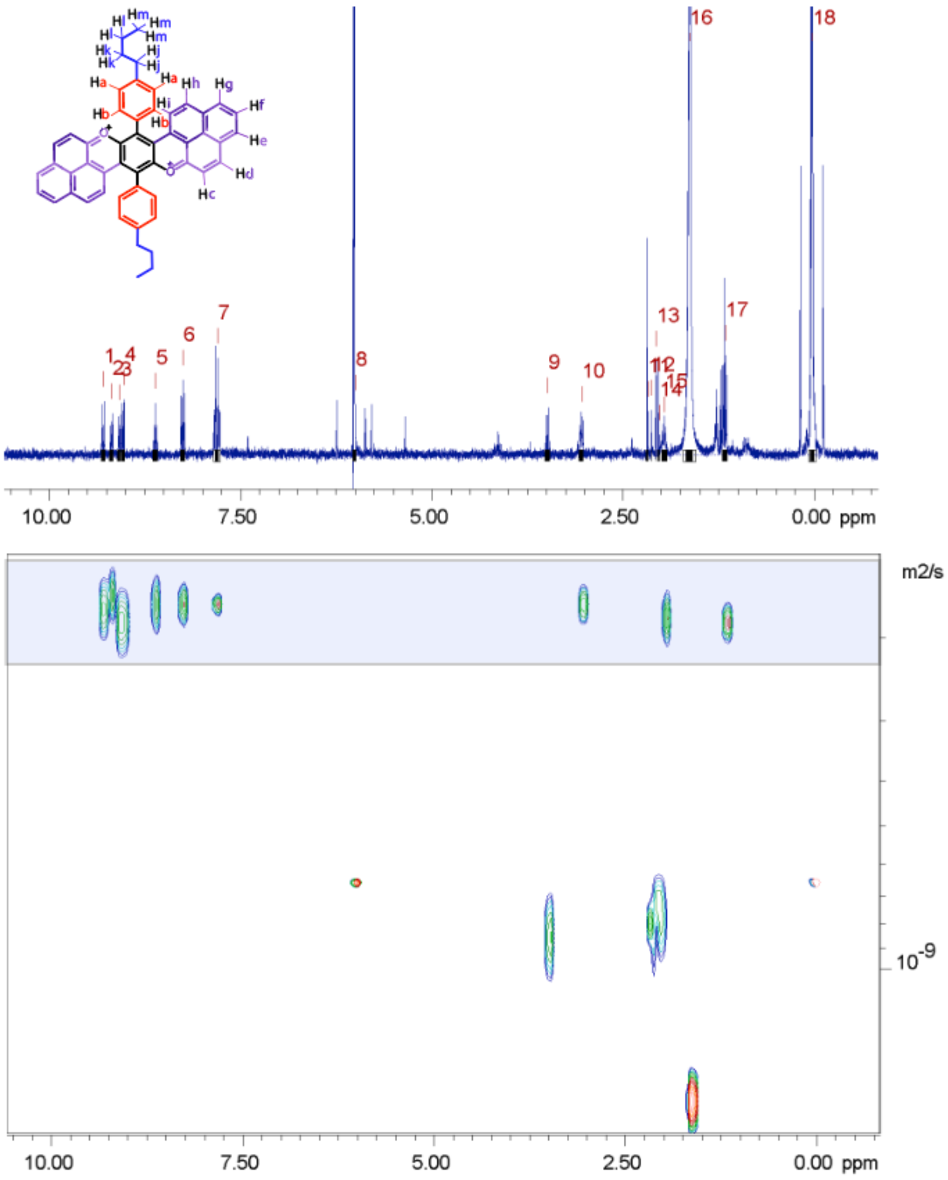

Figure S40: DOSY NMR of 3[nBuPh] in TCE only with TMS as an internal standard.

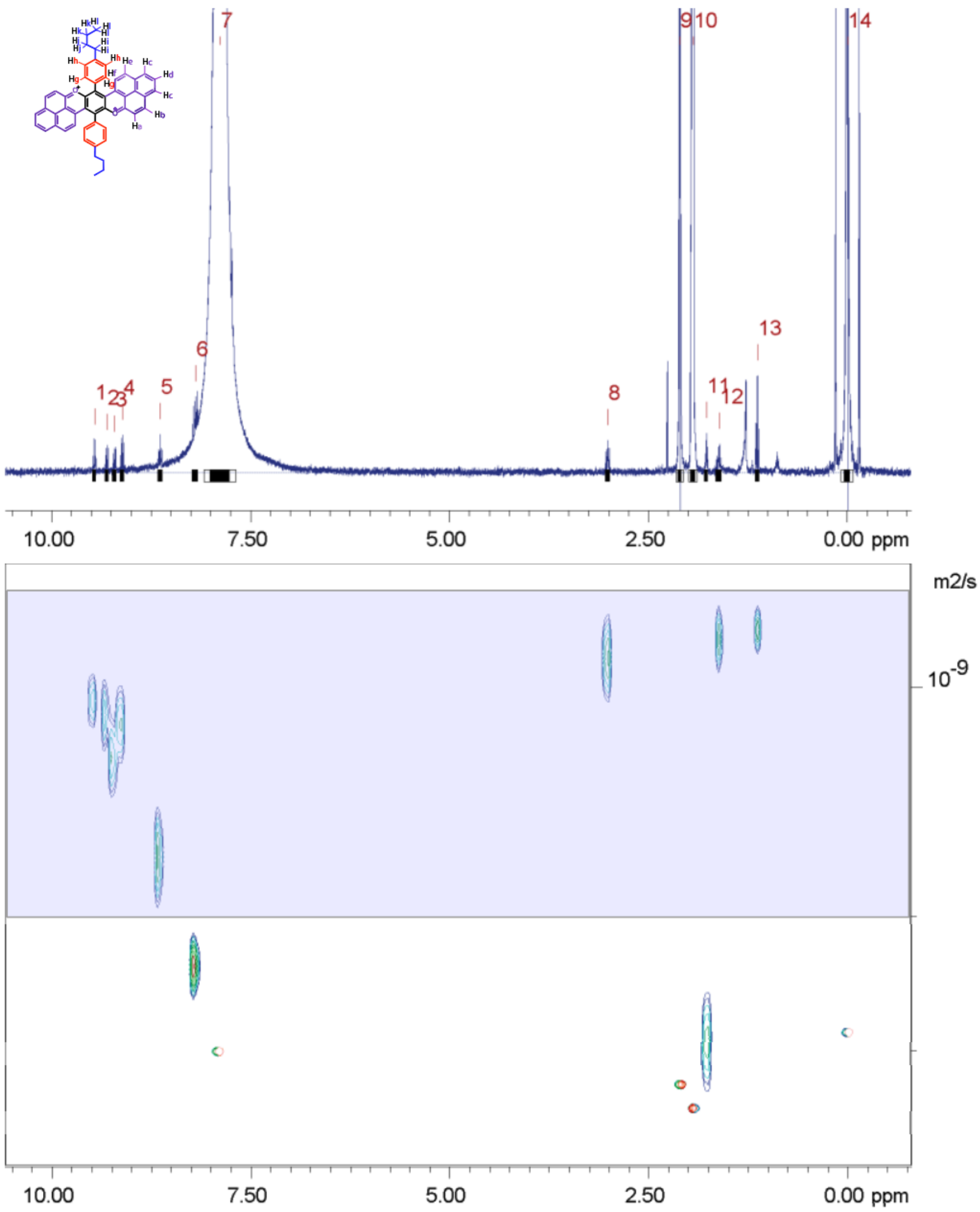

Figure S41: DOSY NMR of 3[nBuPh] in ACN and TFA (1:0.1 molar ratio) with TMS as an internal standard.

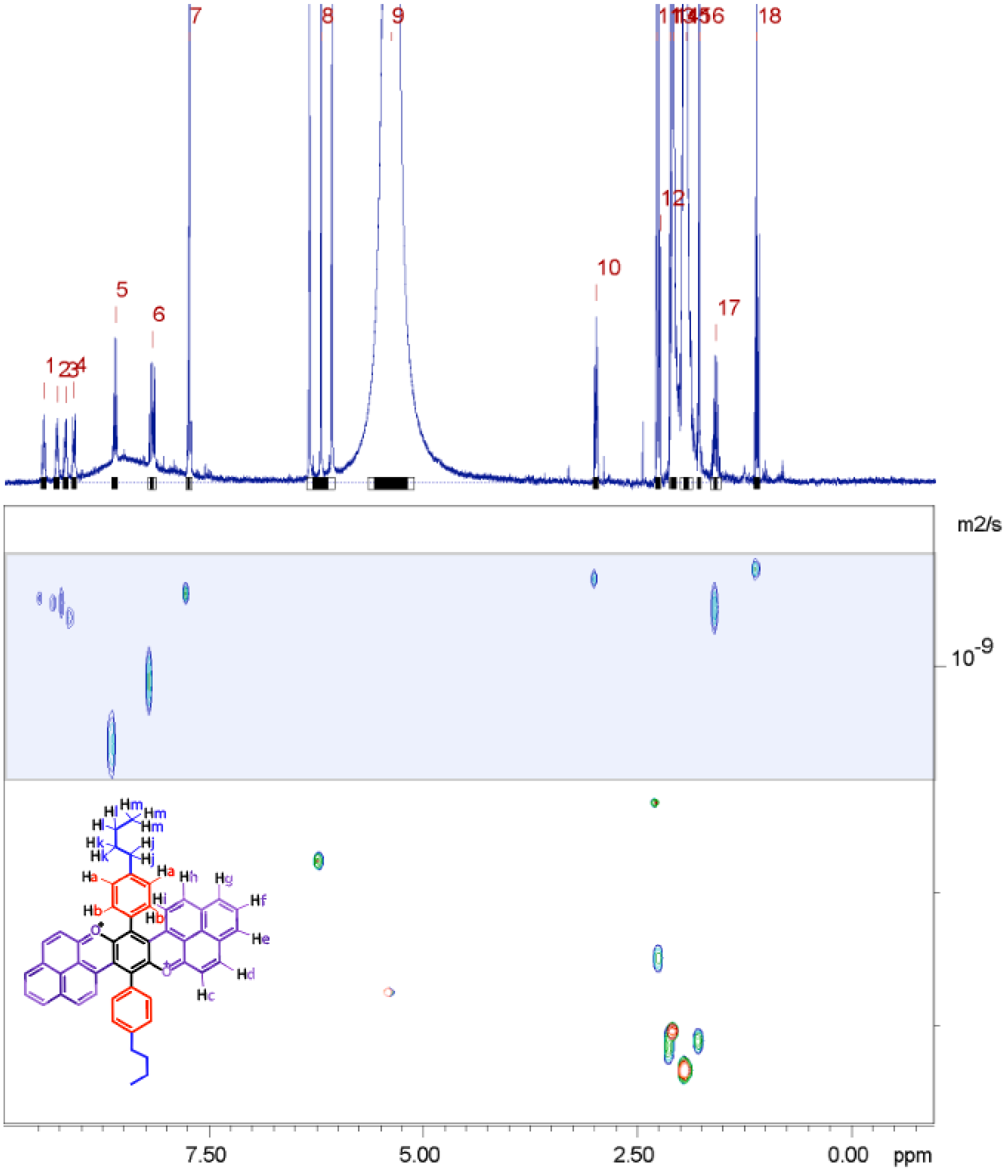

Figure S42: DOSY NMR of 3[nBuPh] in ACN and HBF_4 (1:0.1 molar ratio) with TMS as an internal standard.

Transient Absorption Spectroscopy

Transient Absorption Spectroscopy Experimental Methods

Samples of 2[EtPh], 3[nBuPh], and 4[Hexyl] were prepared to 80 μM concentration in acetonitrile and placed in a 2 mm quartz cuvette fitted with a stir bar. An Ultrafast Helios spectrometer was used to collect transient absorption spectroscopy (TAS) data on 2[EtPh], 3[nBuPh], and 4[Hexyl]. A Coherent Libra amplified Ti:sapphire system at 1.1 W and 1 kHz repetition rate was used to generate 100-fs pulses of 800 nm laser light. 80% of the 800 nm pulse was split using a beam splitter and sent to a Topas-C optical parametric amplifier to generate a pump pulse of 590 nm. The pump pulse was further attenuated to ~1.0 mW. The remaining 20% of the 800 nm pulse was sent through a CaF_2 crystal to generate a white light continuum to be used as the probe pulse. The data was collected over the available 5.1 ns time window. Each dataset comprises of three scans that were averaged together with 250 points in each scan. The samples were stirred throughout the course of the TAS experiment, and the absorbance spectra was collected before and after each experiment to confirm no degradation occurred during the TAS experiment. The data was prepared in Surface Xplorer using a previously published method.[3] A chirp correction was determined from a blank sample for both excitation wavelengths and applied to each dataset, as shown in Figure S44, Figure S46, and Figure S48. A Python-based fitting program produced by the Young lab was used to perform global lifetime analysis (GLA) to determine the decay-associated difference spectra (DADS) and their associated lifetimes. Multiple fits were performed and compared with residual surfaces to determine the best fit for each dataset.

Overview of Transient Absorption Data

2[EtPh]
The representative spectra of 2[EtPh] shows similar features at 590 nm excitation (Figure S43). A prominent ground state bleach (GSB) appears at 525 nm that mirrors the steady-state absorbance spectrum, including the shoulder peaks on either side of the main feature. The GSB bleach persists with similar intensity for ~10 ps before beginning to decay back to zero over the 5 ns window. Redshifted of the GSB, an excited state absorption (ESA) peak appears with a maximum intensity at ~640 nm. On a similar timescale as the GSB, the 640 nm ESA peak begins to decay after ~10 ps with a slight redshift before decaying down to zero over the 5 ns window. Blueshifted of the GSB, two ESA features appear with maximums at 360 nm and 415 nm. In line with the other features, they persist at a similar intensity for ~10 ps. The 360 nm ESA decays to the baseline with a blueshift of ~10 nm and also becomes more flattened. The 415 nm ESA redshifts significantly to form another broader ESA feature. The new, broad ESA shows more absorption around 450 nm. A complete decay back to zero is almost full reached within the 5-ns time window as only a small percent of the original intensity is still present in the last representative spectra.

The TAS data for 2[EtPh] is best fit with three lifetimes: a short lifetime less than 1 ps (τ_1), a 100 ps lifetime (τ_2), and a 1.1 ns lifetime (τ_3). The DADS shown for τ_1 (Figure S43, left, red) is indicative of the ESA features growing in along with the loss of the ground state population. The DADS for τ_2 (Figure S43, left, green) displays the loss of the ESA features at 360 and 415 nm, as well as the redshift of the ESA at 640 nm. Some ground state population is recovered in this process. The DADS for the longest lifetime, τ_3 (Figure S43, left, purple), shows that this longest live state absorbs at 640 nm, 450 nm, and 350 nm. This is the last state that is populated before repopulation of the ground state. This is likely an emissive lifetime based off of the time-resolved photoluminescence data shown in Figure S32.

3[nBuPh]
The representative spectra of 3[nBuPh] shows similar features and decay trends from 590 nm excitation (Figure S43). Generally, TAS of 3[nBuPh] appears to be very similar to TAS of 2[EtPh]. The GSB of

3[nBuPh], signifying the loss of the ground state population, appears as a negative feature at 525 nm and mirrors the steady-state absorbance spectrum well, including the shoulders on either side. The GSB remains at a relatively steady intensity for 5-10 ps before decaying back to zero. The ESA feature redshifted of the bleach has a characteristic peak at ~635 nm that very slightly redshifts before it decays. Two ESA features are present blueshifted of the bleach at ~370 and 415 nm. The 370 nm peak blueshifts about 10 nm as it decays, and the 415 nm peak redshifts significantly to form a new ESA feature with a broader absorption center around 440 nm. An absorbance increase at 450-460 nm is observed with this redshift. The TAS signal decays almost completely back to zero within the 5-ns time window.

The TAS data for 3[nBuPh] is best fit with 3 lifetimes, similar to 2[EtPh]: a short lifetime less than 1 ps (τ_1), a 45 ps lifetime (τ_2), and a 1.1 ns lifetime (τ_3). The DADS shown for τ_1 (Figure S43, center, red) displays the 635 nm ESA growth in the negative feature of the DADS at that wavelength. For τ_2 (Figure S43, center, green), the DADS shows the loss of the ESA features blueshifted of the GSB with some recovery of the ground state population. For τ_3 (Figure S43, center, purple) with an average lifetime of 1.1 ns, the final state that is reached has ESA features at 640 nm, 450 nm, and 350 nm, similar to 2[EtPh]. This is also likely an emissive lifetime based off of the time-resolved photoluminescence data shown in Figure S33.

4[Hexyl]

The TAS data of 4[Hexyl] is similar with 590 nm excitation. A GSB is centered at 510 nm with slight shoulders on either side, mirroring the steady-state absorption features well. There is an additional GSB feature at 570 nm that stems from the additional steady-state absorption feature present at 570 nm that is not present in 2[EtPh] or 3[nBuPh]. The extent of this GSB at 570 nm differs in intensity between the excitation wavelengths. There is broader positive absorption extending through the remaining optical window up to 680 nm. Like 2[EtPh] and 3[nBuPh], the GSB and ESA features redshifted of the GSB grow in and persist at a relatively consistent intensity for ~10 ps before beginning to decay. Blueshifted of the bleach appears to be multiple ESA features. The two most prominent features appear at 350 nm and 430 nm. Additional positive absorption is present between these two peaks with less defined peaks. As the 430 nm feature decays, it redshifts slightly ~10 nm. This redshift does not produce a new prominent ESA feature like 2[EtPh] and 3[nBuPh]. Generally, all features produced from 590 nm excitation of 4[Hexyl] decay with minimal shifting back towards zero. The signal does not almost fully decay back to zero like in 2[EtPh] or 3[nBuPh]. A significant amount of intensity, ~30%, still remains at the end of the 5-ns time window.

The TAS data of 4[Hexyl] is best fit with three lifetimes: a short lifetime less than 1 ps (τ_1), a 30 ps lifetime (τ_2), and a 4.2 ns lifetime (τ_3). The DADS from τ_1 (Figure S43, right, red) show the initial growth of the ESA redshifted of the bleach due to the negative nature of the DADS, and the decay of the ESA blueshifted of the bleach form the positive nature of the DADS, with a bit of repopulation of the ground state population. Both ESAs are then shown to decay through the DADS of τ_2 (Figure S43, right, green) along with further repopulation of the ground state population. The final lifetime, τ_3 (Figure S43, right, purple), then shows the final state likely reached before full repopulation of the ground state. This is likely an emissive lifetime based off of the time-resolved photoluminescence data shown in Figure S34.

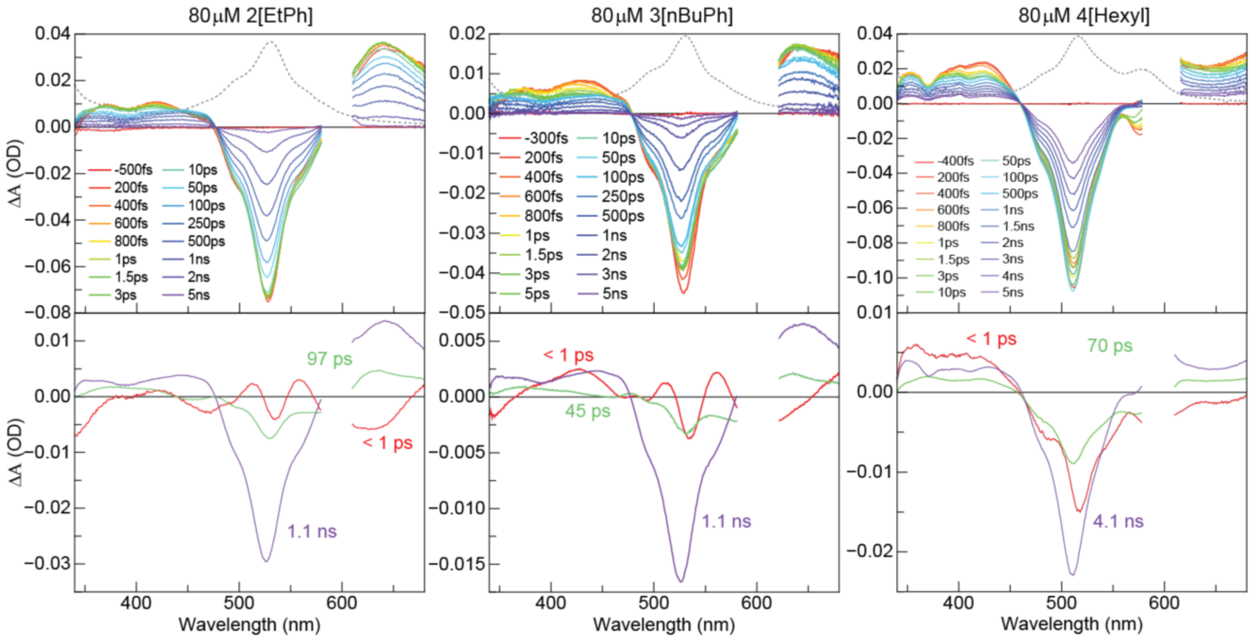

Figure S43. (Top) Representative spectra of each molecule in acetonitrile at 590 nm ex. (Bottom) DADS determined from GLA fitting with lifetimes.

Each dataset also includes the ground-state absorption (black dashed line) overlaid for reference. The ground-state absorption spectrum for bisphenalenyl molecule (black dash line) overlaid for reference.

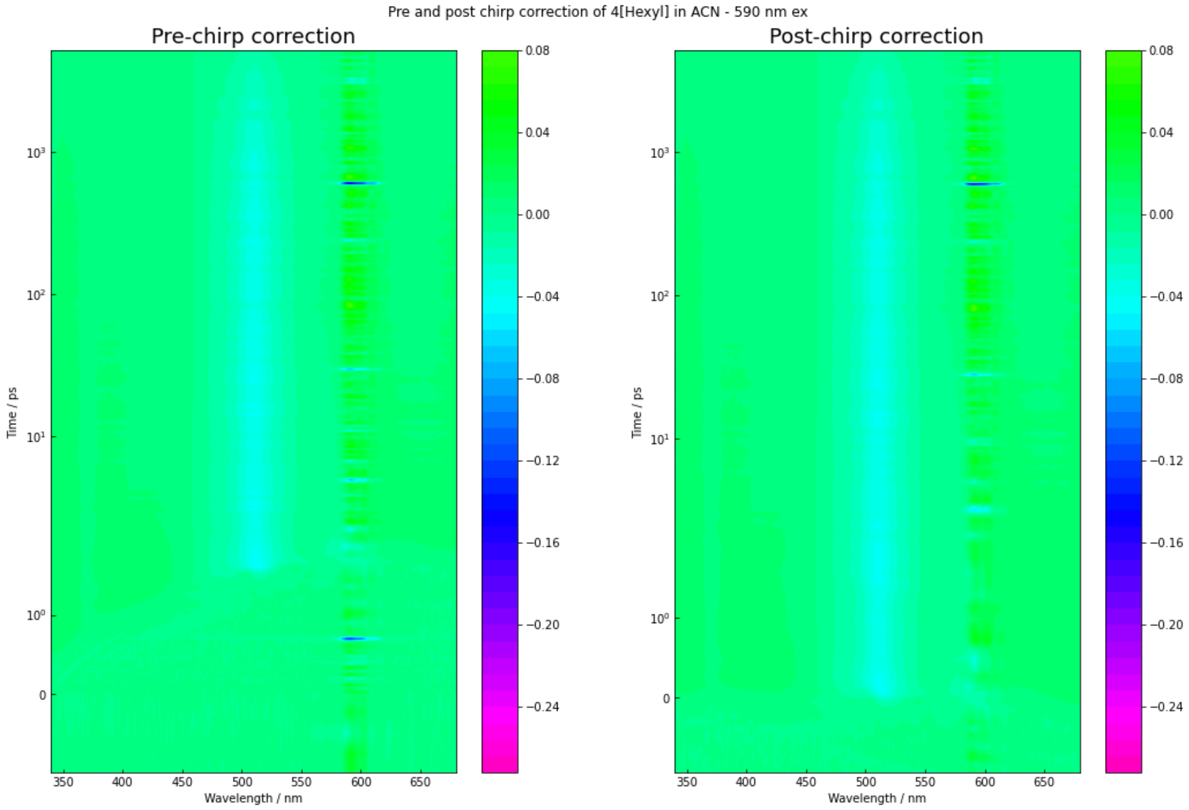

Figure S44. TAS heat map of 80 µM solution of 4[Hexyl] in acetonitrile before (left) and after (right) chirp correction.

Left) Raw pump-probe heat map at 590 nm excitation. x-axis = wavelength; y-axis = delay step position (exponential spacing); color map corresponds to ΔA. Right) Corrected linear time pump-probe map at 590 nm excitation. x-axis = wavelength; y-axis = delay step position; color map corresponds to ΔA.

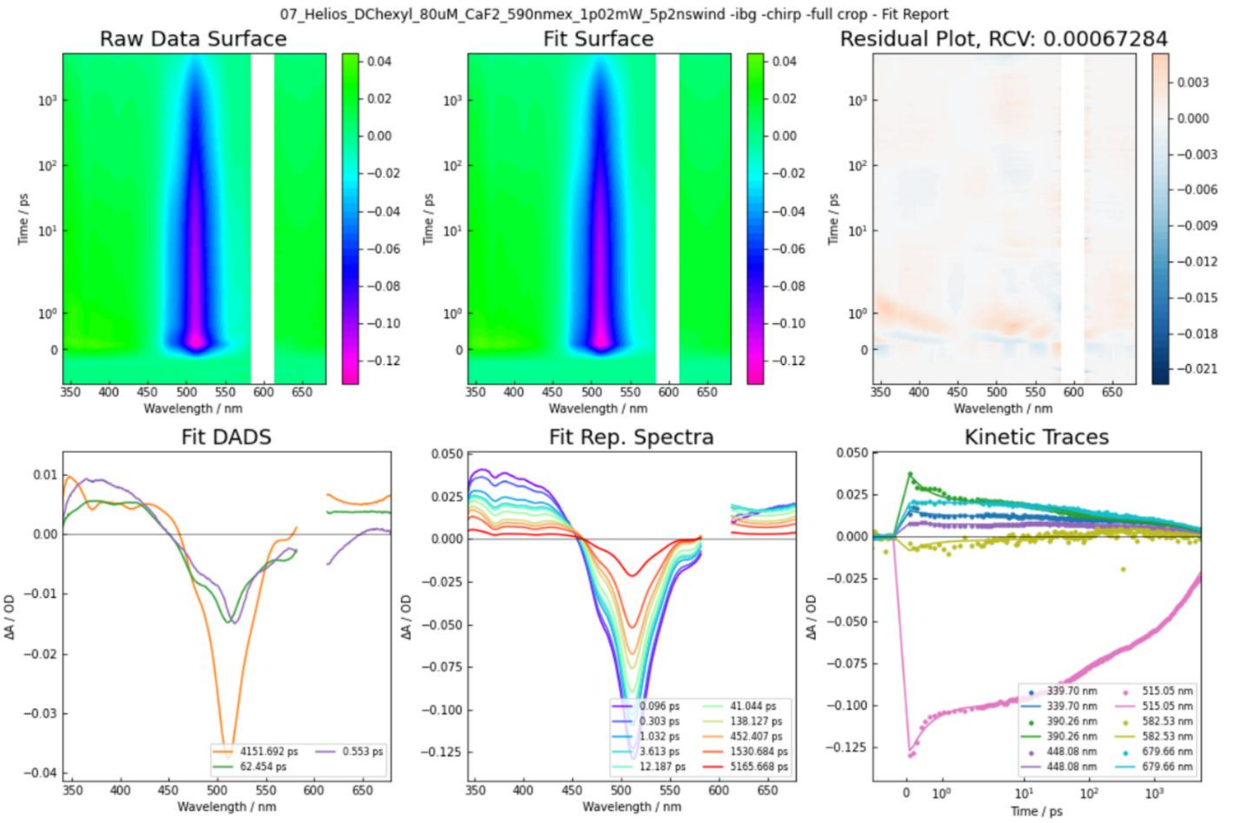

Figure S45. TAS data and fits of 4[Hexyl] in acetonitrile.

Top left) Chirp-corrected pump-probe heat map at 590 nm excitation. x-axis = wavelength; y-axis = delay step position; color map corresponds to ΔA. Top center) Reconstructed surfaces from fit DADS. Top right) Fit residual plots. Bottom right) SVD global fitting spectral components (DADS). Bottom center) Selected representative spectra depicting raw data surface. Bottom right) Single value kinetic traces of TAS data at select wavelengths.

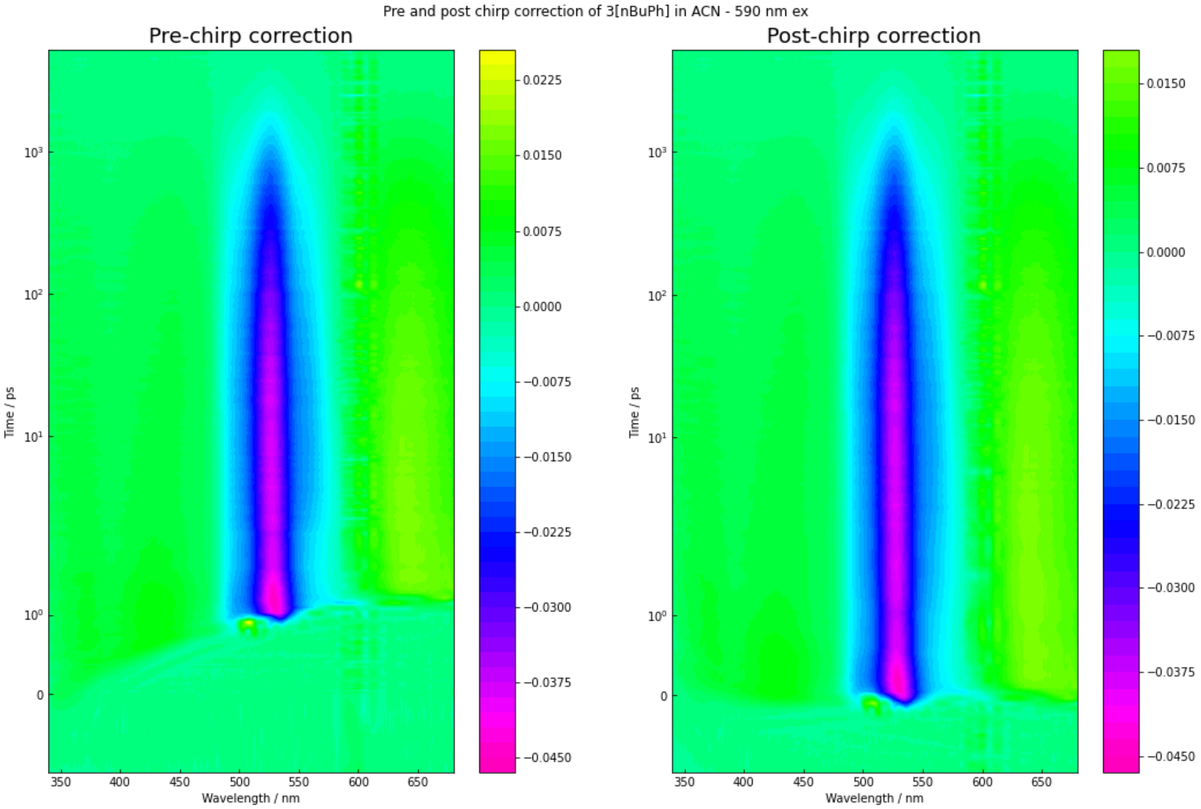

Figure S46. TAS heat map of 80 µM solution of 3[nBuPh] in acetonitrile before (left) and after (right) chirp correction.

Left) Raw pump-probe heat map at 590 nm excitation. x-axis = wavelength; y-axis = delay step position (exponential spacing); color map corresponds to ΔA. Right) Corrected linear time pump-probe map at 590 nm excitation. x-axis = wavelength; y-axis = delay step position; color map corresponds to ΔA.

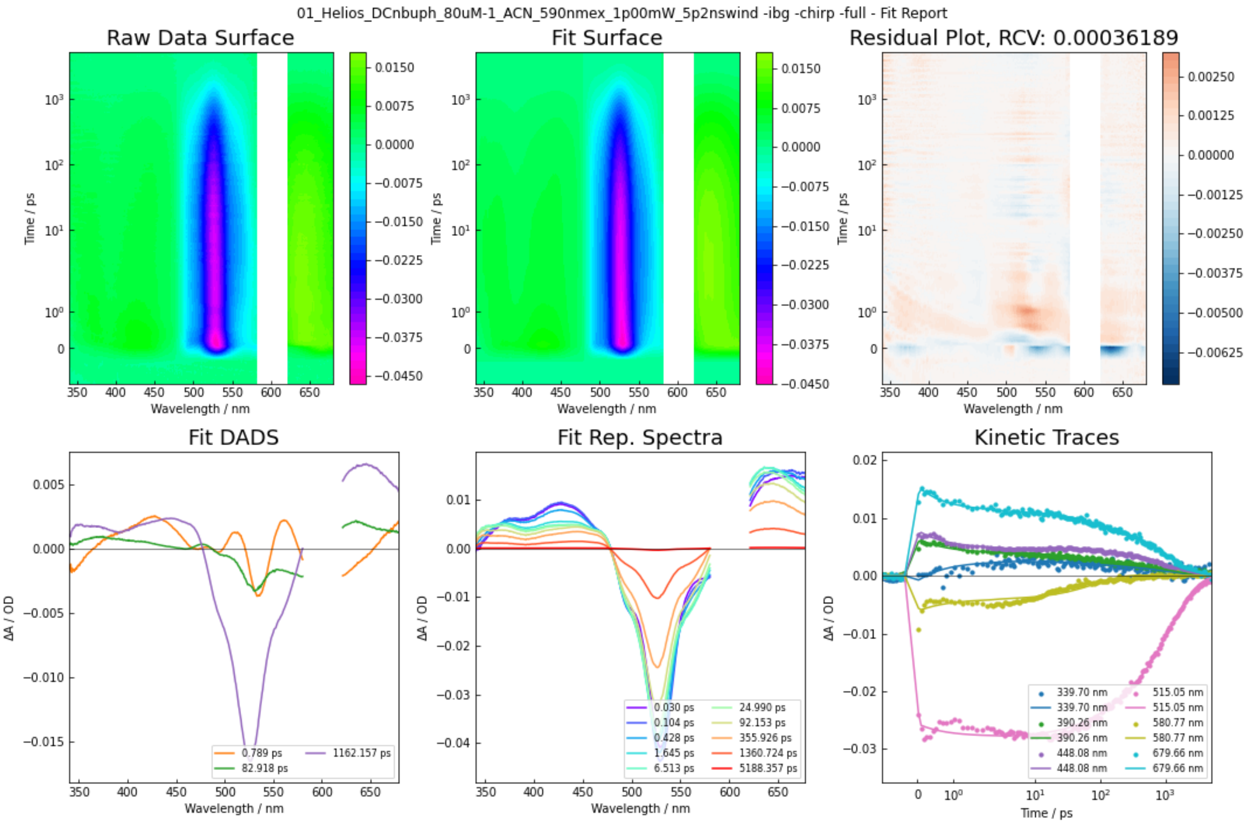

Figure S47. TAS data and fits of 3[nBuPh] in acetonitrile.

Top left) Chirp-corrected pump-probe heat map at 590 nm excitation. x-axis = wavelength; y-axis = delay step position; color map corresponds to ΔA. Top center) Reconstructed surfaces from fit DADS. Top right) Fit residual plots. Bottom right) SVD global fitting spectral components (DADS). Bottom center) Selected representative spectra depicting raw data surface. Bottom right) Single value kinetic traces of TAS data at select wavelengths.

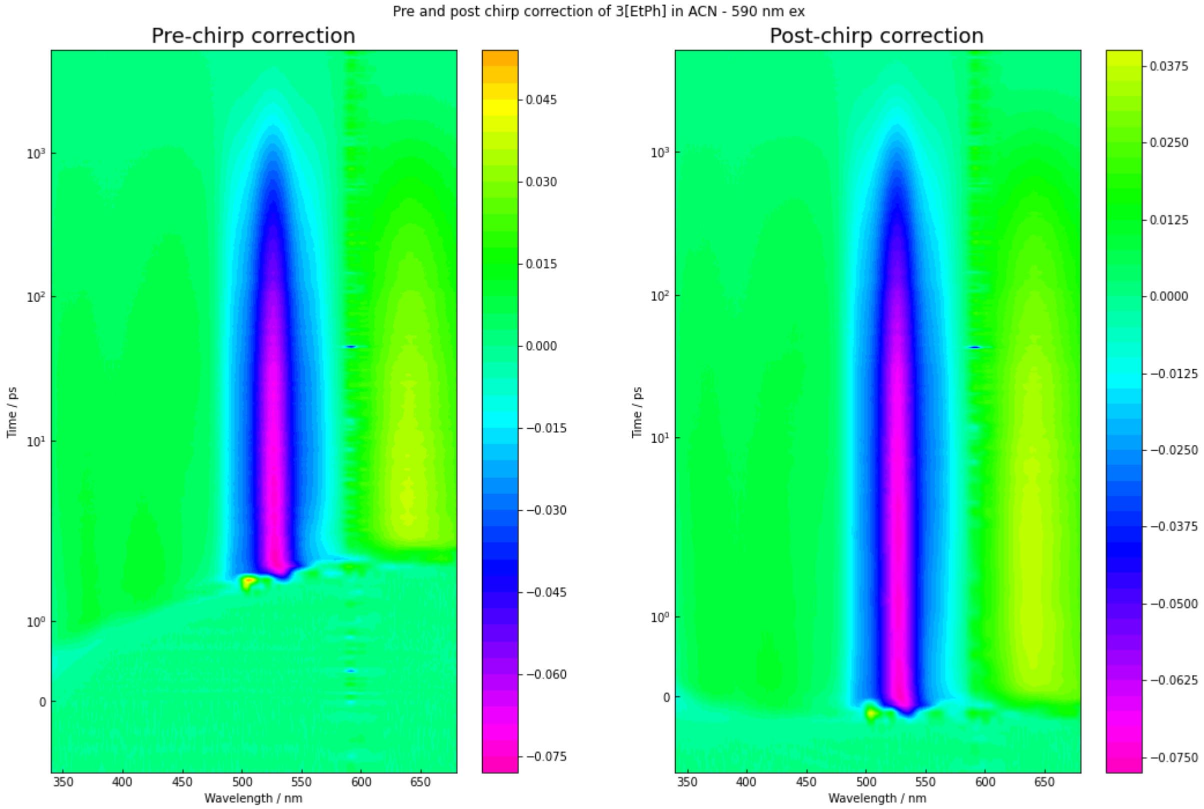

Figure S48. TAS heat map of 80 µM solution of 2[EtPh] in acetonitrile before (left) and after (right) chirp correction.

Left) Raw pump-probe heat map at 590 nm excitation. x-axis = wavelength; y-axis = delay step position (exponential spacing); color map corresponds to ΔA. Right) Corrected linear time pump-probe map at 590 nm excitation. x-axis = wavelength; y-axis = delay step position; color map corresponds to ΔA.

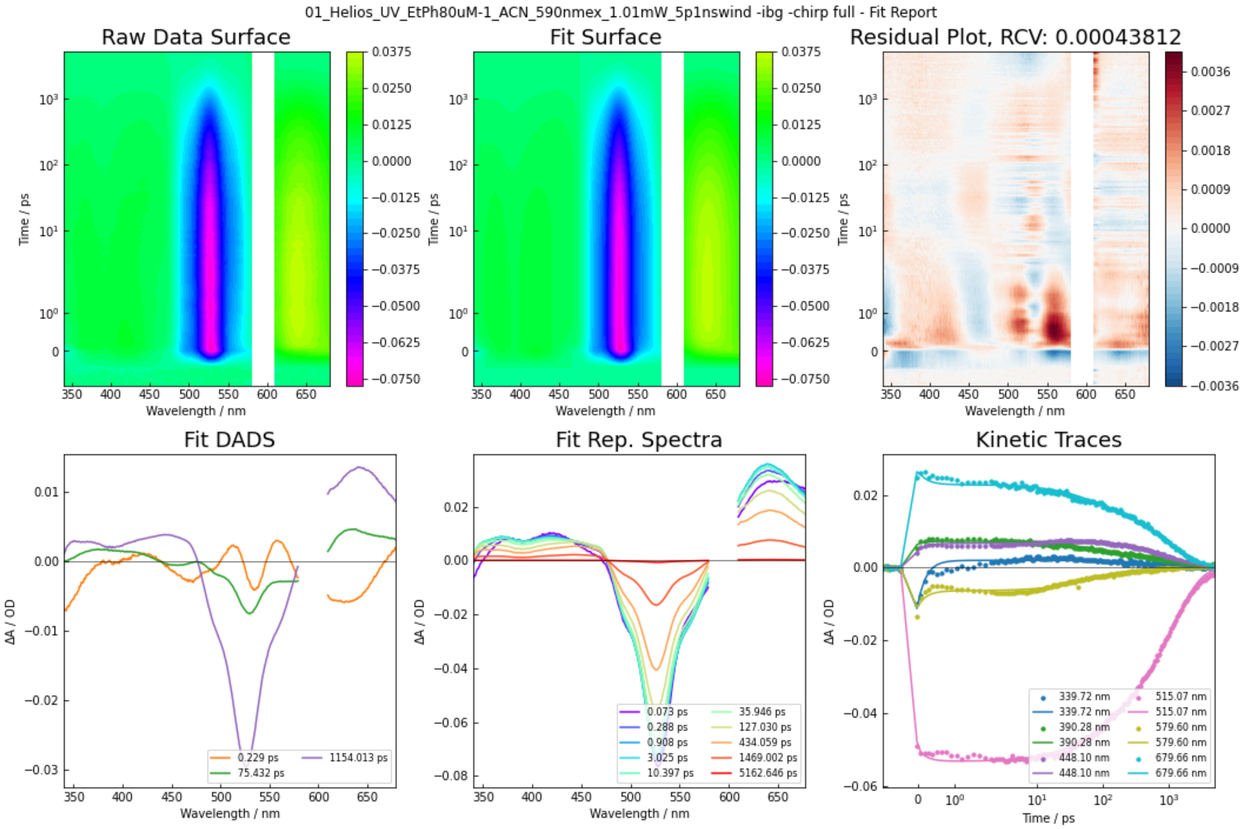

Figure S49. TAS data and fits of 2[EtPh] in acetonitrile.

Top left) Chirp-corrected pump-probe heat map at 590 nm excitation. x-axis = wavelength; y-axis = delay step position; color map corresponds to ΔA. Top center) Reconstructed surfaces from fit DADS. Top right) Fit residual plots. Bottom right) SVD global fitting spectral components (DADS). Bottom center) Selected representative spectra depicting raw data surface. Bottom right) Single value kinetic traces of TAS data at select wavelengths.

	500 nm	526 nm	540 nm
τ_1	0.37 ps	0.17 ps	0.40 ps
τ_2	117.3 ps	83.4 ps	76.9 ps
τ_3	1277 ps	1194 ps	1188 ps

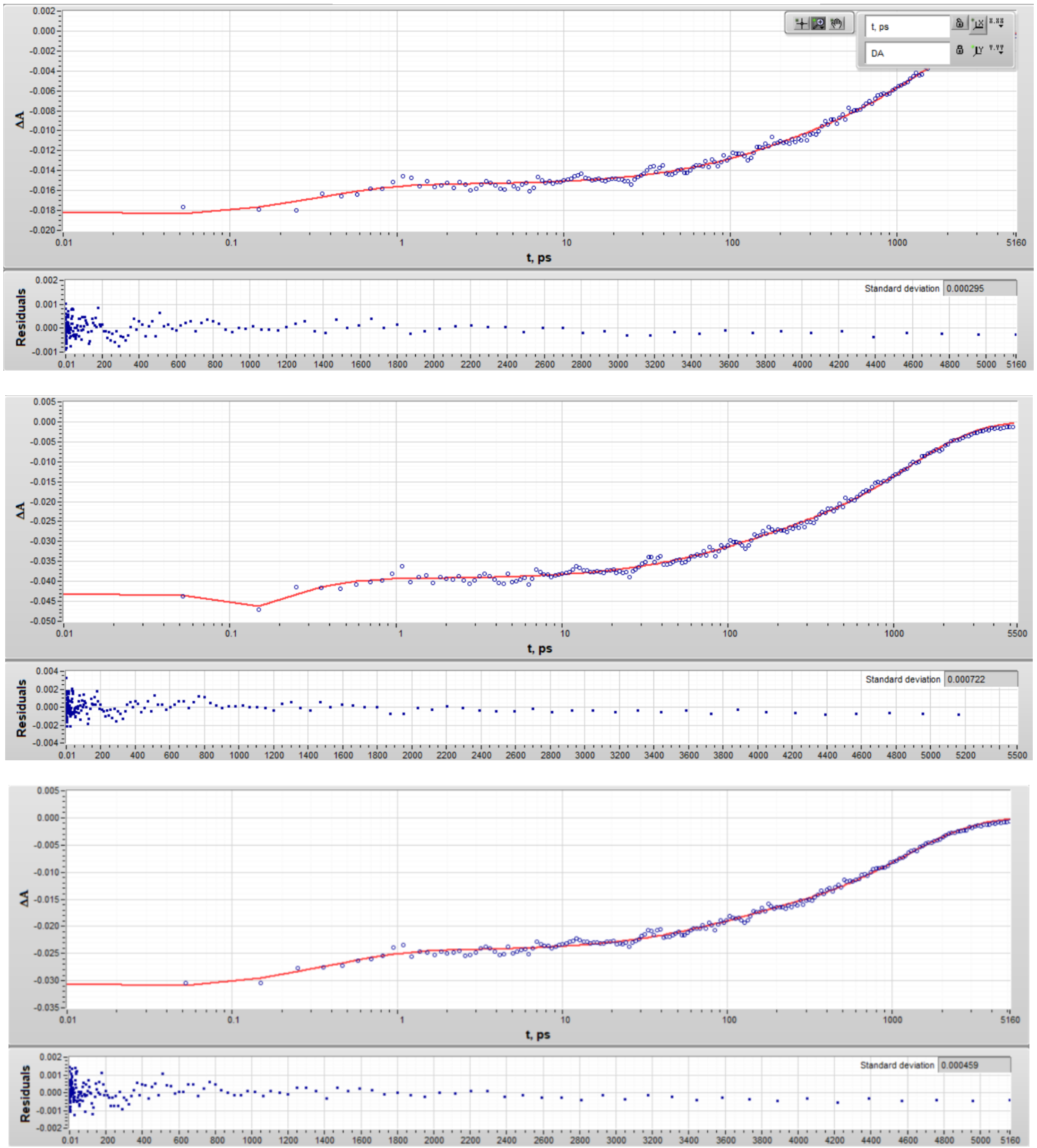

Figure S50: Single wavelength kinetic traces and fits of 2[EtPh] at 500 nm (top), 525 nm (middle), and bottom (540 nm) across the ground state bleach feature. Lifetimes resulting from the best fit are shown at the top.

	495 nm	529 nm	565 nm
τ_1	0.25 ps	0.22 ps	1.65 ps
τ_2	55.1 ps	41.4 ps	24.8 ps
τ_3	1181 ps	1208 ps	1136 ps

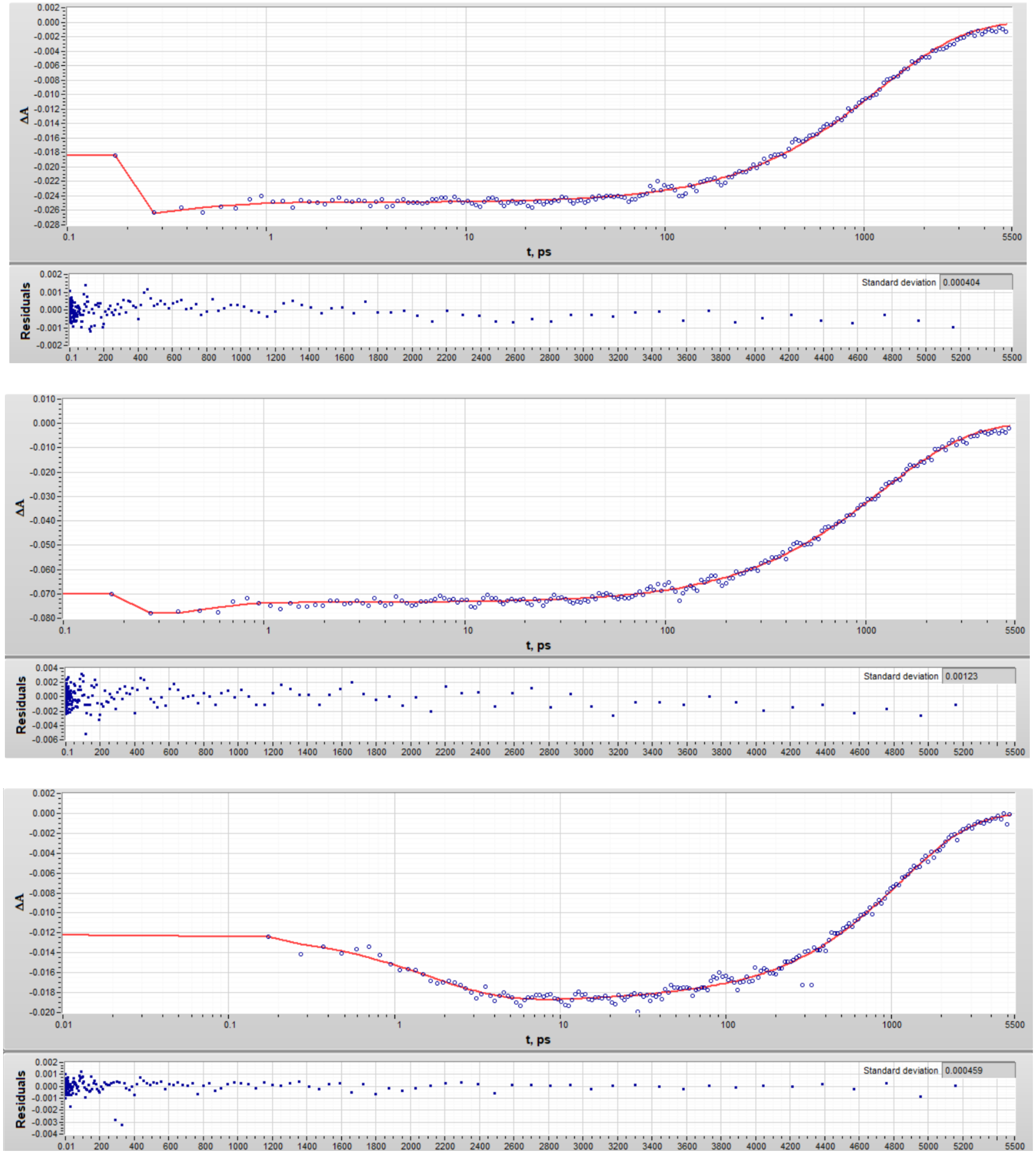

Figure S51: Single wavelength kinetic traces and fits of n[nBuPh] at 495 nm (top), 529 nm (middle), and bottom (565 nm) across the ground state bleach feature. Lifetimes resulting from the best fit are shown at the top.

	500 nm	510 nm	525 nm
τ_1	0.71 ps	0.68 ps	0.61 ps
τ_2	78.7 ps	76.9 ps	79.6 ps
τ_3	4276 ps	4262 ps	4261 ps

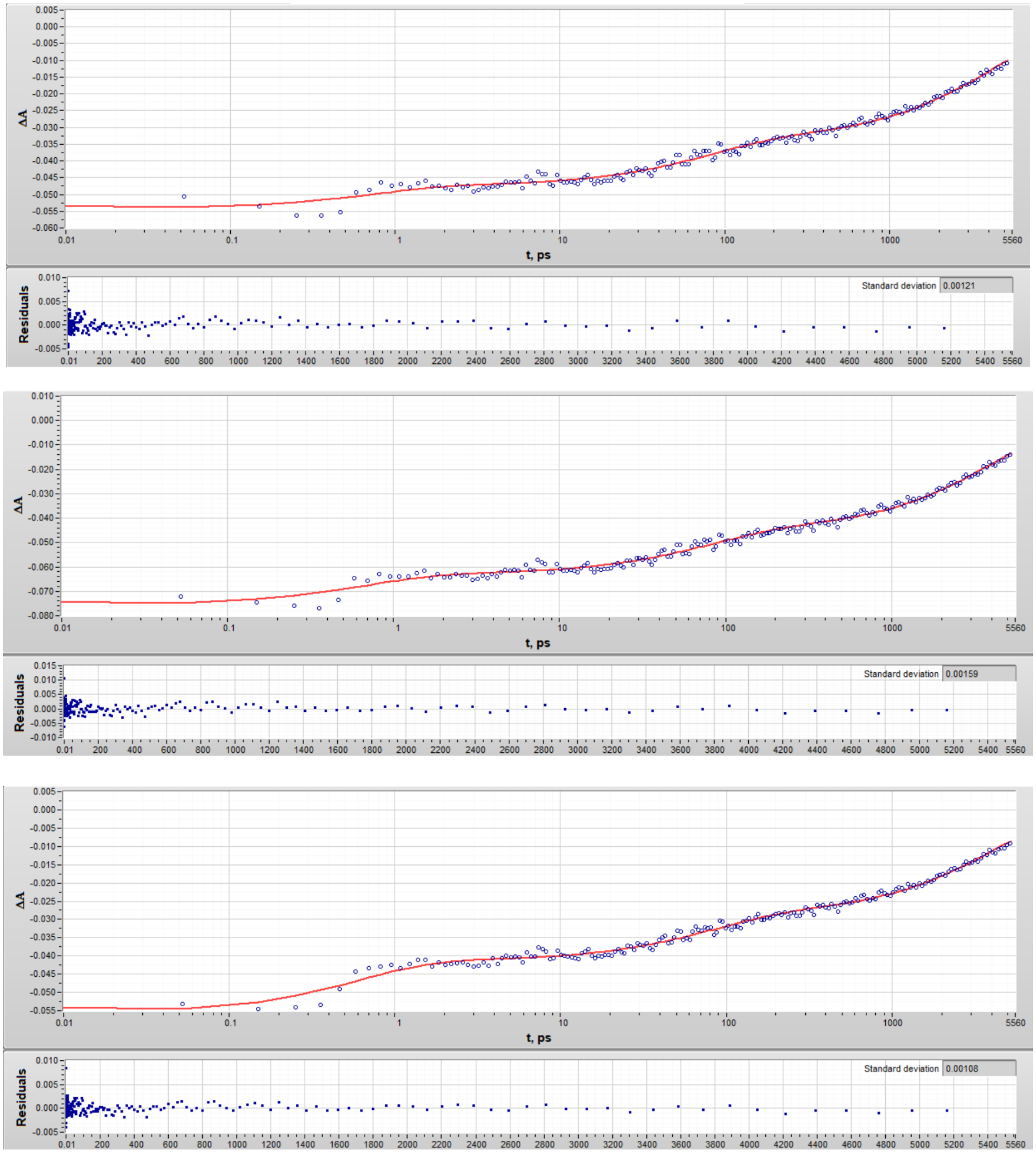

Figure S52: Single wavelength kinetic traces of 4[Hexyl] at 500 nm (top), 510 nm (middle), and bottom (525 nm) across the ground state bleach feature. Lifetimes resulting from the best fit are shown at the top.

Computational Calculations

Ground and Emissive Excited State Structures

All calculations for single molecules and dimers were performed using the Gaussian09 and Gaussian16 programs, respectively. The relaxed ground state geometries of **1, 2, and 3** were optimized using the B3LYP hybrid functional with a double-ζ basis set including polarization and dispersion, 6-31+G(d,p), and a complete polarizable continuum model (PCM) solvent description of acetonitrile. In addition, dispersion was accounted for using the GD3 correction. Each ground state was run with the known charge and multiplicity, +2 singlet. Each singlet was checked for open-shell character through a stability check and each geometry was confirmed as a true minimum with no imaginary frequencies.

All optimized xyz files can be downloaded at https://github.com/fredingroup/PCPLdimers

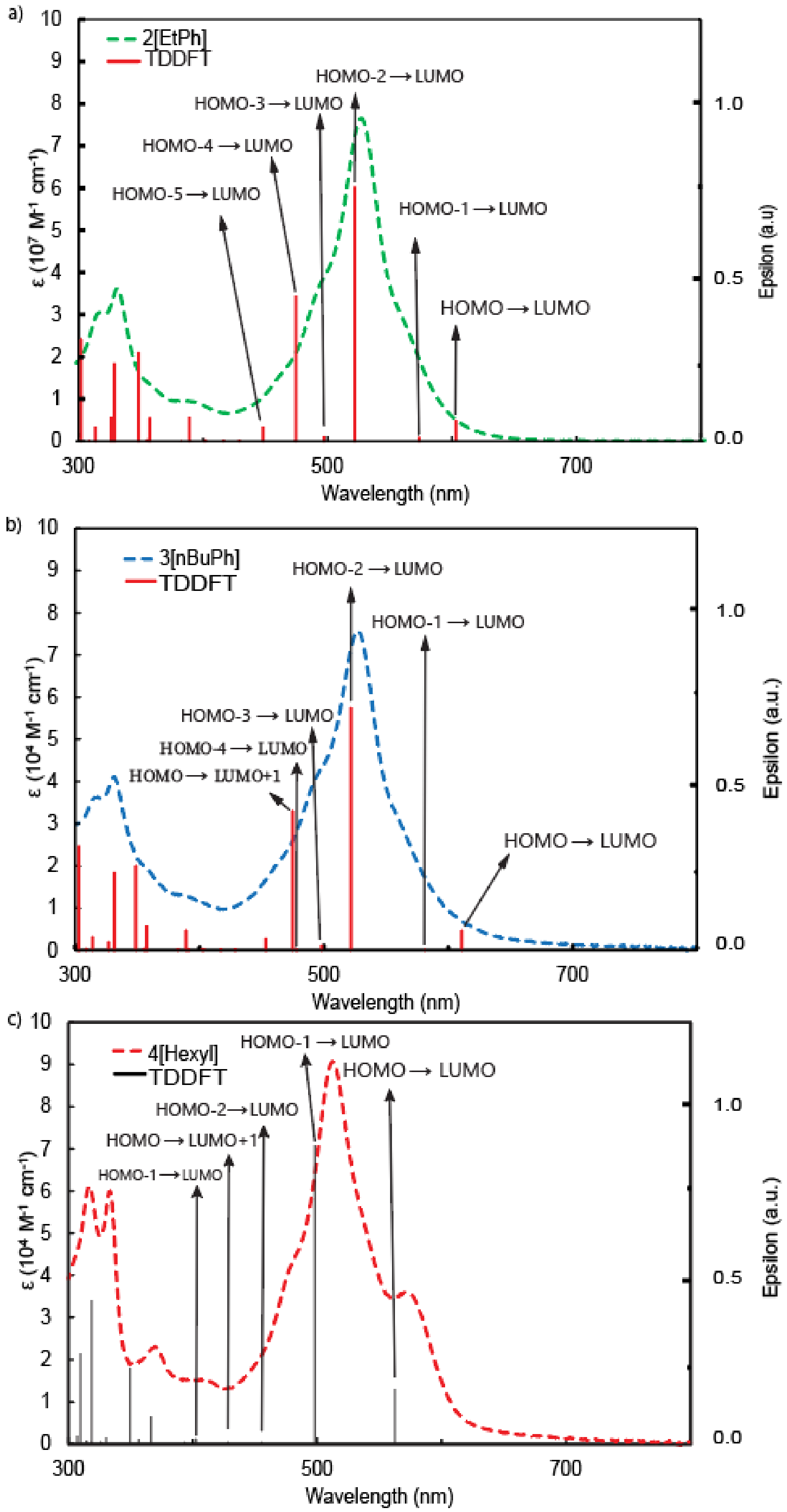

Figure S53: Electronic transition states of 2[EtPh], 3[nBuPh], 4[Hexyl].
a) Absorption spectra (green dash) overlapped with TDDFT sticks of monomer (red) and the transition states of 2[EtPh]. b) Absorption spectra (blue dash) overlapped with TDDFT sticks of monomer (red) and the transition states of 3[nBuPh]. c) Absorption spectra (red dash) overlapped with TDDFT sticks of monomer (black) and the transition states of 4[Hexyl]. B3LYP-D3/6-31+G(d,p)/PCM(ACN).

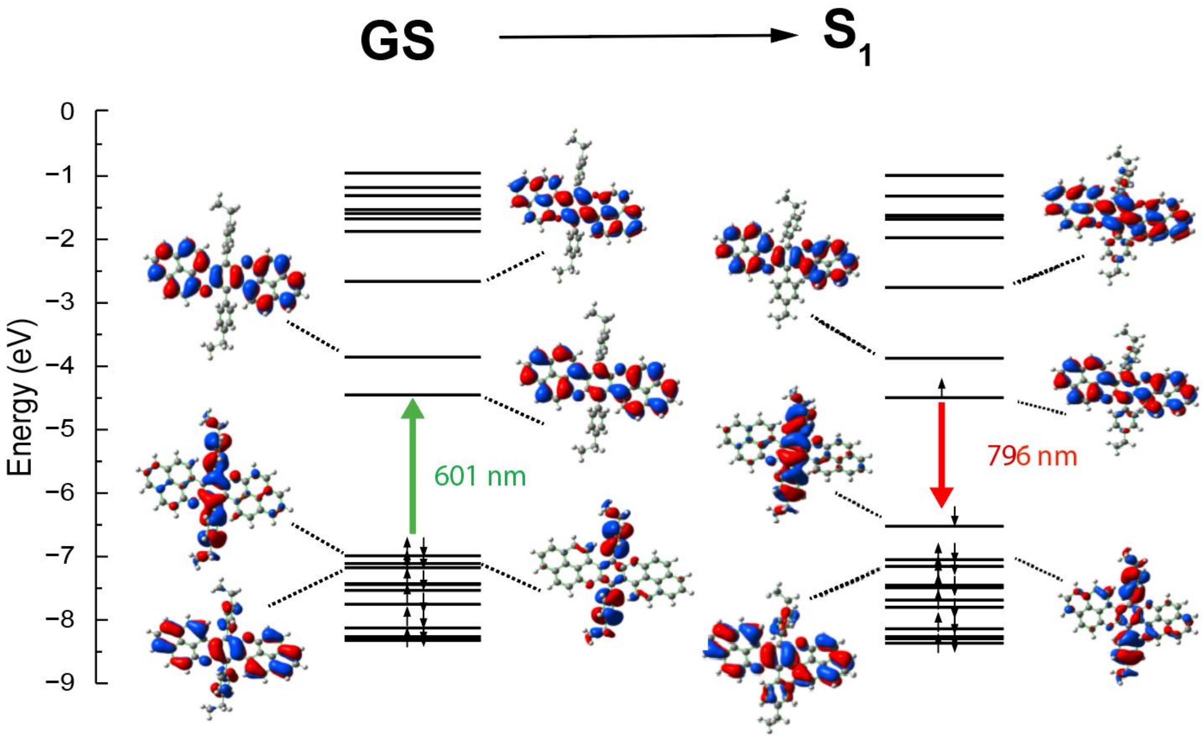

Figure S54: Molecular orbital diagram of 2[EtPh] and predicted PL mechanism. B3LYP-D3/6-31+G(d,p)/PCM(ACN).

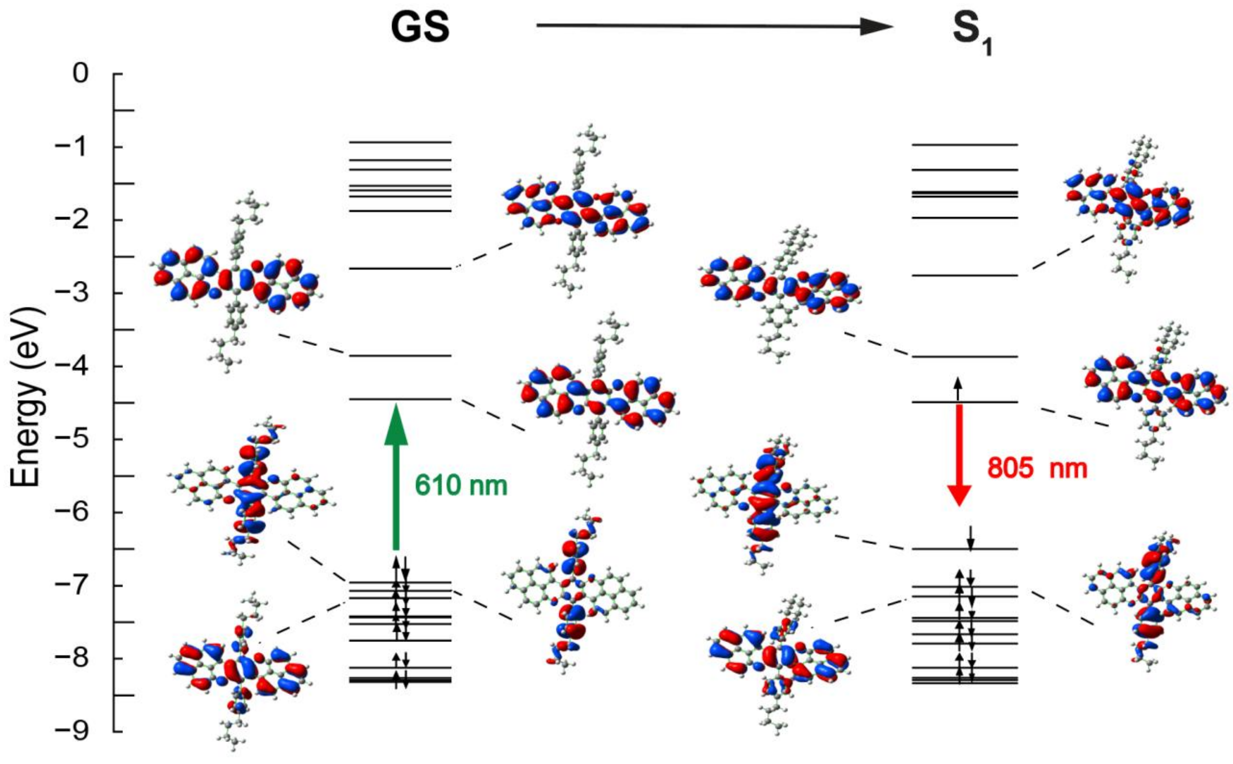

Figure S55: Molecular orbital diagram of 3[nBuPh] and predicted PL mechanism. B3LYP-D3/6-31+G(d,p)/PCM(ACN).

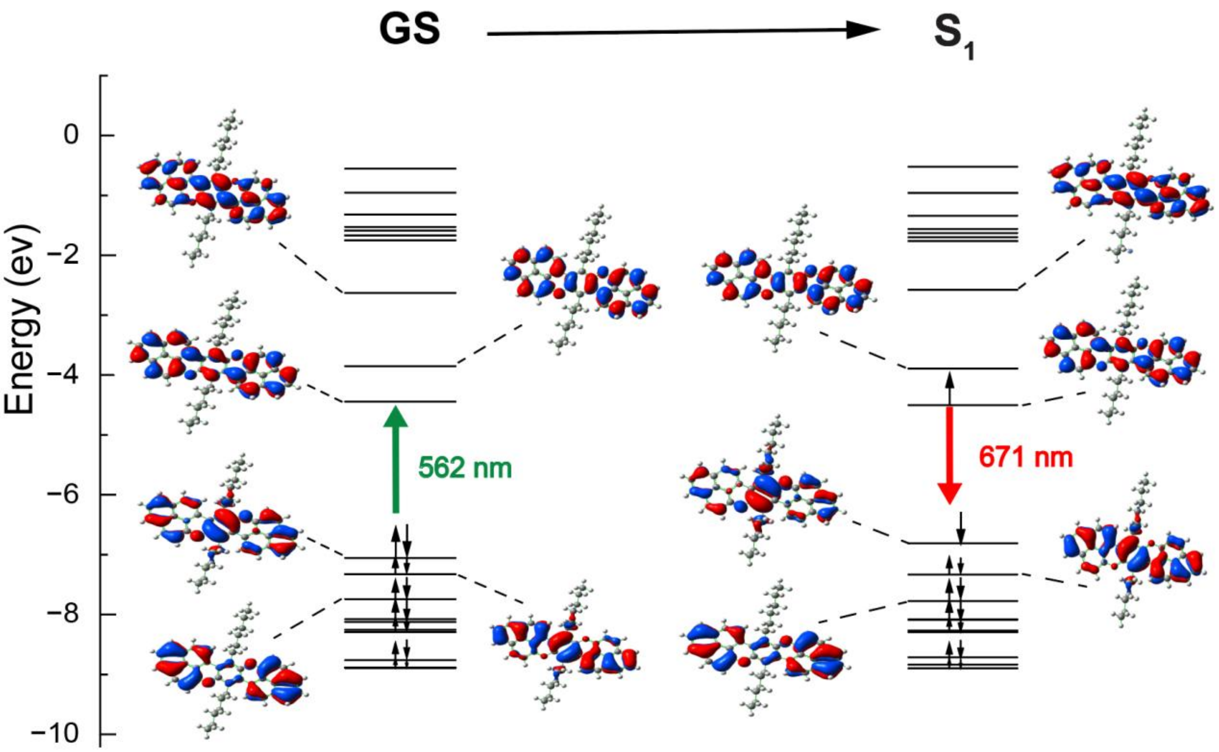

Figure S56: Molecular orbital diagram of 4[Hexyl] and predicted PL mechanism. B3LYP-D3/6-31+G(d,p)/PCM(ACN).

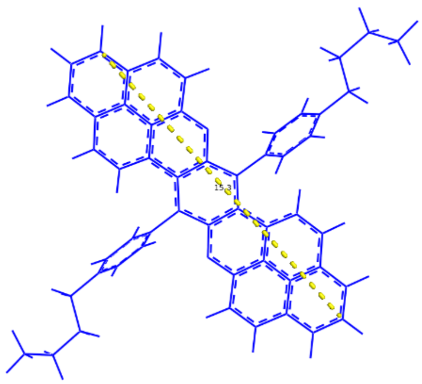

Figure S57: Diameter of simulated geometry structure of 3[nBuPh] to calculate bare radius of molecule (7.53 Å) using PyMol software. B3LYP-D3/6-31+G(d,p)/PCM(ACN).

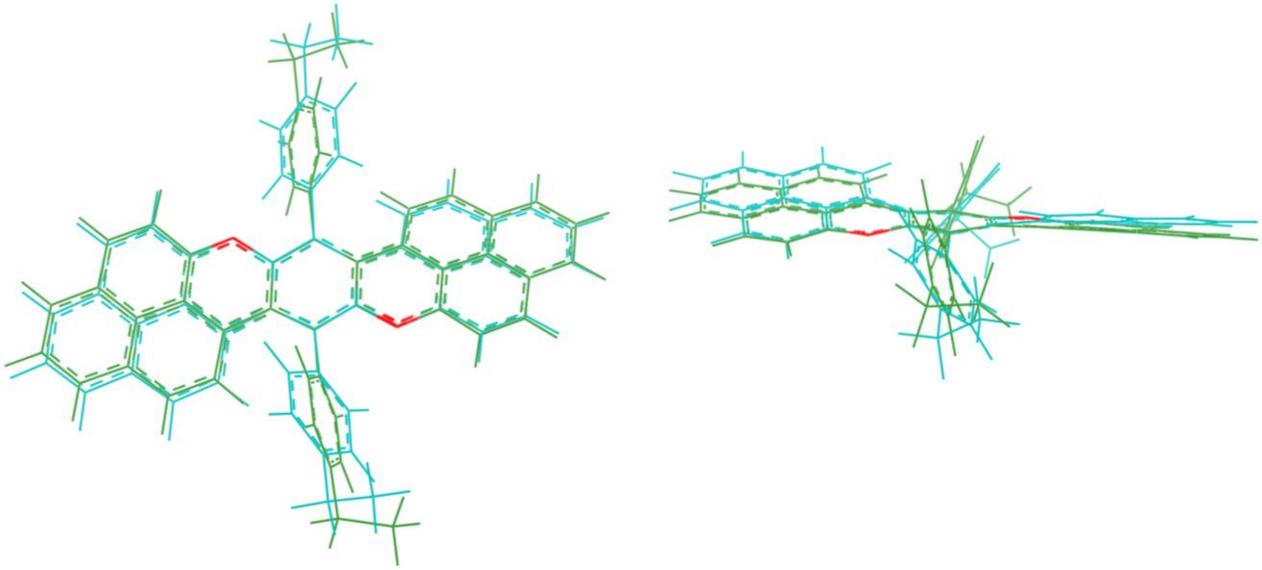

Figure S58: Geometry structure of 2[EtPh] GS (green) and opt S1 (cyan). B3LYP-D3/6-31+G(d,p)/PCM(ACN).

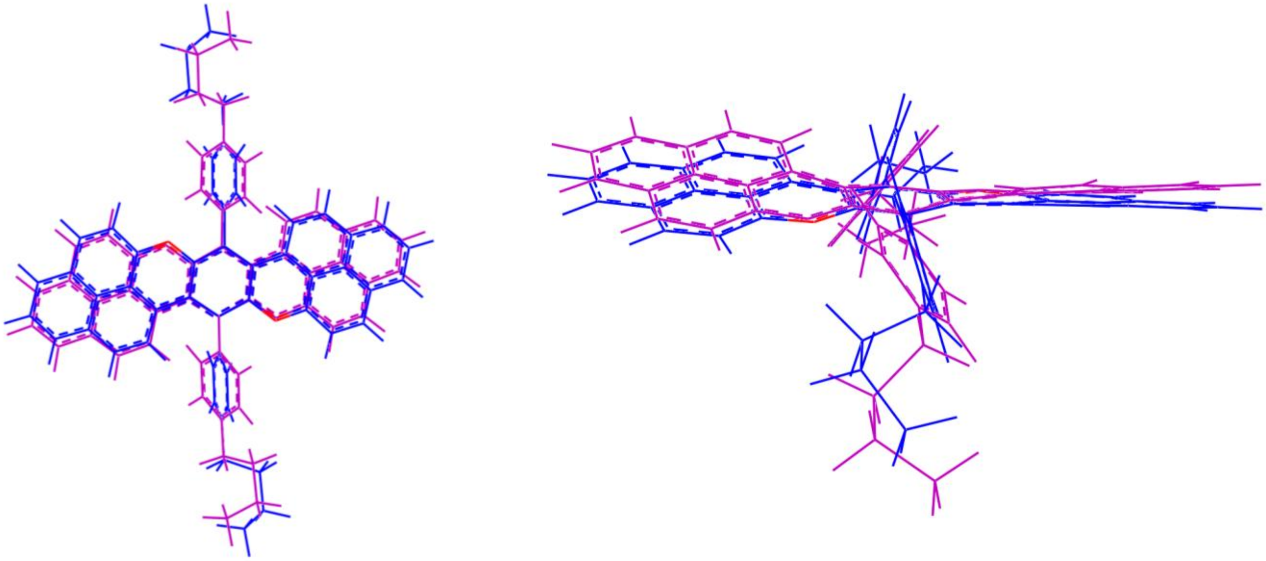

Figure S59: Geometry structure of 3[nBuPh] GS (blue) and opt S1 (purple). B3LYP-D3/6-31+G(d,p)/PCM(ACN).

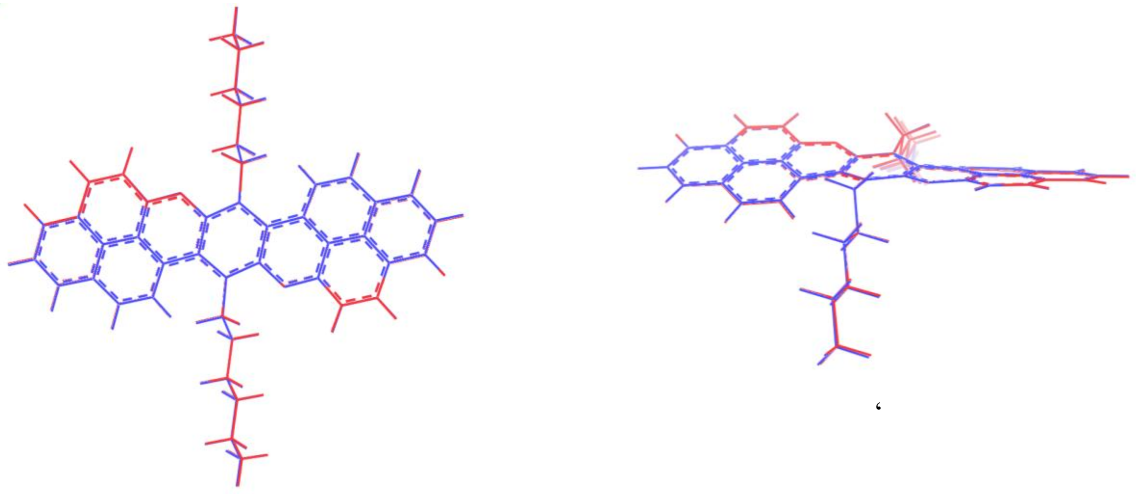

Figure S60: Geometry structure of 4[Hexyl] GS (blue) and opt S1 (red). B3LYP-D3/6-31+G(d,p)/PCM(ACN).

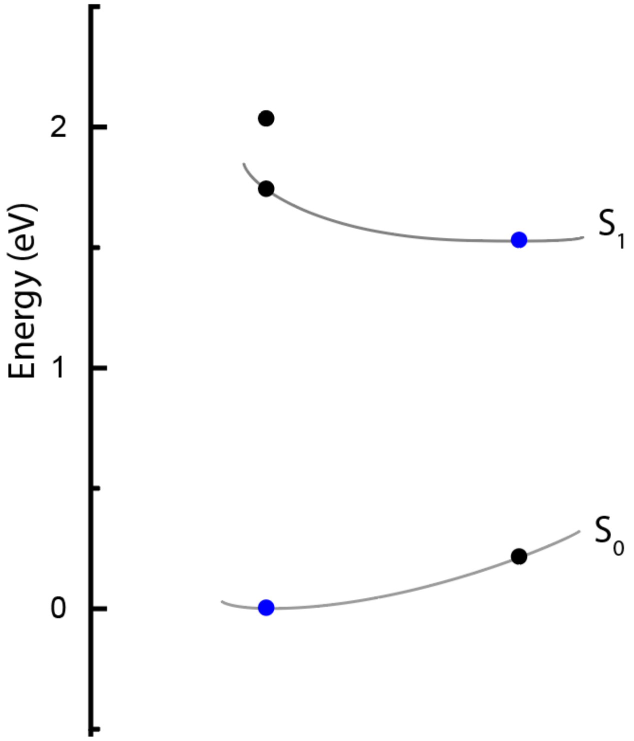

Figure S61: Potential energy curve (PEC) of 3[nBuPh].
Blue points are optimized minima, and black points are single-point energies calculated at the minimum geometries. Schematic representation of the PECs (grey lines) to indicate differences between the calculated minima geometries. B3LYP-D3/6-31+G(d,p)/PCM(ACN).

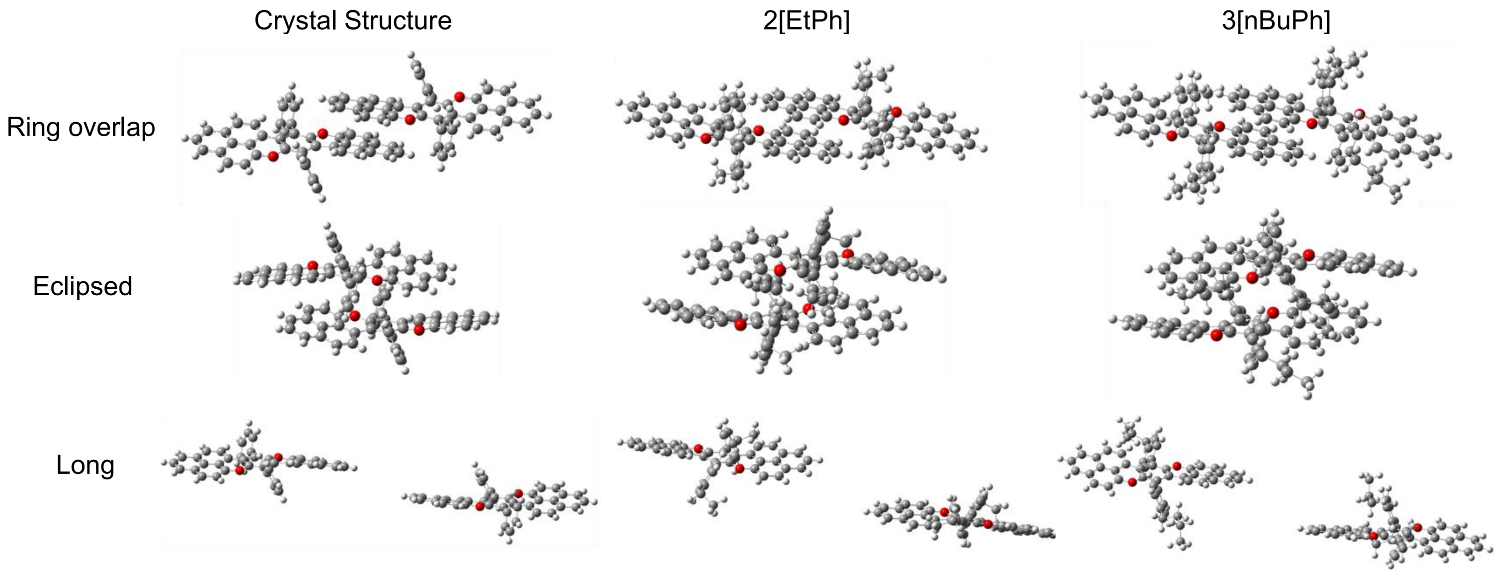

Figure S62: Three dimers cut from the 1[Ph] crystal structure and the optimized 2[EtPh] and 3[nBuPh] dimers. The long 2[EtPh] and 3[nBuPh] dimers had have the C-C intermolecular bond and the intermolecular dihedral constrained to not optimize to an eclipsed structure. B3LYP-D3/6-31+G(d,p)/PCM(ACN).

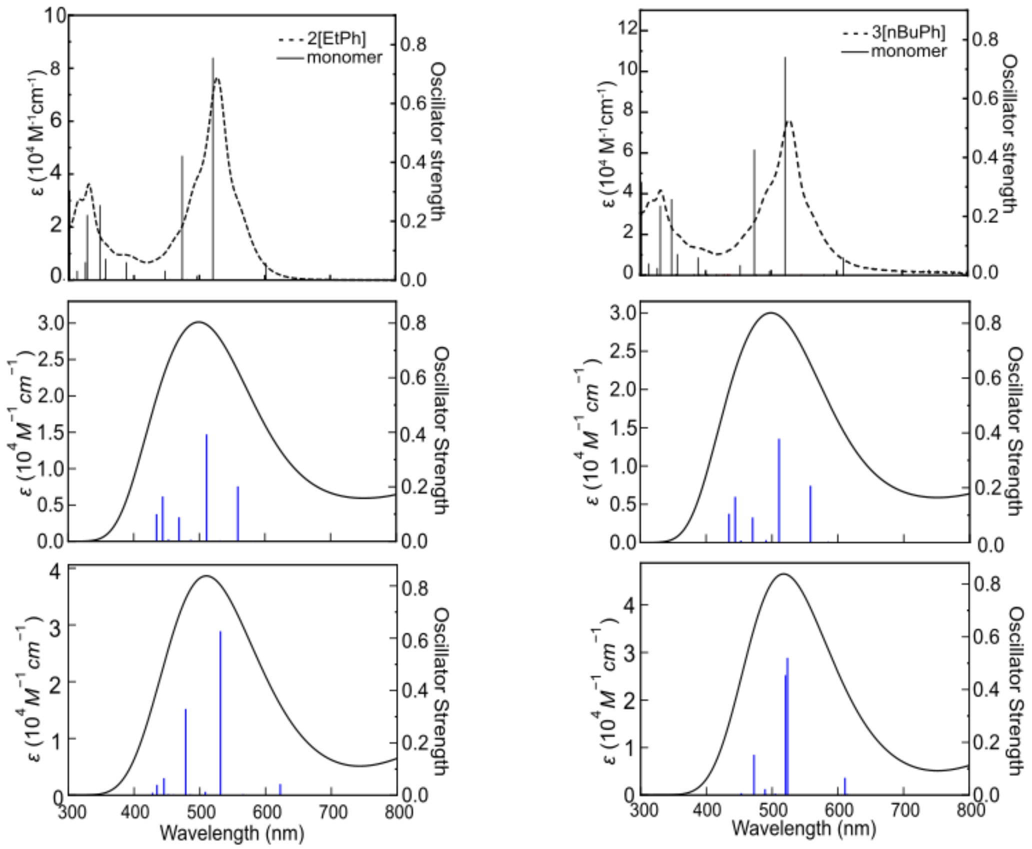

Figure S63: TDDFT of the 2[EtPh] and 3[nBuPh] monomers and dimers.
From top to bottom for each substitution: monomer, ring overlap dimer, and eclipsed dimer. B3LYP-D3/6-31G+(d,p)/PCM(ACN).

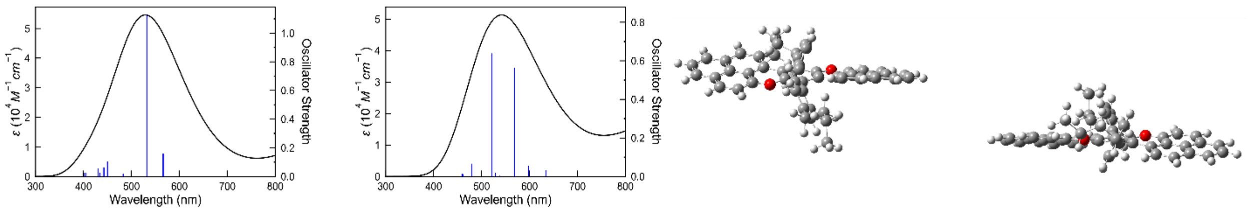

Figure S64: TDDFT of the crystal structure and 2[EtPh], and optimized structure of 3[nBuPh] (left to right) of the long dimer.
The 2[EtPh] and 3[nBuPh] dimers have the C-C intermolecular bond and the intermolecular dihedral constrained to not optimize to an eclipsed structure. THe 3[nBuPh] dimer gave an error in the TDDFT calculation. B3LYP-D3/6-31+G(d,p)/PCM(ACN).

Table S3: Basis set superposition error (BSSE) and corrected interaction energies of 2[EtPh] and 3[nBuPh] ring overlap and eclipsed dimers. B3LYP-D3/6-31+G(d,p)/PCM(ACN).

Dimer	BSSE	Corrected Interaction Energy (kcal/mol)
2[EtPh] overlapped ring	0.00934	9.17
2[EtPh] eclipsed	0.01833	0.66
3[nBuPh] overlapped ring	0.00936	8.79
3[nBuPh] eclipsed	0.01943	-2.61

Table S4: First 30 TDDFT singlet transitions of 2[EtPh] monomer at the ground state singlet geometry. B3LYP-D3/6-31+G/PCM(ACN).

State	Energy(eV)	Wavelength (nm)	Oscillator Strength (f)	Transitions
1	2.0597	601.94	0.0559	167 → 168 0.69868
2	2.166	572.42	0.0003	166 → 168 0.70443
3	2.381	520.72	0.7541	162 → 168 −0.10702<br>165 → 168 0.68671
4	2.4891	498.1	0.0001	164 → 168 0.70244
5	2.4974	496.46	0.0121	163 → 168 0.69926
6	2.6175	473.68	0.4206	162 → 168 0.68161<br>165 → 168 0.10876
7	2.6238	472.54	0.0008	167 → 169 0.69281
8	2.7703	447.54	0.03	166 → 169 0.70489
9	2.8938	428.44	0.0007	158 → 168 −0.12276<br>160 → 168 −0.21348<br>161 → 168 0.62153<br>165 → 169 −0.16134
10	2.9812	415.88	0.0006	160 → 168 −0.30942<br>162 → 169 −0.21743<br>165 → 169 0.58617
11	3.0834	402.1	0.0005	160 → 168 0.13869<br>161 → 168 0.2052<br>162 → 169 0.15947<br>163 → 169 0.59527<br>165 → 169 0.17222
12	3.0943	400.69	0.002	159 → 168 0.12985<br>164 → 169 0.67963

13	3.1789	390.02	0.0005	158	→	168	0.19086
				160	→	168	0.19636
				161	→	168	0.19046
				162	→	169	0.41479
				163	→	169	-0.34965
				165	→	169	0.25928
14	3.1916	388.47	0.0586	157	→	168	0.23809
				158	→	169	-0.10327
				159	→	168	0.57635
				160	→	169	-0.19041
				161	→	169	0.16163
				164	→	169	-0.16045
15	3.2483	381.69	0.0012	157	→	169	0.10294
				158	→	168	-0.10339
				160	→	168	-0.45174
				161	→	168	-0.15149
				162	→	169	0.47556
16	3.4774	356.54	0.0704	157	→	168	0.65001
				159	→	168	-0.23692
17	3.4972	354.52	0.0028	158	→	168	0.62508
				160	→	168	-0.25283
18	3.5641	347.87	0.253	159	→	168	−0.15442
				161	→	169	0.66748
19	3.774	328.52	0.2195	160	→	169	−0.23746
				167	→	170	0.64645
20	3.8065	325.72	0.0592	158	→	169	0.16149
				159	→	168	0.2135
				160	→	169	0.58116
				165	→	170	-0.13425
				167	→	170	0.24883
21	3.8813	319.44	0	157	→	169	0.13961
				158	→	168	0.13892
				159	→	169	0.51036
				166	→	170	-0.42858
22	3.9016	317.78	0	157	→	169	0.17033
				159	→	169	0.38191
				166	→	170	0.55262
23	3.9601	313.08	0.0299	158	→	169	0.49782
				159	→	168	0.10231
				165	→	170	0.47457
24	4.0246	308.07	0.0029	157	→	169	0.65084
				159	→	169	-0.2251

25	4.1089	301.74	0.3029	156<br>158<br>160<br>162<br>165	→<br>→<br>→<br>→<br>→	168<br>169<br>169<br>170<br>170	−0.12403<br>-0.43654<br>0.21057<br>−0.13076<br>0.45482
26	4.2353	292.74	0.0023	164	→	170	0.70251
27	4.2416	292.3	0.0725	163	→	170	0.69752
28	4.297	288.54	0.0716	156<br>162	→<br>→	168<br>170	0.61396<br>-0.29972
29	4.4182	280.62	0.5235	156<br>162<br>165<br>165<br>167	→<br>→<br>→<br>→<br>→	168<br>170<br>170<br>171<br>171	0.25185<br>0.59062<br>0.11658<br>0.11981<br>0.17615
30	4.4747	277.08	0	151<br>154<br>155	→<br>→<br>→	168<br>168<br>168	−0.12541<br>0.20696<br>0.63525

Table S5: First 30 TDDFT singlet transitions of 3[nBuPh] monomer at the ground state singlet geometry. B3LYP-D3/6-31+G/PCM(ACN).

State	Energy (eV)	Wavelength (nm)	Oscillator Strength (f)	Transitions			
1	2.0311	610.41	0.058	183	→	184	0.70152
2	2.1356	580.55	0.0004	182	→	184	0.70456
3	2.3787	521.22	0.7395	178<br>181	→	184<br>184	−0.10712<br>0.68922
4	2.4836	499.22	0.0001	180	→	184	0.70253
5	2.4917	497.59	0.0127	179	→	184	0.70011
6	2.596	477.6	0.0003	183	→	185	0.69579
7	2.6143	474.25	0.425	178<br>181	→<br>→	184<br>184	0.68228<br>0.10893
8	2.7389	452.68	0.0325	182	→	185	0.70491
9	2.894	428.41	0.0007	174<br>176<br>177<br>181	→<br>→<br>→<br>→	184<br>184<br>184<br>185	0.12319<br>0.21554<br>0.62033<br>-0.16297
10	2.9771	416.46	0.0008	176<br>178<br>181	→<br>→<br>→	184<br>185<br>185	0.30949<br>-0.22172<br>0.58439

11	3.0776	402.86	0.0006	176 → 184 −0.13<br>177 → 184 0.20587<br>178 → 185 0.14903<br>179 → 185 0.60641<br>181 → 185 0.17491
12	3.0885	401.43	0.0017	175 → 184 0.1211<br>180 → 185 0.68715
13	3.1768	390.28	0.0006	174 → 184 −0.18929<br>176 → 184 −0.19002<br>177 → 184 0.191<br>178 → 185 0.42709<br>179 → 185 −0.33685<br>181 → 185 0.2658
14	3.1914	388.5	0.0587	173 → 184 0.24616<br>174 → 185 -0.10382<br>175 → 184 0.57498<br>176 → 185 -0.19162<br>177 → 185 -0.16182<br>180 → 185 -0.15152
15	3.2447	382.11	0.0014	173 → 185 −0.10297<br>174 → 184 0.10671<br>176 → 184 0.45746<br>177 → 184 −0.1557<br>178 → 185 0.46591
16	3.4751	356.77	0.0703	173 → 184 0.64637<br>175 → 184 -0.24523
17	3.4967	354.58	0.0028	174 → 184 0.62468<br>176 → 184 -0.252
18	3.5633	347.95	0.2566	175 → 184 0.15223<br>177 → 185 0.66681
19	3.7484	330.76	0.2346	176 → 185 −0.1228<br>183 → 186 0.68093
20	3.8021	326.09	0.0228	174 → 185 0.16315<br>175 → 184 0.21859<br>176 → 185 0.61563<br>181 → 186 0.13546<br>183 → 186 0.12594
21	3.8593	321.26	0.0001	175 → 185 0.21491<br>182 → 186 0.66026

22	3.8922	318.54	0	173	→	185	0.22415
				174	→	184	0.1216
				175	→	185	0.59669
				176	→	184	0.13704
				182	→	186	-0.22893
23	3.9581	313.24	0.0382	174	→	185	0.49367
				175	→	184	0.10492
				181	→	186	-0.47883
24	4.0221	308.26	0.0031	173	→	185	0.64813
				175	→	185	-0.23399
25	4.1061	301.95	0.3156	166	→	184	−0.11849
				174	→	185	0.44209
				176	→	185	−0.21115
				178	→	186	−0.1314
				181	→	186	0.4499
26	4.176	296.89	0.0001	168	→	184	−0.12479
				170	→	184	0.16907
				172	→	184	0.66948
27	4.1894	295.95	0.0018	167	→	184	−0.10703
				169	→	184	0.11919
				171	→	184	0.68223
28	4.217	294.01	0	168	→	184	−0.23126
				170	→	184	0.62683
				172	→	184	-0.20199
29	4.2283	293.22	0.0019	169	→	184	−0.13102
				180	→	186	0.68294
30	4.2286	293.2	0.0063	167	→	184	−0.23498
				169	→	184	0.61714
				171	→	184	−0.13436
				179	→	186	0.12868
				180	→	186	0.14746

Table S6: First 30 TDDFT singlet transitions of 4[Hexyl] monomer at the ground state singlet geometry. B3LYP-D3/6-31+G/PCM(ACN).

State	Energy (eV)	Wavelength (nm)	Oscillator Strength (f)	Transitions
1	2.2042	562.49	0.1912	158 → 160 0.20195<br>159 → 160 0.67401
2	2.4899	497.95	1.0564	158 → 160 0.66888<br>159 → 160 -0.20364
3	2.7232	455.3	0.0005	156 → 160 −0.12963<br>158 → 161 0.29568<br>159 → 161 0.61972
4	2.8977	427.87	0.0009	153 → 160 −0.13767<br>156 → 160 0.2659<br>157 → 160 0.60758<br>158 → 161 -0.13993
5	3.0814	402.36	0.0123	154 → 160 0.33778<br>155 → 160 0.56594<br>156 → 161 0.18344<br>157 → 161 0.1277
6	3.0852	401.87	0.0001	156 → 160 0.3596<br>158 → 161 0.566<br>159 → 161 -0.19292
7	3.1543	393.07	0.0006	153 → 160 −0.22034<br>155 → 161 0.13003<br>156 → 160 0.42616<br>157 → 160 −0.33949<br>158 → 161 −0.22447<br>159 → 161 0.24459
8	3.3875	366.01	0.0905	154 → 160 0.55003<br>155 → 160 -0.39635<br>156 → 161 0.11495
9	3.4828	355.99	0.0092	153 → 160 0.62048<br>156 → 160 0.26529<br>158 → 161 -0.10357
10	3.5533	348.93	0.2643	154 → 160<br>157 → 161<br>−0.12963<br>0.29568<br>0.61972
11	3.7604	329.71	0.0151	153 → 161 −0.13381<br>154 → 160 −0.19774<br>155 → 160 −0.10794<br>156 → 161 0.62264<br>159 → 162 0.17601

12	3.8073	325.65	0.0015	153<br>154<br>155<br>156	→<br>→<br>→<br>→	160<br>161<br>161<br>160	0.17118<br>0.24862<br>0.60981<br>-0.14628
13	3.8995	317.95	0.5073	153<br>154<br>159	→<br>→<br>→	161<br>160<br>162	0.26521<br>0.12734<br>0.61659
14	3.9008	317.84	0.0005	152	→	160	0.69571
15	3.9493	313.94	0.0042	154<br>155	→<br>→	161<br>161	0.63309<br>-0.27787
16	4.0129	308.97	0.314	151<br>153<br>156<br>158<br>159	→<br>→<br>→<br>→<br>→	160<br>161<br>161<br>162<br>162	−0.1224<br>0.56812<br>0.18511<br>−0.25376<br>−0.21463
17	4.0466	306.39	0.0238	151<br>153	→<br>→	160<br>161	0.67785<br>0.16466
18	4.0837	303.61	0	145<br>150	→<br>→	160<br>160	0.10452<br>0.69215
19	4.0852	303.5	0.0001	149	→	160	0.69209
20	4.1991	295.26	0.0486	146<br>147<br>148<br>151<br>153<br>158	→<br>→<br>→<br>→<br>→<br>→	160<br>160<br>160<br>160<br>161<br>162	0.32618<br>-0.36189<br>0.27016<br>-0.11392<br>0.12762<br>0.36676
21	4.2531	291.51	0	147<br>148	→<br>→	160<br>160	0.41525<br>0.56576
22	4.2797	289.71	0.0502	146<br>147<br>148<br>158	→<br>→<br>→<br>→	160<br>160<br>160<br>162	0.30508<br>0.43056<br>-0.31237<br>0.31022
23	4.3583	284.48	0.2144	144<br>146<br>158<br>159	→<br>→<br>→<br>→	160<br>160<br>162<br>163	0.16019<br>0.51313<br>-0.38779<br>-0.14397
24	4.3739	283.46	0.0001	145<br>150	→<br>→	160<br>160	0.69041<br>-0.10177
25	4.3951	282.1	0.0308	144<br>158	→<br>→	160<br>162	0.6722<br>0.11932
26	4.4933	275.93	0.0033	152	→	161	0.69773

27	4.5967	269.73	0.0012	157 → 162 0.64612<br>159 → 166 -0.10059
28	4.6372	267.37	0	138 → 160 −0.10143<br>143 → 160 0.17007<br>151 → 161 0.65528
29	4.6641	265.83	0	137 → 161 −0.11031<br>138 → 160 −0.23671<br>141 → 160 0.19963<br>143 → 160 0.56188<br>151 → 161 −0.23476
30	4.6787	265	0.0001	145 → 161 0.10079<br>150 → 161 0.69144

Table S7: First 20 TDDFT singlet transitions of singlet 2[EtPh] overlapping ring dimer. B3LYP-D3/6-31+G/PCM(ACN).

Excited State	Energy (eV)	Wavelength (nm)	Oscillator Strength (f)	Transitions
1	0.8006	1548.55	1.3766	335 → 336 0.743
2	1.1091	1117.87	0	335 → 336 0.743<br>335 → 337 0.714
3	1.5392	805.53	0.1018	335 → 336 0.743<br>335 → 337 0.714<br>335 → 338 0.705
4	2.0929	592.39	0	335 → 336 0.743<br>335 → 337 0.714<br>335 → 338 0.705<br>335 → 339 0.698
5	2.1172	585.6	0.0035	335 → 336 0.743<br>335 → 337 0.714<br>335 → 338 0.705<br>335 → 339 0.698<br>334 → 336 0.184<br>335 → 340 0.670
6	2.2188	558.78	0.2017	335 → 336 0.743<br>335 → 337 0.714<br>335 → 338 0.705<br>335 → 339 0.698<br>334 → 336 0.184<br>335 → 340 0.670<br>332 → 336 0.124<br>334 → 336 0.656<br>335 → 340 -0.179

7	2.3307	531.95	0.0037	335 → 336 0.743<br>335 → 337 0.714<br>335 → 338 0.705<br>335 → 339 0.698<br>334 → 336 0.184<br>335 → 340 0.670<br>332 → 336 0.124<br>334 → 336 0.656<br>335 → 340 -0.179<br>331 → 336 -0.225<br>332 → 337 -0.123<br>333 → 336 0.628<br>334 → 337 -0.167
8	2.4262	511.03	0.3925	335 → 336 0.743<br>335 → 337 0.714<br>335 → 338 0.705<br>335 → 339 0.698<br>334 → 336 0.184<br>335 → 340 0.670<br>332 → 336 0.124<br>334 → 336 0.656<br>335 → 340 -0.179<br>331 → 336 -0.225<br>332 → 337 -0.123<br>333 → 336 0.628<br>334 → 337 -0.167<br>331 → 336 0.135<br>331 → 337 0.126<br>332 → 336 0.649<br>333 → 337 -0.113<br>334 → 336 -0.132

				335	→	336	0.743
				335	→	337	0.714
				335	→	338	0.705
				335	→	339	0.698
				334	→	336	0.184
				335	→	340	0.670
				332	→	336	0.124
				334	→	336	0.656
				335	→	340	-0.179
				331	→	336	-0.225
				332	→	337	-0.123
9	2.5192	492.15	0.0019	333	→	336	0.628
				334	→	337	-0.167
				331	→	336	0.135
				331	→	337	0.126
				332	→	336	0.649
				333	→	337	-0.113
				334	→	336	-0.132
				330	→	336	0.110
				331	→	336	0.596
				332	→	336	-0.116
				332	→	337	0.102
				333	→	336	0.266
				335	→	336	0.743
				335	→	337	0.714
				335	→	338	0.705
				335	→	339	0.698
				334	→	336	0.184
				335	→	340	0.670
				332	→	336	0.124
				334	→	336	0.656
				335	→	340	-0.179
				331	→	336	-0.225
				332	→	337	-0.123
				333	→	336	0.628
10	2.5458	487.01	0.0058	334	→	337	-0.167
				331	→	336	0.135
				331	→	337	0.126
				332	→	336	0.649
				333	→	337	-0.113
				334	→	336	-0.132
				330	→	336	0.110
				331	→	336	0.596
				332	→	336	-0.116
				332	→	337	0.102
				333	→	336	0.266
				329	→	337	0.153
				330	→	336	0.670

11	2.5535	485.55	0.0005	335 → 336 0.743<br>335 → 337 0.714<br>335 → 338 0.705<br>335 → 339 0.698<br>334 → 336 0.184<br>335 → 340 0.670<br>332 → 336 0.124<br>334 → 336 0.656<br>335 → 340 -0.179<br>331 → 336 -0.225<br>332 → 337 -0.123<br>333 → 336 0.628<br>334 → 337 -0.167<br>331 → 336 0.135<br>331 → 337 0.126<br>332 → 336 0.649<br>333 → 337 -0.113<br>334 → 336 -0.132<br>330 → 336 0.110<br>331 → 336 0.596<br>332 → 336 -0.116<br>332 → 337 0.102<br>333 → 336 0.266<br>329 → 337 0.153<br>330 → 336 0.670<br>329 → 336 0.677<br>330 → 337 0.152

12	2.5896	478.77	0.0004	335 → 336 0.743<br>335 → 337 0.714<br>335 → 338 0.705<br>335 → 339 0.698<br>334 → 336 0.184<br>335 → 340 0.670<br>332 → 336 0.124<br>334 → 336 0.656<br>335 → 340 -0.179<br>331 → 336 -0.225<br>332 → 337 -0.123<br>333 → 336 0.628<br>334 → 337 -0.167<br>331 → 336 0.135<br>331 → 337 0.126<br>332 → 336 0.649<br>333 → 337 -0.113<br>334 → 336 -0.132<br>330 → 336 0.110<br>331 → 336 0.596<br>332 → 336 -0.116<br>332 → 337 0.102<br>333 → 336 0.266<br>329 → 337 0.153<br>330 → 336 0.670<br>329 → 336 0.677<br>330 → 337 0.152<br>331 → 336 -0.147<br>333 → 336 0.125<br>334 → 337 0.664

13	2.6432	469.07	0.0886	335 → 336 0.743<br>335 → 337 0.714<br>335 → 338 0.705<br>335 → 339 0.698<br>334 → 336 0.184<br>335 → 340 0.670<br>332 → 336 0.124<br>334 → 336 0.656<br>335 → 340 -0.179<br>331 → 336 -0.225<br>332 → 337 -0.123<br>333 → 336 0.628<br>334 → 337 -0.167<br>331 → 336 0.135<br>331 → 337 0.126<br>332 → 336 0.649<br>333 → 337 -0.113<br>334 → 336 -0.132<br>330 → 336 0.110<br>331 → 336 0.596<br>332 → 336 -0.116<br>332 → 337 0.102<br>333 → 336 0.266<br>329 → 337 0.153<br>330 → 336 0.670<br>329 → 336 0.677<br>330 → 337 0.152<br>331 → 336 -0.147<br>333 → 336 0.125<br>334 → 337 0.664<br>327 → 337 -0.104<br>328 → 336 0.667<br>333 → 337 0.122

14	2.723	455.33	0.0001	335	→	336	0.743
				335	→	337	0.714
				335	→	338	0.705
				335	→	339	0.698
				334	→	336	0.184
				335	→	340	0.670
				332	→	336	0.124
				334	→	336	0.656
				335	→	340	-0.179
				331	→	336	-0.225
				332	→	337	-0.123
				333	→	336	0.628
				334	→	337	-0.167
				331	→	336	0.135
				331	→	337	0.126
				332	→	336	0.649
				333	→	337	-0.113
				334	→	336	-0.132
				330	→	336	0.110
				331	→	336	0.596
				332	→	336	-0.116
				332	→	337	0.102
				333	→	336	0.266
				329	→	337	0.153
				330	→	336	0.670
				329	→	336	0.677
				330	→	337	0.152
				331	→	336	-0.147
				333	→	336	0.125
				334	→	337	0.664
				327	→	337	-0.104
				328	→	336	0.667
				333	→	337	0.122
				327	→	336	0.667
				328	→	337	-0.150

15	2.7373	452.94	0.0071	335	→	336	0.743
				335	→	337	0.714
				335	→	338	0.705
				335	→	339	0.698
				334	→	336	0.184
				335	→	340	0.670
				332	→	336	0.124
				334	→	336	0.656
				335	→	340	-0.179
				331	→	336	-0.225
				332	→	337	-0.123
				333	→	336	0.628
				334	→	337	-0.167
				331	→	336	0.135
				331	→	337	0.126
				332	→	336	0.649
				333	→	337	-0.113
				334	→	336	-0.132
				330	→	336	0.110
				331	→	336	0.596
				332	→	336	-0.116
				332	→	337	0.102
				333	→	336	0.266
				329	→	337	0.153
				330	→	336	0.670
				329	→	336	0.677
				330	→	337	0.152
				331	→	336	-0.147
				333	→	336	0.125
				334	→	337	0.664
				327	→	337	-0.104
				328	→	336	0.667
				333	→	337	0.122
				327	→	336	0.667
				328	→	337	-0.150
				325	→	336	0.124
				328	→	336	-0.148
				331	→	337	-0.166
				332	→	336	0.148
				333	→	337	0.588

16	2.7914	444.16	0.1648	335	→	336	0.743
				335	→	337	0.714
				335	→	338	0.705
				335	→	339	0.698
				334	→	336	0.184
				335	→	340	0.670
				332	→	336	0.124
				334	→	336	0.656
				335	→	340	-0.179
				331	→	336	-0.225
				332	→	337	-0.123
				333	→	336	0.628
				334	→	337	-0.167
				331	→	336	0.135
				331	→	337	0.126
				332	→	336	0.649
				333	→	337	-0.113
				334	→	336	-0.132
				330	→	336	0.110
				331	→	336	0.596
				332	→	336	-0.116
				332	→	337	0.102
				333	→	336	0.266
				329	→	337	0.153
				330	→	336	0.670
				329	→	336	0.677
				330	→	337	0.152
				331	→	336	-0.147
				333	→	336	0.125
				334	→	337	0.664
				327	→	337	-0.104
				328	→	336	0.667
				333	→	337	0.122
				327	→	336	0.667
				328	→	337	-0.150
				325	→	336	0.124
				328	→	336	-0.148
				331	→	337	-0.166
				332	→	336	0.148
				333	→	337	0.588
				325	→	336	-0.239
				326	→	336	0.592
				335	→	341	-0.223

17	2.8018	442.52	0.0027	335 → 336 0.743
				335 → 337 0.714
				335 → 338 0.705
				335 → 339 0.698
				334 → 336 0.184
				335 → 340 0.670
				332 → 336 0.124
				334 → 336 0.656
				335 → 340 -0.179
				331 → 336 -0.225
				332 → 337 -0.123
				333 → 336 0.628
				334 → 337 -0.167
				331 → 336 0.135
				331 → 337 0.126
				332 → 336 0.649
				333 → 337 -0.113
				334 → 336 -0.132
				330 → 336 0.110
				331 → 336 0.596
				332 → 336 -0.116
				332 → 337 0.102
				333 → 336 0.266
				329 → 337 0.153
				330 → 336 0.670
				329 → 336 0.677
				330 → 337 0.152
				331 → 336 -0.147
				333 → 336 0.125
				334 → 337 0.664
				327 → 337 -0.104
				328 → 336 0.667
				333 → 337 0.122
				327 → 336 0.667
				328 → 337 -0.150
				325 → 336 0.124
				328 → 336 -0.148
				331 → 337 -0.166
				332 → 336 0.148
				333 → 337 0.588
				325 → 336 -0.239
				326 → 336 0.592
				335 → 341 -0.223
				331 → 336 -0.144
				331 → 337 0.140
				332 → 337 0.644

18	2.8512	434.85	0.0999	335 → 336 0.743<br>335 → 337 0.714<br>335 → 338 0.705<br>335 → 339 0.698<br>334 → 336 0.184<br>335 → 340 0.670<br>332 → 336 0.124<br>334 → 336 0.656<br>335 → 340 -0.179<br>331 → 336 -0.225<br>332 → 337 -0.123<br>333 → 336 0.628<br>334 → 337 -0.167<br>331 → 336 0.135<br>331 → 337 0.126<br>332 → 336 0.649<br>333 → 337 -0.113<br>334 → 336 -0.132<br>330 → 336 0.110<br>331 → 336 0.596<br>332 → 336 -0.116<br>332 → 337 0.102<br>333 → 336 0.266<br>329 → 337 0.153<br>330 → 336 0.670<br>329 → 336 0.677<br>330 → 337 0.152<br>331 → 336 -0.147<br>333 → 336 0.125<br>334 → 337 0.664<br>327 → 337 -0.104<br>328 → 336 0.667<br>333 → 337 0.122<br>327 → 336 0.667<br>328 → 337 -0.150<br>325 → 336 0.124<br>328 → 336 -0.148<br>331 → 337 -0.166<br>332 → 336 0.148<br>333 → 337 0.588<br>325 → 336 -0.239<br>326 → 336 0.592<br>335 → 341 -0.223<br>331 → 336 -0.144<br>331 → 337 0.140<br>332 → 337 0.644<br>325 → 336 0.523<br>331 → 337 0.167<br>333 → 337 -0.128<br>334 → 338 0.136<br>335 → 341 -0.343

19	2.889	429.16	0.0011	335 → 336 0.743<br>335 → 337 0.714<br>335 → 338 0.705<br>335 → 339 0.698<br>334 → 336 0.184<br>335 → 340 0.670<br>332 → 336 0.124<br>334 → 336 0.656<br>335 → 340 -0.179<br>331 → 336 -0.225<br>332 → 337 -0.123<br>333 → 336 0.628<br>334 → 337 -0.167<br>331 → 336 0.135<br>331 → 337 0.126<br>332 → 336 0.649<br>333 → 337 -0.113<br>334 → 336 -0.132<br>330 → 336 0.110<br>331 → 336 0.596<br>332 → 336 -0.116<br>332 → 337 0.102<br>333 → 336 0.266<br>329 → 337 0.153<br>330 → 336 0.670<br>329 → 336 0.677<br>330 → 337 0.152<br>331 → 336 -0.147<br>333 → 336 0.125<br>334 → 337 0.664<br>327 → 337 -0.104<br>328 → 336 0.667<br>333 → 337 0.122<br>327 → 336 0.667<br>328 → 337 -0.150<br>325 → 336 0.124<br>328 → 336 -0.148<br>331 → 337 -0.166<br>332 → 336 0.148<br>333 → 337 0.588<br>325 → 336 -0.239<br>326 → 336 0.592<br>335 → 341 -0.223<br>331 → 336 -0.144<br>331 → 337 0.140<br>332 → 337 0.644<br>325 → 336 0.523<br>331 → 337 0.167<br>333 → 337 -0.128<br>334 → 338 0.136<br>335 → 341 -0.343

				324 → 336 0.540<br>324 → 337 -0.142<br>335 → 342 -0.394
20	2.8905	428.94	0.0006	335 → 336 0.743<br>335 → 337 0.714<br>335 → 338 0.705<br>335 → 339 0.698<br>334 → 336 0.184<br>335 → 340 0.670<br>332 → 336 0.124<br>334 → 336 0.656<br>335 → 340 -0.179<br>331 → 336 -0.225<br>332 → 337 -0.123<br>333 → 336 0.628<br>334 → 337 -0.167<br>331 → 336 0.135<br>331 → 337 0.126<br>332 → 336 0.649<br>333 → 337 -0.113<br>334 → 336 -0.132<br>330 → 336 0.110<br>331 → 336 0.596<br>332 → 336 -0.116<br>332 → 337 0.102<br>333 → 336 0.266<br>329 → 337 0.153<br>330 → 336 0.670<br>329 → 336 0.677<br>330 → 337 0.152<br>331 → 336 -0.147<br>333 → 336 0.125<br>334 → 337 0.664<br>327 → 337 -0.104<br>328 → 336 0.667<br>333 → 337 0.122<br>327 → 336 0.667<br>328 → 337 -0.150<br>325 → 336 0.124<br>328 → 336 -0.148<br>331 → 337 -0.166<br>332 → 336 0.148<br>333 → 337 0.588<br>325 → 336 -0.239<br>326 → 336 0.592<br>335 → 341 -0.223<br>331 → 336 -0.144<br>331 → 337 0.140<br>332 → 337 0.644<br>325 → 336 0.523<br>331 → 337 0.167

				Transitions
				333 → 337 -0.128<br>334 → 338 0.136<br>335 → 341 -0.343<br>324 → 336 0.540<br>324 → 337 -0.142<br>335 → 342 -0.394<br>323 → 336 0.237<br>324 → 336 0.354<br>324 → 337 -0.110<br>335 → 342 0.495<br>335 → 343 0.134<br>335 → 346 -0.104

Table S8: First 20 TDDFT singlet transitions of singlet 3[nBuPh] overlapping ring dimer. B3LYP-D3/6-31+G/PCM(ACN).

Excited State	Energy (eV)	Wavelength (nm)	Oscillator Strength (f)	Transitions
1	0.8004	1548.96	1.3736	367 → 368 0.743
2	1.1093	1117.66	0	367 → 368 0.743<br>367 → 369 0.714
3	1.5383	805.99	0.1005	367 → 368 0.743<br>367 → 369 0.714<br>367 → 370 0.705
4	2.0937	592.18	0	367 → 368 0.743<br>367 → 369 0.714<br>367 → 370 0.705<br>367 → 371 0.698
5	2.1173	585.57	0.0036	367 → 368 0.743<br>367 → 369 0.714<br>367 → 370 0.705<br>367 → 371 0.698<br>366 → 368 0.180<br>367 → 372 0.671
6	2.2202	558.44	0.2072	367 → 368 0.743<br>367 → 369 0.714<br>367 → 370 0.705<br>367 → 371 0.698<br>366 → 368 0.180<br>367 → 372 0.671<br>364 → 368 0.124<br>366 → 368 0.657<br>367 → 372 -0.175

7	2.3332	531.39	0.001	367 → 368 0.743<br>367 → 369 0.714<br>367 → 370 0.705<br>367 → 371 0.698<br>366 → 368 0.180<br>367 → 372 0.671<br>364 → 368 0.124<br>366 → 368 0.657<br>367 → 372 -0.175<br>363 → 368 -0.234<br>364 → 369 -0.124<br>365 → 368 0.629<br>366 → 369 -0.166
8	2.4274	510.77	0.3788	367 → 368 0.743<br>367 → 369 0.714<br>367 → 370 0.705<br>367 → 371 0.698<br>366 → 368 0.180<br>367 → 372 0.671<br>364 → 368 0.124<br>366 → 368 0.657<br>367 → 372 -0.175<br>363 → 368 -0.234<br>364 → 369 -0.124<br>365 → 368 0.629<br>366 → 369 -0.166<br>363 → 369 0.128<br>364 → 368 0.662<br>365 → 369 -0.112<br>366 → 368 -0.130
9	2.5186	492.28	0.0002	367 → 368 0.743<br>367 → 369 0.714<br>367 → 370 0.705<br>367 → 371 0.698<br>366 → 368 0.180<br>367 → 372 0.671<br>364 → 368 0.124<br>366 → 368 0.657<br>367 → 372 -0.175<br>363 → 368 -0.234<br>364 → 369 -0.124<br>365 → 368 0.629<br>366 → 369 -0.166<br>363 → 369 0.128<br>364 → 368 0.662<br>365 → 369 -0.112<br>366 → 368 -0.130<br>361 → 368 0.255<br>363 → 368 0.566<br>365 → 368 0.251

10	2.5251	491	0.0091	367 → 368 0.743<br>367 → 369 0.714<br>367 → 370 0.705<br>367 → 371 0.698<br>366 → 368 0.180<br>367 → 372 0.671<br>364 → 368 0.124<br>366 → 368 0.657<br>367 → 372 -0.175<br>363 → 368 -0.234<br>364 → 369 -0.124<br>365 → 368 0.629<br>366 → 369 -0.166<br>363 → 369 0.128<br>364 → 368 0.662<br>365 → 369 -0.112<br>366 → 368 -0.130<br>361 → 368 0.255<br>363 → 368 0.566<br>365 → 368 0.251<br>361 → 369 -0.161<br>362 → 368 0.676
11	2.5313	489.8	0.0003	367 → 368 0.743<br>367 → 369 0.714<br>367 → 370 0.705<br>367 → 371 0.698<br>366 → 368 0.180<br>367 → 372 0.671<br>364 → 368 0.124<br>366 → 368 0.657<br>367 → 372 -0.175<br>363 → 368 -0.234<br>364 → 369 -0.124<br>365 → 368 0.629<br>366 → 369 -0.166<br>363 → 369 0.128<br>364 → 368 0.662<br>365 → 369 -0.112<br>366 → 368 -0.130<br>361 → 368 0.255<br>363 → 368 0.566<br>365 → 368 0.251<br>361 → 369 -0.161<br>362 → 368 0.676<br>361 → 368 0.634<br>362 → 369 -0.139<br>363 → 368 -0.234

12	2.5903	478.66	0	367 → 368 0.743<br>367 → 369 0.714<br>367 → 370 0.705<br>367 → 371 0.698<br>366 → 368 0.180<br>367 → 372 0.671<br>364 → 368 0.124<br>366 → 368 0.657<br>367 → 372 -0.175<br>363 → 368 -0.234<br>364 → 369 -0.124<br>365 → 368 0.629<br>366 → 369 -0.166<br>363 → 369 0.128<br>364 → 368 0.662<br>365 → 369 -0.112<br>366 → 368 -0.130<br>361 → 368 0.255<br>363 → 368 0.566<br>365 → 368 0.251<br>361 → 369 -0.161<br>362 → 368 0.676<br>361 → 368 0.634<br>362 → 369 -0.139<br>363 → 368 -0.234<br>363 → 368 -0.140<br>365 → 368 0.127<br>366 → 369 0.664

13	2.6341	470.68	0.0916	367	→	368	0.743
				367	→	369	0.714
				367	→	370	0.705
				367	→	371	0.698
				366	→	368	0.180
				367	→	372	0.671
				364	→	368	0.124
				366	→	368	0.657
				367	→	372	-0.175
				363	→	368	-0.234
				364	→	369	-0.124
				365	→	368	0.629
				366	→	369	-0.166
				363	→	369	0.128
				364	→	368	0.662
				365	→	369	-0.112
				366	→	368	-0.130
				361	→	368	0.255
				363	→	368	0.566
				365	→	368	0.251
				361	→	369	-0.161
				362	→	368	0.676
				361	→	368	0.634
				362	→	369	-0.139
				363	→	368	-0.234
				363	→	368	-0.140
				365	→	368	0.127
				366	→	369	0.664
				359	→	369	0.108
				360	→	368	0.672
				365	→	369	0.104

14	2.7126	457.07	0.0001	367 → 368 0.743<br>367 → 369 0.714<br>367 → 370 0.705<br>367 → 371 0.698<br>366 → 368 0.180<br>367 → 372 0.671<br>364 → 368 0.124<br>366 → 368 0.657<br>367 → 372 -0.175<br>363 → 368 -0.234<br>364 → 369 -0.124<br>365 → 368 0.629<br>366 → 369 -0.166<br>363 → 369 0.128<br>364 → 368 0.662<br>365 → 369 -0.112<br>366 → 368 -0.130<br>361 → 368 0.255<br>363 → 368 0.566<br>365 → 368 0.251<br>361 → 369 -0.161<br>362 → 368 0.676<br>361 → 368 0.634<br>362 → 369 -0.139<br>363 → 368 -0.234<br>363 → 368 -0.140<br>365 → 368 0.127<br>366 → 369 0.664<br>359 → 369 0.108<br>360 → 368 0.672<br>365 → 369 0.104<br>359 → 368 0.665<br>360 → 369 0.151

15	2.7384	452.76	0.0078	367 → 368 0.743
				367 → 369 0.714
				367 → 370 0.705
				367 → 371 0.698
				366 → 368 0.180
				367 → 372 0.671
				364 → 368 0.124
				366 → 368 0.657
				367 → 372 -0.175
				363 → 368 -0.234
				364 → 369 -0.124
				365 → 368 0.629
				366 → 369 -0.166
				363 → 369 0.128
				364 → 368 0.662
				365 → 369 -0.112
				366 → 368 -0.130
				361 → 368 0.255
				363 → 368 0.566
				365 → 368 0.251
				361 → 369 -0.161
				362 → 368 0.676
				361 → 368 0.634
				362 → 369 -0.139
				363 → 368 -0.234
				363 → 368 -0.140
				365 → 368 0.127
				366 → 369 0.664
				359 → 369 0.108
				360 → 368 0.672
				365 → 369 0.104
				359 → 368 0.665
				360 → 369 0.151
				357 → 368 0.130
				359 → 369 -0.101
				360 → 368 -0.126
				363 → 369 -0.177
				364 → 368 0.148
				365 → 369 0.590

16	2.791	444.22	0.1669	367 → 368 0.743<br>367 → 369 0.714<br>367 → 370 0.705<br>367 → 371 0.698<br>366 → 368 0.180<br>367 → 372 0.671<br>364 → 368 0.124<br>366 → 368 0.657<br>367 → 372 -0.175<br>363 → 368 -0.234<br>364 → 369 -0.124<br>365 → 368 0.629<br>366 → 369 -0.166<br>363 → 369 0.128<br>364 → 368 0.662<br>365 → 369 -0.112<br>366 → 368 -0.130<br>361 → 368 0.255<br>363 → 368 0.566<br>365 → 368 0.251<br>361 → 369 -0.161<br>362 → 368 0.676<br>361 → 368 0.634<br>362 → 369 -0.139<br>363 → 368 -0.234<br>363 → 368 -0.140<br>365 → 368 0.127<br>366 → 369 0.664<br>359 → 369 0.108<br>360 → 368 0.672<br>365 → 369 0.104<br>359 → 368 0.665<br>360 → 369 0.151<br>357 → 368 0.130<br>359 → 369 -0.101<br>360 → 368 -0.126<br>363 → 369 -0.177<br>364 → 368 0.148<br>365 → 369 0.590<br>357 → 368 -0.242<br>358 → 368 0.591<br>365 → 369 0.108<br>367 → 373 -0.220

17	2.8038	442.21	0.0006	367	→	368	0.743
				367	→	369	0.714
				367	→	370	0.705
				367	→	371	0.698
				366	→	368	0.180
				367	→	372	0.671
				364	→	368	0.124
				366	→	368	0.657
				367	→	372	-0.175
				363	→	368	-0.234
				364	→	369	-0.124
				365	→	368	0.629
				366	→	369	-0.166
				363	→	369	0.128
				364	→	368	0.662
				365	→	369	-0.112
				366	→	368	-0.130
				361	→	368	0.255
				363	→	368	0.566
				365	→	368	0.251
				361	→	369	-0.161
				362	→	368	0.676
				361	→	368	0.634
				362	→	369	-0.139
				363	→	368	-0.234
				363	→	368	-0.140
				365	→	368	0.127
				366	→	369	0.664
				359	→	369	0.108
				360	→	368	0.672
				365	→	369	0.104
				359	→	368	0.665
				360	→	369	0.151
				357	→	368	0.130
				359	→	369	-0.101
				360	→	368	-0.126
				363	→	369	-0.177
				364	→	368	0.148
				365	→	369	0.590
				357	→	368	-0.242
				358	→	368	0.591
				365	→	369	0.108
				367	→	373	-0.220
				363	→	368	-0.145
				364	→	369	0.659

18	2.8514	434.83	0.1052	367 → 368 0.743<br>367 → 369 0.714<br>367 → 370 0.705<br>367 → 371 0.698<br>366 → 368 0.180<br>367 → 372 0.671<br>364 → 368 0.124<br>366 → 368 0.657<br>367 → 372 -0.175<br>363 → 368 -0.234<br>364 → 369 -0.124<br>365 → 368 0.629<br>366 → 369 -0.166<br>363 → 369 0.128<br>364 → 368 0.662<br>365 → 369 -0.112<br>366 → 368 -0.130<br>361 → 368 0.255<br>363 → 368 0.566<br>365 → 368 0.251<br>361 → 369 -0.161<br>362 → 368 0.676<br>361 → 368 0.634<br>362 → 369 -0.139<br>363 → 368 -0.234<br>363 → 368 -0.140<br>365 → 368 0.127<br>366 → 369 0.664<br>359 → 369 0.108<br>360 → 368 0.672<br>365 → 369 0.104<br>359 → 368 0.665<br>360 → 369 0.151<br>357 → 368 0.130<br>359 → 369 -0.101<br>360 → 368 -0.126<br>363 → 369 -0.177<br>364 → 368 0.148<br>365 → 369 0.590<br>357 → 368 -0.242<br>358 → 368 0.591<br>365 → 369 0.108<br>367 → 373 -0.220<br>363 → 368 -0.145<br>364 → 369 0.659<br>357 → 368 0.523<br>363 → 369 0.164<br>365 → 369 -0.130<br>366 → 370 0.135<br>367 → 373 -0.340<br>367 → 376 -0.101

19	2.8894	429.09	0.0001	367 → 368 0.743<br>367 → 369 0.714<br>367 → 370 0.705<br>367 → 371 0.698<br>366 → 368 0.180<br>367 → 372 0.671<br>364 → 368 0.124<br>366 → 368 0.657<br>367 → 372 -0.175<br>363 → 368 -0.234<br>364 → 369 -0.124<br>365 → 368 0.629<br>366 → 369 -0.166<br>363 → 369 0.128<br>364 → 368 0.662<br>365 → 369 -0.112<br>366 → 368 -0.130<br>361 → 368 0.255<br>363 → 368 0.566<br>365 → 368 0.251<br>361 → 369 -0.161<br>362 → 368 0.676<br>361 → 368 0.634<br>362 → 369 -0.139<br>363 → 368 -0.234<br>363 → 368 -0.140<br>365 → 368 0.127<br>366 → 369 0.664<br>359 → 369 0.108<br>360 → 368 0.672<br>365 → 369 0.104<br>359 → 368 0.665<br>360 → 369 0.151<br>357 → 368 0.130<br>359 → 369 -0.101<br>360 → 368 -0.126<br>363 → 369 -0.177<br>364 → 368 0.148<br>365 → 369 0.590<br>357 → 368 -0.242<br>358 → 368 0.591<br>365 → 369 0.108<br>367 → 373 -0.220<br>363 → 368 -0.145<br>364 → 369 0.659<br>357 → 368 0.523<br>363 → 369 0.164<br>365 → 369 -0.130<br>366 → 370 0.135<br>367 → 373 -0.340<br>367 → 376 -0.101

				355 → 368 -0.159<br>356 → 368 0.167<br>367 → 374 0.618<br>367 → 375 0.155<br>367 → 378 -0.125
20	2.8939	428.43	0.001	367 → 368 0.743<br>367 → 369 0.714<br>367 → 370 0.705<br>367 → 371 0.698<br>366 → 368 0.180<br>367 → 372 0.671<br>364 → 368 0.124<br>366 → 368 0.657<br>367 → 372 -0.175<br>363 → 368 -0.234<br>364 → 369 -0.124<br>365 → 368 0.629<br>366 → 369 -0.166<br>363 → 369 0.128<br>364 → 368 0.662<br>365 → 369 -0.112<br>366 → 368 -0.130<br>361 → 368 0.255<br>363 → 368 0.566<br>365 → 368 0.251<br>361 → 369 -0.161<br>362 → 368 0.676<br>361 → 368 0.634<br>362 → 369 -0.139<br>363 → 368 -0.234<br>363 → 368 -0.140<br>365 → 368 0.127<br>366 → 369 0.664<br>359 → 369 0.108<br>360 → 368 0.672<br>365 → 369 0.104<br>359 → 368 0.665<br>360 → 369 0.151<br>357 → 368 0.130<br>359 → 369 -0.101<br>360 → 368 -0.126<br>363 → 369 -0.177<br>364 → 368 0.148<br>365 → 369 0.590<br>357 → 368 -0.242<br>358 → 368 0.591<br>365 → 369 0.108<br>367 → 373 -0.220<br>363 → 368 -0.145<br>364 → 369 0.659<br>357 → 368 0.523

				363 → 369 0.164<br>365 → 369 -0.130<br>366 → 370 0.135<br>367 → 373 -0.340<br>367 → 376 -0.101<br>355 → 368 -0.159<br>356 → 368 0.167<br>367 → 374 0.618<br>367 → 375 0.155<br>367 → 378 -0.125<br>355 → 368 0.406<br>355 → 369 -0.115<br>356 → 368 0.509<br>356 → 369 0.121<br>365 → 369 0.112

Table S9: First 30 TDDFT singlet transitions of singlet 2[EtPh] eclipsed dimer. B3LYP-D3/6-31+G/PCM(ACN).

Excited State	Energy (eV)	Wavelength (nm)	Oscillator Strength (f)	Transitions
1	0.1851	6697.11	0.0144	335 → 336 1.253
2	1.0589	1170.86	0	335 → 336 1.253<br>335 → 338 0.719
3	1.0931	1134.29	0.491	335 → 336 1.253<br>335 → 338 0.719<br>335 → 337 0.714
4	1.8249	679.4	0	335 → 336 1.253<br>335 → 338 0.719<br>335 → 337 0.714<br>335 → 339 0.698
5	1.8792	659.76	0.0001	335 → 336 1.253<br>335 → 338 0.719<br>335 → 337 0.714<br>335 → 339 0.698<br>335 → 340 0.698
6	1.9922	622.35	0.0429	335 → 336 1.253<br>335 → 338 0.719<br>335 → 337 0.714<br>335 → 339 0.698<br>335 → 340 0.698<br>334 → 336 0.703
7	2.0158	615.06	0	335 → 336 1.253<br>335 → 338 0.719<br>335 → 337 0.714<br>335 → 339 0.698<br>335 → 340 0.698<br>334 → 336 0.703<br>333 → 336 0.704
8	2.1684	571.77	0	335 → 336 1.253<br>335 → 338 0.719<br>335 → 337 0.714

				335 → 339 0.698<br>335 → 340 0.698<br>334 → 336 0.703<br>333 → 336 0.704<br>330 → 336 -0.143<br>332 → 336 0.688
9	2.1914	565.79	0.0028	335 → 336 1.253<br>335 → 338 0.719<br>335 → 337 0.714<br>335 → 339 0.698<br>335 → 340 0.698<br>334 → 336 0.703<br>333 → 336 0.704<br>330 → 336 -0.143<br>332 → 336 0.688<br>329 → 336 -0.482<br>331 → 336 0.512
10	2.2733	545.4	0	335 → 336 1.253<br>335 → 338 0.719<br>335 → 337 0.714<br>335 → 339 0.698<br>335 → 340 0.698<br>334 → 336 0.703<br>333 → 336 0.704<br>330 → 336 -0.143<br>332 → 336 0.688<br>329 → 336 -0.482<br>331 → 336 0.512<br>330 → 336 0.681<br>332 → 336 0.143
11	2.3332	531.4	0.627	335 → 336 1.253<br>335 → 338 0.719<br>335 → 337 0.714<br>335 → 339 0.698<br>335 → 340 0.698<br>334 → 336 0.703<br>333 → 336 0.704<br>330 → 336 -0.143<br>332 → 336 0.688<br>329 → 336 -0.482<br>331 → 336 0.512<br>330 → 336 0.681<br>332 → 336 0.143<br>326 → 336 0.147<br>329 → 336 0.489<br>331 → 336 0.458<br>335 → 337 -0.127

12	2.4339	509.41	0.0015	335	→	336	1.253
				335	→	338	0.719
				335	→	337	0.714
				335	→	339	0.698
				335	→	340	0.698
				334	→	336	0.703
				333	→	336	0.704
				330	→	336	-0.143
				332	→	336	0.688
				329	→	336	-0.482
				331	→	336	0.512
				330	→	336	0.681
				332	→	336	0.143
				326	→	336	0.147
				329	→	336	0.489
				331	→	336	0.458
				335	→	337	-0.127
				328	→	336	0.699
13	2.4391	508.32	0.013	335	→	336	1.253
				335	→	338	0.719
				335	→	337	0.714
				335	→	339	0.698
				335	→	340	0.698
				334	→	336	0.703
				333	→	336	0.704
				330	→	336	-0.143
				332	→	336	0.688
				329	→	336	-0.482
				331	→	336	0.512
				330	→	336	0.681
				332	→	336	0.143
				326	→	336	0.147
				329	→	336	0.489
				331	→	336	0.458
				335	→	337	-0.127
				328	→	336	0.699
				327	→	336	0.697

14	2.5531	485.61	0.003	335	→	336	1.253
				335	→	338	0.719
				335	→	337	0.714
				335	→	339	0.698
				335	→	340	0.698
				334	→	336	0.703
				333	→	336	0.704
				330	→	336	-0.143
				332	→	336	0.688
				329	→	336	-0.482
				331	→	336	0.512
				330	→	336	0.681
				332	→	336	0.143
				326	→	336	0.147
				329	→	336	0.489
				331	→	336	0.458
				335	→	337	-0.127
				328	→	336	0.699
				327	→	336	0.697
				333	→	338	0.290
				334	→	337	0.633
15	2.5714	482.16	0.0001	335	→	336	1.253
				335	→	338	0.719
				335	→	337	0.714
				335	→	339	0.698
				335	→	340	0.698
				334	→	336	0.703
				333	→	336	0.704
				330	→	336	-0.143
				332	→	336	0.688
				329	→	336	-0.482
				331	→	336	0.512
				330	→	336	0.681
				332	→	336	0.143
				326	→	336	0.147
				329	→	336	0.489
				331	→	336	0.458
				335	→	337	-0.127
				328	→	336	0.699
				327	→	336	0.697
				333	→	338	0.290
				334	→	337	0.633
				325	→	336	-0.240
				333	→	337	0.539
				334	→	338	0.368

16	2.5783	480.88	0.001	335	→	336	1.253
				335	→	338	0.719
				335	→	337	0.714
				335	→	339	0.698
				335	→	340	0.698
				334	→	336	0.703
				333	→	336	0.704
				330	→	336	-0.143
				332	→	336	0.688
				329	→	336	-0.482
				331	→	336	0.512
				330	→	336	0.681
				332	→	336	0.143
				326	→	336	0.147
				329	→	336	0.489
				331	→	336	0.458
				335	→	337	-0.127
				328	→	336	0.699
				327	→	336	0.697
				333	→	338	0.290
				334	→	337	0.633
				325	→	336	-0.240
				333	→	337	0.539
				334	→	338	0.368
				325	→	336	0.644
				333	→	337	0.210
				334	→	338	0.127

17	2.5909	478.53	0.3296	335 → 336 1.253<br>335 → 338 0.719<br>335 → 337 0.714<br>335 → 339 0.698<br>335 → 340 0.698<br>334 → 336 0.703<br>333 → 336 0.704<br>330 → 336 -0.143<br>332 → 336 0.688<br>329 → 336 -0.482<br>331 → 336 0.512<br>330 → 336 0.681<br>332 → 336 0.143<br>326 → 336 0.147<br>329 → 336 0.489<br>331 → 336 0.458<br>335 → 337 -0.127<br>328 → 336 0.699<br>327 → 336 0.697<br>333 → 338 0.290<br>334 → 337 0.633<br>325 → 336 -0.240<br>333 → 337 0.539<br>334 → 338 0.368<br>325 → 336 0.644<br>333 → 337 0.210<br>334 → 338 0.127<br>326 → 336 0.670

18	2.6168	473.79	0.0002	335 → 336 1.253<br>335 → 338 0.719<br>335 → 337 0.714<br>335 → 339 0.698<br>335 → 340 0.698<br>334 → 336 0.703<br>333 → 336 0.704<br>330 → 336 -0.143<br>332 → 336 0.688<br>329 → 336 -0.482<br>331 → 336 0.512<br>330 → 336 0.681<br>332 → 336 0.143<br>326 → 336 0.147<br>329 → 336 0.489<br>331 → 336 0.458<br>335 → 337 -0.127<br>328 → 336 0.699<br>327 → 336 0.697<br>333 → 338 0.290<br>334 → 337 0.633<br>325 → 336 -0.240<br>333 → 337 0.539<br>334 → 338 0.368<br>325 → 336 0.644<br>333 → 337 0.210<br>334 → 338 0.127<br>326 → 336 0.670<br>324 → 336 0.697

19	2.6439	468.94	0	335 → 336 1.253<br>335 → 338 0.719<br>335 → 337 0.714<br>335 → 339 0.698<br>335 → 340 0.698<br>334 → 336 0.703<br>333 → 336 0.704<br>330 → 336 -0.143<br>332 → 336 0.688<br>329 → 336 -0.482<br>331 → 336 0.512<br>330 → 336 0.681<br>332 → 336 0.143<br>326 → 336 0.147<br>329 → 336 0.489<br>331 → 336 0.458<br>335 → 337 -0.127<br>328 → 336 0.699<br>327 → 336 0.697<br>333 → 338 0.290<br>334 → 337 0.633<br>325 → 336 -0.240<br>333 → 337 0.539<br>334 → 338 0.368<br>325 → 336 0.644<br>333 → 337 0.210<br>334 → 338 0.127<br>326 → 336 0.670<br>324 → 336 0.697<br>323 → 336 0.679<br>335 → 341 -0.146

20	2.655	466.99	0	335	→	336	1.253
				335	→	338	0.719
				335	→	337	0.714
				335	→	339	0.698
				335	→	340	0.698
				334	→	336	0.703
				333	→	336	0.704
				330	→	336	-0.143
				332	→	336	0.688
				329	→	336	-0.482
				331	→	336	0.512
				330	→	336	0.681
				332	→	336	0.143
				326	→	336	0.147
				329	→	336	0.489
				331	→	336	0.458
				335	→	337	-0.127
				328	→	336	0.699
				327	→	336	0.697
				333	→	338	0.290
				334	→	337	0.633
				325	→	336	-0.240
				333	→	337	0.539
				334	→	338	0.368
				325	→	336	0.644
				333	→	337	0.210
				334	→	338	0.127
				326	→	336	0.670
				324	→	336	0.697
				323	→	336	0.679
				335	→	341	-0.146
				322	→	336	0.180
				323	→	336	0.152
				335	→	341	0.653

21	2.6924	460.5	0.0021	335	→	336	1.253
				335	→	338	0.719
				335	→	337	0.714
				335	→	339	0.698
				335	→	340	0.698
				334	→	336	0.703
				333	→	336	0.704
				330	→	336	-0.143
				332	→	336	0.688
				329	→	336	-0.482
				331	→	336	0.512
				330	→	336	0.681
				332	→	336	0.143
				326	→	336	0.147
				329	→	336	0.489
				331	→	336	0.458
				335	→	337	-0.127
				328	→	336	0.699
				327	→	336	0.697
				333	→	338	0.290
				334	→	337	0.633
				325	→	336	-0.240
				333	→	337	0.539
				334	→	338	0.368
				325	→	336	0.644
				333	→	337	0.210
				334	→	338	0.127
				326	→	336	0.670
				324	→	336	0.697
				323	→	336	0.679
				335	→	341	-0.146
				322	→	336	0.180
				323	→	336	0.152
				335	→	341	0.653
				321	→	336	0.173
				335	→	342	0.668

22	2.7283	454.44	0	335	→	336	1.253
				335	→	338	0.719
				335	→	337	0.714
				335	→	339	0.698
				335	→	340	0.698
				334	→	336	0.703
				333	→	336	0.704
				330	→	336	-0.143
				332	→	336	0.688
				329	→	336	-0.482
				331	→	336	0.512
				330	→	336	0.681
				332	→	336	0.143
				326	→	336	0.147
				329	→	336	0.489
				331	→	336	0.458
				335	→	337	-0.127
				328	→	336	0.699
				327	→	336	0.697
				333	→	338	0.290
				334	→	337	0.633
				325	→	336	-0.240
				333	→	337	0.539
				334	→	338	0.368
				325	→	336	0.644
				333	→	337	0.210
				334	→	338	0.127
				326	→	336	0.670
				324	→	336	0.697
				323	→	336	0.679
				335	→	341	-0.146
				322	→	336	0.180
				323	→	336	0.152
				335	→	341	0.653
				321	→	336	0.173
				335	→	342	0.668
				322	→	336	-0.144
				333	→	337	-0.380
				334	→	338	0.556

23	2.7428	452.04	0.0033	335 → 336 1.253<br>335 → 338 0.719<br>335 → 337 0.714<br>335 → 339 0.698<br>335 → 340 0.698<br>334 → 336 0.703<br>333 → 336 0.704<br>330 → 336 -0.143<br>332 → 336 0.688<br>329 → 336 -0.482<br>331 → 336 0.512<br>330 → 336 0.681<br>332 → 336 0.143<br>326 → 336 0.147<br>329 → 336 0.489<br>331 → 336 0.458<br>335 → 337 -0.127<br>328 → 336 0.699<br>327 → 336 0.697<br>333 → 338 0.290<br>334 → 337 0.633<br>325 → 336 -0.240<br>333 → 337 0.539<br>334 → 338 0.368<br>325 → 336 0.644<br>333 → 337 0.210<br>334 → 338 0.127<br>326 → 336 0.670<br>324 → 336 0.697<br>323 → 336 0.679<br>335 → 341 -0.146<br>322 → 336 0.180<br>323 → 336 0.152<br>335 → 341 0.653<br>321 → 336 0.173<br>335 → 342 0.668<br>322 → 336 -0.144<br>333 → 337 -0.380<br>334 → 338 0.556<br>321 → 336 0.186<br>331 → 337 -0.141<br>333 → 338 0.597<br>334 → 337 -0.275

24	2.7636	448.63	0.0002	335	→	336	1.253
				335	→	338	0.719
				335	→	337	0.714
				335	→	339	0.698
				335	→	340	0.698
				334	→	336	0.703
				333	→	336	0.704
				330	→	336	-0.143
				332	→	336	0.688
				329	→	336	-0.482
				331	→	336	0.512
				330	→	336	0.681
				332	→	336	0.143
				326	→	336	0.147
				329	→	336	0.489
				331	→	336	0.458
				335	→	337	-0.127
				328	→	336	0.699
				327	→	336	0.697
				333	→	338	0.290
				334	→	337	0.633
				325	→	336	-0.240
				333	→	337	0.539
				334	→	338	0.368
				325	→	336	0.644
				333	→	337	0.210
				334	→	338	0.127
				326	→	336	0.670
				324	→	336	0.697
				323	→	336	0.679
				335	→	341	-0.146
				322	→	336	0.180
				323	→	336	0.152
				335	→	341	0.653
				321	→	336	0.173
				335	→	342	0.668
				322	→	336	-0.144
				333	→	337	-0.380
				334	→	338	0.556
				321	→	336	0.186
				331	→	337	-0.141
				333	→	338	0.597
				334	→	337	-0.275
				329	→	338	-0.206
				330	→	337	-0.130
				331	→	338	0.177
				332	→	337	0.629

25	2.7834	445.44	0.0653	335	→	336	1.253
				335	→	338	0.719
				335	→	337	0.714
				335	→	339	0.698
				335	→	340	0.698
				334	→	336	0.703
				333	→	336	0.704
				330	→	336	-0.143
				332	→	336	0.688
				329	→	336	-0.482
				331	→	336	0.512
				330	→	336	0.681
				332	→	336	0.143
				326	→	336	0.147
				329	→	336	0.489
				331	→	336	0.458
				335	→	337	-0.127
				328	→	336	0.699
				327	→	336	0.697
				333	→	338	0.290
				334	→	337	0.633
				325	→	336	-0.240
				333	→	337	0.539
				334	→	338	0.368
				325	→	336	0.644
				333	→	337	0.210
				334	→	338	0.127
				326	→	336	0.670
				324	→	336	0.697
				323	→	336	0.679
				335	→	341	-0.146
				322	→	336	0.180
				323	→	336	0.152
				335	→	341	0.653
				321	→	336	0.173
				335	→	342	0.668
				322	→	336	-0.144
				333	→	337	-0.380
				334	→	338	0.556
				321	→	336	0.186
				331	→	337	-0.141
				333	→	338	0.597
				334	→	337	-0.275
				329	→	338	-0.206
				330	→	337	-0.130
				331	→	338	0.177
				332	→	337	0.629
				329	→	337	-0.413
				330	→	338	-0.111
				331	→	337	0.422
				332	→	338	0.339

26	2.7969	443.29	0.0001	335	→	336	1.253
				335	→	338	0.719
				335	→	337	0.714
				335	→	339	0.698
				335	→	340	0.698
				334	→	336	0.703
				333	→	336	0.704
				330	→	336	-0.143
				332	→	336	0.688
				329	→	336	-0.482
				331	→	336	0.512
				330	→	336	0.681
				332	→	336	0.143
				326	→	336	0.147
				329	→	336	0.489
				331	→	336	0.458
				335	→	337	-0.127
				328	→	336	0.699
				327	→	336	0.697
				333	→	338	0.290
				334	→	337	0.633
				325	→	336	-0.240
				333	→	337	0.539
				334	→	338	0.368
				325	→	336	0.644
				333	→	337	0.210
				334	→	338	0.127
				326	→	336	0.670
				324	→	336	0.697
				323	→	336	0.679
				335	→	341	-0.146
				322	→	336	0.180
				323	→	336	0.152
				335	→	341	0.653
				321	→	336	0.173
				335	→	342	0.668
				322	→	336	-0.144
				333	→	337	-0.380
				334	→	338	0.556
				321	→	336	0.186
				331	→	337	-0.141
				333	→	338	0.597
				334	→	337	-0.275
				329	→	338	-0.206
				330	→	337	-0.130
				331	→	338	0.177
				332	→	337	0.629
				329	→	337	-0.413
				330	→	338	-0.111
				331	→	337	0.422
				332	→	338	0.339

				322 → 336 0.584<br>330 → 337 0.198<br>334 → 338 0.166<br>335 → 341 -0.133<br>335 → 344 0.151<br>335 → 346 0.118
27	2.8092	441.36	0.0022	335 → 336 1.253<br>335 → 338 0.719<br>335 → 337 0.714<br>335 → 339 0.698<br>335 → 340 0.698<br>334 → 336 0.703<br>333 → 336 0.704<br>330 → 336 -0.143<br>332 → 336 0.688<br>329 → 336 -0.482<br>331 → 336 0.512<br>330 → 336 0.681<br>332 → 336 0.143<br>326 → 336 0.147<br>329 → 336 0.489<br>331 → 336 0.458<br>335 → 337 -0.127<br>328 → 336 0.699

				327 → 336 0.697
				333 → 338 0.290
				334 → 337 0.633
				325 → 336 -0.240
				333 → 337 0.539
				334 → 338 0.368
				325 → 336 0.644
				333 → 337 0.210
				334 → 338 0.127
				326 → 336 0.670
				324 → 336 0.697
				323 → 336 0.679
				335 → 341 -0.146
				322 → 336 0.180
				323 → 336 0.152
				335 → 341 0.653
				321 → 336 0.173
				335 → 342 0.668
				322 → 336 -0.144
				333 → 337 -0.380
				334 → 338 0.556
				321 → 336 0.186
				331 → 337 -0.141
				333 → 338 0.597
				334 → 337 -0.275
				329 → 338 -0.206
				330 → 337 -0.130
				331 → 338 0.177
				332 → 337 0.629
				329 → 337 -0.413
				330 → 338 -0.111
				331 → 337 0.422
				332 → 338 0.339
				322 → 336 0.584
				330 → 337 0.198
				334 → 338 0.166
				335 → 341 -0.133
				335 → 344 0.151
				335 → 346 0.118
				321 → 336 0.568
				329 → 337 -0.106
				331 → 337 -0.191
				333 → 338 -0.220
				335 → 342 -0.108
				335 → 343 -0.139
				335 → 345 0.126

28	2.8514	434.83	0.0394	335	→	336	1.253
				335	→	338	0.719
				335	→	337	0.714
				335	→	339	0.698
				335	→	340	0.698
				334	→	336	0.703
				333	→	336	0.704
				330	→	336	-0.143
				332	→	336	0.688
				329	→	336	-0.482
				331	→	336	0.512
				330	→	336	0.681
				332	→	336	0.143
				326	→	336	0.147
				329	→	336	0.489
				331	→	336	0.458
				335	→	337	-0.127
				328	→	336	0.699
				327	→	336	0.697
				333	→	338	0.290
				334	→	337	0.633
				325	→	336	-0.240
				333	→	337	0.539
				334	→	338	0.368
				325	→	336	0.644
				333	→	337	0.210
				334	→	338	0.127
				326	→	336	0.670
				324	→	336	0.697
				323	→	336	0.679
				335	→	341	-0.146
				322	→	336	0.180
				323	→	336	0.152
				335	→	341	0.653
				321	→	336	0.173
				335	→	342	0.668
				322	→	336	-0.144
				333	→	337	-0.380
				334	→	338	0.556
				321	→	336	0.186
				331	→	337	-0.141
				333	→	338	0.597
				334	→	337	-0.275
				329	→	338	-0.206
				330	→	337	-0.130
				331	→	338	0.177
				332	→	337	0.629
				329	→	337	-0.413
				330	→	338	-0.111
				331	→	337	0.422
				332	→	338	0.339

				322	→	336	0.584
				330	→	337	0.198
				334	→	338	0.166
				335	→	341	-0.133
				335	→	344	0.151
				335	→	346	0.118
				321	→	336	0.568
				329	→	337	-0.106
				331	→	337	-0.191
				333	→	338	-0.220
				335	→	342	-0.108
				335	→	343	-0.139
				335	→	345	0.126
				329	→	337	-0.295
				331	→	337	-0.252
				335	→	343	0.517
				335	→	345	-0.241
29	2.8563	434.07	0	335	→	336	1.253
				335	→	338	0.719
				335	→	337	0.714
				335	→	339	0.698
				335	→	340	0.698
				334	→	336	0.703
				333	→	336	0.704
				330	→	336	-0.143
				332	→	336	0.688
				329	→	336	-0.482
				331	→	336	0.512
				330	→	336	0.681
				332	→	336	0.143
				326	→	336	0.147
				329	→	336	0.489
				331	→	336	0.458
				335	→	337	-0.127
				328	→	336	0.699
				327	→	336	0.697
				333	→	338	0.290
				334	→	337	0.633
				325	→	336	-0.240
				333	→	337	0.539
				334	→	338	0.368
				325	→	336	0.644
				333	→	337	0.210
				334	→	338	0.127
				326	→	336	0.670
				324	→	336	0.697
				323	→	336	0.679
				335	→	341	-0.146
				322	→	336	0.180
				323	→	336	0.152
				335	→	341	0.653

				321 → 336 0.173
				335 → 342 0.668
				322 → 336 -0.144
				333 → 337 -0.380
				334 → 338 0.556
				321 → 336 0.186
				331 → 337 -0.141
				333 → 338 0.597
				334 → 337 -0.275
				329 → 338 -0.206
				330 → 337 -0.130
				331 → 338 0.177
				332 → 337 0.629
				329 → 337 -0.413
				330 → 338 -0.111
				331 → 337 0.422
				332 → 338 0.339
				322 → 336 0.584
				330 → 337 0.198
				334 → 338 0.166
				335 → 341 -0.133
				335 → 344 0.151
				335 → 346 0.118
				321 → 336 0.568
				329 → 337 -0.106
				331 → 337 -0.191
				333 → 338 -0.220
				335 → 342 -0.108
				335 → 343 -0.139
				335 → 345 0.126
				329 → 337 -0.295
				331 → 337 -0.252
				335 → 343 0.517
				335 → 345 -0.241
				330 → 337 -0.368
				335 → 344 0.545
				335 → 346 0.196

30	2.8966	428.04	0.0094	335 → 336 1.253<br>335 → 338 0.719<br>335 → 337 0.714<br>335 → 339 0.698<br>335 → 340 0.698<br>334 → 336 0.703<br>333 → 336 0.704<br>330 → 336 -0.143<br>332 → 336 0.688<br>329 → 336 -0.482<br>331 → 336 0.512<br>330 → 336 0.681<br>332 → 336 0.143<br>326 → 336 0.147<br>329 → 336 0.489<br>331 → 336 0.458<br>335 → 337 -0.127<br>328 → 336 0.699<br>327 → 336 0.697<br>333 → 338 0.290<br>334 → 337 0.633<br>325 → 336 -0.240<br>333 → 337 0.539<br>334 → 338 0.368<br>325 → 336 0.644<br>333 → 337 0.210<br>334 → 338 0.127<br>326 → 336 0.670<br>324 → 336 0.697<br>323 → 336 0.679<br>335 → 341 -0.146<br>322 → 336 0.180<br>323 → 336 0.152<br>335 → 341 0.653<br>321 → 336 0.173<br>335 → 342 0.668<br>322 → 336 -0.144<br>333 → 337 -0.380<br>334 → 338 0.556<br>321 → 336 0.186<br>331 → 337 -0.141<br>333 → 338 0.597<br>334 → 337 -0.275<br>329 → 338 -0.206<br>330 → 337 -0.130<br>331 → 338 0.177<br>332 → 337 0.629<br>329 → 337 -0.413<br>330 → 338 -0.111<br>331 → 337 0.422<br>332 → 338 0.339

				322 → 336 0.584<br>330 → 337 0.198<br>334 → 338 0.166<br>335 → 341 -0.133<br>335 → 344 0.151<br>335 → 346 0.118<br>321 → 336 0.568<br>329 → 337 -0.106<br>331 → 337 -0.191<br>333 → 338 -0.220<br>335 → 342 -0.108<br>335 → 343 -0.139<br>335 → 345 0.126<br>329 → 337 -0.295<br>331 → 337 -0.252<br>335 → 343 0.517<br>335 → 345 -0.241<br>330 → 337 -0.368<br>335 → 344 0.545<br>335 → 346 0.196<br>319 → 336 0.117<br>321 → 336 0.189<br>329 → 337 0.217<br>331 → 337 0.325<br>332 → 338 -0.169<br>335 → 342 -0.112<br>335 → 343 0.407<br>335 → 345 0.249

Table S10: First 20 TDDFT singlet transitions of singlet 3[nBuPh] eclipsed dimer. B3LYP-D3/6-31+G/PCM(ACN).

Excited State	Energy (eV)	Wavelength (nm)	Oscillator Strength (f)	Transitions
1	0.4553	2723.31	0.0714	367 → 368 0.813
2	1.0362	1196.57	0.6425	367 → 368 0.813<br>363 → 368 -0.109<br>367 → 369 0.712
3	1.0959	1131.3	0.0002	367 → 368 0.813<br>363 → 368 -0.109<br>367 → 369 0.712<br>367 → 370 0.714
4	1.783	695.37	0	367 → 368 0.813<br>363 → 368 -0.109<br>367 → 369 0.712<br>367 → 370 0.714<br>367 → 371 0.699
5	2.0197	613.88	0.0036	367 → 368 0.813<br>363 → 368 -0.109<br>367 → 369 0.712<br>367 → 370 0.714<br>367 → 371 0.699<br>366 → 368 0.218<br>367 → 372 0.662
6	2.0296	610.88	0.0655	367 → 368 0.813<br>363 → 368 -0.109<br>367 → 369 0.712<br>367 → 370 0.714<br>367 → 371 0.699<br>366 → 368 0.218<br>367 → 372 0.662<br>366 → 368 0.667<br>367 → 372 -0.216
7	2.101	590.13	0.0002	367 → 368 0.813<br>363 → 368 -0.109<br>367 → 369 0.712<br>367 → 370 0.714<br>367 → 371 0.699<br>366 → 368 0.218<br>367 → 372 0.662<br>366 → 368 0.667<br>367 → 372 -0.216<br>365 → 368 0.701
8	2.2913	541.12	0.0002	367 → 368 0.813<br>363 → 368 -0.109<br>367 → 369 0.712<br>367 → 370 0.714<br>367 → 371 0.699<br>366 → 368 0.218<br>367 → 372 0.662<br>366 → 368 0.667

				367 → 372 -0.216<br>365 → 368 0.701<br>364 → 368 0.694
9	2.3139	535.81	0.0002	367 → 368 0.813<br>363 → 368 -0.109<br>367 → 369 0.712<br>367 → 370 0.714<br>367 → 371 0.699<br>366 → 368 0.218<br>367 → 372 0.662<br>366 → 368 0.667<br>367 → 372 -0.216<br>365 → 368 0.701<br>364 → 368 0.694<br>362 → 368 0.696
10	2.369	523.36	0.5205	367 → 368 0.813<br>363 → 368 -0.109<br>367 → 369 0.712<br>367 → 370 0.714<br>367 → 371 0.699<br>366 → 368 0.218<br>367 → 372 0.662<br>366 → 368 0.667<br>367 → 372 -0.216<br>365 → 368 0.701<br>364 → 368 0.694<br>362 → 368 0.696<br>361 → 368 0.426<br>363 → 368 0.546
11	2.3821	520.48	0.4544	367 → 368 0.813<br>363 → 368 -0.109<br>367 → 369 0.712<br>367 → 370 0.714<br>367 → 371 0.699<br>366 → 368 0.218<br>367 → 372 0.662<br>366 → 368 0.667<br>367 → 372 -0.216<br>365 → 368 0.701<br>364 → 368 0.694<br>362 → 368 0.696<br>361 → 368 0.426<br>363 → 368 0.546<br>361 → 368 0.553<br>363 → 368 -0.417

12	2.4559	504.84	0.0051	367 → 368 0.813<br>363 → 368 -0.109<br>367 → 369 0.712<br>367 → 370 0.714<br>367 → 371 0.699<br>366 → 368 0.218<br>367 → 372 0.662<br>366 → 368 0.667<br>367 → 372 -0.216<br>365 → 368 0.701<br>364 → 368 0.694<br>362 → 368 0.696<br>361 → 368 0.426<br>363 → 368 0.546<br>361 → 368 0.553<br>363 → 368 -0.417<br>366 → 369 0.690
13	2.5296	490.13	0.0031	367 → 368 0.813<br>363 → 368 -0.109<br>367 → 369 0.712<br>367 → 370 0.714<br>367 → 371 0.699<br>366 → 368 0.218<br>367 → 372 0.662<br>366 → 368 0.667<br>367 → 372 -0.216<br>365 → 368 0.701<br>364 → 368 0.694<br>362 → 368 0.696<br>361 → 368 0.426<br>363 → 368 0.546<br>361 → 368 0.553<br>363 → 368 -0.417<br>366 → 369 0.690<br>359 → 368 0.348<br>360 → 368 -0.395<br>365 → 369 0.435<br>366 → 370 0.151

14	2.5344	489.21	0.0227	367	→	368	0.813
				363	→	368	-0.109
				367	→	369	0.712
				367	→	370	0.714
				367	→	371	0.699
				366	→	368	0.218
				367	→	372	0.662
				366	→	368	0.667
				367	→	372	-0.216
				365	→	368	0.701
				364	→	368	0.694
				362	→	368	0.696
				361	→	368	0.426
				363	→	368	0.546
				361	→	368	0.553
				363	→	368	-0.417
				366	→	369	0.690
				359	→	368	0.348
				360	→	368	-0.395
				365	→	369	0.435
				366	→	370	0.151
				359	→	368	0.248
				360	→	368	0.576
				365	→	369	0.289
15	2.5433	487.49	0.0023	367	→	368	0.813
				363	→	368	-0.109
				367	→	369	0.712
				367	→	370	0.714
				367	→	371	0.699
				366	→	368	0.218
				367	→	372	0.662
				366	→	368	0.667
				367	→	372	-0.216
				365	→	368	0.701
				364	→	368	0.694
				362	→	368	0.696
				361	→	368	0.426
				363	→	368	0.546
				361	→	368	0.553
				363	→	368	-0.417
				366	→	369	0.690
				359	→	368	0.348
				360	→	368	-0.395
				365	→	369	0.435
				366	→	370	0.151
				359	→	368	0.248
				360	→	368	0.576
				365	→	369	0.289
				359	→	368	0.554
				365	→	369	-0.399
				366	→	370	-0.125

16	2.6242	472.46	0.1525	367 → 368 0.813<br>363 → 368 -0.109<br>367 → 369 0.712<br>367 → 370 0.714<br>367 → 371 0.699<br>366 → 368 0.218<br>367 → 372 0.662<br>366 → 368 0.667<br>367 → 372 -0.216<br>365 → 368 0.701<br>364 → 368 0.694<br>362 → 368 0.696<br>361 → 368 0.426<br>363 → 368 0.546<br>361 → 368 0.553<br>363 → 368 -0.417<br>366 → 369 0.690<br>359 → 368 0.348<br>360 → 368 -0.395<br>365 → 369 0.435<br>366 → 370 0.151<br>359 → 368 0.248<br>360 → 368 0.576<br>365 → 369 0.289<br>359 → 368 0.554<br>365 → 369 -0.399<br>366 → 370 -0.125<br>358 → 368 0.679

17	2.6338	470.74	0.0019	367 → 368 0.813<br>363 → 368 -0.109<br>367 → 369 0.712<br>367 → 370 0.714<br>367 → 371 0.699<br>366 → 368 0.218<br>367 → 372 0.662<br>366 → 368 0.667<br>367 → 372 -0.216<br>365 → 368 0.701<br>364 → 368 0.694<br>362 → 368 0.696<br>361 → 368 0.426<br>363 → 368 0.546<br>361 → 368 0.553<br>363 → 368 -0.417<br>366 → 369 0.690<br>359 → 368 0.348<br>360 → 368 -0.395<br>365 → 369 0.435<br>366 → 370 0.151<br>359 → 368 0.248<br>360 → 368 0.576<br>365 → 369 0.289<br>359 → 368 0.554<br>365 → 369 -0.399<br>366 → 370 -0.125<br>358 → 368 0.679<br>357 → 368 -0.102<br>367 → 373 0.674

18	2.6835	462.02	0.0003	367 → 368 0.813<br>363 → 368 -0.109<br>367 → 369 0.712<br>367 → 370 0.714<br>367 → 371 0.699<br>366 → 368 0.218<br>367 → 372 0.662<br>366 → 368 0.667<br>367 → 372 -0.216<br>365 → 368 0.701<br>364 → 368 0.694<br>362 → 368 0.696<br>361 → 368 0.426<br>363 → 368 0.546<br>361 → 368 0.553<br>363 → 368 -0.417<br>366 → 369 0.690<br>359 → 368 0.348<br>360 → 368 -0.395<br>365 → 369 0.435<br>366 → 370 0.151<br>359 → 368 0.248<br>360 → 368 0.576<br>365 → 369 0.289<br>359 → 368 0.554<br>365 → 369 -0.399<br>366 → 370 -0.125<br>358 → 368 0.679<br>357 → 368 -0.102<br>367 → 373 0.674<br>362 → 369 0.126<br>364 → 369 0.151<br>365 → 369 -0.228<br>366 → 370 0.624

19	2.7334	453.59	0.0037	367 → 368 0.813<br>363 → 368 -0.109<br>367 → 369 0.712<br>367 → 370 0.714<br>367 → 371 0.699<br>366 → 368 0.218<br>367 → 372 0.662<br>366 → 368 0.667<br>367 → 372 -0.216<br>365 → 368 0.701<br>364 → 368 0.694<br>362 → 368 0.696<br>361 → 368 0.426<br>363 → 368 0.546<br>361 → 368 0.553<br>363 → 368 -0.417<br>366 → 369 0.690<br>359 → 368 0.348<br>360 → 368 -0.395<br>365 → 369 0.435<br>366 → 370 0.151<br>359 → 368 0.248<br>360 → 368 0.576<br>365 → 369 0.289<br>359 → 368 0.554<br>365 → 369 -0.399<br>366 → 370 -0.125<br>358 → 368 0.679<br>357 → 368 -0.102<br>367 → 373 0.674<br>362 → 369 0.126<br>364 → 369 0.151<br>365 → 369 -0.228<br>366 → 370 0.624<br>355 → 368 -0.145<br>363 → 369 -0.364<br>365 → 370 0.328<br>367 → 374 0.469

20	2.7385	452.74	0.007	367	→	368	0.813
				363	→	368	-0.109
				367	→	369	0.712
				367	→	370	0.714
				367	→	371	0.699
				366	→	368	0.218
				367	→	372	0.662
				366	→	368	0.667
				367	→	372	-0.216
				365	→	368	0.701
				364	→	368	0.694
				362	→	368	0.696
				361	→	368	0.426
				363	→	368	0.546
				361	→	368	0.553
				363	→	368	-0.417
				366	→	369	0.690
				359	→	368	0.348
				360	→	368	-0.395
				365	→	369	0.435
				366	→	370	0.151
				359	→	368	0.248
				360	→	368	0.576
				365	→	369	0.289
				359	→	368	0.554
				365	→	369	-0.399
				366	→	370	-0.125
				358	→	368	0.679
				357	→	368	-0.102
				367	→	373	0.674
				362	→	369	0.126
				364	→	369	0.151
				365	→	369	-0.228
				366	→	370	0.624
				355	→	368	-0.145
				363	→	369	-0.364
				365	→	370	0.328
				367	→	374	0.469
				355	→	368	0.238
				356	→	368	-0.110
				363	→	369	0.134
				365	→	370	-0.407
				367	→	374	0.473

Table S11: First 30 TDDFT singlet transitions of singlet 1[Ph] crystal long dimer. B3LYP-D3/6-31+G/PCM(ACN).

Excited State	Energy (eV)	Wavelength (nm)	Oscillator Strength (f)	Transitions
1	0.0003	3635169.48	0	299 → 304 -0.167<br>301 → 304 0.109<br>303 → 304 -30.406<br>303 → 306 -1.000
2	1.0049	1233.75	0.8828	299 → 304 -0.167<br>301 → 304 0.109<br>303 → 304 -30.406<br>303 → 306 -1.000<br>303 → 306 0.720
3	1.0135	1223.34	0	299 → 304 -0.167<br>301 → 304 0.109<br>303 → 304 -30.406<br>303 → 306 -1.000<br>303 → 306 0.720<br>303 → 305 0.720
4	1.8864	657.26	0	299 → 304 -0.167<br>301 → 304 0.109<br>303 → 304 -30.406<br>303 → 306 -1.000<br>303 → 306 0.720<br>303 → 305 0.720<br>303 → 307 0.699
5	1.8865	657.22	0.0009	299 → 304 -0.167<br>301 → 304 0.109<br>303 → 304 -30.406<br>303 → 306 -1.000<br>303 → 306 0.720<br>303 → 305 0.720<br>303 → 307 0.699<br>303 → 308 0.700
6	2.1905	566.01	0.1588	299 → 304 -0.167<br>301 → 304 0.109<br>303 → 304 -30.406<br>303 → 306 -1.000<br>303 → 306 0.720<br>303 → 305 0.720<br>303 → 307 0.699<br>303 → 308 0.700<br>299 → 304 0.219<br>301 → 304 0.669
7	2.1908	565.93	0	299 → 304 -0.167<br>301 → 304 0.109<br>303 → 304 -30.406<br>303 → 306 -1.000<br>303 → 306 0.720<br>303 → 305 0.720<br>303 → 307 0.699

				303 → 308 0.700<br>299 → 304 0.219<br>301 → 304 0.669<br>300 → 304 0.236<br>302 → 304 0.663
8	2.331	531.9	1.1266	299 → 304 -0.167<br>301 → 304 0.109<br>303 → 304 -30.406<br>303 → 306 -1.000<br>303 → 306 0.720<br>303 → 305 0.720<br>303 → 307 0.699<br>303 → 308 0.700<br>299 → 304 0.219<br>301 → 304 0.669<br>300 → 304 0.236<br>302 → 304 0.663<br>299 → 304 0.666<br>301 → 304 -0.216
9	2.3385	530.18	0	299 → 304 -0.167<br>301 → 304 0.109<br>303 → 304 -30.406<br>303 → 306 -1.000<br>303 → 306 0.720<br>303 → 305 0.720<br>303 → 307 0.699<br>303 → 308 0.700<br>299 → 304 0.219<br>301 → 304 0.669<br>300 → 304 0.236<br>302 → 304 0.663<br>299 → 304 0.666<br>301 → 304 -0.216<br>300 → 304 0.658<br>302 → 304 -0.232
10	2.5688	482.65	0.0194	299 → 304 -0.167<br>301 → 304 0.109<br>303 → 304 -30.406<br>303 → 306 -1.000<br>303 → 306 0.720<br>303 → 305 0.720<br>303 → 307 0.699<br>303 → 308 0.700<br>299 → 304 0.219<br>301 → 304 0.669<br>300 → 304 0.236<br>302 → 304 0.663<br>299 → 304 0.666<br>301 → 304 -0.216<br>300 → 304 0.658

				302 → 304 -0.232<br>297 → 304 0.649<br>303 → 310 -0.258
11	2.5688	482.65	0	299 → 304 -0.167<br>301 → 304 0.109<br>303 → 304 -30.406<br>303 → 306 -1.000<br>303 → 306 0.720<br>303 → 305 0.720<br>303 → 307 0.699<br>303 → 308 0.700<br>299 → 304 0.219<br>301 → 304 0.669<br>300 → 304 0.236<br>302 → 304 0.663<br>299 → 304 0.666<br>301 → 304 -0.216<br>300 → 304 0.658<br>302 → 304 -0.232<br>297 → 304 0.649<br>303 → 310 -0.258<br>298 → 304 0.649<br>303 → 309 -0.257
12	2.6303	471.37	0.0005	299 → 304 -0.167<br>301 → 304 0.109<br>303 → 304 -30.406<br>303 → 306 -1.000<br>303 → 306 0.720<br>303 → 305 0.720<br>303 → 307 0.699<br>303 → 308 0.700<br>299 → 304 0.219<br>301 → 304 0.669<br>300 → 304 0.236<br>302 → 304 0.663<br>299 → 304 0.666<br>301 → 304 -0.216<br>300 → 304 0.658<br>302 → 304 -0.232<br>297 → 304 0.649<br>303 → 310 -0.258<br>298 → 304 0.649<br>303 → 309 -0.257<br>297 → 304 0.231<br>303 → 310 0.640

13	2.6304	471.35	0	299 → 304 -0.167<br>301 → 304 0.109<br>303 → 304 -30.406<br>303 → 306 -1.000<br>303 → 306 0.720<br>303 → 305 0.720<br>303 → 307 0.699<br>303 → 308 0.700<br>299 → 304 0.219<br>301 → 304 0.669<br>300 → 304 0.236<br>302 → 304 0.663<br>299 → 304 0.666<br>301 → 304 -0.216<br>300 → 304 0.658<br>302 → 304 -0.232<br>297 → 304 0.649<br>303 → 310 -0.258<br>298 → 304 0.649<br>303 → 309 -0.257<br>297 → 304 0.231<br>303 → 310 0.640<br>298 → 304 0.231<br>303 → 309 0.640
14	2.7382	452.8	0	299 → 304 -0.167<br>301 → 304 0.109<br>303 → 304 -30.406<br>303 → 306 -1.000<br>303 → 306 0.720<br>303 → 305 0.720<br>303 → 307 0.699<br>303 → 308 0.700<br>299 → 304 0.219<br>301 → 304 0.669<br>300 → 304 0.236<br>302 → 304 0.663<br>299 → 304 0.666<br>301 → 304 -0.216<br>300 → 304 0.658<br>302 → 304 -0.232<br>297 → 304 0.649<br>303 → 310 -0.258<br>298 → 304 0.649<br>303 → 309 -0.257<br>297 → 304 0.231<br>303 → 310 0.640<br>298 → 304 0.231<br>303 → 309 0.640<br>293 → 304 0.428<br>296 → 304 -0.309<br>299 → 305 0.175

				300	→	306	0.175
				301	→	305	0.255
				302	→	306	0.250
15	2.7382	452.8	0.0027	299	→	304	-0.167
				301	→	304	0.109
				303	→	304	-30.406
				303	→	306	-1.000
				303	→	306	0.720
				303	→	305	0.720
				303	→	307	0.699
				303	→	308	0.700
				299	→	304	0.219
				301	→	304	0.669
				300	→	304	0.236
				302	→	304	0.663
				299	→	304	0.666
				301	→	304	-0.216
				300	→	304	0.658
				302	→	304	-0.232
				297	→	304	0.649
				303	→	310	-0.258
				298	→	304	0.649
				303	→	309	-0.257
				297	→	304	0.231
				303	→	310	0.640
				298	→	304	0.231
				303	→	309	0.640
				293	→	304	0.428
				296	→	304	-0.309
				299	→	305	0.175
				300	→	306	0.175
				301	→	305	0.255
				302	→	306	0.250
				294	→	304	0.427
				295	→	304	-0.314
				299	→	306	0.170
				300	→	305	0.177
				301	→	306	0.252
				302	→	305	0.252

16	2.7564	449.8	0.106	299 → 304 -0.167<br>301 → 304 0.109<br>303 → 304 -30.406<br>303 → 306 -1.000<br>303 → 306 0.720<br>303 → 305 0.720<br>303 → 307 0.699<br>303 → 308 0.700<br>299 → 304 0.219<br>301 → 304 0.669<br>300 → 304 0.236<br>302 → 304 0.663<br>299 → 304 0.666<br>301 → 304 -0.216<br>300 → 304 0.658<br>302 → 304 -0.232<br>297 → 304 0.649<br>303 → 310 -0.258<br>298 → 304 0.649<br>303 → 309 -0.257<br>297 → 304 0.231<br>303 → 310 0.640<br>298 → 304 0.231<br>303 → 309 0.640<br>293 → 304 0.428<br>296 → 304 -0.309<br>299 → 305 0.175<br>300 → 306 0.175<br>301 → 305 0.255<br>302 → 306 0.250<br>294 → 304 0.427<br>295 → 304 -0.314<br>299 → 306 0.170<br>300 → 305 0.177<br>301 → 306 0.252<br>302 → 305 0.252<br>294 → 304 0.239<br>295 → 304 0.618<br>301 → 306 0.112<br>302 → 305 0.111

17	2.7569	449.73	0	299	→	304	-0.167
				301	→	304	0.109
				303	→	304	-30.406
				303	→	306	-1.000
				303	→	306	0.720
				303	→	305	0.720
				303	→	307	0.699
				303	→	308	0.700
				299	→	304	0.219
				301	→	304	0.669
				300	→	304	0.236
				302	→	304	0.663
				299	→	304	0.666
				301	→	304	-0.216
				300	→	304	0.658
				302	→	304	-0.232
				297	→	304	0.649
				303	→	310	-0.258
				298	→	304	0.649
				303	→	309	-0.257
				297	→	304	0.231
				303	→	310	0.640
				298	→	304	0.231
				303	→	309	0.640
				293	→	304	0.428
				296	→	304	-0.309
				299	→	305	0.175
				300	→	306	0.175
				301	→	305	0.255
				302	→	306	0.250
				294	→	304	0.427
				295	→	304	-0.314
				299	→	306	0.170
				300	→	305	0.177
				301	→	306	0.252
				302	→	305	0.252
				294	→	304	0.239
				295	→	304	0.618
				301	→	306	0.112
				302	→	305	0.111
				293	→	304	0.234
				296	→	304	0.619
				301	→	305	0.114
				302	→	306	0.111

				299	→	304	-0.167
				301	→	304	0.109
				303	→	304	-30.406
				303	→	306	-1.000
				303	→	306	0.720
				303	→	305	0.720
				303	→	307	0.699
				303	→	308	0.700
				299	→	304	0.219
				301	→	304	0.669
				300	→	304	0.236
				302	→	304	0.663
				299	→	304	0.666
				301	→	304	-0.216
				300	→	304	0.658
				302	→	304	-0.232
				297	→	304	0.649
				303	→	310	-0.258
				298	→	304	0.649
				303	→	309	-0.257
				297	→	304	0.231
				303	→	310	0.640
				298	→	304	0.231
				303	→	309	0.640
18	2.7619	448.9	0	293	→	304	0.428
				296	→	304	-0.309
				299	→	305	0.175
				300	→	306	0.175
				301	→	305	0.255
				302	→	306	0.250
				294	→	304	0.427
				295	→	304	-0.314
				299	→	306	0.170
				300	→	305	0.177
				301	→	306	0.252
				302	→	305	0.252
				294	→	304	0.239
				295	→	304	0.618
				301	→	306	0.112
				302	→	305	0.111
				293	→	304	0.234
				296	→	304	0.619
				301	→	305	0.114
				302	→	306	0.111
				293	→	304	0.506
				299	→	305	-0.195
				300	→	306	-0.194
				301	→	305	-0.282
				302	→	306	-0.271
19	2.762	448.9	0.001	299	→	304	-0.167
				301	→	304	0.109

				303 → 304 -30.406<br>303 → 306 -1.000<br>303 → 306 0.720<br>303 → 305 0.720<br>303 → 307 0.699<br>303 → 308 0.700<br>299 → 304 0.219<br>301 → 304 0.669<br>300 → 304 0.236<br>302 → 304 0.663<br>299 → 304 0.666<br>301 → 304 -0.216<br>300 → 304 0.658<br>302 → 304 -0.232<br>297 → 304 0.649<br>303 → 310 -0.258<br>298 → 304 0.649<br>303 → 309 -0.257<br>297 → 304 0.231<br>303 → 310 0.640<br>298 → 304 0.231<br>303 → 309 0.640<br>293 → 304 0.428<br>296 → 304 -0.309<br>299 → 305 0.175<br>300 → 306 0.175<br>301 → 305 0.255<br>302 → 306 0.250<br>294 → 304 0.427<br>295 → 304 -0.314<br>299 → 306 0.170<br>300 → 305 0.177<br>301 → 306 0.252<br>302 → 305 0.252<br>294 → 304 0.239<br>295 → 304 0.618<br>301 → 306 0.112<br>302 → 305 0.111<br>293 → 304 0.234<br>296 → 304 0.619<br>301 → 305 0.114<br>302 → 306 0.111<br>293 → 304 0.506<br>299 → 305 -0.195<br>300 → 306 -0.194<br>301 → 305 -0.282<br>302 → 306 -0.271<br>294 → 304 0.504<br>299 → 306 -0.191<br>300 → 305 -0.200

				301	→	306	-0.275
				302	→	305	-0.280
20	2.8029	442.35	0.0646	299	→	304	-0.167
				301	→	304	0.109
				303	→	304	-30.406
				303	→	306	-1.000
				303	→	306	0.720
				303	→	305	0.720
				303	→	307	0.699
				303	→	308	0.700
				299	→	304	0.219
				301	→	304	0.669
				300	→	304	0.236
				302	→	304	0.663
				299	→	304	0.666
				301	→	304	-0.216
				300	→	304	0.658
				302	→	304	-0.232
				297	→	304	0.649
				303	→	310	-0.258
				298	→	304	0.649
				303	→	309	-0.257
				297	→	304	0.231
				303	→	310	0.640
				298	→	304	0.231
				303	→	309	0.640
				293	→	304	0.428
				296	→	304	-0.309
				299	→	305	0.175
				300	→	306	0.175
				301	→	305	0.255
				302	→	306	0.250
				294	→	304	0.427
				295	→	304	-0.314
				299	→	306	0.170
				300	→	305	0.177
				301	→	306	0.252
				302	→	305	0.252
				294	→	304	0.239
				295	→	304	0.618
				301	→	306	0.112
				302	→	305	0.111
				293	→	304	0.234
				296	→	304	0.619
				301	→	305	0.114
				302	→	306	0.111
				293	→	304	0.506
				299	→	305	-0.195
				300	→	306	-0.194
				301	→	305	-0.282
				302	→	306	-0.271

				294 → 304 0.504<br>299 → 306 -0.191<br>300 → 305 -0.200<br>301 → 306 -0.275<br>302 → 305 -0.280<br>289 → 304 -0.120<br>292 → 304 0.670
21	2.8033	442.28	0	299 → 304 -0.167<br>301 → 304 0.109<br>303 → 304 -30.406<br>303 → 306 -1.000<br>303 → 306 0.720<br>303 → 305 0.720<br>303 → 307 0.699<br>303 → 308 0.700<br>299 → 304 0.219<br>301 → 304 0.669<br>300 → 304 0.236<br>302 → 304 0.663<br>299 → 304 0.666<br>301 → 304 -0.216<br>300 → 304 0.658<br>302 → 304 -0.232<br>297 → 304 0.649<br>303 → 310 -0.258<br>298 → 304 0.649<br>303 → 309 -0.257<br>297 → 304 0.231<br>303 → 310 0.640<br>298 → 304 0.231<br>303 → 309 0.640<br>293 → 304 0.428<br>296 → 304 -0.309<br>299 → 305 0.175<br>300 → 306 0.175<br>301 → 305 0.255<br>302 → 306 0.250<br>294 → 304 0.427<br>295 → 304 -0.314<br>299 → 306 0.170<br>300 → 305 0.177<br>301 → 306 0.252<br>302 → 305 0.252<br>294 → 304 0.239<br>295 → 304 0.618<br>301 → 306 0.112<br>302 → 305 0.111<br>293 → 304 0.234<br>296 → 304 0.619<br>301 → 305 0.114<br>302 → 306 0.111

				293	→	304	0.506
				299	→	305	-0.195
				300	→	306	-0.194
				301	→	305	-0.282
				302	→	306	-0.271
				294	→	304	0.504
				299	→	306	-0.191
				300	→	305	-0.200
				301	→	306	-0.275
				302	→	305	-0.280
				289	→	304	-0.120
				292	→	304	0.670
				290	→	304	-0.115
				291	→	304	0.670
				296	→	304	-0.101
22	2.8535	434.49	0	299	→	304	-0.167
				301	→	304	0.109
				303	→	304	-30.406
				303	→	306	-1.000
				303	→	306	0.720
				303	→	305	0.720
				303	→	307	0.699
				303	→	308	0.700
				299	→	304	0.219
				301	→	304	0.669
				300	→	304	0.236
				302	→	304	0.663
				299	→	304	0.666
				301	→	304	-0.216
				300	→	304	0.658
				302	→	304	-0.232
				297	→	304	0.649
				303	→	310	-0.258
				298	→	304	0.649
				303	→	309	-0.257
				297	→	304	0.231
				303	→	310	0.640
				298	→	304	0.231
				303	→	309	0.640
				293	→	304	0.428
				296	→	304	-0.309
				299	→	305	0.175
				300	→	306	0.175
				301	→	305	0.255
				302	→	306	0.250
				294	→	304	0.427
				295	→	304	-0.314
				299	→	306	0.170
				300	→	305	0.177
				301	→	306	0.252
				302	→	305	0.252

				294	→	304	0.239
				295	→	304	0.618
				301	→	306	0.112
				302	→	305	0.111
				293	→	304	0.234
				296	→	304	0.619
				301	→	305	0.114
				302	→	306	0.111
				293	→	304	0.506
				299	→	305	-0.195
				300	→	306	-0.194
				301	→	305	-0.282
				302	→	306	-0.271
				294	→	304	0.504
				299	→	306	-0.191
				300	→	305	-0.200
				301	→	306	-0.275
				302	→	305	-0.280
				289	→	304	-0.120
				292	→	304	0.670
				290	→	304	-0.115
				291	→	304	0.670
				296	→	304	-0.101
				290	→	304	0.653
				303	→	313	0.161
23	2.8536	434.49	0.024	299	→	304	-0.167
				301	→	304	0.109
				303	→	304	-30.406
				303	→	306	-1.000
				303	→	306	0.720
				303	→	305	0.720
				303	→	307	0.699
				303	→	308	0.700
				299	→	304	0.219
				301	→	304	0.669
				300	→	304	0.236
				302	→	304	0.663
				299	→	304	0.666
				301	→	304	-0.216
				300	→	304	0.658
				302	→	304	-0.232
				297	→	304	0.649
				303	→	310	-0.258
				298	→	304	0.649
				303	→	309	-0.257
				297	→	304	0.231
				303	→	310	0.640
				298	→	304	0.231
				303	→	309	0.640
				293	→	304	0.428
				296	→	304	-0.309

				299	→	305	0.175
				300	→	306	0.175
				301	→	305	0.255
				302	→	306	0.250
				294	→	304	0.427
				295	→	304	-0.314
				299	→	306	0.170
				300	→	305	0.177
				301	→	306	0.252
				302	→	305	0.252
				294	→	304	0.239
				295	→	304	0.618
				301	→	306	0.112
				302	→	305	0.111
				293	→	304	0.234
				296	→	304	0.619
				301	→	305	0.114
				302	→	306	0.111
				293	→	304	0.506
				299	→	305	-0.195
				300	→	306	-0.194
				301	→	305	-0.282
				302	→	306	-0.271
				294	→	304	0.504
				299	→	306	-0.191
				300	→	305	-0.200
				301	→	306	-0.275
				302	→	305	-0.280
				289	→	304	-0.120
				292	→	304	0.670
				290	→	304	-0.115
				291	→	304	0.670
				296	→	304	-0.101
				290	→	304	0.653
				303	→	313	0.161
				289	→	304	0.652
				303	→	314	0.156
24	2.8809	430.36	0.0564	299	→	304	-0.167
				301	→	304	0.109
				303	→	304	-30.406
				303	→	306	-1.000
				303	→	306	0.720
				303	→	305	0.720
				303	→	307	0.699
				303	→	308	0.700
				299	→	304	0.219
				301	→	304	0.669
				300	→	304	0.236
				302	→	304	0.663
				299	→	304	0.666
				301	→	304	-0.216

				300 → 304 0.658<br>302 → 304 -0.232<br>297 → 304 0.649<br>303 → 310 -0.258<br>298 → 304 0.649<br>303 → 309 -0.257<br>297 → 304 0.231<br>303 → 310 0.640<br>298 → 304 0.231<br>303 → 309 0.640<br>293 → 304 0.428<br>296 → 304 -0.309<br>299 → 305 0.175<br>300 → 306 0.175<br>301 → 305 0.255<br>302 → 306 0.250<br>294 → 304 0.427<br>295 → 304 -0.314<br>299 → 306 0.170<br>300 → 305 0.177<br>301 → 306 0.252<br>302 → 305 0.252<br>294 → 304 0.239<br>295 → 304 0.618<br>301 → 306 0.112<br>302 → 305 0.111<br>293 → 304 0.234<br>296 → 304 0.619<br>301 → 305 0.114<br>302 → 306 0.111<br>293 → 304 0.506<br>299 → 305 -0.195<br>300 → 306 -0.194<br>301 → 305 -0.282<br>302 → 306 -0.271<br>294 → 304 0.504<br>299 → 306 -0.191<br>300 → 305 -0.200<br>301 → 306 -0.275<br>302 → 305 -0.280<br>289 → 304 -0.120<br>292 → 304 0.670<br>290 → 304 -0.115<br>291 → 304 0.670<br>296 → 304 -0.101<br>290 → 304 0.653<br>303 → 313 0.161<br>289 → 304 0.652<br>303 → 314 0.156<br>303 → 312 0.668<br>303 → 314 -0.182

25	2.8812	430.32	0	299 → 304 -0.167<br>301 → 304 0.109<br>303 → 304 -30.406<br>303 → 306 -1.000<br>303 → 306 0.720<br>303 → 305 0.720<br>303 → 307 0.699<br>303 → 308 0.700<br>299 → 304 0.219<br>301 → 304 0.669<br>300 → 304 0.236<br>302 → 304 0.663<br>299 → 304 0.666<br>301 → 304 -0.216<br>300 → 304 0.658<br>302 → 304 -0.232<br>297 → 304 0.649<br>303 → 310 -0.258<br>298 → 304 0.649<br>303 → 309 -0.257<br>297 → 304 0.231<br>303 → 310 0.640<br>298 → 304 0.231<br>303 → 309 0.640<br>293 → 304 0.428<br>296 → 304 -0.309<br>299 → 305 0.175<br>300 → 306 0.175<br>301 → 305 0.255<br>302 → 306 0.250<br>294 → 304 0.427<br>295 → 304 -0.314<br>299 → 306 0.170<br>300 → 305 0.177<br>301 → 306 0.252<br>302 → 305 0.252<br>294 → 304 0.239<br>295 → 304 0.618<br>301 → 306 0.112<br>302 → 305 0.111<br>293 → 304 0.234<br>296 → 304 0.619<br>301 → 305 0.114<br>302 → 306 0.111<br>293 → 304 0.506<br>299 → 305 -0.195<br>300 → 306 -0.194<br>301 → 305 -0.282<br>302 → 306 -0.271<br>294 → 304 0.504<br>299 → 306 -0.191

				300 → 305 -0.200<br>301 → 306 -0.275<br>302 → 305 -0.280<br>289 → 304 -0.120<br>292 → 304 0.670<br>290 → 304 -0.115<br>291 → 304 0.670<br>296 → 304 -0.101<br>290 → 304 0.653<br>303 → 313 0.161<br>289 → 304 0.652<br>303 → 314 0.156<br>303 → 312 0.668<br>303 → 314 -0.182<br>303 → 311 0.669<br>303 → 313 -0.179
26	2.932	422.87	0.001	299 → 304 -0.167<br>301 → 304 0.109<br>303 → 304 -30.406<br>303 → 306 -1.000<br>303 → 306 0.720<br>303 → 305 0.720<br>303 → 307 0.699<br>303 → 308 0.700<br>299 → 304 0.219<br>301 → 304 0.669<br>300 → 304 0.236<br>302 → 304 0.663<br>299 → 304 0.666<br>301 → 304 -0.216<br>300 → 304 0.658<br>302 → 304 -0.232<br>297 → 304 0.649<br>303 → 310 -0.258<br>298 → 304 0.649<br>303 → 309 -0.257<br>297 → 304 0.231<br>303 → 310 0.640<br>298 → 304 0.231<br>303 → 309 0.640<br>293 → 304 0.428<br>296 → 304 -0.309<br>299 → 305 0.175<br>300 → 306 0.175<br>301 → 305 0.255<br>302 → 306 0.250<br>294 → 304 0.427<br>295 → 304 -0.314<br>299 → 306 0.170<br>300 → 305 0.177<br>301 → 306 0.252

				302	→	305	0.252
				294	→	304	0.239
				295	→	304	0.618
				301	→	306	0.112
				302	→	305	0.111
				293	→	304	0.234
				296	→	304	0.619
				301	→	305	0.114
				302	→	306	0.111
				293	→	304	0.506
				299	→	305	-0.195
				300	→	306	-0.194
				301	→	305	-0.282
				302	→	306	-0.271
				294	→	304	0.504
				299	→	306	-0.191
				300	→	305	-0.200
				301	→	306	-0.275
				302	→	305	-0.280
				289	→	304	-0.120
				292	→	304	0.670
				290	→	304	-0.115
				291	→	304	0.670
				296	→	304	-0.101
				290	→	304	0.653
				303	→	313	0.161
				289	→	304	0.652
				303	→	314	0.156
				303	→	312	0.668
				303	→	314	-0.182
				303	→	311	0.669
				303	→	313	-0.179
				289	→	304	-0.140
				299	→	306	0.270
				300	→	305	0.284
				301	→	306	-0.135
				302	→	305	-0.156
				303	→	312	0.141
				303	→	314	0.504

27	2.9322	422.84	0	299	→	304	-0.167
				301	→	304	0.109
				303	→	304	-30.406
				303	→	306	-1.000
				303	→	306	0.720
				303	→	305	0.720
				303	→	307	0.699
				303	→	308	0.700
				299	→	304	0.219
				301	→	304	0.669
				300	→	304	0.236
				302	→	304	0.663
				299	→	304	0.666
				301	→	304	-0.216
				300	→	304	0.658
				302	→	304	-0.232
				297	→	304	0.649
				303	→	310	-0.258
				298	→	304	0.649
				303	→	309	-0.257
				297	→	304	0.231
				303	→	310	0.640
				298	→	304	0.231
				303	→	309	0.640
				293	→	304	0.428
				296	→	304	-0.309
				299	→	305	0.175
				300	→	306	0.175
				301	→	305	0.255
				302	→	306	0.250
				294	→	304	0.427
				295	→	304	-0.314
				299	→	306	0.170
				300	→	305	0.177
				301	→	306	0.252
				302	→	305	0.252
				294	→	304	0.239
				295	→	304	0.618
				301	→	306	0.112
				302	→	305	0.111
				293	→	304	0.234
				296	→	304	0.619
				301	→	305	0.114
				302	→	306	0.111
				293	→	304	0.506
				299	→	305	-0.195
				300	→	306	-0.194
				301	→	305	-0.282
				302	→	306	-0.271
				294	→	304	0.504
				299	→	306	-0.191

				300 → 305 -0.200
				301 → 306 -0.275
				302 → 305 -0.280
				289 → 304 -0.120
				292 → 304 0.670
				290 → 304 -0.115
				291 → 304 0.670
				296 → 304 -0.101
				290 → 304 0.653
				303 → 313 0.161
				289 → 304 0.652
				303 → 314 0.156
				303 → 312 0.668
				303 → 314 -0.182
				303 → 311 0.669
				303 → 313 -0.179
				289 → 304 -0.140
				299 → 306 0.270
				300 → 305 0.284
				301 → 306 -0.135
				302 → 305 -0.156
				303 → 312 0.141
				303 → 314 0.504
				290 → 304 -0.141
				299 → 305 0.279
				300 → 306 0.273
				301 → 305 -0.146
				302 → 306 -0.143
				303 → 311 0.144
				303 → 313 0.505

28	3.0643	404.61	0.0244	299 → 304 -0.167<br>301 → 304 0.109<br>303 → 304 -30.406<br>303 → 306 -1.000<br>303 → 306 0.720<br>303 → 305 0.720<br>303 → 307 0.699<br>303 → 308 0.700<br>299 → 304 0.219<br>301 → 304 0.669<br>300 → 304 0.236<br>302 → 304 0.663<br>299 → 304 0.666<br>301 → 304 -0.216<br>300 → 304 0.658<br>302 → 304 -0.232<br>297 → 304 0.649<br>303 → 310 -0.258<br>298 → 304 0.649<br>303 → 309 -0.257<br>297 → 304 0.231<br>303 → 310 0.640<br>298 → 304 0.231<br>303 → 309 0.640<br>293 → 304 0.428<br>296 → 304 -0.309<br>299 → 305 0.175<br>300 → 306 0.175<br>301 → 305 0.255<br>302 → 306 0.250<br>294 → 304 0.427<br>295 → 304 -0.314<br>299 → 306 0.170<br>300 → 305 0.177<br>301 → 306 0.252<br>302 → 305 0.252<br>294 → 304 0.239<br>295 → 304 0.618<br>301 → 306 0.112<br>302 → 305 0.111<br>293 → 304 0.234<br>296 → 304 0.619<br>301 → 305 0.114<br>302 → 306 0.111<br>293 → 304 0.506<br>299 → 305 -0.195<br>300 → 306 -0.194<br>301 → 305 -0.282<br>302 → 306 -0.271<br>294 → 304 0.504<br>299 → 306 -0.191

				300 → 305 -0.200<br>301 → 306 -0.275<br>302 → 305 -0.280<br>289 → 304 -0.120<br>292 → 304 0.670<br>290 → 304 -0.115<br>291 → 304 0.670<br>296 → 304 -0.101<br>290 → 304 0.653<br>303 → 313 0.161<br>289 → 304 0.652<br>303 → 314 0.156<br>303 → 312 0.668<br>303 → 314 -0.182<br>303 → 311 0.669<br>303 → 313 -0.179<br>289 → 304 -0.140<br>299 → 306 0.270<br>300 → 305 0.284<br>301 → 306 -0.135<br>302 → 305 -0.156<br>303 → 312 0.141<br>303 → 314 0.504<br>290 → 304 -0.141<br>299 → 305 0.279<br>300 → 306 0.273<br>301 → 305 -0.146<br>302 → 306 -0.143<br>303 → 311 0.144<br>303 → 313 0.505<br>286 → 304 0.143<br>287 → 304 0.167<br>299 → 306 0.220<br>300 → 305 0.237<br>301 → 306 -0.145<br>302 → 305 -0.167<br>303 → 314 -0.348<br>303 → 315 0.361<br>303 → 320 0.115

29	3.0653	404.48	0	299	→	304	-0.167
				301	→	304	0.109
				303	→	304	-30.406
				303	→	306	-1.000
				303	→	306	0.720
				303	→	305	0.720
				303	→	307	0.699
				303	→	308	0.700
				299	→	304	0.219
				301	→	304	0.669
				300	→	304	0.236
				302	→	304	0.663
				299	→	304	0.666
				301	→	304	-0.216
				300	→	304	0.658
				302	→	304	-0.232
				297	→	304	0.649
				303	→	310	-0.258
				298	→	304	0.649
				303	→	309	-0.257
				297	→	304	0.231
				303	→	310	0.640
				298	→	304	0.231
				303	→	309	0.640
				293	→	304	0.428
				296	→	304	-0.309
				299	→	305	0.175
				300	→	306	0.175
				301	→	305	0.255
				302	→	306	0.250
				294	→	304	0.427
				295	→	304	-0.314
				299	→	306	0.170
				300	→	305	0.177
				301	→	306	0.252
				302	→	305	0.252
				294	→	304	0.239
				295	→	304	0.618
				301	→	306	0.112
				302	→	305	0.111
				293	→	304	0.234
				296	→	304	0.619
				301	→	305	0.114
				302	→	306	0.111
				293	→	304	0.506
				299	→	305	-0.195
				300	→	306	-0.194
				301	→	305	-0.282
				302	→	306	-0.271
				294	→	304	0.504
				299	→	306	-0.191

				300 → 305 -0.200<br>301 → 306 -0.275<br>302 → 305 -0.280<br>289 → 304 -0.120<br>292 → 304 0.670<br>290 → 304 -0.115<br>291 → 304 0.670<br>296 → 304 -0.101<br>290 → 304 0.653<br>303 → 313 0.161<br>289 → 304 0.652<br>303 → 314 0.156<br>303 → 312 0.668<br>303 → 314 -0.182<br>303 → 311 0.669<br>303 → 313 -0.179<br>289 → 304 -0.140<br>299 → 306 0.270<br>300 → 305 0.284<br>301 → 306 -0.135<br>302 → 305 -0.156<br>303 → 312 0.141<br>303 → 314 0.504<br>290 → 304 -0.141<br>299 → 305 0.279<br>300 → 306 0.273<br>301 → 305 -0.146<br>302 → 306 -0.143<br>303 → 311 0.144<br>303 → 313 0.505<br>286 → 304 0.143<br>287 → 304 0.167<br>299 → 306 0.220<br>300 → 305 0.237<br>301 → 306 -0.145<br>302 → 305 -0.167<br>303 → 314 -0.348<br>303 → 315 0.361<br>303 → 320 0.115<br>285 → 304 0.146<br>288 → 304 0.169<br>299 → 305 0.237<br>300 → 306 0.216<br>301 → 305 -0.162<br>302 → 306 -0.144<br>303 → 313 -0.342<br>303 → 316 0.369<br>303 → 319 0.114

30	3.0904	401.19	0.0235	299	→	304	-0.167
				301	→	304	0.109
				303	→	304	-30.406
				303	→	306	-1.000
				303	→	306	0.720
				303	→	305	0.720
				303	→	307	0.699
				303	→	308	0.700
				299	→	304	0.219
				301	→	304	0.669
				300	→	304	0.236
				302	→	304	0.663
				299	→	304	0.666
				301	→	304	-0.216
				300	→	304	0.658
				302	→	304	-0.232
				297	→	304	0.649
				303	→	310	-0.258
				298	→	304	0.649
				303	→	309	-0.257
				297	→	304	0.231
				303	→	310	0.640
				298	→	304	0.231
				303	→	309	0.640
				293	→	304	0.428
				296	→	304	-0.309
				299	→	305	0.175
				300	→	306	0.175
				301	→	305	0.255
				302	→	306	0.250
				294	→	304	0.427
				295	→	304	-0.314
				299	→	306	0.170
				300	→	305	0.177
				301	→	306	0.252
				302	→	305	0.252
				294	→	304	0.239
				295	→	304	0.618
				301	→	306	0.112
				302	→	305	0.111
				293	→	304	0.234
				296	→	304	0.619
				301	→	305	0.114
				302	→	306	0.111
				293	→	304	0.506
				299	→	305	-0.195
				300	→	306	-0.194
				301	→	305	-0.282
				302	→	306	-0.271
				294	→	304	0.504
				299	→	306	-0.191

				300 → 305 -0.200<br>301 → 306 -0.275<br>302 → 305 -0.280<br>289 → 304 -0.120<br>292 → 304 0.670<br>290 → 304 -0.115<br>291 → 304 0.670<br>296 → 304 -0.101<br>290 → 304 0.653<br>303 → 313 0.161<br>289 → 304 0.652<br>303 → 314 0.156<br>303 → 312 0.668<br>303 → 314 -0.182<br>303 → 311 0.669<br>303 → 313 -0.179<br>289 → 304 -0.140<br>299 → 306 0.270<br>300 → 305 0.284<br>301 → 306 -0.135<br>302 → 305 -0.156<br>303 → 312 0.141<br>303 → 314 0.504<br>290 → 304 -0.141<br>299 → 305 0.279<br>300 → 306 0.273<br>301 → 305 -0.146<br>302 → 306 -0.143<br>303 → 311 0.144<br>303 → 313 0.505<br>286 → 304 0.143<br>287 → 304 0.167<br>299 → 306 0.220<br>300 → 305 0.237<br>301 → 306 -0.145<br>302 → 305 -0.167<br>303 → 314 -0.348<br>303 → 315 0.361<br>303 → 320 0.115<br>285 → 304 0.146<br>288 → 304 0.169<br>299 → 305 0.237<br>300 → 306 0.216<br>301 → 305 -0.162<br>302 → 306 -0.144<br>303 → 313 -0.342<br>303 → 316 0.369<br>303 → 319 0.114<br>286 → 304 0.111<br>287 → 304 -0.237<br>297 → 306 0.108

				298 → 305 0.111<br>299 → 306 -0.133<br>300 → 305 -0.158<br>302 → 305 0.118<br>303 → 314 0.165<br>303 → 315 0.536

Table S12: First 20 TDDFT singlet transitions of singlet 2[EtPh] constrained long dimer. B3LYP-D3/6-31+G/PCM(ACN).

Excited State	Energy (eV)	Wavelength (nm)	Oscillator Strength (f)	Transitions
1	0.004	308849.4	0.0001	335 → 336 -8.320<br>335 → 337 0.239<br>335 → 338 -0.121
2	0.8676	1429.01	0.3085	335 → 336 -8.320<br>335 → 337 0.239<br>335 → 338 -0.121<br>335 → 337 0.716
3	1.1854	1045.94	0.601	335 → 336 -8.320<br>335 → 337 0.239<br>335 → 338 -0.121<br>335 → 337 0.716<br>333 → 336 0.105<br>335 → 338 0.714
4	1.7747	698.63	0	335 → 336 -8.320<br>335 → 337 0.239<br>335 → 338 -0.121<br>335 → 337 0.716<br>333 → 336 0.105<br>335 → 338 0.714<br>335 → 339 0.703
5	1.8939	654.67	0	335 → 336 -8.320<br>335 → 337 0.239<br>335 → 338 -0.121<br>335 → 337 0.716<br>333 → 336 0.105<br>335 → 338 0.714<br>335 → 339 0.703<br>335 → 340 0.697
6	1.9542	634.46	0.0335	335 → 336 -8.320<br>335 → 337 0.239<br>335 → 338 -0.121<br>335 → 337 0.716<br>333 → 336 0.105<br>335 → 338 0.714<br>335 → 339 0.703<br>335 → 340 0.697<br>334 → 336 0.705

7	2.0751	597.48	0.0562	335 → 336 -8.320<br>335 → 337 0.239<br>335 → 338 -0.121<br>335 → 337 0.716<br>333 → 336 0.105<br>335 → 338 0.714<br>335 → 339 0.703<br>335 → 340 0.697<br>334 → 336 0.705<br>332 → 336 0.702
8	2.1802	568.67	0.5647	335 → 336 -8.320<br>335 → 337 0.239<br>335 → 338 -0.121<br>335 → 337 0.716<br>333 → 336 0.105<br>335 → 338 0.714<br>335 → 339 0.703<br>335 → 340 0.697<br>334 → 336 0.705<br>332 → 336 0.702<br>333 → 336 0.695<br>335 → 338 -0.115
9	2.3057	537.72	0.0035	335 → 336 -8.320<br>335 → 337 0.239<br>335 → 338 -0.121<br>335 → 337 0.716<br>333 → 336 0.105<br>335 → 338 0.714<br>335 → 339 0.703<br>335 → 340 0.697<br>334 → 336 0.705<br>332 → 336 0.702<br>333 → 336 0.695<br>335 → 338 -0.115<br>331 → 336 0.702
10	2.3466	528.37	0.0195	335 → 336 -8.320<br>335 → 337 0.239<br>335 → 338 -0.121<br>335 → 337 0.716<br>333 → 336 0.105<br>335 → 338 0.714<br>335 → 339 0.703<br>335 → 340 0.697<br>334 → 336 0.705<br>332 → 336 0.702<br>333 → 336 0.695<br>335 → 338 -0.115<br>331 → 336 0.702<br>329 → 336 0.686<br>330 → 336 -0.142

11	2.3763	521.76	0.6389	335 → 336 -8.320<br>335 → 337 0.239<br>335 → 338 -0.121<br>335 → 337 0.716<br>333 → 336 0.105<br>335 → 338 0.714<br>335 → 339 0.703<br>335 → 340 0.697<br>334 → 336 0.705<br>332 → 336 0.702<br>333 → 336 0.695<br>335 → 338 -0.115<br>331 → 336 0.702<br>329 → 336 0.686<br>330 → 336 -0.142<br>329 → 336 0.143<br>330 → 336 0.682
12	2.5664	483.11	0.0005	335 → 336 -8.320<br>335 → 337 0.239<br>335 → 338 -0.121<br>335 → 337 0.716<br>333 → 336 0.105<br>335 → 338 0.714<br>335 → 339 0.703<br>335 → 340 0.697<br>334 → 336 0.705<br>332 → 336 0.702<br>333 → 336 0.695<br>335 → 338 -0.115<br>331 → 336 0.702<br>329 → 336 0.686<br>330 → 336 -0.142<br>329 → 336 0.143<br>330 → 336 0.682<br>332 → 337 -0.219<br>335 → 341 0.665

13	2.586	479.44	0.0664	335 → 336 -8.320<br>335 → 337 0.239<br>335 → 338 -0.121<br>335 → 337 0.716<br>333 → 336 0.105<br>335 → 338 0.714<br>335 → 339 0.703<br>335 → 340 0.697<br>334 → 336 0.705<br>332 → 336 0.702<br>333 → 336 0.695<br>335 → 338 -0.115<br>331 → 336 0.702<br>329 → 336 0.686<br>330 → 336 -0.142<br>329 → 336 0.143<br>330 → 336 0.682<br>332 → 337 -0.219<br>335 → 341 0.665<br>328 → 336 0.697
14	2.5944	477.89	0.0006	335 → 336 -8.320<br>335 → 337 0.239<br>335 → 338 -0.121<br>335 → 337 0.716<br>333 → 336 0.105<br>335 → 338 0.714<br>335 → 339 0.703<br>335 → 340 0.697<br>334 → 336 0.705<br>332 → 336 0.702<br>333 → 336 0.695<br>335 → 338 -0.115<br>331 → 336 0.702<br>329 → 336 0.686<br>330 → 336 -0.142<br>329 → 336 0.143<br>330 → 336 0.682<br>332 → 337 -0.219<br>335 → 341 0.665<br>328 → 336 0.697<br>330 → 337 0.119<br>332 → 337 0.651<br>335 → 341 0.201

15	2.6351	470.5	0.0004	335	→	336	-8.320
				335	→	337	0.239
				335	→	338	-0.121
				335	→	337	0.716
				333	→	336	0.105
				335	→	338	0.714
				335	→	339	0.703
				335	→	340	0.697
				334	→	336	0.705
				332	→	336	0.702
				333	→	336	0.695
				335	→	338	-0.115
				331	→	336	0.702
				329	→	336	0.686
				330	→	336	-0.142
				329	→	336	0.143
				330	→	336	0.682
				332	→	337	-0.219
				335	→	341	0.665
				328	→	336	0.697
				330	→	337	0.119
				332	→	337	0.651
				335	→	341	0.201
				325	→	336	0.111
				327	→	336	0.160
				333	→	338	-0.106
				334	→	338	0.663

16	2.6671	464.86	0.001	335	→	336	-8.320
				335	→	337	0.239
				335	→	338	-0.121
				335	→	337	0.716
				333	→	336	0.105
				335	→	338	0.714
				335	→	339	0.703
				335	→	340	0.697
				334	→	336	0.705
				332	→	336	0.702
				333	→	336	0.695
				335	→	338	-0.115
				331	→	336	0.702
				329	→	336	0.686
				330	→	336	-0.142
				329	→	336	0.143
				330	→	336	0.682
				332	→	337	-0.219
				335	→	341	0.665
				328	→	336	0.697
				330	→	337	0.119
				332	→	337	0.651
				335	→	341	0.201
				325	→	336	0.111
				327	→	336	0.160
				333	→	338	-0.106
				334	→	338	0.663
				325	→	336	0.176
				326	→	336	0.107
				327	→	336	0.645
				334	→	338	-0.173

17	2.6882	461.22	0.0124	335 → 336 -8.320<br>335 → 337 0.239<br>335 → 338 -0.121<br>335 → 337 0.716<br>333 → 336 0.105<br>335 → 338 0.714<br>335 → 339 0.703<br>335 → 340 0.697<br>334 → 336 0.705<br>332 → 336 0.702<br>333 → 336 0.695<br>335 → 338 -0.115<br>331 → 336 0.702<br>329 → 336 0.686<br>330 → 336 -0.142<br>329 → 336 0.143<br>330 → 336 0.682<br>332 → 337 -0.219<br>335 → 341 0.665<br>328 → 336 0.697<br>330 → 337 0.119<br>332 → 337 0.651<br>335 → 341 0.201<br>325 → 336 0.111<br>327 → 336 0.160<br>333 → 338 -0.106<br>334 → 338 0.663<br>325 → 336 0.176<br>326 → 336 0.107<br>327 → 336 0.645<br>334 → 338 -0.173<br>326 → 336 0.684<br>327 → 336 -0.136

18	2.6965	459.79	0.0068	335	→	336	-8.320
				335	→	337	0.239
				335	→	338	-0.121
				335	→	337	0.716
				333	→	336	0.105
				335	→	338	0.714
				335	→	339	0.703
				335	→	340	0.697
				334	→	336	0.705
				332	→	336	0.702
				333	→	336	0.695
				335	→	338	-0.115
				331	→	336	0.702
				329	→	336	0.686
				330	→	336	-0.142
				329	→	336	0.143
				330	→	336	0.682
				332	→	337	-0.219
				335	→	341	0.665
				328	→	336	0.697
				330	→	337	0.119
				332	→	337	0.651
				335	→	341	0.201
				325	→	336	0.111
				327	→	336	0.160
				333	→	338	-0.106
				334	→	338	0.663
				325	→	336	0.176
				326	→	336	0.107
				327	→	336	0.645
				334	→	338	-0.173
				326	→	336	0.684
				327	→	336	-0.136
				323	→	336	-0.454
				324	→	336	0.520

				335	→	336	-8.320
				335	→	337	0.239
				335	→	338	-0.121
				335	→	337	0.716
				333	→	336	0.105
				335	→	338	0.714
				335	→	339	0.703
				335	→	340	0.697
				334	→	336	0.705
				332	→	336	0.702
				333	→	336	0.695
				335	→	338	-0.115
				331	→	336	0.702
				329	→	336	0.686
				330	→	336	-0.142
				329	→	336	0.143
				330	→	336	0.682
				332	→	337	-0.219
				335	→	341	0.665
19	2.697	459.71	0.0001	328	→	336	0.697
				330	→	337	0.119
				332	→	337	0.651
				335	→	341	0.201
				325	→	336	0.111
				327	→	336	0.160
				333	→	338	-0.106
				334	→	338	0.663
				325	→	336	0.176
				326	→	336	0.107
				327	→	336	0.645
				334	→	338	-0.173
				326	→	336	0.684
				327	→	336	-0.136
				323	→	336	-0.454
				324	→	336	0.520
				325	→	336	0.636
				326	→	336	-0.128
				327	→	336	-0.150
				335	→	344	0.184

20	2.7013	458.98	0.015	335 → 336 -8.320<br>335 → 337 0.239<br>335 → 338 -0.121<br>335 → 337 0.716<br>333 → 336 0.105<br>335 → 338 0.714<br>335 → 339 0.703<br>335 → 340 0.697<br>334 → 336 0.705<br>332 → 336 0.702<br>333 → 336 0.695<br>335 → 338 -0.115<br>331 → 336 0.702<br>329 → 336 0.686<br>330 → 336 -0.142<br>329 → 336 0.143<br>330 → 336 0.682<br>332 → 337 -0.219<br>335 → 341 0.665<br>328 → 336 0.697<br>330 → 337 0.119<br>332 → 337 0.651<br>335 → 341 0.201<br>325 → 336 0.111<br>327 → 336 0.160<br>333 → 338 -0.106<br>334 → 338 0.663<br>325 → 336 0.176<br>326 → 336 0.107<br>327 → 336 0.645<br>334 → 338 -0.173<br>326 → 336 0.684<br>327 → 336 -0.136<br>323 → 336 -0.454<br>324 → 336 0.520<br>325 → 336 0.636<br>326 → 336 -0.128<br>327 → 336 -0.150<br>335 → 344 0.184<br>323 → 336 0.520<br>324 → 336 0.456

References

(1) Modjewski, M.; Lindeman, S. V.; Rathore, R. A Versatile Preparation of Geländer-Type *p* - Terphenyls from a Readily Available Diacetylenic Precursor. *Org. Lett.* **2009**, *11* (20), 4656–4659. https://doi.org/10.1021/ol901938f.

(2) Evans, R.; Dal Poggetto, G.; Nilsson, M.; Morris, G. A. Improving the Interpretation of Small Molecule Diffusion Coefficients. *Anal. Chem.* **2018**, *90* (6), 3987–3994. https://doi.org/10.1021/acs.analchem.7b05032.

(3) Hamburger, R.; Rumble, C.; Young, E. R. An Introduction to Processing, Fitting, and Interpreting Transient Absorption Data. **2024**.
